# Trends in benzene inverse sandwich complexes of the alkaline-earth metals Mg, Ca, Sr and Ba

**DOI:** 10.1039/d5sc05373k

**Published:** 2025-08-29

**Authors:** Dawid Jędrzkiewicz, Michael Morasch, Oliver P. E. Townrow, Bastian Rösch, Jens Langer, Zachary Mathe, Sjoerd Harder

**Affiliations:** a Inorganic and Organometallic Chemistry, Friedrich-Alexander-Universität Erlangen-Nürnberg Egerlandstraße 1 91058 Erlangen Germany dawid.jedrzkiewicz@gmail.com sjoerd.harder@fau.de; b Institute of Nanotechnology, Karlsruher Institut für Technologie Hermann-von-Helmholtz-Platz 1 76344 Eggenstein-Leopoldshafen Germany; c Department of Inorganic Spectroscopy, Max Planck Institute for Chemical Energy Conversion Stiftstraße 34-36 45470 Mülheim an der Ruhr Germany

## Abstract

Mechanochemical reduction of β-diketiminate (BDI) barium iodide precursors with K/KI resulted in the first barium inverse sandwich complexes containing the benzene dianion in yields of up to 54%. This most challenging isolation of highly reactive (BDI)Ba–(C_6_H_6_)–Ba(BDI) complexes, completes the family of heavier benzene inverse sandwich complexes and allows for a comparison of trends in the series from Mg, Ca, Sr to Ba. Syntheses, stabilities, structures, electronic states and reactivities of the full range are compared. Crucial for isolation of the Ba inverse sandwich complexes are the *t*Bu-substituents in the ligand backbone which push the bulky aryl rings towards the large Ba metal cations. These secondary Ba⋯(π-Ar) interactions result in an unexpected high stability. Another trend is found for the ring puckering in the bridging benzene^2−^ dianion which steadily increases from Ba to Mg. DFT calculations show the general ionic character of (BDI)Ae–(C_6_H_6_)–Ae(BDI) complexes (Ae = Mg, Ca, Sr, Ba) and reveal only small energy differences between closed-shell singlet or open-shell triplet states. The most reactive (BDI)Ba–(C_6_H_6_)–Ba(BDI) complexes could be considered the first Ba^I^ synthons. They reduce a range of polyaromatic hydrocarbons, H_2_ or even convert (BDI)MgI precursors into well-known (BDI)Mg–Mg(BDI) complexes. Reactions with heavier (BDI)AeI (Ae = Ca, Sr) gave (BDI)Ae–(C_6_H_6_)–Ae(BDI) and (BDI)BaI.

## Abstract 2

The challenging isolation of unique barium benzene inverse sandwich complexes was achieved by solid-state mechanochemical reduction. These first Ba(i) synthons allow for a precise comparison of trends in the series from Mg, Ca, Sr to Ba.

## Graphical abstract

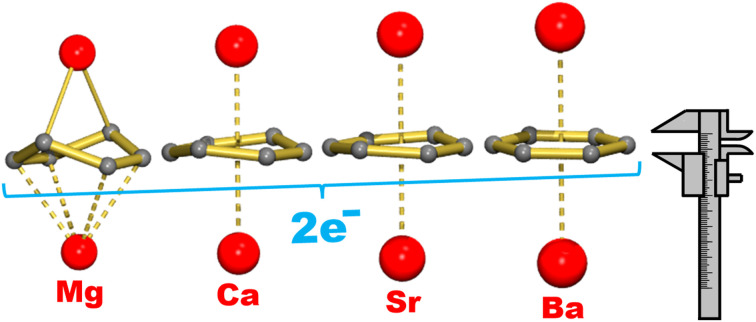

## Introduction

**Scheme 1:**
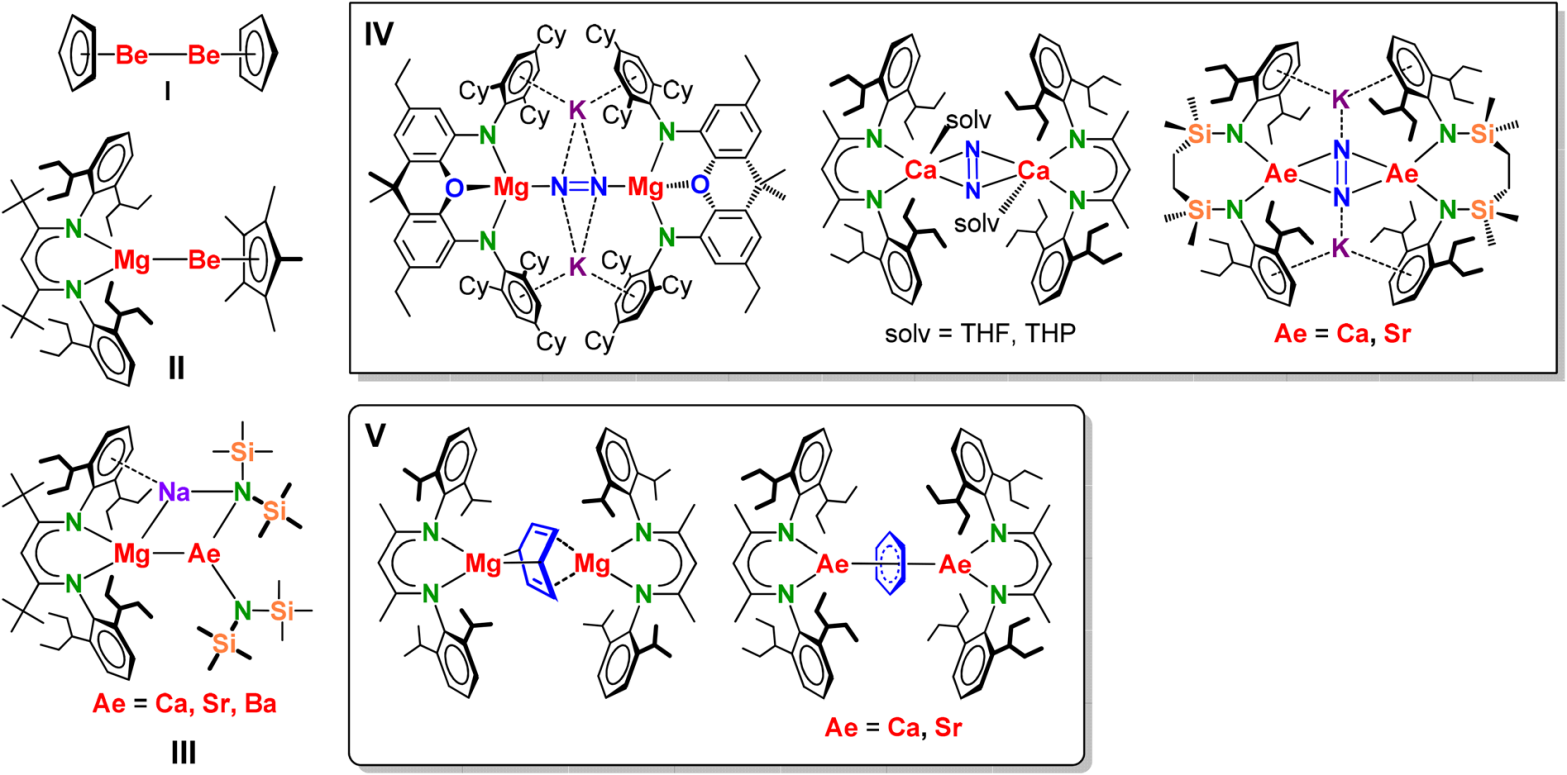
Recently reported compounds containing Ae–Ae or Mg–Ae bonds (I–III) and examples of Ae^I^ synthons with N_2_^2−^ (IV) or C_6_H_6_^2−^ (V) electron reservoirs.

Recent developments in low oxidation state alkaline earth metal (Ae) chemistry have expanded beyond the well-established dimeric magnesium(i) complexes that find widespread applications as precise reducing agents.^1–4^ The most significant synthetic milestones include isolation of the first complex with a Be^I^–Be^I^ bond (I, Scheme 1),^5^ unique Mg^0^ complexes,^6^ and a range of derivatives featuring heterobimetallic Mg–Be, Mg–Ca, Mg–Sr, and Mg–Ba bonding (II–III, Scheme 1).^7,8^ Based on the decreasing electronegativity along the row Be > Mg > Ca > Sr > Ba, the formal oxidation states in II are Mg^II^–Be^0^ whereas those in III are Mg^0^–Ae^II^. Examples of heavier, homometallic Ae^I^–Ae^I^ complexes are hitherto unknown. Computational analysis predicts that the Ca–Ca bond is much weaker than existing Be–Be and Mg–Mg bonds, making it too reactive to be isolable.^9–12^ This trend is expected to continue down the group.^13,16^ Nonetheless, the reactivity of heavier Ae^I^ complexes can be studied by means of Ae^I^ synthons, *i.e.* formal Ae^II^ complexes showing the reactivity of electron-rich Ae^I^ reagents. Whereas complexes with Be–Be or Mg–Mg bonds have a non-nuclear attractor (NNA), *i.e.* a local maximum of electron density, at the centre of the Be–Be and Mg–Mg axes, Ae^I^ synthons typically feature a molecular 2e^−^ reservoir that bridges the two Ae^II^ centres. Examples of such electron reservoirs include the dinitrogen dianion N_2_^2−^ (IV, Scheme 1)^9,14–16^ and the benzene dianion C_6_H_6_^2−^ (V, Scheme 1).^9,17–20^ Both of these dianions release their additional electrons easily, which makes them very strong reductants but they can also act as Brønsted bases or nucleophiles.^6,18,19^

The strongly reducing character of these Ae^I^ synthons is determined by the very negative reduction potentials of N_2_ or C_6_H_6_. However, we found that the metals also have a strong influence. Their reactivities sharply increase with the size of the Ae metal. Longer and more ionic Ae–(N_2_)–Ae or Ae–(C_6_H_6_)–Ae bonds facilitate 2e^−^ loss but they are also weaker and more dynamic which impedes complex stability by ligand exchange reactions.^16,18^ Complexes with the larger Ae metals need to be stabilised by superbulky β-diketiminate (BDI) ligands.^9,21^ The replacement of the DIPP-substituents (DIPP = 2,6-iPr-phenyl) in the popular ^DIPP^BDI ligand for much bulkier DIPeP-moieties in ^DIPeP^BDI (DIPeP = 2,6-CHEt_2_-phenyl) allowed for the synthesis of the first Ca dinitrogen complex.^9^ The additional advantage of the DIPeP-substituent is its solubilizing property which makes the final complexes even soluble in inert alkane solvents. Although successful in Ca chemistry, the same ^DIPeP^BDI ligand was insufficiently bulky for stabilisation of a similar Sr dinitrogen complex.^16^ However, using a dianionic bis-amide ligand with DIPeP-substituents enabled the isolation of a first Sr dinitrogen complex.^16^ The two additional K^+^ cations generate together with two Sr^2+^ cations a metal crown that efficiently encapsulates the N_2_^2−^ dianion (IV). Such complexes are reminiscent of K-aluminyl chemistry^22^ and Mulvey's inverse crown ethers.^23^

To our knowledge, highly reducing Ba^I^ synthons are hitherto non-existent. We recently reported a Ba–anthracene complex but considerable electron delocalisation in the anthracene dianion makes such complexes much more stable and less reducing.^24^ We now turn our attention to the challenging synthesis of a highly reactive Ba benzene complex. Complexes with this largest group 2 metal need stabilisation by our most bulky BDI ligand: ^DIPeP^BDI*. In this ligand, which also has been used in the synthesis of Mg^0^ complexes (II, III),^6,8^ the Me-backbone substituents have been replaced for large *t*Bu-groups that direct the DIPeP rings towards the metal centre.^25^ Herein, we present a hybrid approach combining solid-state mechanochemical and solution methods to access Ba benzene inverse sandwich complexes. Completing the series of (BDI)Ae–(C_6_H_6_)–Ae(BDI) complexes (Ae = Mg, Ca, Sr, Ba), this now allows for discussion of trends in structure, electronics and reactivity.

## Results and discussion

### Syntheses and solution studies

**Scheme 2:**
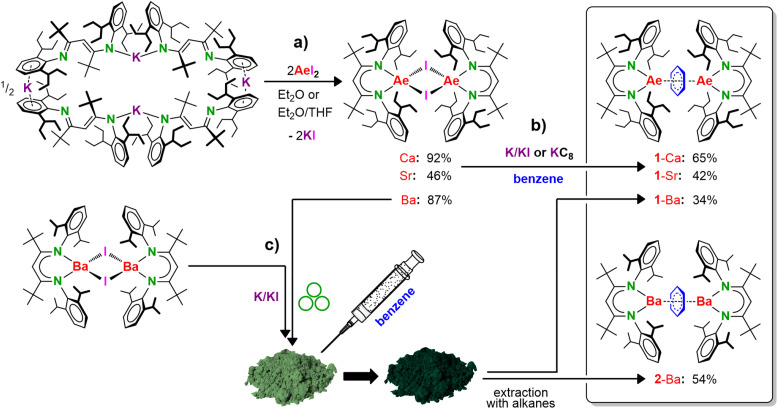
(a) Syntheses of the precursors [(^DIPeP^BDI*)Ae(μ-I)]_2_ (Ae = Ca, Sr, Ba). (b) Synthesis of Ae benzene inverse sandwich complexes by classical reduction in solution (1-Ca, 1-Sr). (c) Synthesis of Ba benzene inverse sandwich complexes by a hybrid solid-state/solution synthesis (1-Ba, 2-Ba).

Following a salt-metathesis protocol, a series of Ae iodide precursors [(^DIPeP^BDI*)Ae(μ-I)]_2_ (Ae = Ca, Sr, Ba) was prepared from (^DIPeP^BDI*)K and the corresponding AeI_2_ in yields of 92%, 46% and 87%, respectively (Scheme 2a and Fig. S6–S24, S97–S100). The potassium precursor was obtained in quantitative yield by deprotonation of ^DIPeP^BDI*–H with benzyl potassium and crystallised as the solvent-free cyclic tetramer [(^DIPeP^BDI*)K]_4_ (SI: Fig. S1–S5 and S99).

Reduction of [(^DIPeP^BDI*)Ae(μ-I)]_2_ (Ae = Ca, Sr) with 5% w/w K/KI^26^ or KC_8_ in benzene (Scheme 2b) led to the target compounds [(^DIPeP^BDI*)Ae]_2_(η^6^:η^6^-C_6_H_6_) (1-Ae, Ae = Ca or Sr) in reasonable yields (1-Ca: 65%, 1-Sr: 42%). These yields are high when compared to those for similar complexes with the less bulky ^DIPeP^BDI ligand (Ca: 35%, Sr: 26%).^9,18^ The better yields for 1-Ae are due to more efficient steric protection and the lower solubility facilitating crystallisation.

The reduction of [(^DIPeP^BDI*)Ba(μ-I)]_2_ with K/KI in benzene was found to be more problematic. Within two hours at room temperature, the Ba benzene inverse sandwich [(^DIPeP^BDI*)Ba]_2_(η^6^:η^6^-C_6_H_6_) (1-Ba) had been formed selectively. However, the complex is highly soluble and upon removal of the benzene solvent under vacuum, it decomposed into a mixture in which mainly homoleptic species (^DIPeP^BDI*)_2_Ba and small amounts of biphenyl were identified (Fig. S97). A similar phenomenon has been observed previously for (^DIPeP^BDI)Sr–(C_6_H_6_)–Sr(^DIPeP^BDI) (V). Highly concentrated benzene solutions led to biphenyl formation which could only be suppressed at low temperature.^18^ However, switching to the even more reactive Ba complexes, even concentration of the benzene solution by freeze-drying at −15 °C did not prevent product decomposition. Using an apolar aliphatic solvent with a small amount of benzene could enable product isolation by crystallisation, circumventing the need for solvent removal. However, this resulted in poor product selectivity. Lowering the temperature to 0 °C increased the selectivity, but drastically elongated reaction times.

Since a ball-milling approach has previously been very successful in Mg and Ca low-oxidation-state chemistry,^18,29,30^ we tried a hybrid solid-state/solution synthesis. The optimised protocol is as follows: [(^DIPeP^BDI*)Ba(μ-I)]_2_ was ball-milled with a 4-fold excess of 5% K/KI for 30 minutes. After addition of a small amount of benzene, the resulting powder changed its colour from light-green to black, indicating formation of the C_6_H_6_^2−^ dianion. Subsequently, the product was extracted with *n*-pentane. Cooling the pentane solution to −35 °C led to crystallisation of [(^DIPeP^BDI*)Ba]_2_(η^6^:η^6^-C_6_H_6_) (1-Ba) in 34% yield (Scheme 2c). This procedure also proved effective for reduction of the known [(^DIPP^BDI*)Ba(μ-I)]_2_ precursor.^27^ Due to its lower solubility in hydrocarbons, the product [(^DIPP^BDI*)Ba]_2_(η^6^:η^6^-C_6_H_6_) (2-Ba) crystallised much faster than 1-Ba, resulting in a higher yield of 54%.

The new complexes 1-Ae (Ca, Sr, Ba) and 2-Ba extend the family of known Ae benzene inverse sandwich complexes (Table 1) which now also includes the heaviest, least stable and most reactive Ba complexes. This allows for a comprehensive evaluation of trends.

**Table 1:** Stability of complexes of Ae benzene inverse sandwich complexes (BDI)Ae–(C_6_H_6_)–Ae(BDI) with BDI ligands of various bulk. Shown are the times for full decomposition monitored by ^1^H NMR spectroscopy in cyclohexane at room temperature, unless stated otherwise

Ae	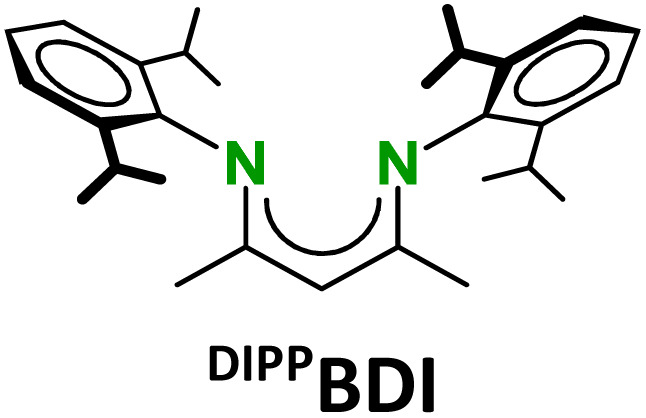	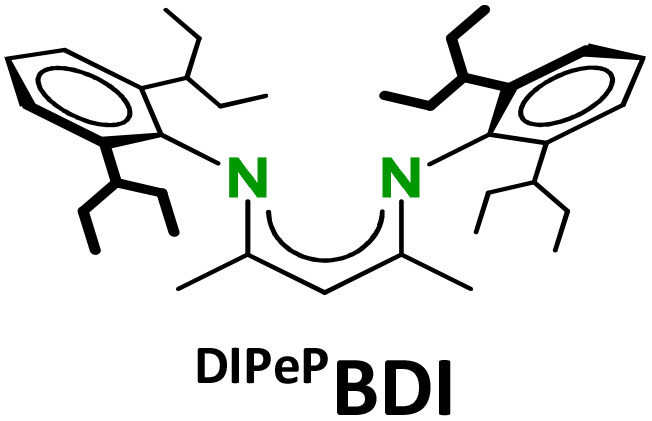	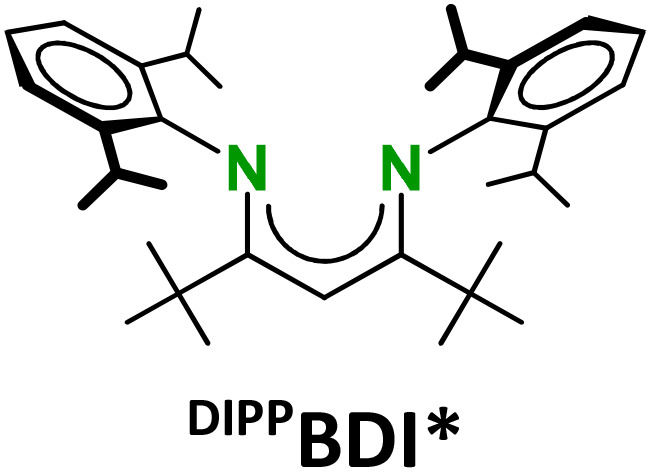	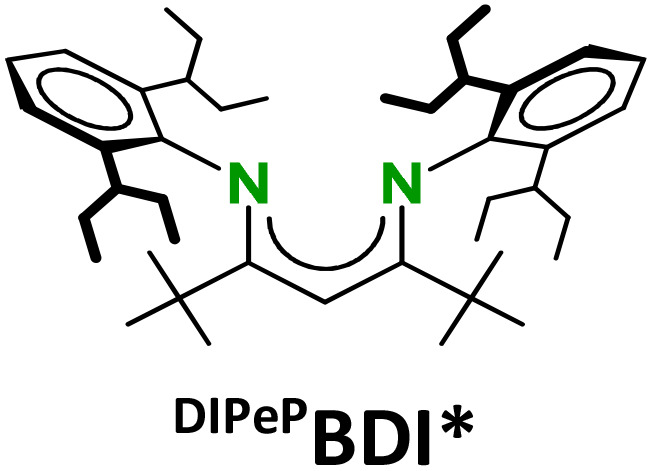
Mg	12 h^a^^,^^20^	3 days^b^^,^^17^	—d	—d
Ca	30 min^c^^,^^18^	2 days	—d	20 days
Sr	—d	1 day	—d	8 days
Ba	—d	—d	13 days	15 days

a In benzene at 60 °C.

b In toluene at 120 °C.

c Compound not isolated but generated *in situ* in benzene.

d Compound not obtained.

NMR data for the new complexes are shown in the SI (Fig. S25–S44 and S79). In all cases, the BDI* signals could be fully assigned. However, dianionic C_6_H_6_^2−^ remained silent, also upon cooling to −80 °C. Also in ^13^C NMR spectra no signals for the benzene dianion were observed. This is even the case in complexes with 99% ^13^C-labeled benzene. It is of interest to note that for all Ca, Sr and Ba benzene complexes reported so far, we have never observed NMR signals corresponding to the bridging C_6_H_6_^2−^ dianion.^9,18^ As discussed previously, this is likely due to a narrow singlet-triplet gap which allows thermal population of the open-shell state causing paramagnetic behaviour (see SI for details). Preliminary electron paramagnetic resonance (EPR) spectra of ^13^C_6_H_6_-bridged complexes also support the assignments of benzene dianions with triplet character (Fig. S108). In contrast, both Mg benzene inverse sandwich complexes show signals for the benzene dianion, which is likely due to their different nature. Whereas Ca, Sr and Ba complexes are highly ionic, Mg complexes have a significant covalent bonding component, resulting in a metalla-norbornadiene-like structure (Scheme 1, V).

While Ae benzene inverse sandwich complexes are at room temperature stable in the solid state, in solution they show only limited lifetimes. Direct comparison (Table 1) shows that their stabilities generally decrease with increasing metal size or decreasing ligand bulk. The family of complexes includes four different ligands with either DIPP- or the bulkier DIPeP-substituents (^DIPP^BDI or ^DIPeP^BDI) and backbone Me- or *t*Bu-substituents (BDI or BDI*). Although the latter backbone substituents are far away from the metal centre, they have a considerable influence on steric shielding by pushing the N–Ar groups towards the remote metal.^27,28^ Especially the BDI*-based inverse sandwich complexes persist for weeks in alkane solutions at room temperature. There is also a clear correlation between complex stability and the size of the Ae metal. Using small ligands like ^DIPP^BDI or ^DIPeP^BDI, the Ca and Sr complexes are increasingly unstable and the Ba complex could not even be obtained. The minimal ligand size required to stabilise the Ae–[C_6_H_6_]–Ae core: ^DIPP^BDI for Mg,^17^^DIPeP^BDI for Ca, Sr,^18^ and ^DIPP^BDI* for Ba. Only with the larger set of BDI* ligands we have been able to isolate Ba benzene inverse sandwich complexes. With the largest ligand, ^DIPeP^BDI*, complex stability decreases from Ca to Sr but the Ba complex shows a surprisingly high stability and only fully decomposed after 15 days.

As described elsewhere for decomposition of Ca and Sr benzene inverse sandwich complexes,^9,18^ also the Ba complexes 1-Ba and 2-Ba decompose by release of benzene and form a mixture of unknown species (Fig. S80–S82). The only identified products of decomposition are homoleptic Ba species. Some of the signals in ^1^H NMR spectra of decomposed 1-Ba and 2-Ba (Fig. S82 and S83) match those of homoleptic [(^DIPeP^BDI*)_2_Ba] and [(^DIPP^BDI*)_2_Ba] (Fig. S63 and S69), which we prepared for comparison according to alternative synthetic routes (see SI for details). We were not able to isolate or identify any products related to reductive C–H bond activation in the ligand, as observed previously in the decomposition of Ca and Sr benzene inverse sandwich complexes.^9,18^ The stability of 1-Ba and 2-Ba in benzene solution will be described below in the context reactivity studies.

### Crystal structures

**Fig. 1:**
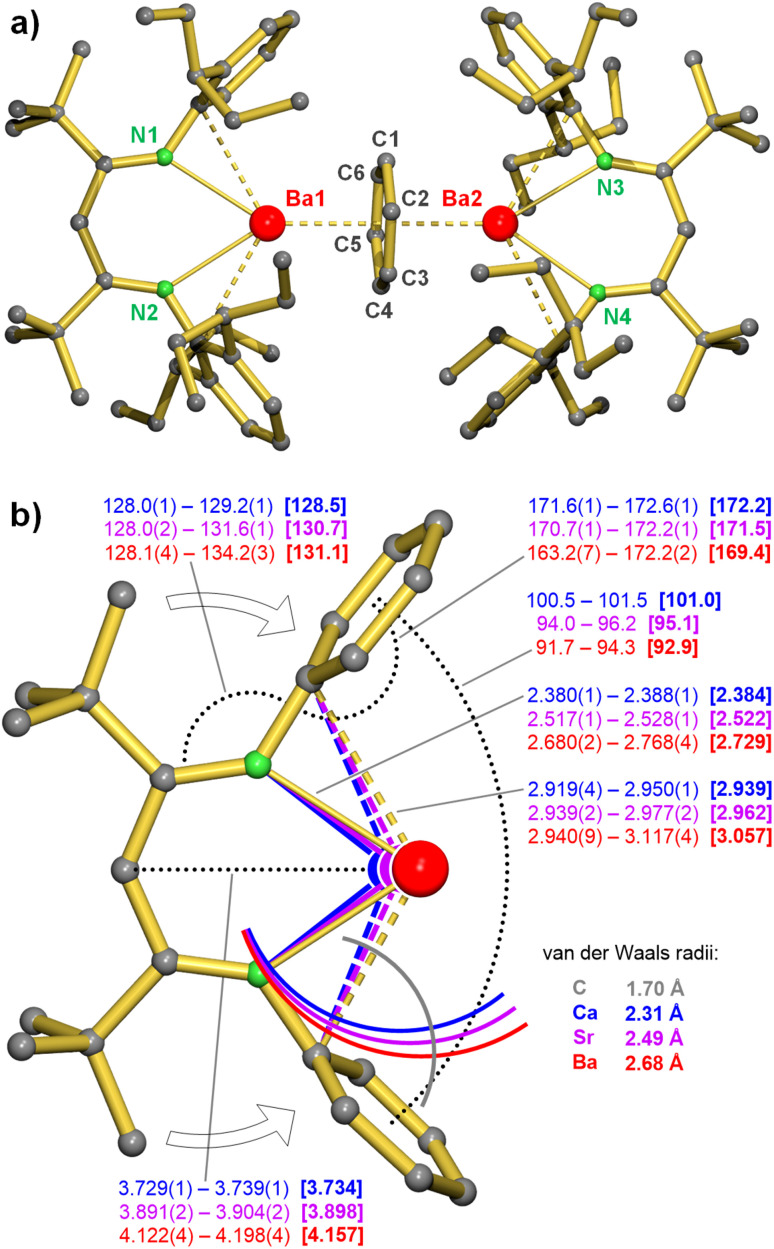
(a) Crystal structure of 1-Ba; H atoms omitted for clarity. (b) Selected bond distances (Å) and angles (°) in 1-Ca (blue), 1-Sr (purple) and 1-Ba (red). The penetrating van der Waals radii around the Ae metals and C_*ipso*_ indicate bonding secondary Ae⋯C_*ipso*_(Ar) π-interactions.

The molecular structures of the BDI complexes 1-Ca, 1-Sr and 1-Ba (Fig. 1 and S104–S106) are similar to those of previously reported Ca, Sr sandwich complexes^9,18^ and also show similarities to corresponding Yb or Sm complexes of a similar build-up.^31^ They are all binuclear metal complexes with bidentate BDI ligands and η^6^:η^6^-bridging benzene moieties. However, there are marked structural differences and trends.

Although the BDI ligand is generally described as a bidentate ligand, the structure of 1-Ba shows significant distortions (Fig. 1a). The aryl rings in the ^DIPeP^BDI* ligand are clearly bend to the Ba metal. This is evident from the N–C_*ipso*_⋯C_*para*_ angles which are ideally 180° but in 1-Ba takes on values ranging from 163.2(7)° to 172.2(2)° (average: 169.4°). This distortion is less extreme for 1-Sr (average: 171.5°) and 1-Ca (average: 172.2°). Also the C_β_–N–C_*ipso*_ angles are clearly distorted from the idealised 120° angle and increase from 1-Ca (average: 128.5°) to 1-Sr (average: 130.7°) to 1-Ba (average: 131.1°). The two Ar rings span a cavity in which the metal is bound through additional Ae⋯C(Ar) π-interactions. The latter interactions gain importance with metal size: Ca < Sr < Ba. This is evident from the angles between the Ar planes decreasing down the group: 1-Ca (101.5°) > 1-Sr (95.1°) > 1-Ba (92.9°). These interactions are partially due to the bulky *t*Bu-substituents in the ligand backbone, pushing the Ar ring towards the metal. For comparison, the same ligand with backbone Me-substituents, ^DIPeP^BDI, shows much larger pockets with following angles between the Ar planes: Mg 141.8°,^17^ Ca 122.8°, Sr 131.8°. This demonstrates that the BDI* ligands with small metal pockets are superior in stabilisation of larger metals like Ca, Sr and especially Ba. Any Ae⋯C(π) distance below 3.13 Å (Ca), 3.30 Å (Sr) or 3.48 Å (Ba) could be considered a bonding interaction.^32^ Especially in saturating the large electropositive Ba^2+^ cation, such secondary interactions play a pivotal role and they can even compete with Ba–THF coordination.^33^ This may explain why the Ba benzene inverse sandwich complexes 1-Ba and 2-Ba are unexpectedly stable towards thermal decomposition (*vide supra*, Table 1). Since 1-Ba and 2-Ba are equally stable, these secondary Ae⋯C(Ar) π-interactions seem to outweigh the shielding advantage of Et_2_CH- *versus* Me_2_CH-substituents.

**Fig. 2:**
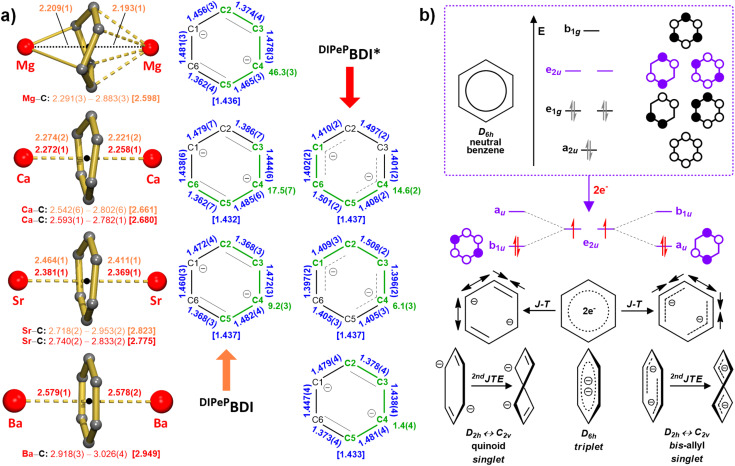
(a) Structures and selected geometric parameters for the Ae–(C_6_H_6_)–Ae moieties in Ae benzene inverse sandwich complexes stabilised by ^DIPeP^BDI or ^DIPeP^BDI* ligands (bond lengths/distances in Å and angles in °). The Ae–C and Ae–centroid distances are marked in red (for ^DIPeP^BDI* complexes) or in orange (for ^DIPeP^BDI complexes). The C–C bond lengths are shown in blue. The largest C–C–C–C torsion angles are marked in green. (b) Molecular orbital diagram for benzene and the effect of 2e^−^ reduction and Jahn–Teller distortions (1st and 2nd).

Apart from differences in the BDI ligand geometry, there are also clear trends in Ae–(C_6_H_6_)–Ae bonding. Whereas the bridging C_6_H_6_^2−^ ring in the Mg complexes is severely puckered, it gradually flattens going the larger Ae metals (Fig. 2a). It can be quantified by the values of the C–C–C–C torsion angles in the benzene ring which range from to 46.3(3)° for the Mg complex down to 1.4(4)° for 1-Ba. Flattening of the bridging benzene dianion may be explained by the increasing ionicity of the Ae–(C_6_H_6_) bond combined with the increasing size and softness of the Ae^2+^ cation. The hard Mg^2+^ cation is significantly more polarizing than the soft Ba^2+^ cation, thus favouring more covalent Mg–C 2e bonds. In contrast, the large Ba^2+^ prefers interaction with a large and soft C_6_H_6_^2−^ dianion. The amount of ring puckering also influences Ae–C bond distances. The most extreme puckered benzene ring shows a wide range of Mg–C bond distances: 2.209(1)–2.883(3) Å, while Ba–C bond distances for the least puckered benzene ring are in a narrow range 2.918(3)–3.026(4) Å (Fig. 2a). Whereas aromatic benzene has a planar structure with delocalised C–C bonds of equal length, the bridging (η^6^:η^6^)-C_6_H_6_^2−^ dianions in all structures show unequal C–C bonds and varying degrees of ring puckering (Fig. 2a). Their geometries can be interpreted as a quinoid structure (two short and four longer C–C bonds) or a bis-allyl structure (four short and two longer C–C bonds). A comprehensive computational study on Li–(C_6_H_6_)–Li explains the origin of these distortions.^34^ In its most symmetric *D*_6h_ form, the C_6_H_6_^2−^ dianion is in a singlet state in which the two additional electrons reside mainly in the periphery on the H atoms occupying a Rydberg molecular orbital (MO) with anti-bonding C–H character. A more stable alternative is the triplet state with single occupation of the two degenerated e_2u_ orbitals (Fig. 2b), resulting in a C_6_H_6_^2−^ dianion with equal C–C bonds. However, the benzene dianion can be considerably stabilised by Jahn–Teller distortions. This either leads to a quinoid structure, resulting in lowering of the b_1u_ MO, or to a bis-allyl structure which gives energy gain due to lowering of the a_u_ MO. Additionally, the second-order Jahn–Teller effect leads to a loss of [C_6_H_6_]^2−^ planarity, which is gradually neutralised for heavier Ae^2+^ cations (Ca < Sr < Ba), which prefer ionic bonding with diffuse p-orbitals (Fig. 2a). In the crystal structures presented herein, the geometries of [C_6_H_6_]^2−^ dianions point to a singlet ground state with either quinoid or bis-allyl distortions. However, as mentioned (*vide supra*), a narrow singlet-triplet gap allows thermal population of the open-shell state causing paramagnetic behaviour in solution. The recently reported Eu complex, [(^DIPeP^BDI)Eu·THF]_2_(η^6^:η^6^-C_6_H_6_),^35^ showed the successful stabilisation of a long sought-after benzene dianion in the planar triplet state. This was enabled by antiferromagnetic coupling of two Eu^II^ nuclei through strong d–π* bonding interactions with the bridging [C_6_H_6_]^2−^ dianion.

### Computational studies

Molecular structures of 1-Ca, 1-Sr and 1-Ba were optimised in singlet closed-shell, singlet open-shell, and triplet states at the (U)B3PW91/def2-TZVP level of theory (see SI for details). To address the trends in the full series of Ae benzene inverse sandwich complexes, we included the structure of [(^DIPP^BDI)Mg]_2_(η^2^:η^4^-C_6_H_6_) which we abbreviate as 1′-Mg.^17^

**Fig. 3:**
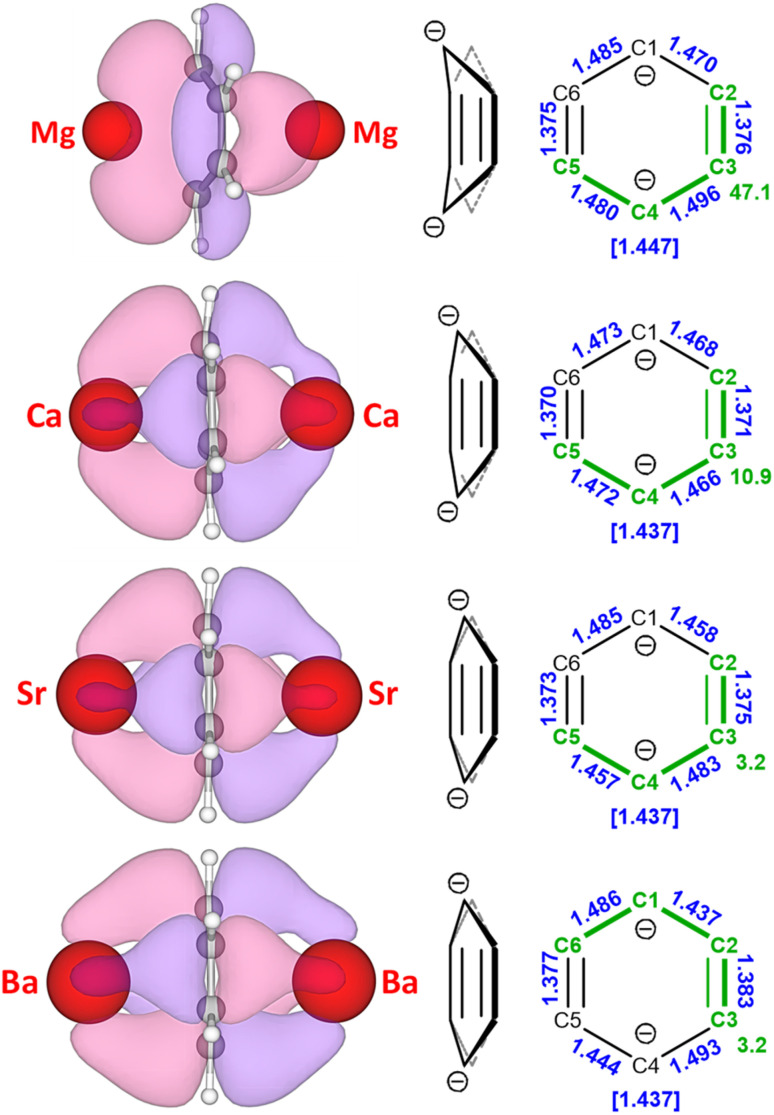
HOMO (isosurface 0.03 a.u.) representing Ae–(C_6_H_6_)–Ae bonding calculated for 1′-Mg, 1-Ca, 1-Sr and 1-Ba in the singlet state. Also shown is the geometry of the benzene dianion with C–C bond lengths (Å) marked in blue and the largest C–C–C–C torsion angles (°) marked in green.

Optimisation of all structures in the closed shell singlet state reproduced the crystal structures quite well (Tables S8 and S9). The dominant ionic character in all of these complexes is confirmed by relatively high positive NPA charges on the metals (1′-Mg: +1.69/+1.75, 1-Ca: +1.71/+1.74, 1-Sr: +1.74/+1.76, 1-Ba: +1.72/+1.75) and high negative charges on the bridging benzene dianions (−1.63/−1.56/−1.59/−1.58). The benzene dianions in the optimised structures adopt quinoidal structures, which is in agreement with crystal structures of 1′-Mg and 1-Ba, but contrasts with those of 1-Ca and 1-Sr, where bis-allyl forms are observed (Fig. 2a). This is in line with the very small energy difference between both isomers.^34^ The calculated structures generally present comparable ring puckering, when compared with experimental data. Calculated C–C–C–C torsion angles have values ranging from 47.1° (1′-Mg) to 3.2° (1-Sr and 1-Ba), while average C–C bond distances are in the 1.437–1.447 Å range (Fig. 3). NBO analysis shows that Mg–benzene bonding in 1′-Mg is comprised of Mg 3s-orbital and benzene π*-orbitals (b_1u_). The extreme boat form of the bridging benzene dianion results in asymmetric bonding. For the heavier Ae metals the ring flattens and not only valence s-orbitals but also empty d-orbitals participate in Ae–benzene bonding by accepting electron density (see Table S7). This is well presented by the calculated HOMO orbitals, which are formed by the overlap of the *n*s or *n*s/(*n* − 1)d orbitals of the Ae metal centres with the six 2p_*z*_ atomic orbitals of the benzene carbon atoms (Fig. 3).

Open-shell singlet/triplet calculations confirm that diradical character of the heavier 1-Ae complexes (Ca, Sr, Ba) is related to stable triplet states. These have been calculated to be 6.5 kcal mol^−1^ more stable for 1-Ca and are slightly more stable for 1-Sr and 1-Ba (1.3 kcal mol^−1^). In the triplet states, the benzene dianions show high *D*_6h_ symmetry and are nearly planar (the largest C–C–C–C torsion angles: 0.6° for 1-Ca, 0.7° 1-Sr and 2.0° for 1-Ba). The C–C bond lengths are almost equal with values ranging from 1.433–1.437 Å (Fig. S109). In all cases, the SOMO and SOMO−1 in the triplet states are equivalent to the LUMO and HOMO in the singlet states, respectively (Fig. S110–S112).

### Reactivity studies

**Scheme 3:**
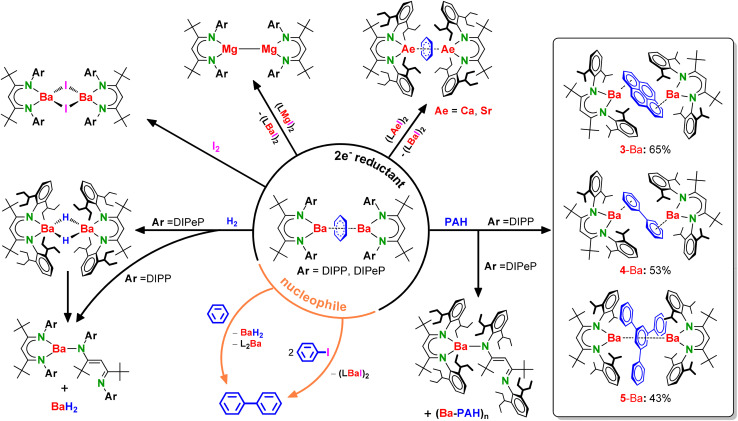
Reactivity of Ba benzene complexes depending on aryl (Ar = DIPP, DIPeP) substituents of ^Ar^BDI* ligand.

Preliminary studies on the reactivity of Ba benzene inverse sandwich complexes are summarised in Scheme 3.

### H_2_ activation

The methylcyclohexane solutions of 1-Ba and 2-Ba reacted instantaneously with 1.5 bar H_2_ at −120 °C. For 1-Ba the formation of the expected Ba hydride was confirmed by ^1^H NMR spectroscopy (Fig. S85). Diagnostic for a hydride ligand bound to Ba is the low-field singlet at 8.98 ppm, which is within the characteristic range of chemical shifts for Ba hydrides (7.92–10.39 ppm).^36–38^ However, although NMR spectroscopy confirmed hydride formation, the complex decomposed at room temperature over the course of two hours *via* ligand scrambling to homoleptic (^DIPeP^BDI*)_2_Ba and insoluble BaH_2_ (Fig. S86). Any attempt to stabilize and crystallize the heteroleptic Ba hydride complex at −35 °C failed. Also the addition of O-donors like THF or THP did not lead to product crystallisation. Complex 2-Ba reacted fast with H_2_ but due to the less bulky ^DIPP^BDI* ligand, only the well-characterised homoleptic complex (^DIPP^BDI*)_2_Ba was observed. Note, that the related Ba hydride complex [(^DIPP/TCHP^BDI)BaH]_2_ with the bulky ^DIPP/TCHP^BDI ligand (HC{MeCN(DIPP)}{MeCN(TCHP)}, TCHP = 2,4,6-tricyclohexylphenyl) could be isolated and characterised in the solid-state. This is likely due to its poor solubility and rapid crystallisation within 10 minutes from the reaction mixture.^39^ However, this complex decomposed immediately upon dissolving in THF-*d*_8_. Good solubility and slow crystallisation of our complexes resulted in product decomposition. It should be noted that bulkier ligands not always work in favour of complex stability.^40^ Both homoleptic complexes, (^DIPP^BDI*)_2_Ba and (^DIPeP^BDI*)_2_Ba, are spectroscopically characterised as asymmetric (κ^2^-BDI*)Ba(κ^1^-BDI*) complexes and resemble the previously reported (^DIPeP^BDI)_2_Ca complex featuring a bidentate and monodentate BDI ligand.^9^ Whereas BDI ligands normally prefer bidentate (BDI)–Ae coordination,^41^ using superbulky ligands requires loss of one of the Ae–N bonds to relieve steric strain. For a symmetric (^DIPP^BDI*)_2_Ba complex with two bidentate ligands, we calculated that cleavage of one of the Ba–N bonds is highly exergonic: Δ*G*_298 K_ = −37.6 kcal mol^−1^, Δ*H*^0^ = −52.8 kcal mol^−1^ (Table S5 and Fig. S113).

### Reduction with I_2_ or (BDI*)Ae iodides

Reaction of 1-Ba and 2-Ba with iodine led to almost selective formation of the (BDI*)BaI precursors that were used in their synthesis (Fig. S87 and S88). Both Ba benzene complexes also reacted with lighter Ae iodide precursors: [(^DIPeP^BDI*)AeI]_2_ (Ae = Mg, Ca, Sr). In case of Mg, benzene was eliminated and with moderate selectivity the formation of the Mg^I^ complexes [(^DIPP^BDI*)Mg]_2_ and [(^DIPeP^BDI*)Mg]_2_ was observed (Fig. S89 and S90). This shows that 1-Ba and 2-Ba can substitute K^0^ and act as soluble reducing agents for the reduction of Mg^II^ to Mg^I^. However, the anti-aromatic benzene dianion in these Ba^I^ synthons is not able to reduce Ca^II^ or Sr^II^. Reaction of 1-Ba with [(^DIPeP^BDI*)AeI]_2_ (Ae = Ca, Sr) in benzene or in cyclohexane did not result in metal reduction. Instead, we found an iodide/benzene ligand exchange, resulting in formation of 1-Ca and 1-Sr (Fig. S91 and S92). Interestingly, these ligand exchange processes are not reversible, *i.e.*1-Ca and 1-Sr do not react with [(^DIPeP^BDI*)BaI]_2_. Therefore, the driving force for these ligand exchange processes must lie in differences in Ae–benzene and Ae–I bond strengths. Apparently, the Ba^2+^ cation has a very high affinity for the iodide anion.

### Reduction of polyaromatic hydrocarbons

The reduction of selected polyaromatic hydrocarbons (PAHs) with 2-Ba is clean and selective as has been demonstrated for pyrene, biphenyl and 1,3,5-triphenyl-benzene (TPBz). In contrast, reaction of the bulkier complex 1-Ba led to formation of homoleptic (^DIPeP^BDI*)_2_Ba as the main product. A possible explanation must be related to the only difference between both complexes: ligand bulk. Apparently, the extreme bulk of the ^DIPeP^BDI* ligand stabilizes the benzene complex 1-Ba but is not favourable for stabilisation of complexes with larger PAH^2−^ dianions. We assume that after initial formation of a (^DIPeP^BDI*)Ba–(PAH)–Ba(^DIPeP^BDI*) complex, the product rapidly decomposes to (^DIPeP^BDI*)_2_Ba and Ba(PAH). Such a scenario was found for reactions of 1-Ba with all PAHs that were tested (pyrene, biphenyl and TPBz). Note that this procedure enabled the isolation of (^DIPeP^BDI*)_2_Ba, which could not be obtained selectively *via* a standard salt metathesis method (SI: Fig. S84, S93 and S94). Additional confirmation for decomposition of (^DIPeP^BDI*)Ba–(PAH)–Ba(^DIPeP^BDI*) species by the Schlenk equilibrium comes from the reaction of 1-Ba with 9,10-bis(trimethylsilyl)anthracene (TMS-Anth). Besides formation of (^DIPeP^BDI*)_2_Ba, the previously reported complex [Ba(TMS-Anth)]_*n*_ (ref. 24) was identified in the ^1^H NMR spectrum (Fig. S95).

The ^DIPP^BDI*-based Ba inverse sandwich compounds 3-, 4- and 5-Ba containing pyrene, biphenyl, and TPBz dianions were prepared from *in situ* generated 1-Ba (Scheme 2c) and isolated as crystals in yields of 65%, 53% and 43%, respectively. Although all were isolated in crystalline form, crystallographic characterisation was only successful for the pyrene complex 3-Ba (Fig. S107). All Ba–PAH compounds present well-resolved NMR spectra, facilitating the complete assignment of ^1^H and ^13^C NMR signals (Fig. S45–S62). Though not crystallographically characterised, the most interesting complex is 5-Ba, for which NMR analysis points to η^6^-coordination of Ba centres to the inner ring of triphenylbenzene, in a similar manner like for Westerhausen's Ca inverse sandwich.^42^ This stands in contrast with a previously published Mg complex in which the (1,3,5-Ph_3_C_6_H_3_)^2−^ dianion is sandwiched between two [(^DIPP^BDI)Mg]^+^ cations coordinating to one of its outer Ph rings (Fig. S78).^30^ This arrangement is likely dictated by steric congestion. Latter Mg compound was prone to decomposition by rearomatisation of triphenylbenzene and Mg^II^ → Mg^I^ reduction.

### [C_6_H_6_]^2−^ as nucleophile

The nucleophilic behaviour of the [C_6_H_6_]^2−^ dianion in 1-Ba and 2-Ba is confirmed by its reactivity with iodobenzene, forming free biphenyl and (BDI*)Ba iodide by nucleophilic aromatic substitution (Fig. S96). Dissolved in benzene, both Ba benzene complexes also mediate dehydrogenative benzene–benzene coupling, which reduces their lifetimes in benzene solution to 2–3 days. Attempting to remove benzene solvent by freeze-drying at −15 °C led to biphenyl formation. In contrast to previously reported reactivity with Ca or Sr benzene complexes,^18^ the product is detected as neutral free biphenyl accompanied with homoleptic (BDI*)_2_Ba and presumably BaH_2_ (Fig. S97 and S98).

## Conclusions

In order to isolate the most reactive and unstable Ae benzene inverse sandwich complex, (BDI)Ba–(C_6_H_6_)–Ba(BDI), we used a combination of ball-milling and solution chemistry. To stabilize such labile complexes, superbulky BDI ligands are crucial.

The availability of benzene inverse sandwich complexes throughout the series Mg, Ca, Sr and Ba allows for evaluation of metal effects on stability, structure, electronics and reactivity. Using the ^DIPeP^BDI ligand allowed for isolation benzene inverse sandwich complexes for the metals Mg, Ca and Sr which show increased lability with metal size. For the bigger ligand ^DIPeP^BDI* with *t*Bu-backbone substituents, Ca, Sr and Ba complexes were isolated. The crucial ligand architecture required for the challenging isolation of the Ba complexes are the *t*Bu-substituents in the ligand backbone. These push the Ar rings towards the large Ba metal cations, stabilizing the complexes with secondary Ba⋯(π-Ar) interactions. The latter results in an unexpected high stability for these Ba complexes which dissolved in alkanes decompose over a period of up to 15 days.

Another noticeable trend is found in the structure of the bridging benzene dianions. Whereas in the Mg–(C_6_H_6_)–Mg moiety the ring is strongly distorted from planarity and features a boat conformation, moving to heavier Ae metals result in flattening of this structure. The ring in the Ba–(C_6_H_6_)–Ba moiety is essentially flat. All crystal structures show rings with combinations of short and long C–C bonds as is typical for closed-shell singlet states. This can either be a quinoidal structure (two short C=C bonds and four longer C–C bonds) or a bis-allyl structure (two long C–C single bonds and four shorter allylic C–C bonds). Interestingly, in solution all benzene rings are NMR silent due to paramagnetic behaviour. This is due to low-lying triplet states in which the benzene ring is planar and the two unpaired electrons are in degenerate e_2u_ orbitals resulting in equal C–C bond distances. DFT calculations predict hardly any energy differences between these states. NBO analysis shows that all complexes have a large extent of ionic character with high positive charges on the metals (+1.69/+1.76) and high negative charges on the benzene ligands (−1.58/−1.63).

Preliminary reactivity studies of inverse sandwich complexes of type (BDI)Ba–(C_6_H_6_)–Ba(BDI) show fast reaction with H_2_ to give (BDI)BaH and benzene, showing that they are efficient Ba^I^ synthons. Unfortunately, while (^DIPeP^BDI*)BaH could be detected in solution, its high instability precluded further characterisation by crystal structure determination. Heteroleptic (BDI)BaH complexes decomposed in solution to homoleptic products (BDI)_2_Ba and BaH_2_. The complex (^DIPeP^BDI*)_2_Ba shows the typical NMR signals for an asymmetric complex with one bidentate and one mono-dentate BDI ligand. This is a demonstration of the bulk of the ^DIPeP^BDI* ligand. Even for the largest group 2 metal Ba, a complex with two bidentate ligands was calculated to be 37.6 kcal mol^−1^ (Δ*G*_298 K_) higher in energy than the bidendate/monodentate combination.

We could also demonstrate that these Ba^I^ synthons The Ba^I^ synthons are able to reduce a range of PAH's. However, more interesting is the Mg^II^ to Mg^I^ reduction. Reaction of (BDI)Ba–(C_6_H_6_)–Ba(BDI) with a (BDI)MgI precursor led to formation of (BDI)Mg–Mg(BDI), (BDI)BaI and benzene. However, heavier Ae metal cations like Ca^2+^ or Sr^2+^ could not be reduced. Reaction of (BDI)Ba–(C_6_H_6_)–Ba(BDI) reagents with a (BDI)AeI (Ae = Ca, Sr) precursor led to formation of the corresponding (BDI)BaI and (BDI)Ae–(C_6_H_6_)–Ae(BDI). This iodide/benzene^2−^ exchange does not work in the other direction, showing that the lighter benzene inverse sandwich complexes are more stable than the heavier ones.

Finally, the benzene^2−^ dianion in (BDI)Ba–(C_6_H_6_)–Ba(BDI) can also act as a nucleophile. It is well-known that the heavier Ae metals can facilitate nucleophilic aromatic substitution. This is for example key to H/D exchange at aromatic rings using Ae metal hydride catalysts.^43^ Thus, reaction of (BDI)Ba–(C_6_H_6_)–Ba(BDI) with PhI or simply benzene led to biphenyl formation.

We are currently exploring further reactivity of these first Ba^I^ synthons.

## Author contributions

D. Jędrzkiewicz: conceptualisation, investigation, validation, formal analysis, writing – original draft, visualisation. M. Morasch, O. P. E. Townrow, B. Rösch: investigation, validation, formal analysis. J. Langer: formal analysis, validation. Z. Mathe: formal analysis, validation. S. Harder: conceptualisation, writing – original draft – review and editing, validation, supervision, project administration.

## Conflicts of interest

There are no conflicts to declare.

## Supplementary Material

SC-OLF-D5SC05373K-s001

SC-OLF-D5SC05373K-s002

## Data Availability

CCDC 2435047–2435054 and 2435056 contain the supplementary crystallographic data for this paper.^44*a*–*i*^ Supplementary information: Synthetic procedures, selected NMR spectra, details for crystal structure determination, details for EPR characterization, details for computational work. See DOI: https://doi.org/10.1039/d5sc05373k.
