# Supplementary material for: Trends in benzene inverse sandwich complexes of the alkaline-earth metals Mg, Ca, Sr and Ba
Source: Chem Sci. 2025 Aug 29;16(38):17793–802. doi: 10.1039/d5sc05373k (PMC12409674; doi:10.1039/d5sc05373k)
Supplement: SC-016-D5SC05373K-s001 [file SC-016-D5SC05373K-s001.pdf]

Supplementary Information (SI) for Chemical Science.
This journal is © The Royal Society of Chemistry 2025

Supporting Information

Trends in benzene inverse sandwich complexes of the alkaline-earth metals Mg, Ca, Sr and Ba

Dawid Jędrzkiewicz*, Michael Morasch, Oliver P. E. Townrow, Lukas Klerner, Bastian Rösch, Jens Langer, Zachary Mathe, Sjoerd Harder*

Inorganic and Organometallic Chemistry, Universität Erlangen-Nürnberg, Egerlandstrasse 1, 91058 Erlangen, Germany. Fax: (+49) 9131-8527387

E-mail: sjoerd.harder@fau.de, dawid.jedrzkiewicz@gmail.com

Contents

1. Supporting Experimental Data S2

1.1 General Experimental Procedures S2

1.2 Syntheses S3

1.3 NMR Characterisation S11

1.4 NMR spectra of Ae benzene compounds S50

1.5 NMR Experiments S51

1.6 Crystal Structure Determination S61

1.7 EPR Measurements S73

1.8 Computational Details S74

2. References S94

1. Supporting Experimental Data

1.1 General Experimental Procedures

All experiments were conducted in dry glassware under an inert nitrogen or argon atmosphere by applying standard Schlenk techniques or gloveboxes (MBraun) using freshly dried and degassed solvents. Hexanes, *n*-pentane, cyclohexane, benzene, diethyl ether (Et_2O), tetrahydrofuran (THF) were degassed with nitrogen, dried over a column with activated aluminum oxide (Innovative Technology, Pure Solv 400-4-MD, Solvent Purification System) and then stored under inert atmosphere over molecular sieves (3 Å). Iodobenzene (Alpha Asear) was dried over freshly grounded CaH_2, distilled and stored over molecular sieves (3 Å) under inert atmosphere. Deuterated benzene (C_6D_6), deuterated cyclohexane (C_6D_{12}) and deuterated toluene (C_7D_8) were purchased from Deutero GmbH, degassed and dried over molecular sieves (3 Å) and stored under an inert atmosphere. Following reagents were obtained commercially and used without further purification: N_2 (Linde, 4N) and H_2 (Linde, 5N), potassium (chunks, washed with hexane, 98% metal basis, Sigma Aldrich), calcium (granules, redistilled, 99.5% metal basis), strontium (granules, redistilled, 99.5% metal basis), barium (Fischer Scientific, >99%), trimethylsilyl chloride (Sigma Aldrich, purified by redistillation, ≥99%), 9,10-dibromoanthracene (Sigma Aldrich, 98%), Benzene-$^{13}C_6$ (Sigma Aldrich, 99 atom % ^{13}C, 99%).

Iodine (Sigma Aldrich, 99.99%) was sublimed and stored under nitrogen atmosphere, 1,3,5-triphenylbenzene (Sigma Aldrich, 97%) was purified by recrystallisation from methanol, dried under reduces pressure and stored under N_2, biphenyl (Sigma Aldrich, >99%) and pyrene (Alfa Aesar, 98 %) were sublimed under reduced pressure and stored under N_2, 2,6-($CHEt_2$)-aniline (DIPeP aniline) was synthesised according to a slightly modified literature procedure.[S1] The following compounds were prepared according to literature procedures: CaI_2, SrI_2, BaI_2,[S2] 5% w.w K/KI,[S3] KC_8[S4], benzyl potassium (KBn),[S5] (^{DIPP}BDI*)-H,[S6] (^{DIPeP}BDI*)-H,[S7] (^{DIPeP}BDI [(^{DIPP}BDI*)Ba(μ-I)]$_2$,[S8] 9,10-bis(trimethylsilyl)anthracene.[S9]

The ball-mill used was a ULTRA-TURRAX® Tube Drive P control from IKA. Mechanochemical reactions were performed in 20 mL polypropylene vessel with three stainless steel balls (diameter: 5 mm, weight: 0.52 g, type: AISI 304).

NMR spectra were measured on Bruker Avance III H 400 MHz and Bruker Avance III HD 600 MHz NMR spectrometers. Chemical shifts (δ) are denoted in ppm (parts per million) and coupling constants in Hz (Hertz). ^1H and ^{13}C NMR spectra were referenced to the solvent residual signal ($SiMe_4$ = 0 ppm). Signal multiplicities are described using common abbreviations: s (singlet), d (doublet), t (triplet), q (quartet), p (quintet), h (heptet), m (multiplet) and br (broad). Elemental analysis was performed with a Hekatech Eurovector EA3000 analyzer. All crystal structures have been measured on a SuperNova (Agilent) diffractometer with dual Cu and Mo microfocus sources and an Atlas S2 detector.

Perpendicular-mode X-band electron paramagnetic resonance (EPR) spectroscopy was performed with a Magnettech MS5000 spectrometer equipped with a LN2 cryostat.

1.2 Syntheses

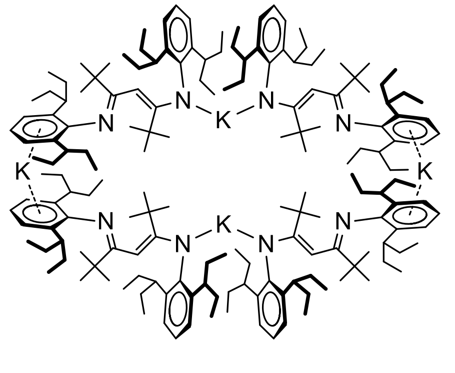

[(^{DIPeP}BDI*)K]$_4$ (^{DIPeP}BDI*)-H (2.46 g, 4.0 mmol) and KBn (521 mg, 4.0 mmol) were dissolved in THF (50 mL) and stirred for 8h at room temperature. The solvent was removed *in vacuo* and the residue was stripped with hexanes (20 mL). The resulting suspension was dried under high vacuum to give a pale-yellow powder as the product in a yield of 96.3% (2.51 g, 0.963 mmol). Yellow block-like crystals suitable for X-ray diffraction analysis were obtained from saturated benzene solution layered with hexanes.

^{1}H NMR (600.13 MHz, C_6D_6, 298K): δ = 7.04 (br d, q, ^3J = 7.7 Hz, 8H, Ar**H**), 6.94 (br t, q, ^3J = 7.5 Hz, 4H, Ar**H**), 6.78 (br d, q, ^3J = 7.7 Hz, 8H, Ar**H**), 6.47 (br t, q, ^3J = 7.5 Hz, 4H, Ar**H**), 4.22 (br s, 4H, C**H**^{backbone}), 2.98 – 2.90 (m, 8H, C**H**$^{3\text{-}Pent}$), 2.90 – 2.83 (m, 8H, C**H**$^{3\text{-}Pent}$), 1.93 – 1.78 (m, 24H, C**H$_2$**$^{3\text{-}Pent}$), 1.70 (s, 36H, C**H$_3$**$^{t\text{-}Bu}$), 1.64 – 1.51 (m, 16H, C**H$_2$**$^{3\text{-}Pent}$), 1.50 – 1.33 (m, 24H, C**H$_2$**$^{3\text{-}Pent}$), 1.15 – 1.10 (m, 24H, C**H$_3$**$^{3\text{-}Pent}$), 1.09 (s, 36H, C**H$_3$**$^{t\text{-}Bu}$), 1.03 (br d, J = 7.7 Hz, 48H, C**H$_3$**$^{3\text{-}Pent}$), 0.90 – 0.69 (m, 24H, C**H$_3$**$^{3\text{-}Pent}$).

^{13}C NMR (150.92 MHz, C_6D_6, 298K): δ = 173.0 (**C**$^{t\text{-}Bu}$), 162.7 (**C**$^{t\text{-}Bu}$), 154.2 (Ar**C**-C), 151.5 (Ar**C**-C), 136.8 (Ar**C**-C), 134.9 (Ar**C**-C), 125.7 (Ar**C**H), 124.5 (Ar**C**H), 121.2 (Ar**C**H), 115.4 (Ar**C**H), 88.6 (**C**H^{backbone}), 45.0 (**C**(CH_3)$_3$), 40.9 (**C**H$^{i\text{-}Pr}$), 39.4 (**C**(CH_3)$_3$), 32.9, C(**C**H$_3$)$_3$), 32.3 (**C**H$_2^{3\text{-}Pent}$), 29.6 (C(**C**H$_3$)$_3$), 26.5 (**C**H$_2^{3\text{-}Pent}$), 25.7 (**C**H$_2^{3\text{-}Pent}$), 13.0 (**C**H$_3^{3\text{-}Pent}$), 12.4 (**C**H$_2^{3\text{-}Pent}$), 12.0 (**C**H$_2^{3\text{-}Pent}$), 11.9 (**C**H$_3^{i\text{-}Pr}$) ppm.

Elemental analysis calculated for $C_{172}H_{276}N_8K_4$ (M = 2612.56 g/mol): C 79.08; H 10.65; N 4.29 %. Found: C 79.51; H 10.93; N 4.01 %.

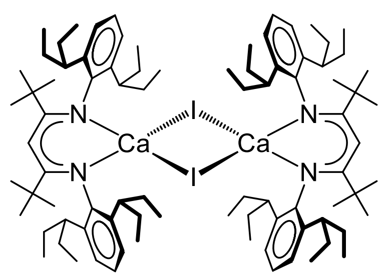

[(^{DIPeP}BDI*)Ca(μ-I)]$_2$ [(^{DIPeP}BDI*)K]$_4$ (1.31 g, 0.5 mmol) was dissolved in Et_2O (50 mL), then CaI_2 (588 mg, 2.0 mmol) was added and the resulting suspension was stirred overnight. Residue was filtered off and extracted with Et_2O (2 x 20 mL). The filtrate was combined with extracts, solvent was removed and the resulting yellow solid was washed with cold *n*-pentane (2 x 5 mL) and dried *in vacuo* to give a pale-yellow powder as the product. Yield: 92.2% (1.44 g, 0.92 mmol). Colorless block-like crystals suitable for X-ray diffraction analysis were obtained from a saturated benzene solution.

^{1}H NMR (600.13 MHz, C_6D_6, 323K): δ = 7.02 (br s, 12H, Ar**H**), 5.40 (s, 2H, C**H**^{backbone}), 3.04 (br s, 8H, C**H**$^{3\text{-}Pent}$), 1.89 – 1.80 (m, 16H, C**H$_2$**$^{3\text{-}Pent}$), 1.79 – 1.71 (m, 8H, C**H$_2$**$^{3\text{-}Pent}$), 1.68 – 1.57 (m, 8H, C**H$_2$**$^{3\text{-}Pent}$), 1.23 (s, 36H, C**H$_3$**$^{t\text{-}Bu}$), 1.02 (t, ^3J = 7.4 Hz, 24H, C**H$_3$**$^{3\text{-}Pent}$), 0.89 (br s, 24H, C**H$_3$**$^{3\text{-}Pent}$).

^{13}C NMR – not measured due to low solubility and fast crystallisation in C_6D_6 and toluene-d_8.

Elemental analysis for $C_{86}H_{138}N_4Ca_2I_2$ (M = 1562.04 g/mol): Calculated for one co-crystallised benzene molecule (M = 1640.16 g/mol): C 67.37; H 8.85; N 3.42 %. Found: C 67.05; H 8.85; N 3.59 %.

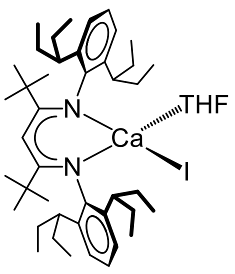

[(^{DIPeP}BDI*)CaI · THF] (^{DIPeP}BDI*)-H (1.23 g, 2.0 mmol) and KBn (260 mg, 2.0 mmol) were dissolved in THF (30 mL) and stirred for 8h at room temperature until the red color of the solution disappeared. Then CaI_2 (588 mg, 2.0 mmol) was added and the resulting suspension was stirred overnight. The solvent was removed *in vacuo* and the residue extracted with benzene (3 x 20 mL). The filtrate was combined with extracts and the volume was reduced to 5 mL, and then 5 mL of *n*-pentane was added. Leaving it standing at room temperature overnight gave yellow block-like crystals suitable for X-ray diffraction analysis that were filtered off, washed with cold *n*-pentane and dried *in vacuo* (yield: 0.95 g, 1.11 mmol). The filtrate was dried *in vacuo*, dissolved in 5 mL of pentane and kept at -40 °C to give a second crop of crystals (0.56 g, 0.66 mmol). Combined yield: 1.51 g, 1.77 mmol, 88.7%).

^{1}H NMR (600.13 MHz, C_6D_6, 298K): δ = 6.95 (br s, 6H, Ar**H**), 5.60 (s, 1H, C**H**^{backbone}), 3.41 (br s, 4H, α-C**H₂**^{THF}), 3.10 (p, ^3J = 5.2 Hz, 4H, C**H**$^{3\text{-Pent}}$), 1.82 (dp, ^3J = 15.2, 7.5 Hz, 6H, C**H₂**$^{3\text{-Pent}}$), 1.73 (dp, ^3J = 14.1, 7.4 Hz, 6H, C**H₂**$^{3\text{-Pent}}$), 1.49 – 1.39 (m, 4H, C**H₂**$^{3\text{-Pent}}$), 1.26 (s, 18H, C**H₃**$^{t\text{-Bu}}$), 1.17 (br s, 4H, β-C**H₂**^{THF}), 1.04 (t, ^3J = 7.4 Hz, 12H, C**H₃**$^{3\text{-Pent}}$), 0.79 (t, ^3J = 7.4 Hz, 12H, C**H₃**$^{3\text{-Pent}}$) ppm.

^{13}C NMR (150.92 MHz, C_6D_6, 298K): δ = 175.3 ((*t*-Bu)**C**), 149.0 (Ar**C**-N), 135.9 (Ar**C**-C), 125.6 (ArCH), 121.5 (ArCH), 90.2 (**C**H^{backbone}), 70.3 (α-**C**H_2^{THF}), 44.6 (**C**$(CH_3)_3$), 40.1 (**C**H$^{3\text{-Pent}}$), 32.4 (C(**C**$H_3)_3$), 26.5 (**C**$H_2^{3\text{-Pent}}$), 24.9 (**C**$H_2^{3\text{-Pent}}$), 24.5 (β-**C**H_2^{THF}), 12.1 (**C**$H_3^{3\text{-Pent}}$), 8.9 (**C**$H_3^{3\text{-Pent}}$) ppm.

Elemental analysis calculated for $C_{47}H_{78}N_2OCaI$ (M = 853.13 g/mol) Calculated for one co-crystallised pentane molecule (M = 925.28 g/mol): C 67.50; H 9.70; N 3.03 %. Found: C 66.52; H 10.08; N 2.70 %.

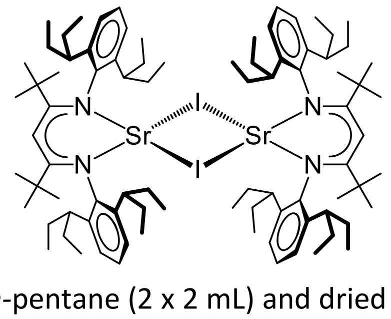

[(^{DIPeP}BDI*)Sr(*μ*-I)]₂ [(^{DIPeP}BDI*)K]₄ (1.00 g, 0.38 mmol) and SrI_2 (532 mg, 1.56 mmol) were suspended in Et_2O (15 mL) and stirred overnight. After removing the solvent *in vacuo*, the residue was extracted with boiling benzene (10 mL) and the hot solution was slowly cooled to room-temperature before placing it at 8 °C. After three days, small block-like crystals suitable for X-ray diffraction analysis formed, which were isolated by decantation of the supernatant, washed with cold *n*-pentane (2 x 2 mL) and dried *in vacuo* to obtain the product as a fine yellow crystalline solid (583 mg, 0.352 mmol, 46 %).

^{1}H NMR (600.13 MHz, THF-*d₆*, 298K): δ = 6.65 (d, ^3J = 7.4 Hz, 8H, Ar**H**), 6.36 (t, ^3J = 7.4 Hz, 4H, Ar**H**), 3.77 (s, 2H, C**H**^{backbone}), 2.93 (p, ^3J = 6.8 Hz, 8H, C**H**$^{3\text{-Pent}}$), 1.67 – 1.56 (m, 16H, C**H₂**$^{3\text{-Pent}}$), 1.56 – 1.47 (m, 16H, C**H₂**$^{3\text{-Pent}}$), 1.15 (br s, 36H, C**H₃**$^{t\text{-Bu}}$), 0.84 (t, ^3J = 7.4 Hz, 24H, C**H₃**$^{3\text{-Pent}}$), 0.82 (t, ^3J = 7.4 Hz, 24H, C**H₃**$^{3\text{-Pent}}$) ppm.

^{13}C NMR (150.92 MHz, THF-*d₆*, 298K): δ = 156.0 ((*t*-Bu)**C**), 135.2 (Ar**C**-N), 129.2 (Ar**C**-C), 123.3 (ArCH), 115.8 (Ar**C**H), 87.4 (**C**H^{backbone}), 42.4 (**C**$(CH_3)_3$), 41.5 (**C**H$^{3\text{-Pent}}$), 32.3 (C(**C**$H_3)_3$), 28.5 (**C**$H_2^{3\text{-Pent}}$), 27.9 (**C**$H_2^{3\text{-Pent}}$), 13.0 (**C**$H_3^{3\text{-Pent}}$), 12.6 (**C**$H_3^{3\text{-Pent}}$) ppm.

Elemental analysis for $C_{86}H_{138}N_4Sr_2I_2$ (M = 1657.09 g/mol): C 62.33; H 8.39; N 3.38 %. Found: C 62.17; H 8.34; N 3.27 %.

[(^{DIPeP}BDI*)Ba(*μ*-I)]$_2$ [(^{DIPeP}BDI*)K]$_4$ (1.31 g, 0.5 mmol) was dissolved in the mixture of THF/Et_2O (5mL / 55 mL), then BaI_2 (782 mg, 2.0 mmol) was added and the resulting suspension was stirred overnight. The residue was filtered off and extracted with Et_2O (2 x 60 mL). The filtrate was combined with extracts, solvent was removed and the remaining yellow solid was stripped with cold *n*-pentane (20 mL) and dried *in vacuo* at 60 °C for 4 h to give the product as yellow powder. Yield: 87.2% (1.53 g, 0.87 mmol). Yellow block-like crystals suitable for X-ray diffraction analysis were obtained from saturated THF solution layered on top of a mixture of benzene and hexanes (1mL / 5 mL / 5mL).

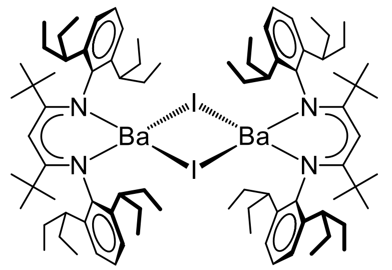

^{1}H NMR (600.13 MHz, C_6D_6, 323K): δ = 7.21 (d, ^3J = 7.7 Hz, 8H, Ar**H**), 7.12 (t, ^3J = 7.7 Hz, 4H, Ar**H**), 5.30 (s, 2H, C**H**^{backbone}), 2.66 – 2.58 (m, 8H, C**H**$^{3\text{-}Pent}$), 1.93 (hept, ^3J = 7.4 Hz, 8H, C**H$_2$**$^{3\text{-}Pent}$), 1.73 (hept, ^3J = 7.4 Hz, 8H, C**H$_2$**$^{3\text{-}Pent}$), 1.69 – 1.61 (m, 8H, C**H$_2$**$^{3\text{-}Pent}$), 1.58 – 1.50 (m, 8H, C**H$_2$**$^{3\text{-}Pent}$), 1.30 (s, 36H, C**H$_3$**$^{t\text{-}Bu}$), 1.15 (t, ^3J = 7.2 Hz, 24H, C**H$_3$**$^{3\text{-}Pent}$), 0.96 (t, ^3J = 7.4 Hz, 24H, C**H$_3$**$^{3\text{-}Pent}$) ppm.

^{13}C NMR (150.92 MHz, C_6D_6, 298K): δ = 170.7 ((*t*-Bu)**C**), 147.2 (Ar**C**-N), 134.7 (Ar**C**-C), 128.0 (ArCH), 122.5 (Ar**C**H), 97.3 (**C**H^{backbone}), 44.1 (**C**(CH_3)$_3$), 41.0 (**C**H$^{3\text{-}Pent}$), 32.5 (C(**C**H$_3$)$_3$), 27.6 (**C**H$_2$$^{3\text{-}Pent}$), 23.0 (**C**H$_2$$^{3\text{-}Pent}$), 13.1 (**C**H$_3$$^{3\text{-}Pent}$), 10.4 (**C**H$_3$$^{3\text{-}Pent}$) ppm.

Elemental analysis for $C_{86}H_{138}N_4Ba_2I_2$ (M = 1756.54 g/mol): C 58.81; H 7.92; N 3.19 %. Found: C 59.33; H 7.81; N 3.11 %.

[{(^{DIPeP}BDI*)Ca}$_2$(*η*^{6}:*η*^{6}-C_6H_6)] [(^{DIPeP}BDI*)Ca(*μ*-I)]$_2$ (391 mg, 0.25 mmol) was placed in 100 mL Schenk flask and dissolved in benzene (50 mL). Then K/KI (5% w/w, 2.34 g, 3.0 mmol, 6 eq. K) was added and the suspension was stirred overnight at room temperature. The solution was filtered off and evaporated to dryness. The resulting dark crude solid was recrystallised from *n*-pentane (5 mL) giving a first crop of black crystals suitable for X-ray measurement at -40 °C in 2 days. Crystals were isolated by decantation, washed with cold *n*-pentane (-40 °C, 1x 0.5 mL) and dried in *vacuo* (yield 171 mg, 123 µmol). The remaining filtrate was concentrated to 1 mL and kept at -40 °C to give a second crop of black crystals (25 mg, 18 µmol). Overall yield: 56.4% (196 mg, 141 µmol).

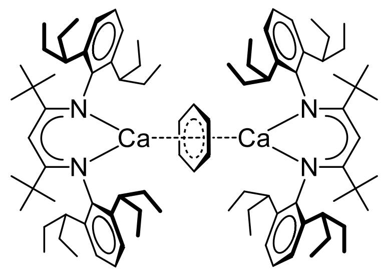

^{1}H NMR (600.13 MHz, C_6D_{12}, 298K): δ = 6.28 (br s, 4H, Ar**H**), 6.12 (br s, 8H, Ar**H**), 5.87 (s, 2H, C**H**^{backbone}), 3.50 (br s, 8H, C**H**$^{3\text{-}Pent}$), 1.98 (br s, 8H, C**H$_2$**$^{3\text{-}Pent}$), 1.72 (br s, 16H, C**H$_2$**$^{3\text{-}Pent}$), 1.52 (br s, 36H, C**H$_3$**$^{t\text{-}Bu}$), 1.93 (br s, 24H, C**H$_3$**$^{3\text{-}Pent}$), 0.75 (br s, 8H, C**H$_2$**$^{3\text{-}Pent}$), 0.19 (br s, 24H, C**H$_3$**$^{3\text{-}Pent}$) ppm.

^{13}C NMR (150.92 MHz, C_6D_{12}, 298K): δ = 173.8 ((*t*-Bu)**C**), 149.8 (Ar**C**-N), 140.9 (Ar**C**-C), 126.5 (ArCH), 121.6 (Ar**C**H), 97.2 (**C**H^{backbone}), 44.9 (**C**(CH_3)$_3$), 42.5 (**C**H$^{3\text{-}Pent}$), 35.1 (C(**C**H$_3$)$_3$), 32.9 (**C**H$_2$$^{3\text{-}Pent}$), 24.4 (**C**H$_2$$^{3\text{-}Pent}$), 11.3 (**C**H$_3$$^{3\text{-}Pent}$), 10.4 (**C**H$_3$$^{3\text{-}Pent}$) ppm.

$C_6H_6^{2-}$ anion is NMR silent.

Elemental analysis for $C_{92}H_{144}N_4Ca_2$ (M = 1386.35 g/mol): Calculated: C 79.71; H 10.47; N 4.04 %. Found: C 79.84; H 10.72; N 4.02 %.

[{(^{DIPeP}BDI*)Sr}$_2$(η^6:η^6-C_6H_6)] [(^{DIPeP}BDI*)Sr(μ-I)]$_2$ (200 mg, 0.121 mmol) and KC_8 (97.6 mg, 0.723 mmol) were suspended in benzene (5 mL) and stirred overnight. Subsequently the mixture was filtrated, the solvent removed *in vacuo* and the residue was stripped with *n*-pentane (3 x 5 mL). The black solid residue was then dissolved in *n*-pentane (600 µL) and kept at -45 °C. After 1 week, crystals suitable for X-ray diffraction analysis were obtained and isolated by decantation of the supernatant. After drying *in vacuo* (BDI*)Sr-benzene was obtained as a black crystalline solid (75.0 mg, 0.051 mmol, 42%).

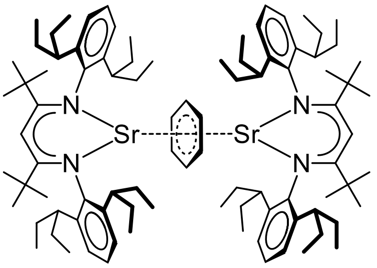

^{1}H NMR (600.13 MHz, C_6D_{12}, 298K): δ = 6.23 (s, 2H, C**H**^{backbone}), 5.98 (br s, 8H, Ar**H**), 5.21 (br s, 2H, Ar**H**), 4.09 (br s, 8H, C**H**$^{3\text{-}Pent}$), 2.07 (br s, 8H, C**H$_2$**$^{3\text{-}Pent}$), 2.01 (br s, 8H, C**H$_2$**$^{3\text{-}Pent}$), 1.77 (br s, 36H, C**H$_3$**$^{t\text{-}Bu}$), 1.04 (t, ^3J = 6.5 Hz, 24H, C**H$_3$**$^{3\text{-}Pent}$), 0.45 (br s, 8H, C**H$_2$**$^{3\text{-}Pent}$), -0.17 (br s, 24H, C**H$_3$**$^{3\text{-}Pent}$), -1.84 (br s, 8H, C**H$_2$**$^{3\text{-}Pent}$), ppm.

^{13}C NMR (150.92 MHz, C_6D_{12}, 298K): δ = 174.3 ((*t*-Bu)**C**), 155.0 (Ar**C**-N), 145.6 (Ar**C**-C), 132.3 (Ar**C**H), 118.5 (Ar**C**H), 95.7 (**C**H^{backbone}), 47.9 (**C**H$^{3\text{-}Pent}$), 44.6 (**C**(CH_3)$_3$), 34.9 (C(**C**H$_3$)$_3$), 26.3 (overlapped, **C**H$_2$$^{3\text{-}Pent}$), 13.9 (**C**H$_3$$^{3\text{-}Pent}$), 13.3 (**C**H$_3$$^{3\text{-}Pent}$) ppm. **C**H$_2$$^{3\text{-}Pent}$ signal was not identified due to the lack of correlation peaks on HSQC/HMBC spectra and intensive carbon signals from impurities.

$C_6H_6^{2-}$ anion is NMR silent.

Elemental analysis for $C_{92}H_{144}N_4Sr_2$ (M = 1481.28 g/mol): Calculated for 0.5 co-crystallised *n*-pentane molecule: C 74.80; H 9.96; N 3.69 %. Found: C 75.26; H 10.03; N 3.71 %.

[{(^{DIPeP}BDI*)Ba}$_2$(η^6:η^6-C_6H_6)] [(^{DIPeP}BDI*)Ba(μ-I)]$_2$ (110 mg, 63 µmol) and K/KI (5% w/w, 195 mg, 250 µmol) were placed in a vessel equipped with 4 stainless steel balls and ground over 30 minutes with ball-mill speed 1800 rpm (30 Hz). To the obtained dark green powder, 200 µL (226 µmol) of benzene was added and after 5 minutes the resulting dark brown slurry was extracted with 5 mL of *n*-pentane. The dark extract was kept at -35 °C. After few weeks, black crystals suitable for X-ray measurement appeared and were isolated by decantation and dried by evaporation at atmospheric pressure. Caution: applying high-vacuum results in product decomposition. Yield: 34.4% (34 mg, 21 µmol).

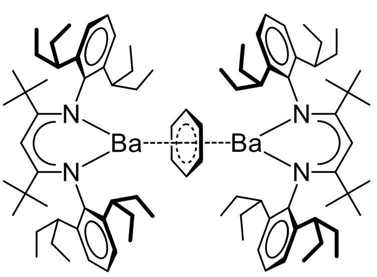

^{1}H NMR (600.13 MHz, C_6D_{12}, 298K): δ = 6.36 (s, 2H, C**H**^{backbone}), 5.81 (t, ^3J = 7.5 Hz, 4H, Ar**H**), 5.03 (d, ^3J = 7.9 Hz, 8H, Ar**H**), 4.40 (br s, 8H, C**H**$^{3\text{-}Pent}$), 2.18 (br s, 16H, C**H$_2$**$^{3\text{-}Pent}$), 1.89 (s, 36H, C**H$_3$**$^{t\text{-}Bu}$), 1.09 (t, ^3J = 7.5 Hz, 24H, C**H$_3$**$^{3\text{-}Pent}$), -0.03 (br s, 24H, C**H$_3$**$^{3\text{-}Pent}$), -0.06 (br s, 8H, C**H$_2$**$^{3\text{-}Pent}$), -2.04 (br s, 8H, C**H$_2$**$^{3\text{-}Pent}$) ppm.

^{13}C NMR (150.92 MHz, C_6D_{12}, 298K): δ = 173.0 ((*t*-Bu)**C**), 158.3 (Ar**C**-N), 147.5 (Ar**C**-C), 136.6 (Ar**C**H), 118.2 (Ar**C**H), 108.6 (**C**H$_2$$^{3\text{-}Pent}$), 95.3 (**C**H^{backbone}), 46.8 (**C**H$^{3\text{-}Pent}$), 44.2 (**C**(CH_3)$_3$), 35.1 (C(**C**H$_3$)$_3$), 29.3 (**C**H$_3$$^{3\text{-}Pent}$), 26.7 (**C**H$_2$$^{3\text{-}Pent}$), 14.1 (**C**H$_3$$^{3\text{-}Pent}$) ppm.

$C_6H_6^{2-}$ anion is NMR silent.

Elemental analysis for $C_{92}H_{144}N_4Ba_2$ (M = 1580.85 g/mol): Calculated: C 69.90; H 9.18; N 3.54 %. Found: C 69.67; H 9.01; N 3.49 %.

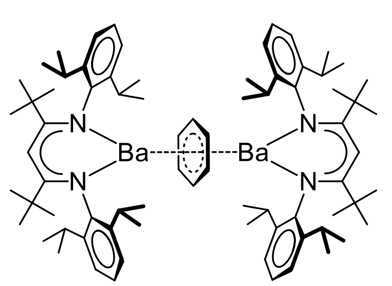

[{(^{DIPP}BDI*)Ba}$_2$(η^6:η^6-C_6H_6)] [(^{DIPP}BDI*)Ba(μ-I)]$_2$ (100 mg, 62 µmol) and K/KI (5% w/w, 195 mg, 250 µmol) were placed in a vessel equipped with 4 stainless steel balls and ground over 30 minutes with ball-mill speed 1800 rpm (30 Hz). To the obtained dark green powder, 200 µL (226 µmol) of benzene was added and after 5 minutes the resulting dark brown slurry was extracted with 5 mL of cyclohexane. The dark brown extract was mixed with *n*-pentane (15 mL) and kept at -35 °C. Multiple fused black needle-like crystals (not suitable for X-ray measurement) formed overnight and were isolated by decantation, washed with cold *n*-pentane (-35 °C, 4x 0.5 mL), and dried by evaporation at atmospheric pressure. Caution: applying high-vacuum results in product decomposition. Yield: 53.6% (45 mg, 33 µmol).

^{1}H NMR (600.13 MHz, C_6D_{12}, 298K): δ = 6.33 (s, 2H, C**H**^{backbone}), 5.77 (t, ^3J = 7.6 Hz, 4H, Ar**H**), 5.05 (d, ^3J = 7.7 Hz, 8H, Ar**H**), 4.68 (br s, 8H, C**H**$^{i\text{-}Pr}$), 1.89 (s, 36H, C**H$_3$**$^{t\text{-}Bu}$), 1.79 (d, ^3J = 6.8 Hz, 24H, C**H$_3$**$^{i\text{-}Pr}$), -0.50 (d, ^3J = 6.4 Hz, 24H, C**H$_3$**$^{i\text{-}Pr}$) ppm.

^{13}C NMR (150.92 MHz, C_6D_{12}, 298K): δ = 172.2 ((*t*-Bu)**C**), 156.3 (Ar**C**-N), 148.7 (Ar**C**H), 134.8 (Ar**C**-C), 121.4 (**C**H$^{i\text{-}Pr}$), 119.3 (Ar**C**H), 95.6 (**C**H^{backbone}), 44.3 (**C**(CH_3)$_3$), 34.8 (C(**C**H_3)$_3$), 32.3 (**C**H$^{i\text{-}Pr}$), 25.0 (**C**$H_3$$^{i\text{-}Pr}$) ppm.

$C_6H_6^{2-}$ anion is NMR silent.

Elemental analysis for $C_{76}H_{112}N_4Ba_2$ (M = 1356.41 g/mol): Calculated: C 67.30; H 8.32; N 4.13 %. Found: C 67.79; H 8.57; N 4.40 %.

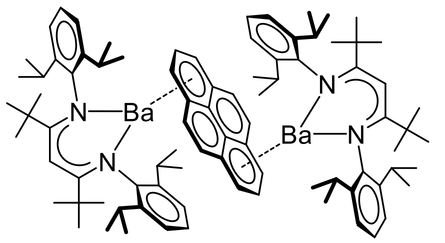

[{(^{DIPP}BDI*)Ba}$_2$(η^6:η^6-$C_{16}H_{10}$)] [(^{DIPP}BDI*)Ba(μ-I)]$_2$ (100 mg, 62 µmol) and K/KI (5% w/w, 195 mg, 250 µmol) were placed in a vessel equipped with 4 stainless steel balls and ground over 30 minutes with ball-mill speed 1800 rpm (30 Hz). To the obtained dark green powder, 200 µL (226 µmol) of benzene was added and after 5 minutes the resulting dark brown slurry was extracted with 5 mL of cyclohexane. To the dark brown extract, pyrene (6 mg, 30 µmol) was added and the resulting solution changed color to dark deep-blue. Volatiles were removed by evaporation, the residue was washed with benzene (4x 0.5 mL) and dried *in vacuo*. Yield: 65.0% (59 mg, 40 µmol). Crystals suitable for X-ray measurement were obtained from a saturated cyclohexane solution layered on top of benzene (0.5 mL / 2 mL) after 4 days at room temperature.

^{1}H NMR (600.13 MHz, C_6D_{12}, 298K): δ = 7.66 (t, ^3J = 7.8 Hz, 4H, Ar**H**), 7.58 (d, ^3J = 7.9 Hz, 8H, Ar**H**), 5.57 (s, 2H, C**H**^{backbone}), 3.88 (p, ^3J = 6.8 Hz, 8H, C**H**$^{i\text{-}Pr}$), 1.55 (s, 36H, C**H$_3$**$^{t\text{-}Bu}$), 1.50 (d, ^3J = 7.0 Hz, 24H, C**H$_3$**$^{i\text{-}Pr}$), 1.39 (d, overlapped, 24H, C**H$_3$**$^{i\text{-}Pr}$), 1.31 (t, ^3J = 7.8 Hz, 4H, Ar**H**^{pyrene}), 0.70 (s, 4H, Ar**H**^{pyrene}), -0.48 (d, ^3J = 7.7 Hz, 4H, Ar**H**^{pyrene}) ppm.

^{13}C NMR (150.92 MHz, C_6D_{12}, 298K): δ = 168.9 (**C**$^{t\text{-}Bu}$), 158.8 (Ar**C**-C^{pyrene}), 148.6 (Ar**C**-N), 147.3 (Ar**C**-C^{pyrene}), 143.5 (Ar**C**H^{pyrene}), 139.8 (Ar**C**-C), 127.0 (Ar**C**H), 125.7 (Ar**C**H), 116.0 (Ar**C**H^{pyrene}

101.1 (Ar**C**H^{pyrene}), 95.4 (**C**H^{backbone}), 44.6 (**C**(CH_3)$_3$), 33.4 (C(**C**H_3)$_3$), 28.9 (**C**H$^{i\text{-Pr}}$), 25.9 (**C**H$_3^{i\text{-Pr}}$), 24.5 (**C**H$_3^{i\text{-Pr}}$) ppm.

Elemental analysis for $C_{86}H_{116}N_4Ba_2$ (M = 1480.56 g/mol): Calculated for three co-crystallised benzene molecules): C 72.84; H 7.88; N 3.27 %. Found: C .72.89; H 7.98; N 3.27 %.

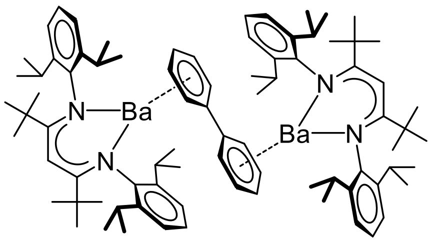

[{(^{DIPP}BDI*)Ba}$_2$(η^6:η^6-$C_{12}H_{10}$)] [(^{DIPP}BDI*)Ba(μ-I)]$_2$ (100 mg, 62 µmol) and K/KI (5% w/w, 195 mg, 250 µmol) were placed in a vessel equipped with 4 stainless steel balls and ground over 30 minutes with ball-mill speed 1800 rpm (30 Hz). To the obtained dark green powder, 200 µL (226 µmol) of benzene was added and after 5 minutes the resulting dark brown slurry was extracted with 5 mL of cyclohexane. To the dark brown extract, biphenyl (5 mg, 31 µmol) was added and the resulting solution changed color to dark deep-red. Sample was dried *in vacuo* and the residue was dissolved with 2 mL of *n*-pentane and placed at -35 °C. After two weeks a microcrystalline black solid formed which was isolated by decantation, washed with cold *n*-pentane (-35 °C, 2x 0.2 mL) and dried *in vacuo*. Yield: 53.1% (47 mg, 33 µmol).

^{1}H NMR (600.13 MHz, C_6D_6, 298K): δ = 7.01 (d, ^3J = 7.5 Hz, 8H, Ar**H**), 6.89 (t, ^3J = 7.6 Hz, 4H, Ar**H**), 5.30 (s, 2H, C**H**^{backbone}), 4.90 (dd, ^3J = 8.9, 6.2 Hz, 4H, ArH^{biphenyl}**),** 3.57 (dd, ^3J = 9.2, 1.4 Hz, 4H, Ar**H**^{biphenyl}**),** 3.43 (tt, ^3J = 6.0, 1.3 Hz, 2H, Ar**H**^{biphenyl}**),** 3.33 (h, ^3J = 6.9 Hz, 8H, C**H**$^{i\text{-Pr}}$), 1.37 (s, 36H, C**H**$_3^{t\text{-Bu}}$), 1.29 (d, ^3J = 7.0 Hz, 24H, C**H**$_3^{i\text{-Pr}}$), 1.22 (d, ^3J = 6.8 Hz, 24H, C**H**$_3^{i\text{-Pr}}$) ppm.

^{13}C NMR (150.92 MHz, C_6D_6, 298K): δ = 168.4 (**C**$^{t\text{-Bu}}$), 148.5 (Ar**C**-N), 138.5 (Ar**C**-C), 133.0 (Ar**C**H^{biphenyl}), 125.7 (Ar**C**H), 122.6 (Ar**C**H), 110.1 (Ar**C**H^{biphenyl}), 109.6 (Ar**C**-C^{biphenyl}), 93.9 (**C**H^{backbone}), 86.1 (ArCH^{biphenyl}), 44.0 (**C**(CH_3)$_3$), 32.8 (C(**C**H_3)$_3$), 28.0 (**C**H$_3^{i\text{-Pr}}$), 25.9 (**C**H$^{i\text{-Pr}}$), 23.8 (**C**H$_3^{i\text{-Pr}}$) ppm.

Elemental analysis for $C_{82}H_{116}N_4Ba_2$ (M = 1432.51 g/mol): Calculated: C 68.75; H 8.16; N 3.91 %. Found: C 69.21; H 8.00; N 3.47 %.

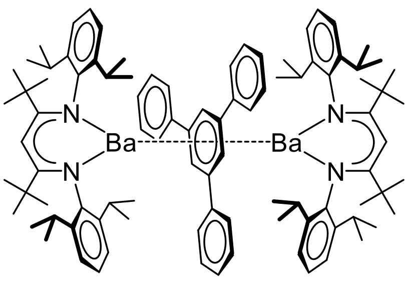

[{(^{DIPP}BDI*)Ba}$_2$(η^6:η^6-1,3,5-$Ph_3C_6H_3$)] [(^{DIPP}BDI*)Ba(μ-I)]$_2$ (100 mg, 62 µmol) and K/KI (5% w/w, 195 mg, 250 µmol) were placed in a vessel equipped with 4 stainless steel balls and ground over 30 minutes with ball-mill speed 1800 rpm (30 Hz). To the obtained dark green powder, 200 µL (226 µmol) of benzene was added and after 5 minutes the resulting dark brown slurry was extracted with 5 mL of cyclohexane. To the dark brown extract, 1,3,5-triphenylbenzene (9 mg, 30 µmol) was added and the resulting solution changed color to dark deep-turquoise. The sample was dried *in vacuo* and the residue was dissolved with 2 mL of *n*-pentane and placed at -35 °C. After two weeks, the microcrystalline black solid that had formed was isolated by decantation, washed with cold *n*-pentane (-35 °C, 2x 0.2 mL) and dried *in vacuo*. Yield: 43.2% (42 mg, 27 µmol).

^{1}H NMR (600.13 MHz, C_6D_6, 298K): δ = 6.78 (d, ^3J = 7.6 Hz, 8H, Ar**H**), 6.69 (t, ^3J = 7.7 Hz, 4H, Ar**H**), 5.84 (t, ^3J = 7.6 Hz, 6H, Ar**H**$^{1,3,5\text{-tpb}}$), 5.55 (d, ^3J = 8.2 Hz, 6H, Ar**H**$^{1,3,5\text{-tpb}}$), 5.46 (s, 2H,

C**H**^backbone^), 4.93 (t, ^3J = 7.0 Hz, 3H, Ar**H**$^{1,3,5\text{-tpb}}$), 3.95 (s, 3H, Ar**H**$^{1,3,5\text{-tpb}}$), 3.50 (h, ^3J = 6.9 Hz, 8H, C**H**$^{i\text{-Pr}}$), 1.42 (s, 36H, C**H₃**$^{t\text{-Bu}}$), 1.22 (d, ^3J = 7.0 Hz, 24H, C**H₃**$^{i\text{-Pr}}$), 0.96 (d, ^3J = 6.9 Hz, 24H, C**H₃**$^{i\text{-Pr}}$) ppm.

^{13}C NMR (150.92 MHz, C_6D_6, 298K): δ = 168.9 (**C**$^{t\text{-Bu}}$), 148.7 (Ar**C**-N), 138.9 (Ar**C**-C), 130.5 (Ar**CH**$^{1,3,5\text{-tpb}}$), 129.4 (Ar**C**-C$^{1,3,5\text{-tpb}}$), 129.1 (Ar**C**-C$^{1,3,5\text{-tpb}}$), 125.6 (Ar**C**H), 124.8 (Ar**CH**$^{1,3,5\text{-tpb}}$), 122.6 (ArCH), 115.4 (Ar**CH**$^{1,3,5\text{-tpb}}$), 102.8 (Ar**CH**$^{1,3,5\text{-tpb}}$), 94.4 (**C**H^{backbone}), 44.1 (**C**(CH_3)$_3$), 32.9 (C(**C**H_3)$_3$), 28.1 (**C**$H_3^{i\text{-Pr}}$), 25.3 (**C**H$^{i\text{-Pr}}$), 23.7 (**C**$H_3^{i\text{-Pr}}$) ppm. Ar**C**-**C**$^{1,3,5\text{-tpb}}$ signals were not detected.

Elemental analysis for $C_{94}H_{124}N_4Ba_2$ (M = 1584.71 g/mol): Calculated: C 71.25; H 7.89; N 3.54 %. Found: C 69.71; H 8.55; N 3.14 %.

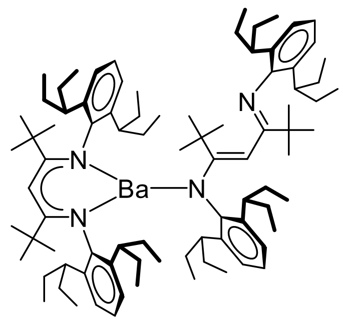

[(κ^2,κ^1-^{DIPeP}BDI*)$_2$Ba] *Preparation via a salt metathesis protocol:* [(^{DIPeP}BDI*)K]$_4$ (131 mg, 50 µmol) was dissolved in benzene (10 mL), then BaI_2 (39 mg, 100 µmol) was added and the resulting suspension was refluxed for 3 days. The mixture was cooled to room temperature and filtered. The filtrate was evaporated to dryness under vacuum. The residue contained a mixture of [(^{DIPeP}BDI*)K]$_4$, [(^{DIPeP}BDI*)Ba(μ-I)]$_2$ and [(κ^2,κ^1-^{DIPeP}BDI*)$_2$Ba] in ratio *ca*. 8:1:2, from which the latter could not be completely separated (**Figure S84**). Longer refluxing times did not change the ratio towards target homoleptic compound. Therefore, salt-metathesis is not the method of choice.

Preparation via decomposition of in situ generated [{(^{DIPeP}BDI)Ba}$_2$(η^6:η^6-1,3,5-$Ph_3C_6H_3$)]:* [(^{DIPeP}BDI*)Ba(μ-I)]$_2$ (110 mg, 63 µmol) and K/KI (5% w/w, 195 mg, 250 µmol) were placed in a vessel equipped with 4 stainless steel balls and ground over 30 minutes with ball-mill speed 1800 rpm (30 Hz). To the obtained dark green powder 5 mL of benzene was added and after 5 minutes the resulting dark brown mixture was filtered off. To the dark brown filtrate, 1,3,5-triphenylbenzene (10 mg, 32 µmol) was added. The resulting solution changed color to turquoise which faded within seconds to a bright red with formation of a black precipitate on the walls of the flask. The solution, which contains the product and residual traces of 1,3,5-$Ph_3C_6H_3$ (**Figure S93**) was separated and the solvent was removed *in vacuo* to give the product as a yellow solid. Yield: 88.1% (76 mg, 56 µmol). The black precipitate on the walls of the flask is likely insoluble [Ba(1,3,5-$Ph_3C_6H_3$)]$_n$ (see Figure S94).

^{1}H NMR (600.13 MHz, C_6D_{12}, 298K): δ = 7.27 (d, ^3J = 7.5 Hz, 1H, Ar**H**), 7.13 (t, ^3J = 6.9 Hz, 2H, Ar**H**), 7.05 (d, ^3J = 5.9 Hz, 1H, Ar**H**), 7.13 (t, ^3J = 7.5 Hz, 2H, Ar**H**), 6.95 – 6.88 (m, 2H, Ar**H**), 6.85 (t, ^3J = 7.6 Hz, 2H, Ar**H**), 6.83 – 6.78 (m, 2H, Ar**H**), 5.28 (s, 2H, C**H**^{backbone}), 4.36 (s, 2H, C**H**^{backbone}), 3.36 – 3.28 (m, 1H, C**H**$^{3\text{-Pent}}$), 3.20 – 3.08 (m, 2H, C**H**$^{3\text{-Pent}}$), 3.01 – 2.90 (m, 3H, C**H**$^{3\text{-Pent}}$), 2.80 – 2.73 (m, 1H, C**H**$^{3\text{-Pent}}$), 2.57 – 2.45 (m, 1H, C**H**$^{3\text{-Pent}}$), 1.96 (s, 9H, C**H₃**$^{t\text{-Bu}}$), 1.94 – 1.30 (m, 32H, C**H₂**$^{3\text{-Pent}}$), 1.22 (t, ^3J = 7.4 Hz, 6H, C**H₃**$^{3\text{-Pent}}$), 1.18 (t, ^3J = 8.1 Hz, 6H, C**H₃**$^{3\text{-Pent}}$), 1.14 (s, 9H, C**H₃**$^{t\text{-Bu}}$), 1.12 (s, 9H, C**H₃**$^{t\text{-Bu}}$), 1.09 (s, 9H, C**H₃**$^{t\text{-Bu}}$), 1.01 – 0.95 (m, 12H, C**H₃**$^{3\text{-Pent}}$), 0.91 – 0.86 (m, 6H, C**H₃**$^{3\text{-Pent}}$), 0.85 – 0.79 (m, 6H, C**H₃**$^{3\text{-Pent}}$), 0.76 (t, ^3J = 7.3 Hz, 3H, C**H₃**$^{3\text{-Pent}}$), 0.71 (t, ^3J = 7.1 Hz, 3H, C**H₃**$^{3\text{-Pent}}$), 0.32 (t, ^3J = 7.6 Hz, 6H, C**H₃**$^{3\text{-Pent}}$) ppm.

^{13}C NMR (150.92 MHz, C_6D_{12}, 298K): δ = 176.7, 174.3, 168.6, 165.3 ((*t*-Bu)**C**), 152.9, 152.0, 149.2, 147.1 (Ar**C**-N), 145.7, 136.7, 135.4, 135.2, 133.9, 133.1, 132.8, (Ar**C**-C), 129.5, 129.3, 128.1, 127.2, 126.4, 126.0, 125.9, 124.8, 124.1, 123.9, 122.4, 122.2, 118.5, 118.0 (Ar**C**H), 93.3,

88.9 (**C**H^{backbone}), 44.9, 44.7, 44.4, 40.9 (**C**(CH_3)$_3$), 42.2, 41.3, 41.2, 41.1, 40.1, 39.3, 38.3, 36.5, 34.4, 32.9, 31.8, 31.6, 30.3, 29.9, 29.1, 27.8, 27.5, 26.7, 26.2, 25.7, 24.9, 24.7, 24.1, 23.8, 23.2, 23.2, 22.8, 22.7 (**C**$H_2^{3\text{-Pent}}$), 26.7 (**C**$H_2^{3\text{-Pent}}$), 32.26, 32.07, 31.31, 31.02 (C(**C**H_3)$_3$), 14.3, 13.5, 13.4, 13.4, 13.3, 12.3, 12.2, 12.0, 11.9, 11.4, 11.2, 11.1, 9.4, 8.8, 8.1, 7.4, 7.1 (**C**$H_3^{3\text{-Pent}}$) ppm.

Elemental analysis for $C_{86}H_{138}N_4Ba$ (M = 1365.41 g/mol): Calculated: C 75.65; H 10.19; N 4.10 %. Found: C 74.56; H 9.90; N 4.67 %.

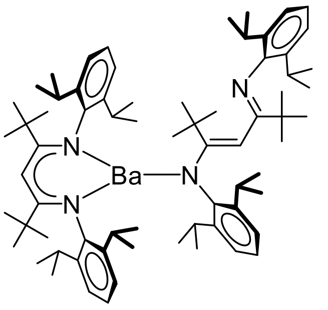

[(*κ*^{2},*κ*^{1}-^{DIPP}BDI*)$_2$Ba] [(^{DIPP}BDI*)-H] (101 mg, 200 µmol) and benzyl potassium (26 mg, 200 µmol) were dissolved in diethyl ether (5 mL) and stirred for 6h at room temperature until the red color of the solution disappeared. The solvent was removed *in vacuo*, then the resulting solid and BaI_2 (39 mg, 100 µmol) were suspended in 2 mL of benzene and refluxed for 3 days. The mixture was cooled to room temperature and filtered. The solvent of the filtrate was removed *in vacuo* and the residue was extracted with 5 mL of *n*-pentane. The *n*-Pentane extract was kept at -35 °C. Overnight, multiple small yellow needle-like crystals formed (not suitable for X-ray measurement), which were isolated by decantation, washed with cold *n*-pentane (-35 °C, 2x 0.2 mL) and dried *in vacuo* (49 mg, 43 µmol). The filtrate was concentrated to 2 mL and kept at -35 °C to give a second crop of crystals over few days (17 mg, 15 µmol). Overall yield: 58.0% (66 mg, 58 µmol).

^{1}H NMR (600.13 MHz, C_6D_{12}, 298K): δ = 7.21 (d, ^3J = 7.7 Hz, 2H, Ar**H**), 7.03 (t, ^3J = 7.6 Hz, 2H, Ar**H**), 7.00 (t, ^3J = 7.6 Hz, 2H, Ar**H**), 6.95 (br s, 4H, Ar**H**), 6.93 – 6.88 (m, 2H, Ar**H**), 5.38 (s, 1H, C**H**^{backbone}), 4.21 (s, 1H, C**H**^{backbone}), 3.44 (p, ^3J = 6.8 Hz, 2H, C**H**$^{i\text{-Pr}}$), 3.08 (br s, 6H, C**H**$^{i\text{-Pr}}$), 1.90 (s, 9H, C**H$_3$**$^{t\text{-Bu}}$), 1.43 (d, ^3J = 4.6 Hz, 12H, C**H$_3$**$^{i\text{-Pr}}$), 1.38 (br s, 12H, C**H$_3$**$^{i\text{-Pr}}$), 1.12 (br s, 6H, C**H$_3$**$^{i\text{-Pr}}$), 1.09 (s, 9H, C**H$_3$**$^{t\text{-Bu}}$), 1.07 (s, 18H, C**H$_3$**$^{t\text{-Bu}}$), 0.98 (br s, 6H, C**H$_3$**$^{i\text{-Pr}}$), 0.66 (br s, 12H, C**H$_3$**$^{i\text{-Pr}}$) ppm.

^{13}C NMR (150.92 MHz, C_6D_{12}, 298K): δ = 175.4 ((*t*-Bu)**C**), 165.1 ((*t*-Bu)**C**), 150.2 (Ar**C**-N), 147.5 (Ar**C**-N), 136.9 (Ar**C**-C), 134.9 (Ar**C**-C), 124.3 (Ar**C**H), 123.9 (Ar**C**H), 123.0 (Ar**C**H), 119.3 (Ar**C**H), 94.3 (**C**H^{backbone}), 90.5 (**C**H^{backbone}), 44.8 (**C**(CH_3)$_3$), 44.4 (**C**(CH_3)$_3$), 40.7 (**C**(CH_3)$_3$), 31.9 (C(**C**H_3)$_3$), 31.3 (C(**C**H_3)$_3$), 29.9 (C(**C**H_3)$_3$), 28.4 (**C**H$^{i\text{-Pr}}$), 27.6 (**C**H$^{i\text{-Pr}}$), 26.5 (**C**$H_3^{i\text{-Pr}}$), 23.3 (**C**$H_3^{i\text{-Pr}}$) ppm.

Elemental analysis for $C_{92}H_{144}N_4Ba_2$ (M = 1140.97 g/mol): Calculated: C 73.69; H 9.36; N 4.91 %. Found: C 73.96; H 9.23; N 5.26 %.

1.3 NMR Characterisation

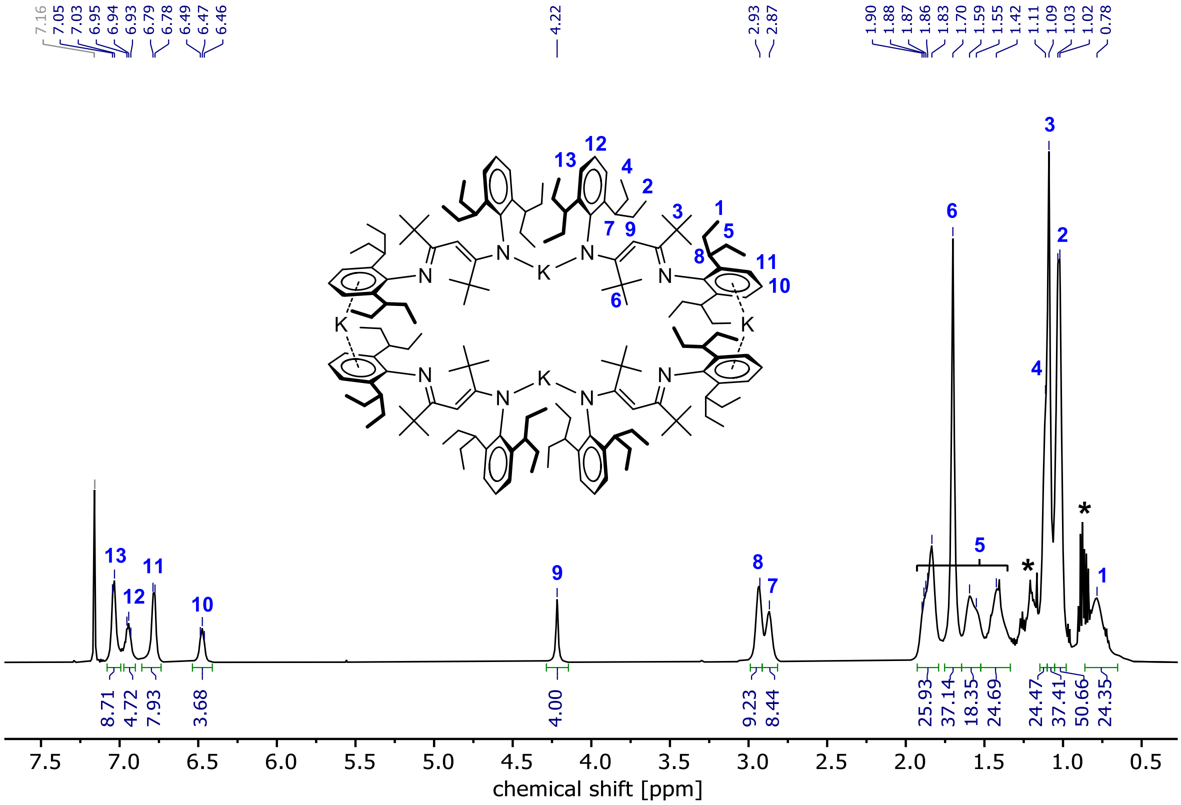

Figure S1. ^{1}H NMR (600.13 MHz, 298 K, C_6D_6) of [(^{DIPeP}BDI*)K]$_4$. * denotes hexanes.

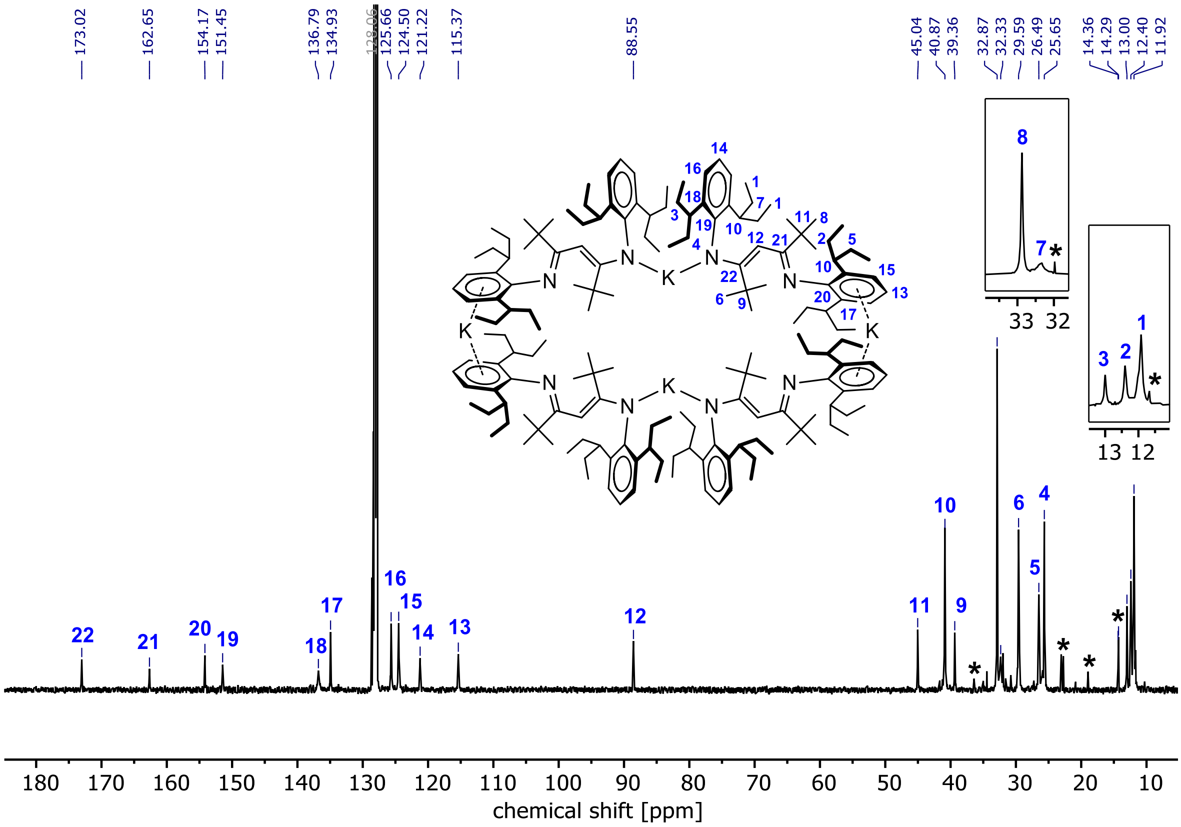

Figure S2. ^{13}C NMR (150.92 MHz, 298 K, C_6D_6) of [(^{DIPeP}BDI*)K]$_4$. * denotes hexanes.

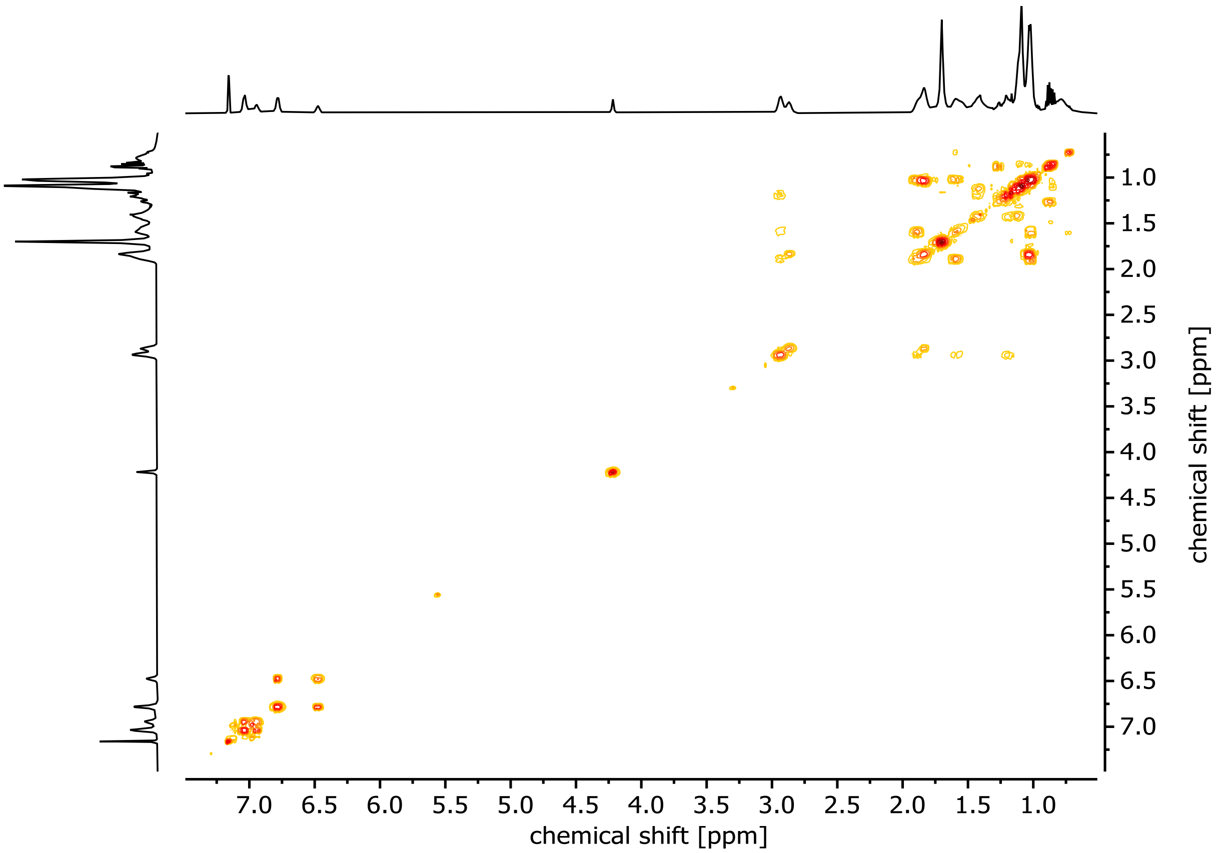

Figure S3. ^{1}H-^{1}H COSY NMR (600.13 MHz, 298 K, C_6D_6) of $[(^{DIPeP}BDI^*)K]_4$.

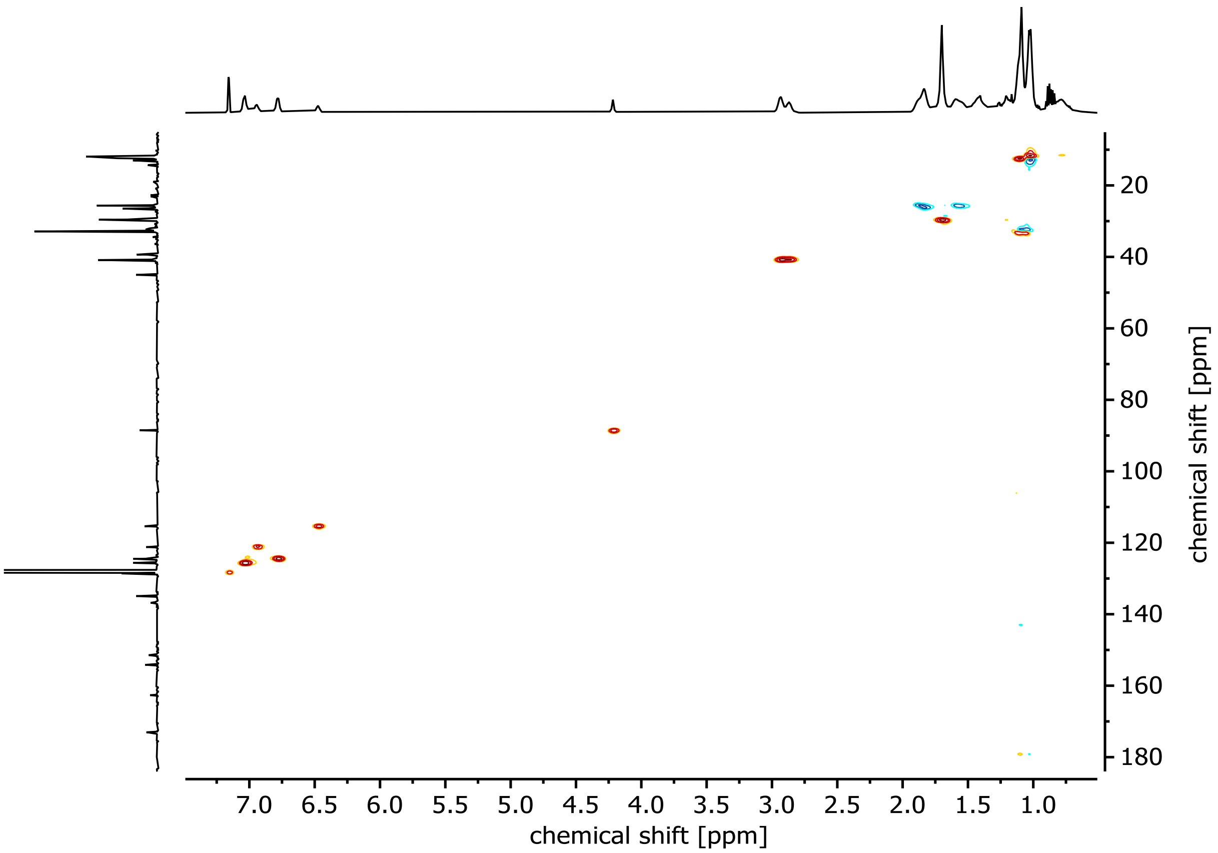

Figure S4. ^{1}H-^{13}C HSQC NMR (600.13/150.92 MHz, 298 K, C_6D_6) of $[(^{DIPeP}BDI^*)K]_4$.

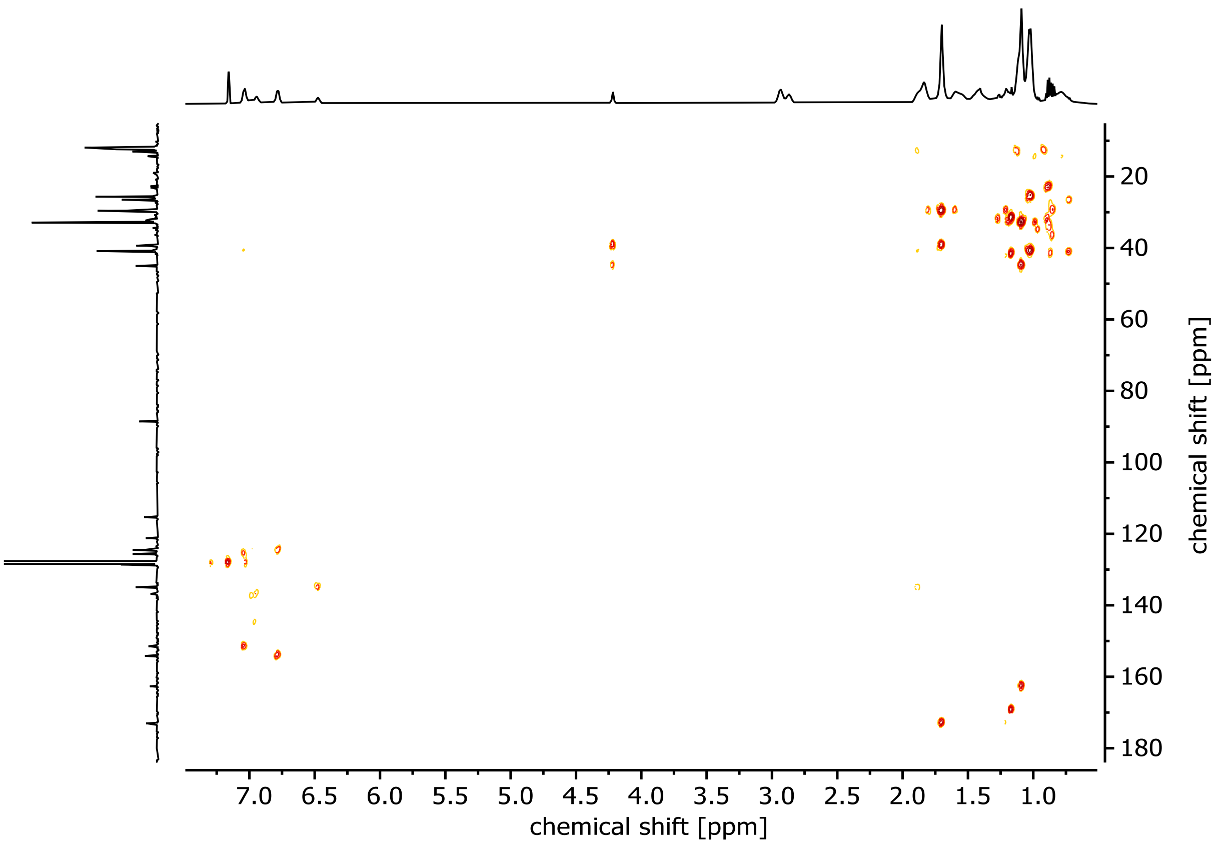

Figure S5. ^1H-^{13}C HMBC NMR (600.13/150.92 MHz, 298 K, C_6D_6) of $[(^{DIPeP}BDI^*)K]_4$.

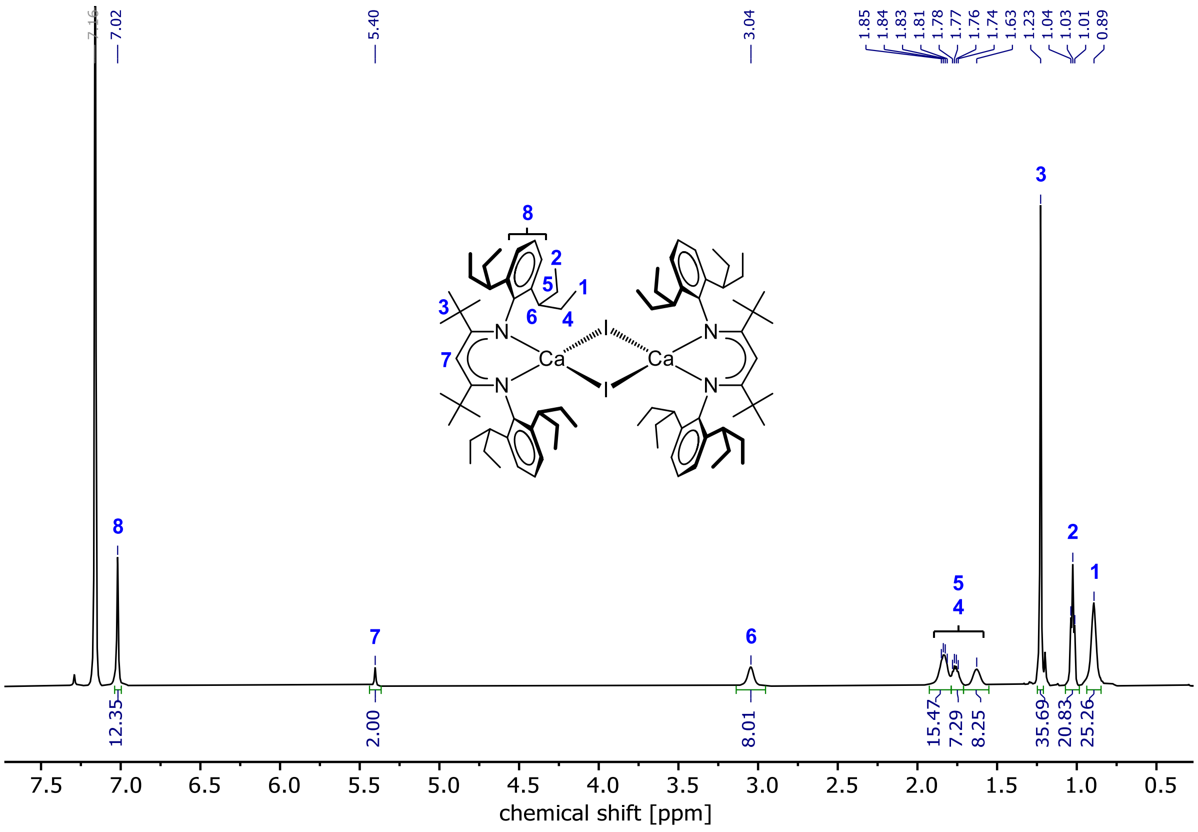

Figure S6. ^1H NMR (600.13 MHz, 323 K, C_6D_6) of $[(^{DIPeP}BDI^*)Ca(\mu\text{-}I)]_2$.

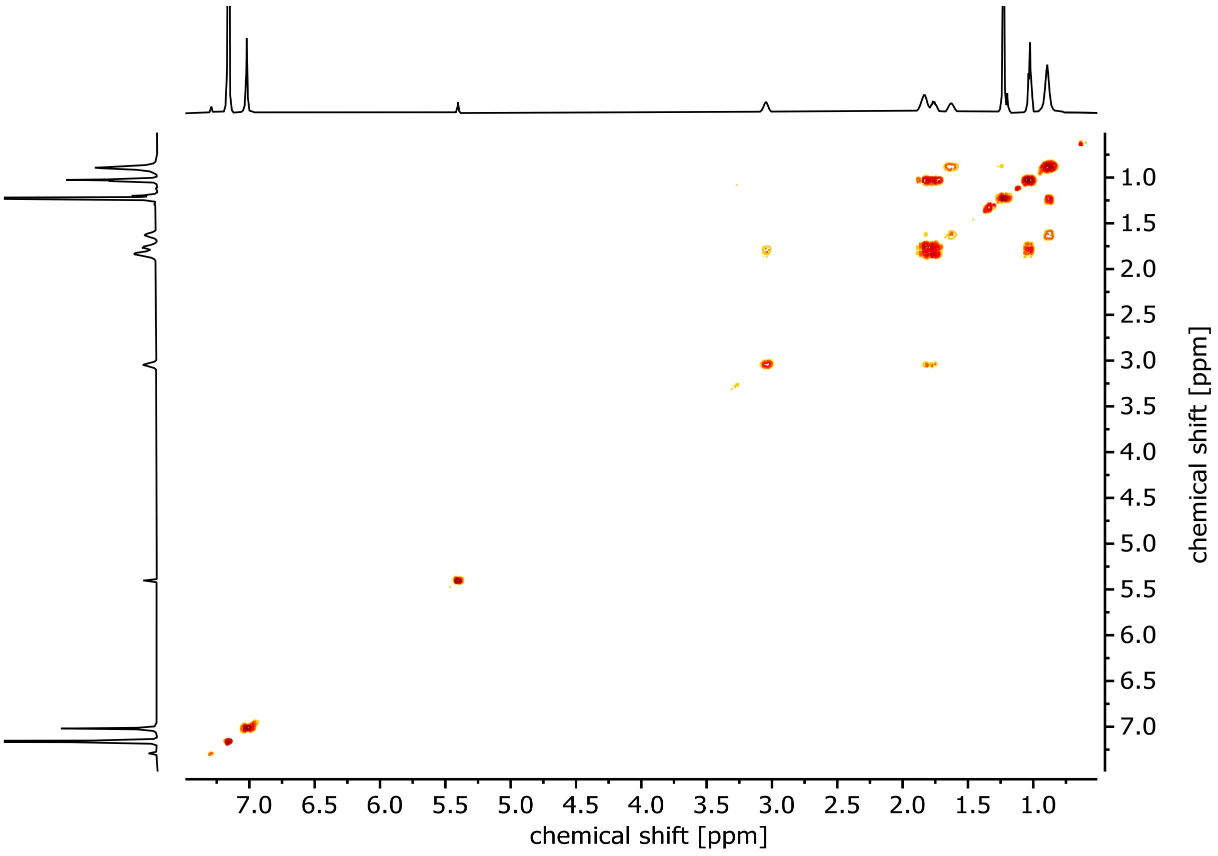

Figure S7. ^{1}H-^{1}H COSY NMR (600.13 MHz, 323 K, C_6D_6) of [(^{DIPeP}BDI*)Ca(μ-I)]$_2$.

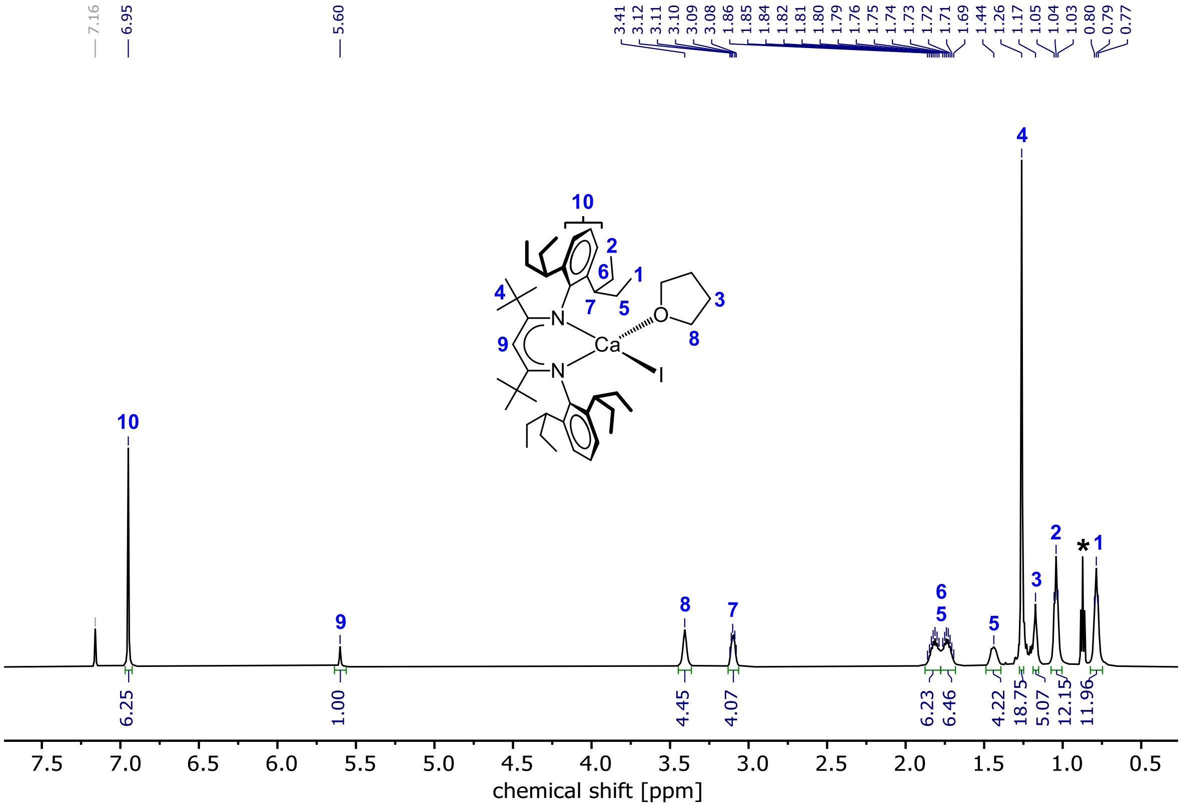

Figure S8. ^{1}H NMR (600.13 MHz, 298 K, C_6D_6) of [(^{DIPeP}BDI*)CaI · THF)]. * denotes *n*-pentane.

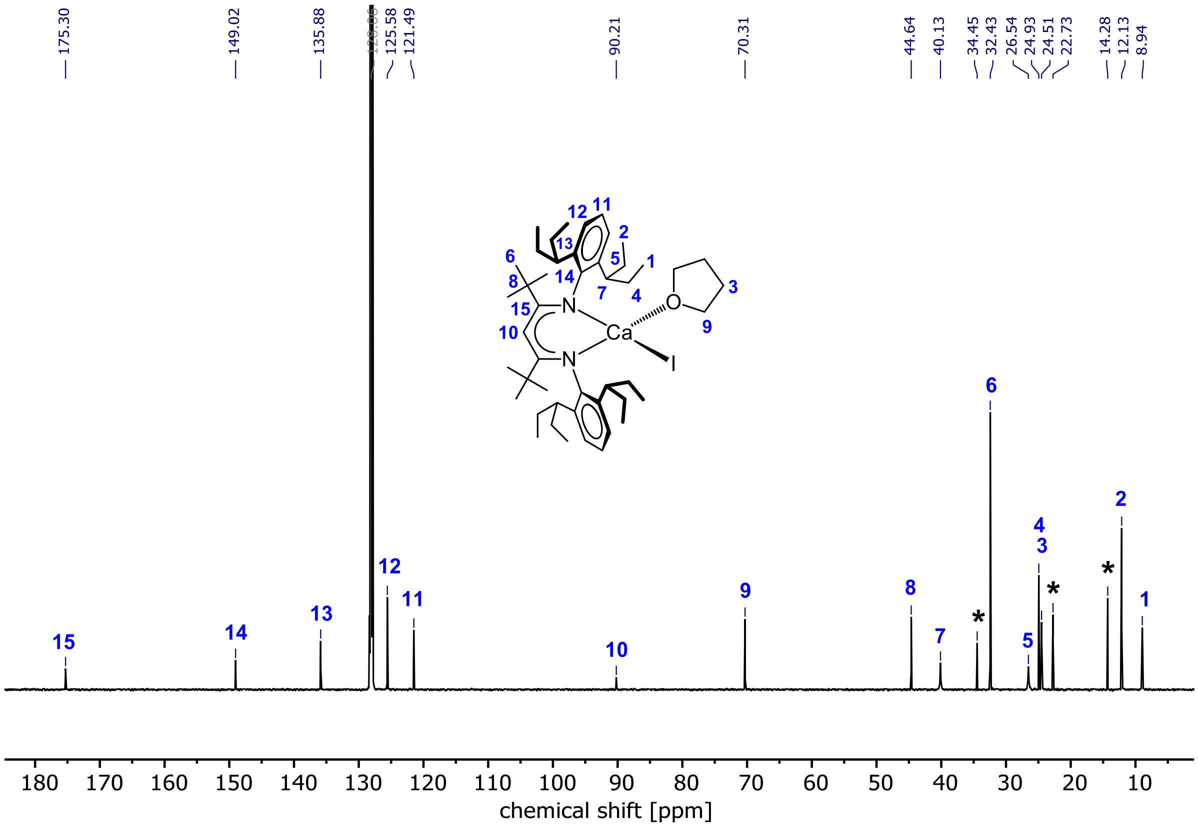

Figure S9. ^{13}C NMR (150.92 MHz, 298 K, C_6D_6) of [(^{DIPeP}BDI*)CaI·THF)]. * denotes *n*-pentane.

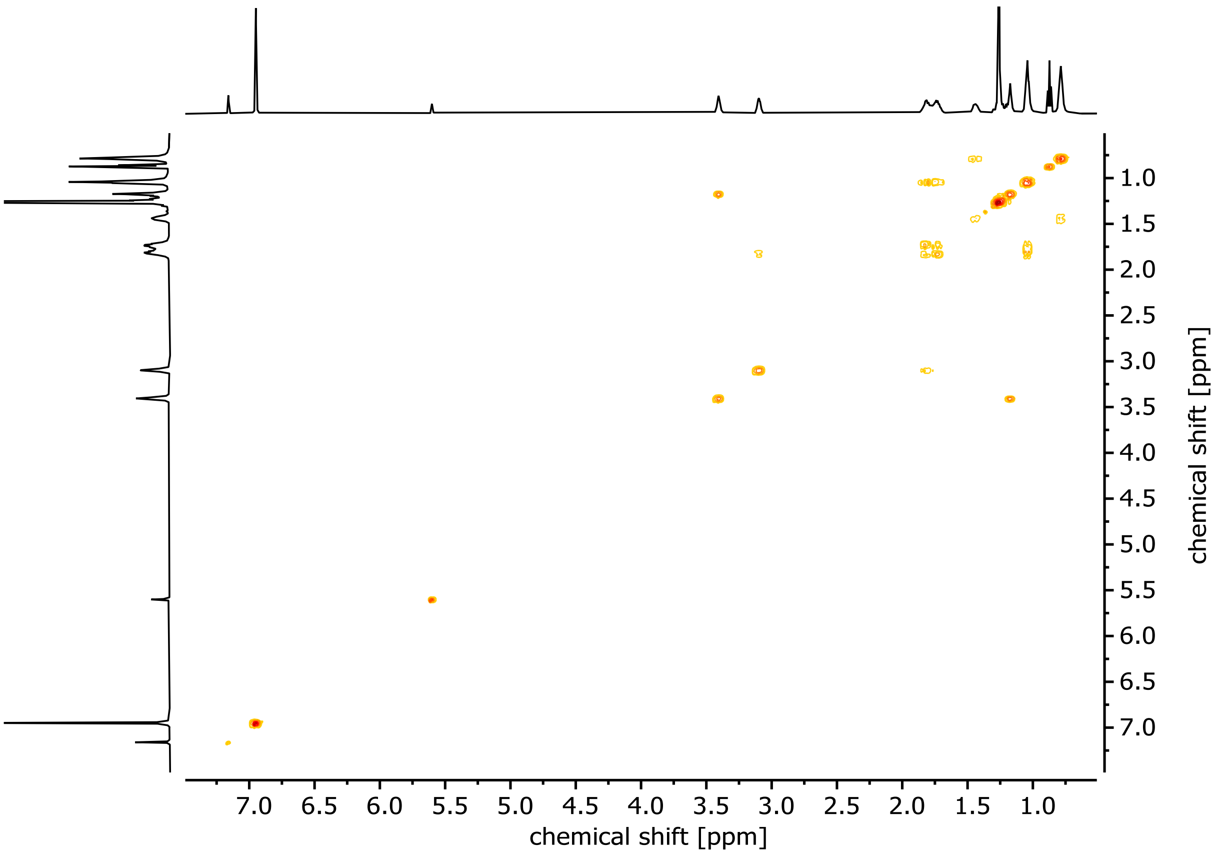

Figure S10. ^{1}H-^{1}H COSY NMR (600.13 MHz, 298 K, C_6D_6) of [(^{DIPeP}BDI*)CaI·THF)].

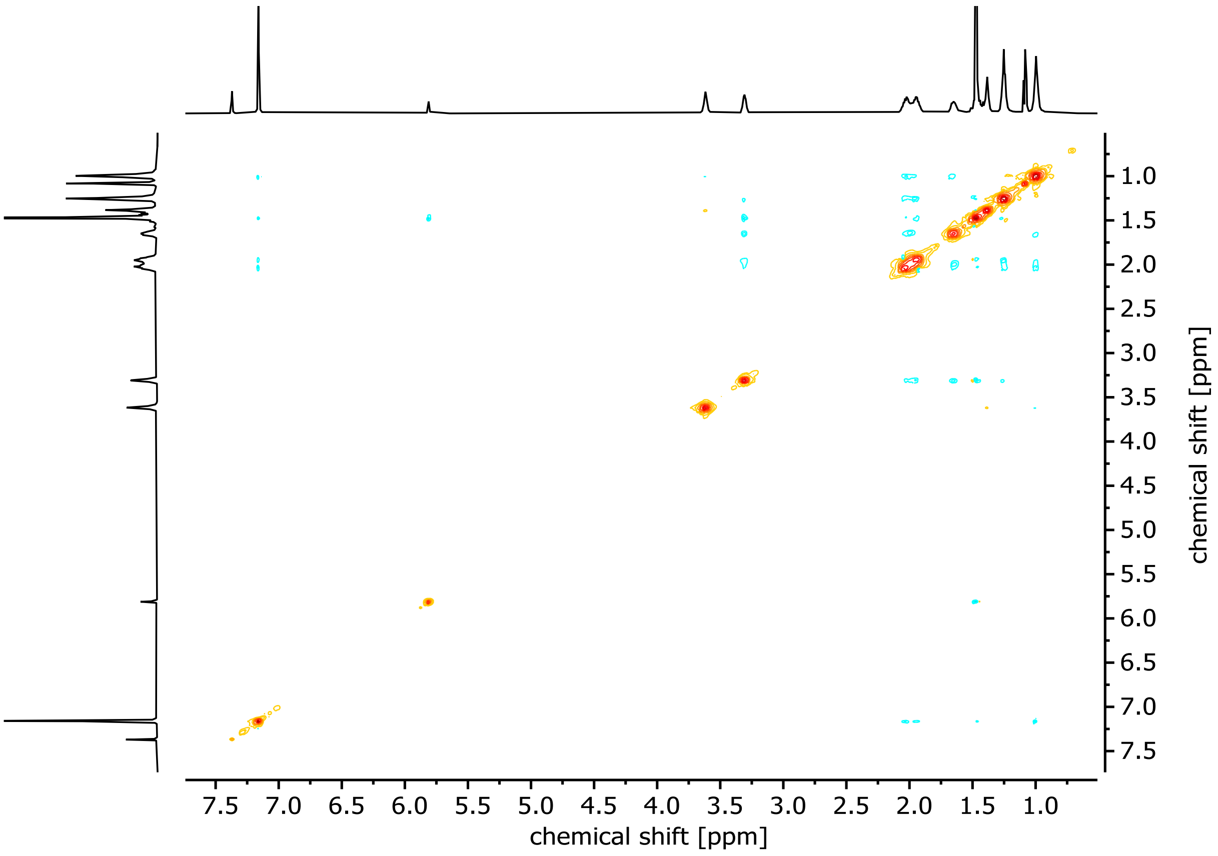

Figure S11. ^{1}H-^{1}H NOESY (600.13 MHz, 298 K, C_6D_6) of [(^{DIPeP}BDI*)CaI·THF)].

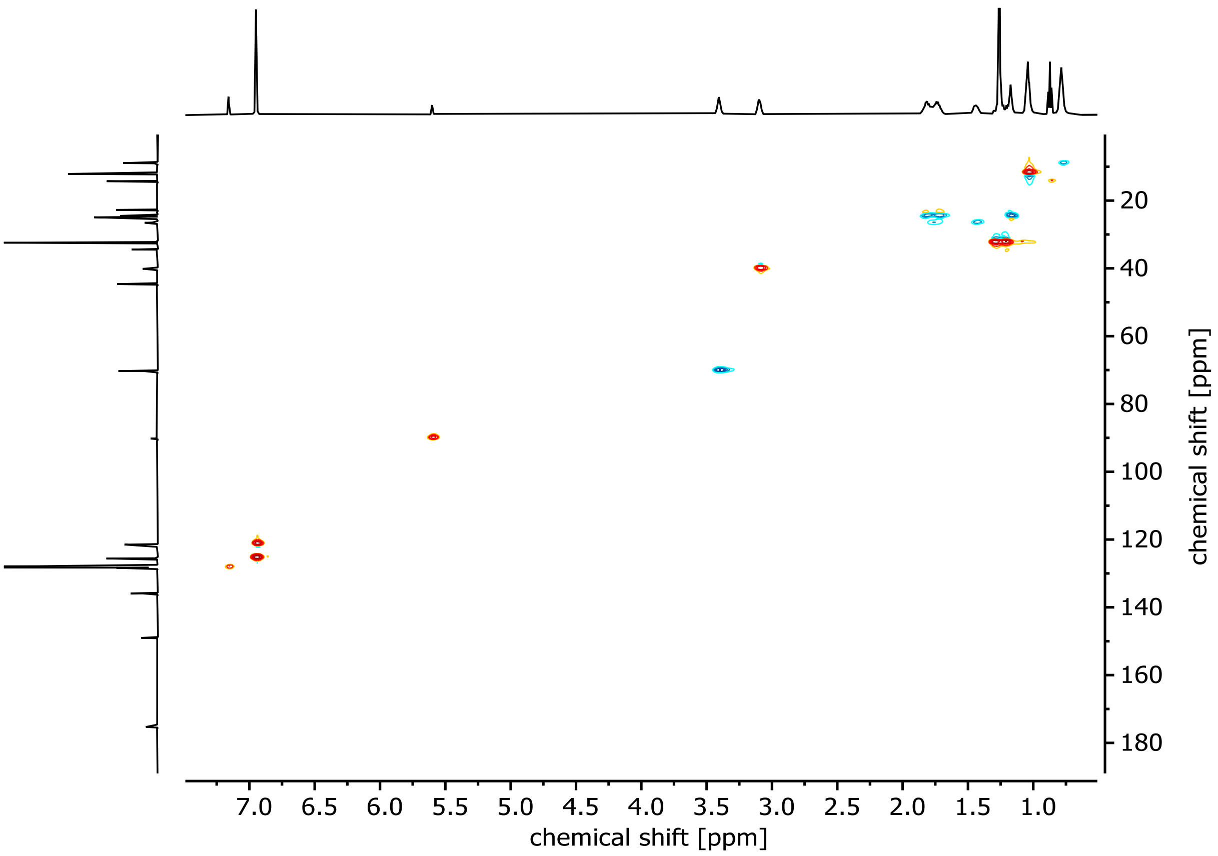

Figure S12. ^{1}H-^{13}C HSQC NMR (600.13/150.92 MHz, 298 K, C_6D_6) of [(^{DIPeP}BDI*)CaI·THF)].

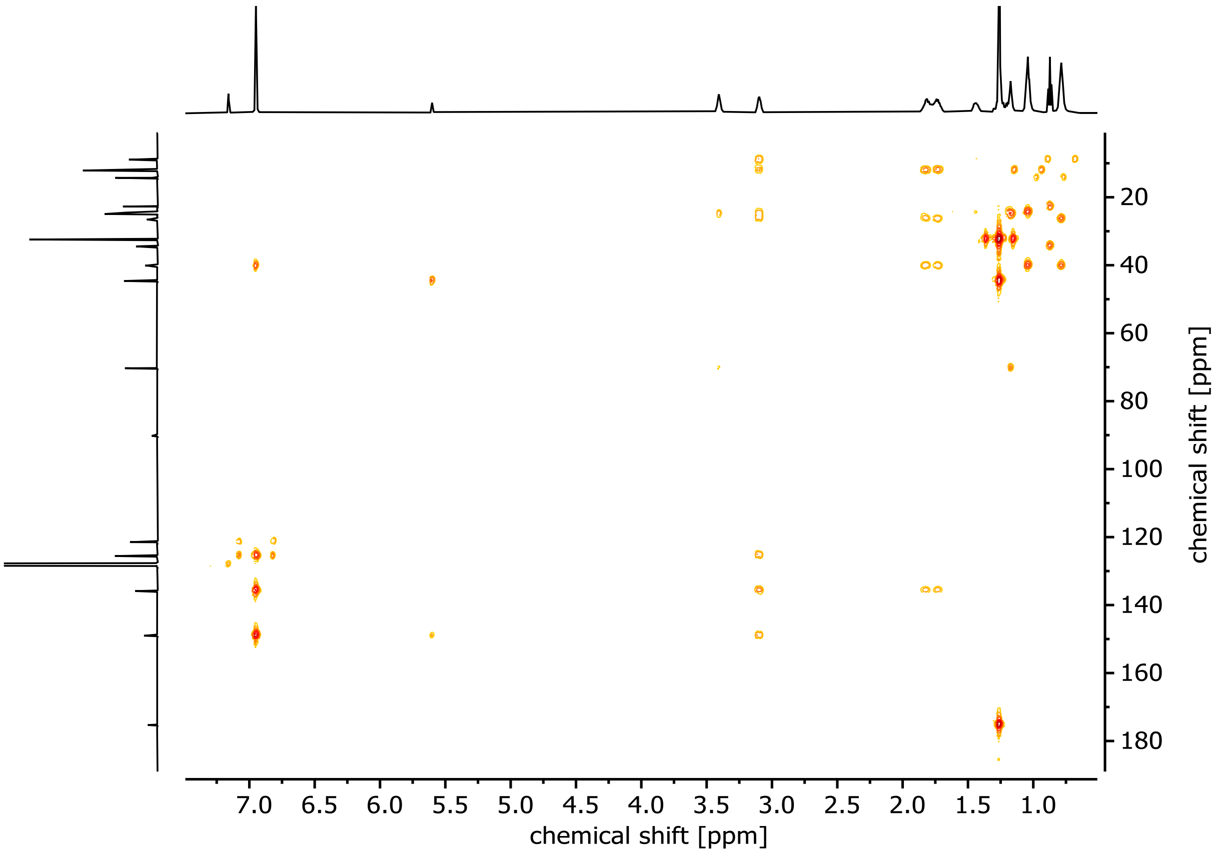

Figure S13. ^{1}H-^{13}C HMBC NMR (600.13/150.92 MHz, 298 K, C_6D_6) of [(^{DIPeP}BDI*)CaI · THF)].

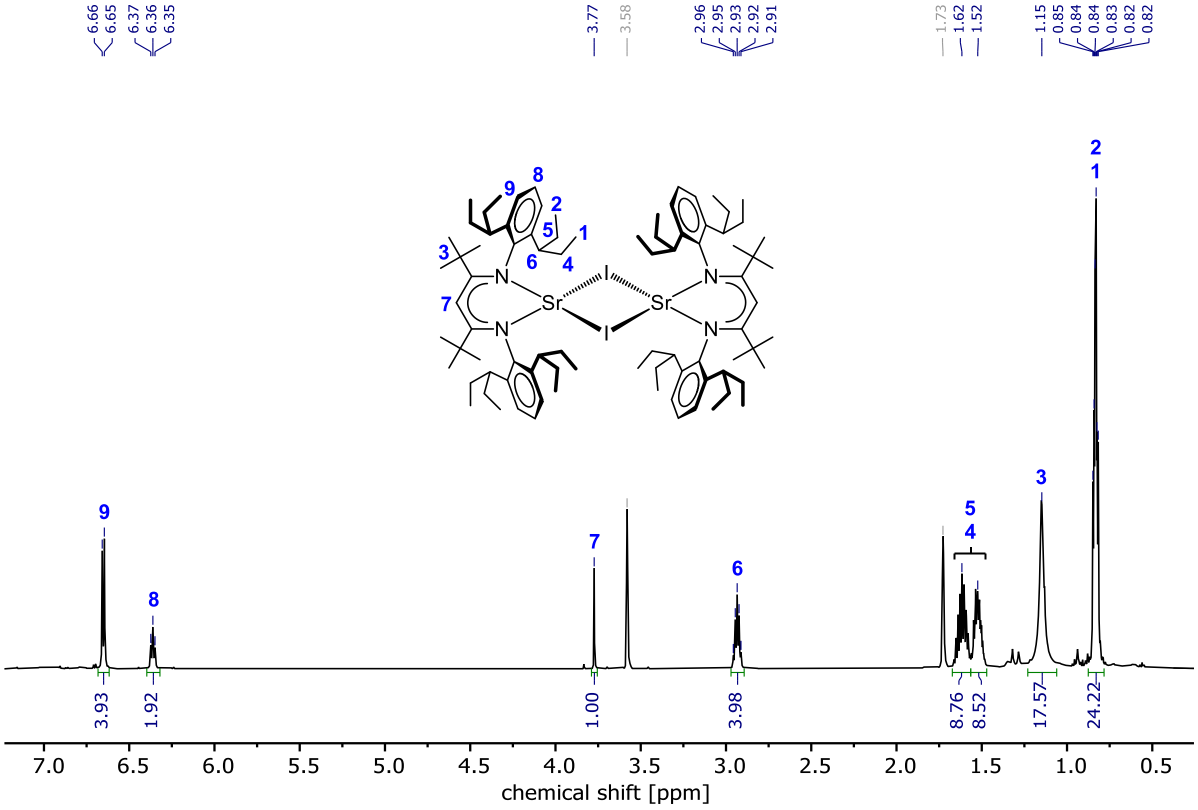

Figure S14. ^{1}H NMR (600.13 MHz, 298 K, THF-d_6) of [(^{DIPeP}BDI*)Sr(μ-I)]$_2$.

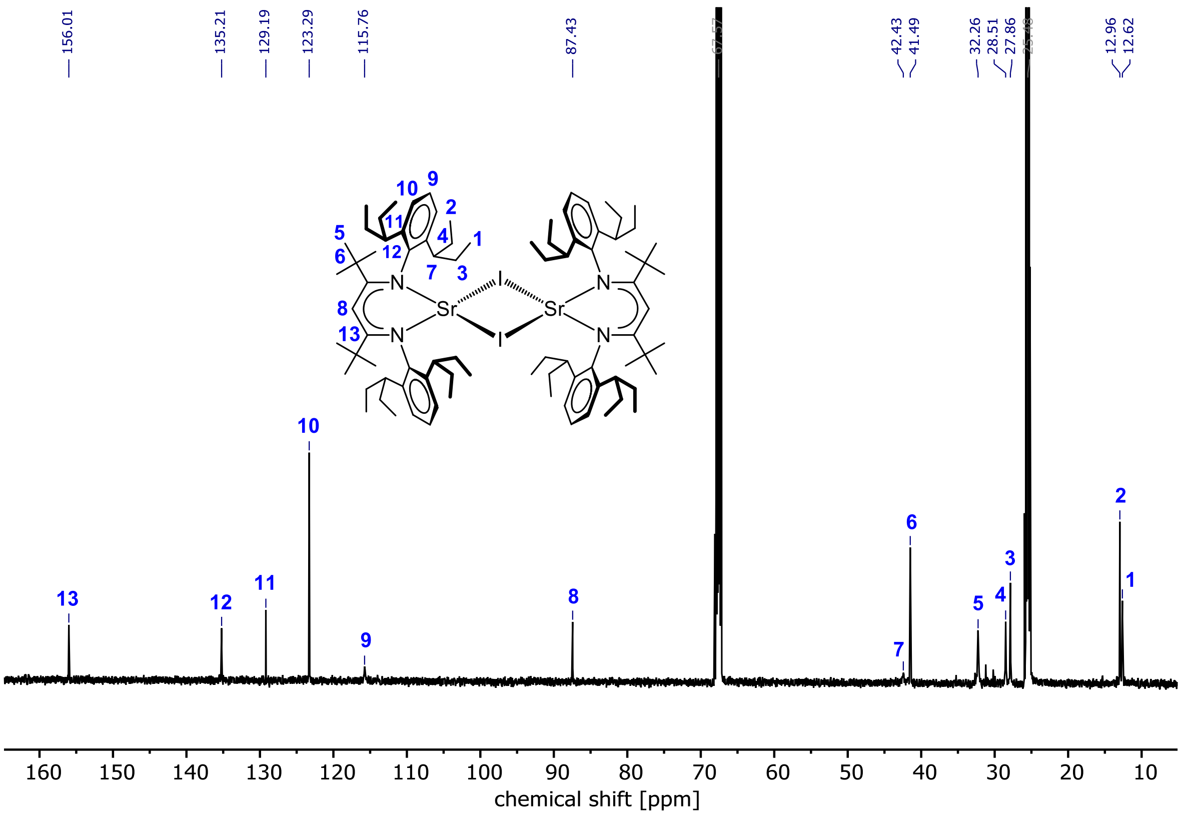

Figure S15. ^{13}C NMR (150.92 MHz, 298 K, C_6D_6) of [(^{DIPeP}BDI*)Sr(μ-I)]$_2$.

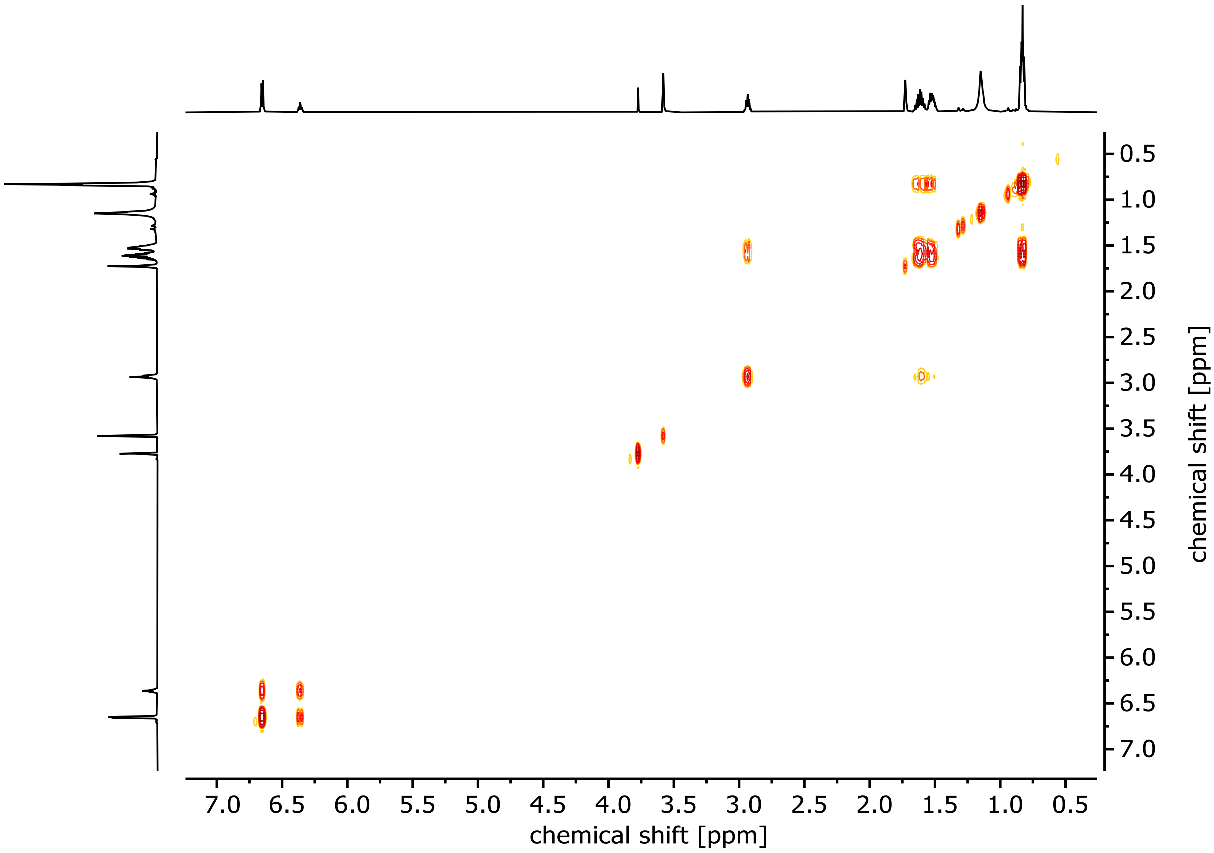

Figure S16. ^{1}H-^{1}H COSY NMR (600.13 MHz, 298 K, C_6D_6) of [(^{DIPeP}BDI*)Sr(μ-I)]$_2$.

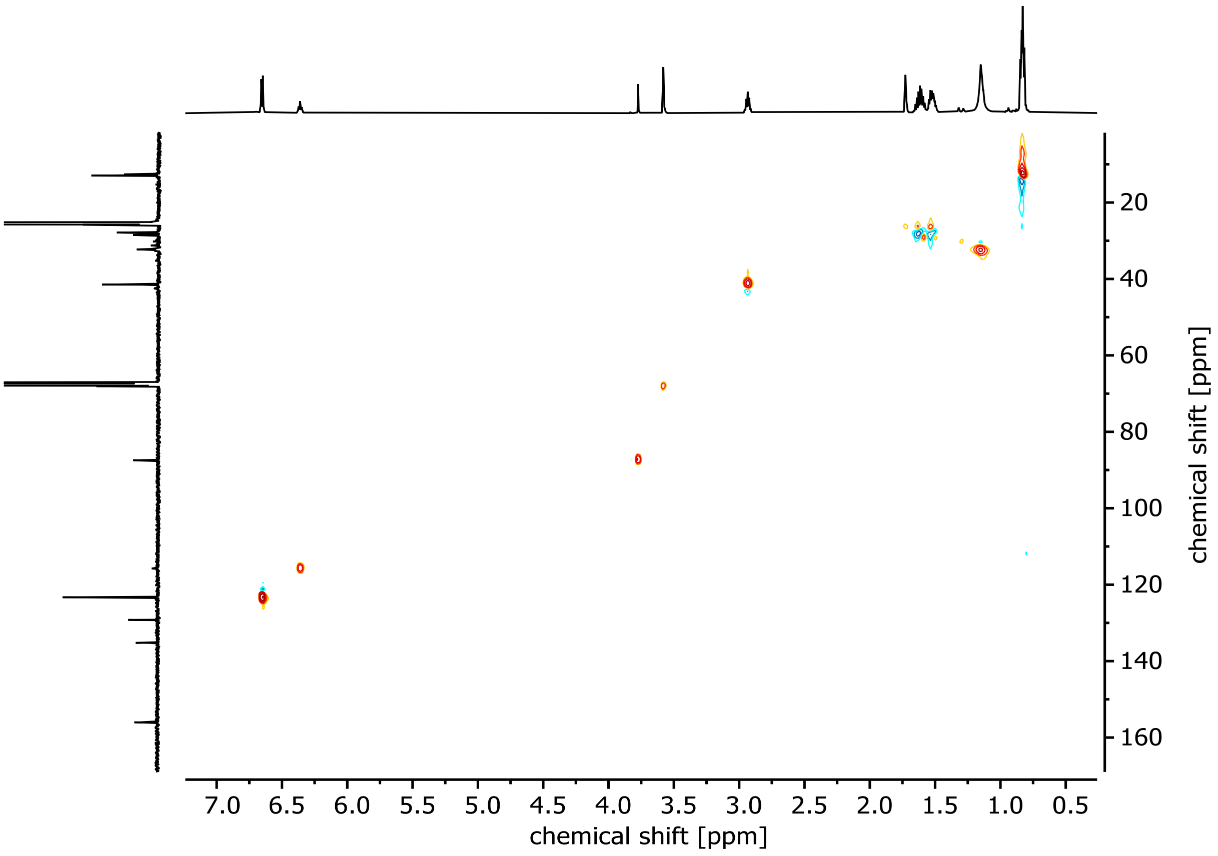

Figure S17. ^{1}H-^{13}C HSQC NMR (600.13/150.92 MHz, 298 K, C_6D_6) of $[(^{DIPeP}BDI^*)Sr(\mu\text{-}I)]_2$.

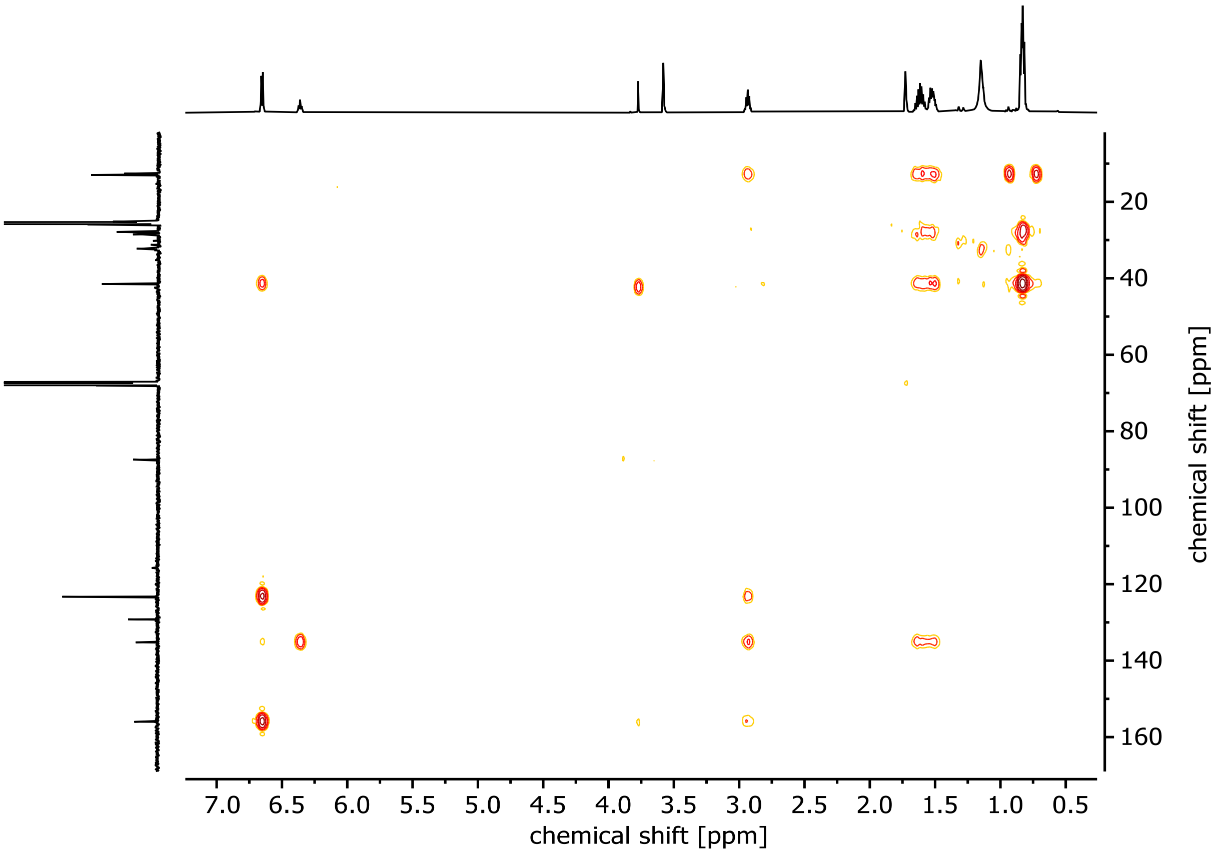

Figure S18. ^{1}H-^{13}C HMBC NMR (600.13/150.92 MHz, 298 K, C_6D_6) of $[(^{DIPeP}BDI^*)Sr(\mu\text{-}I)]_2$.

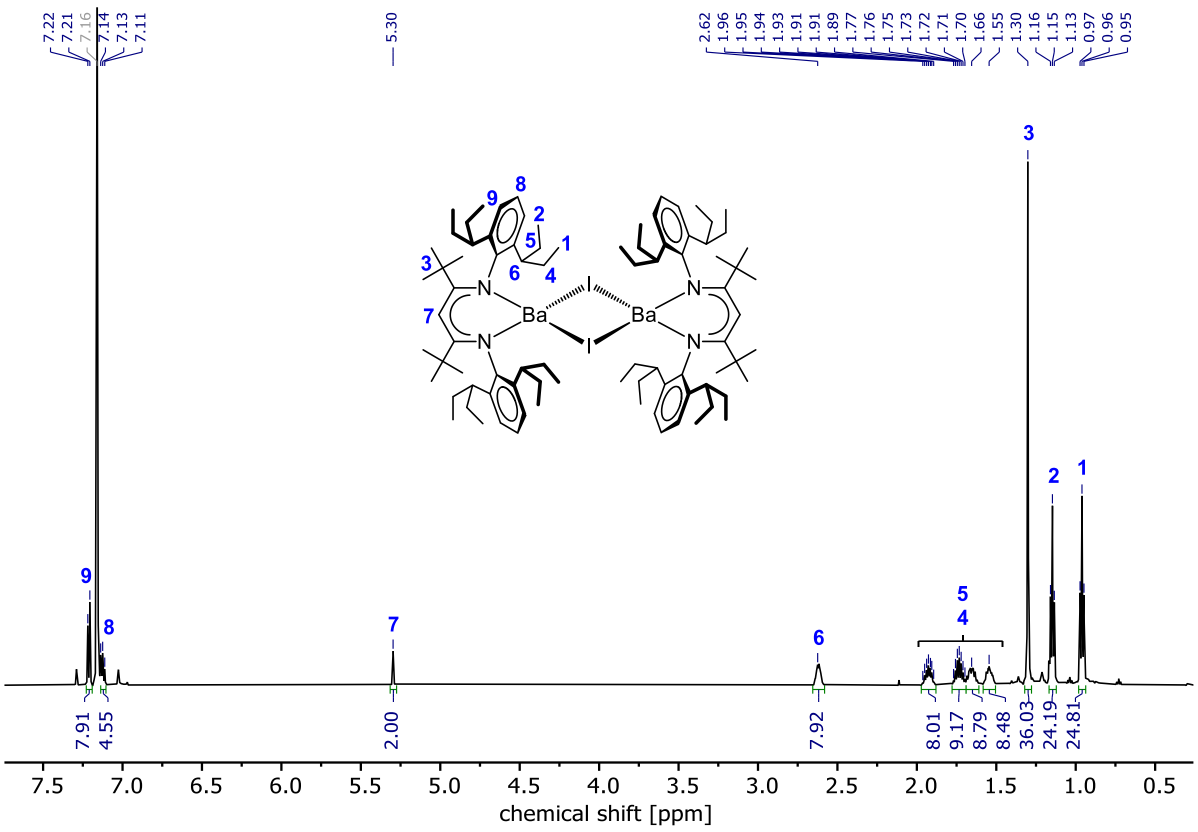

Figure S19. ^{1}H NMR (600.13 MHz, 298 K, C_6D_6) of [(^{DIPeP}BDI*)Ba(μ-I)]$_2$.

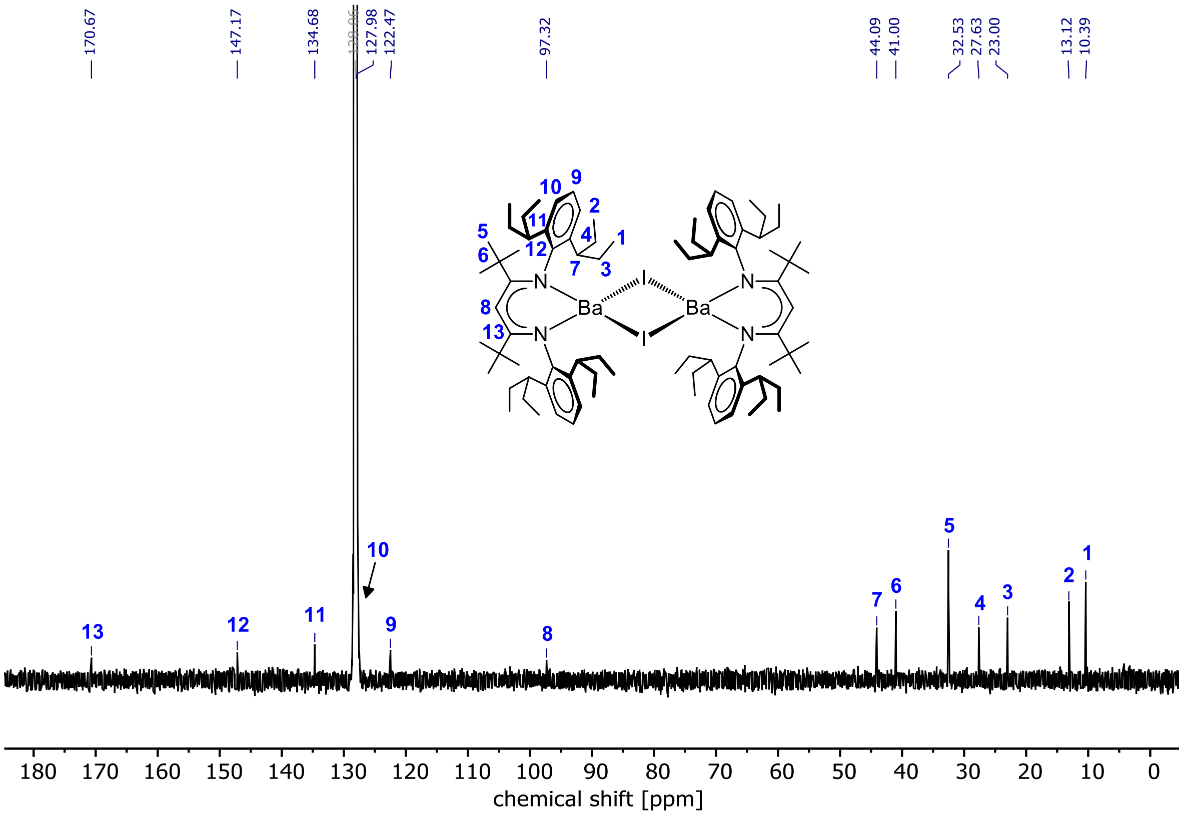

Figure S20. ^{13}C NMR (150.92 MHz, 298 K, C_6D_6) of [(^{DIPeP}BDI*)Ba(μ-I)]$_2$.

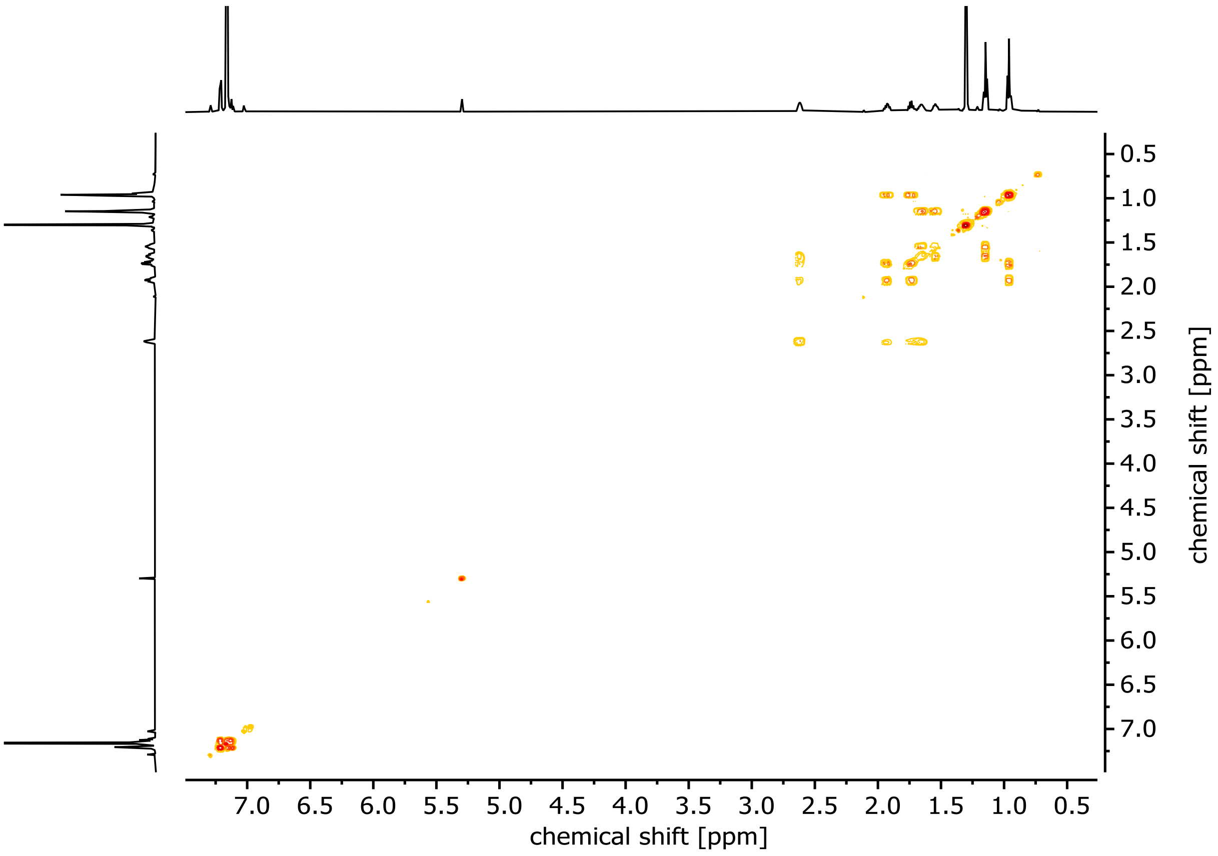

Figure S21. ^{1}H-^{1}H COSY NMR (600.13 MHz, 298 K, C_6D_6) of [(^{DIPeP}BDI*)Ba(μ-I)]$_2$.

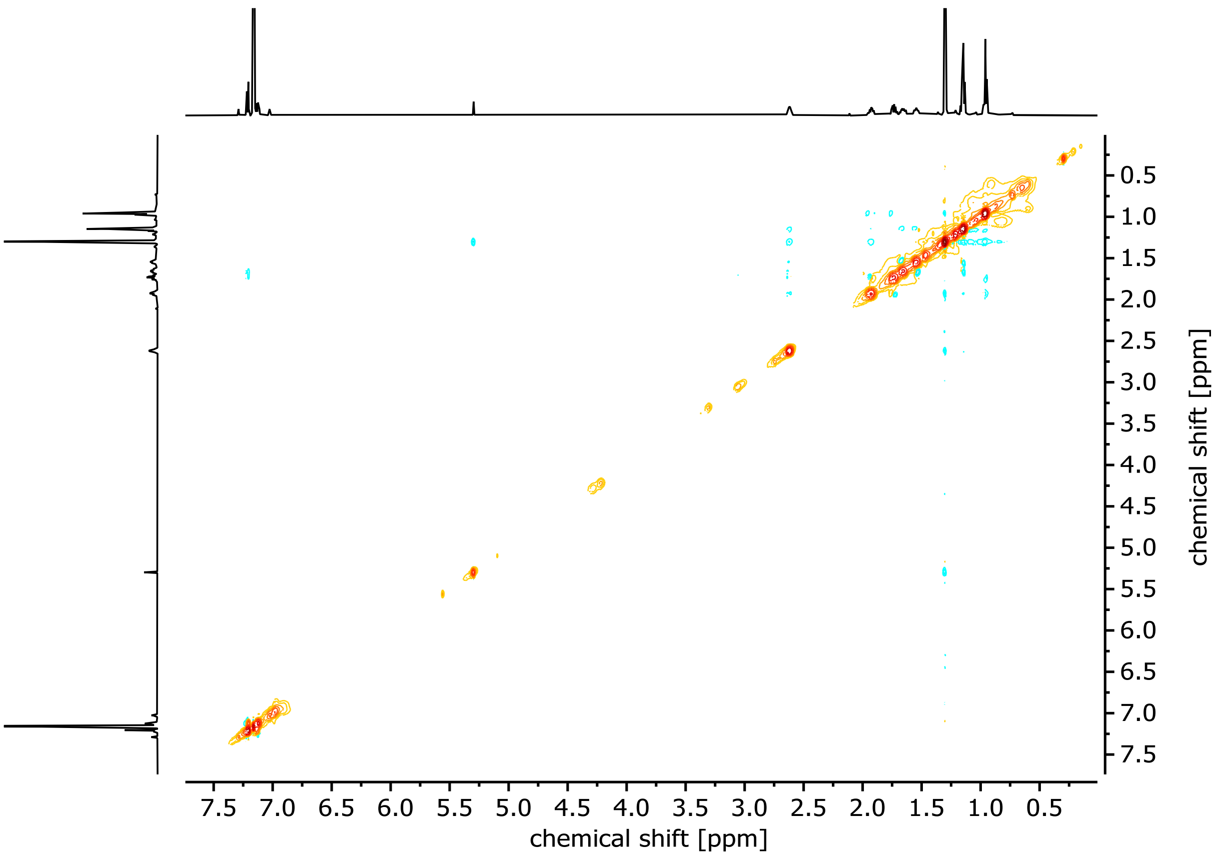

Figure S22. ^{1}H-^{1}H NOESY (400.13 MHz, 298 K, C_6D_6) of [(^{DIPeP}BDI*)Ba(μ-I)]$_2$.

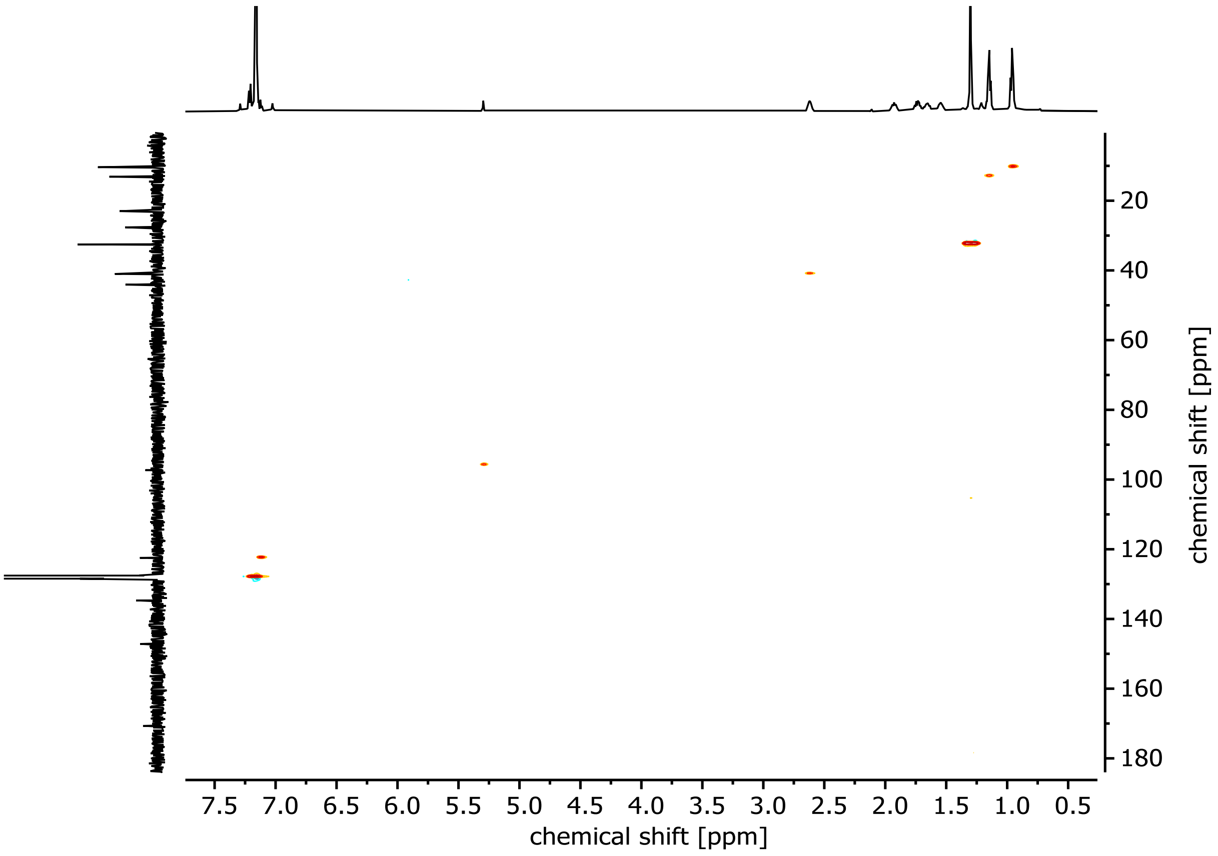

Figure S23. ^{1}H-^{13}C HSQC NMR (600.13/150.92 MHz, 298 K, C_6D_6) of [(^{DIPeP}BDI*)Ba(μ-I)]$_2$.

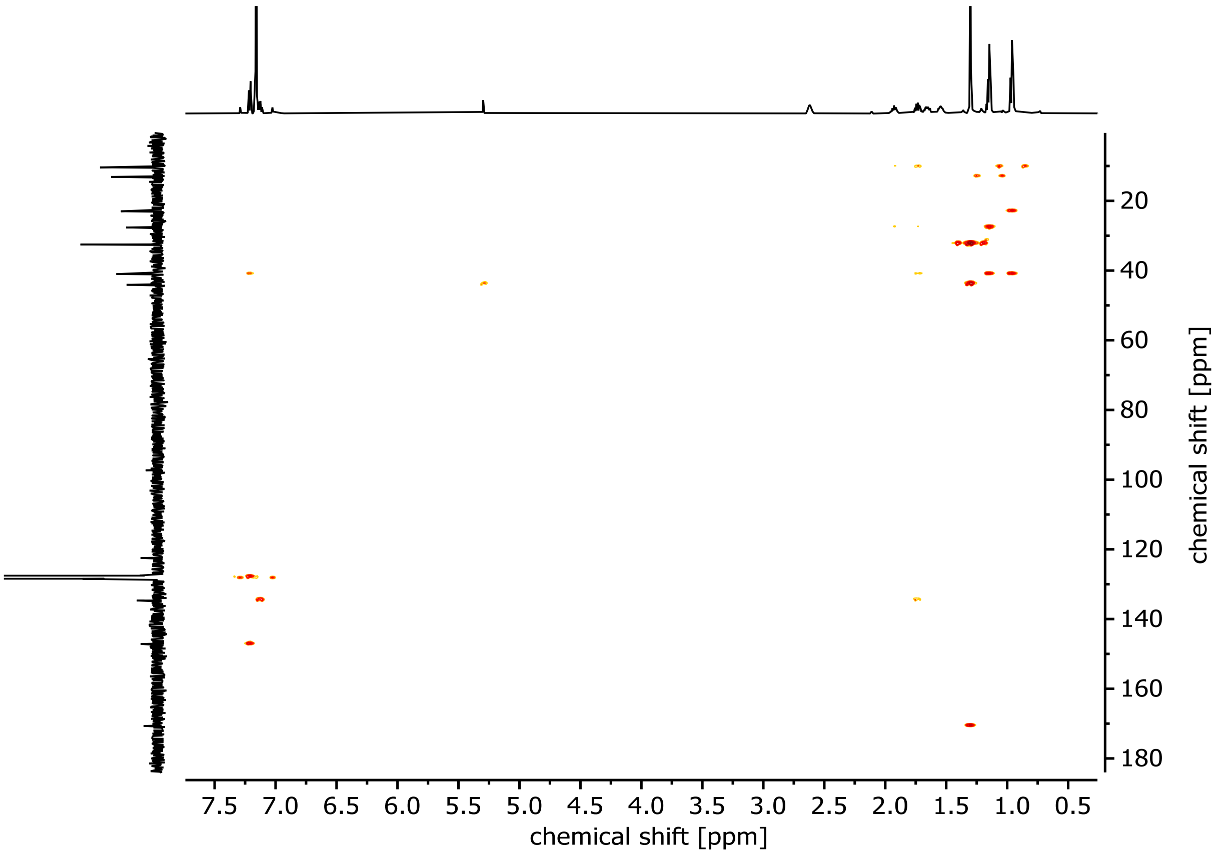

Figure S24. ^{1}H-^{13}C HMBC NMR (600.13/150.92 MHz, 298 K, C_6D_6) of [(^{DIPeP}BDI*)Ba(μ-I)]$_2$.

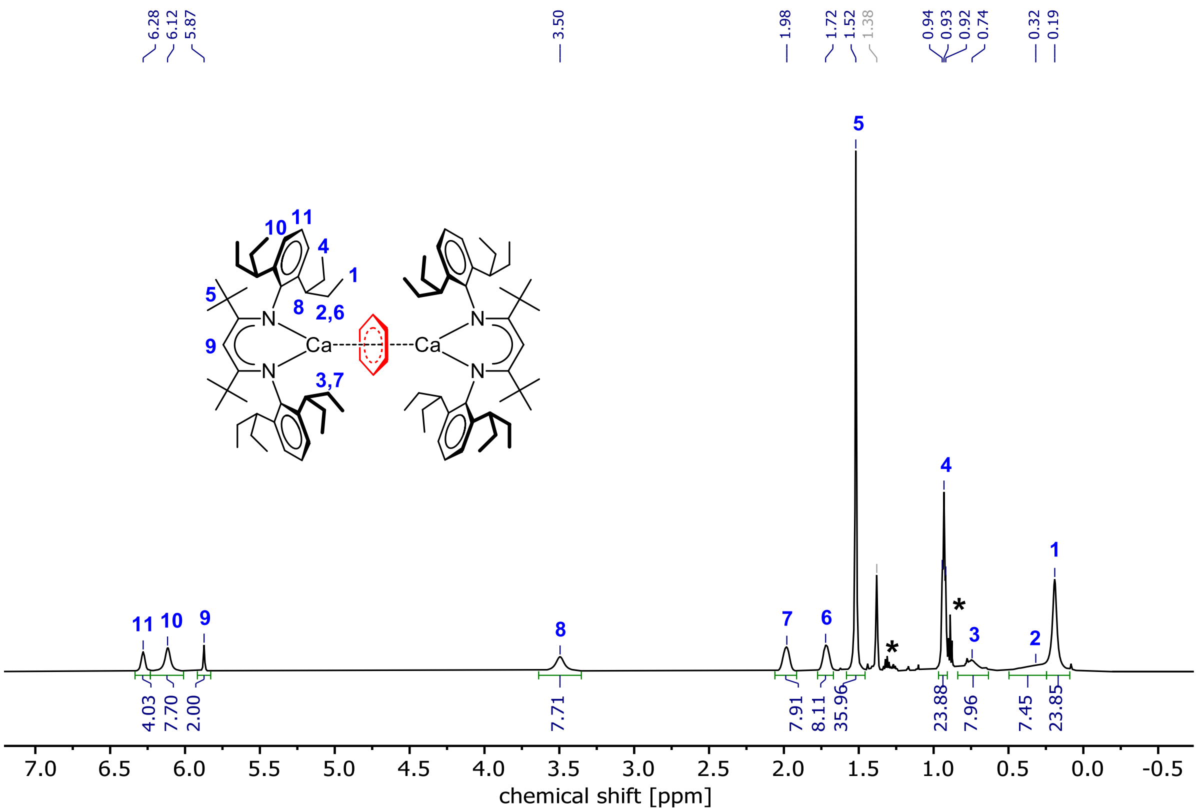

Figure S25. ^{1}H NMR (600.13 MHz, 298 K, C_6D_{12}) of [{(^{DIPeP}BDI*)Ca}$_2$(η^6:η^6-C_6H_6)]. * denotes co-crystallised *n*-pentane. The $C_6H_6^{2-}$ anion is NMR silent.

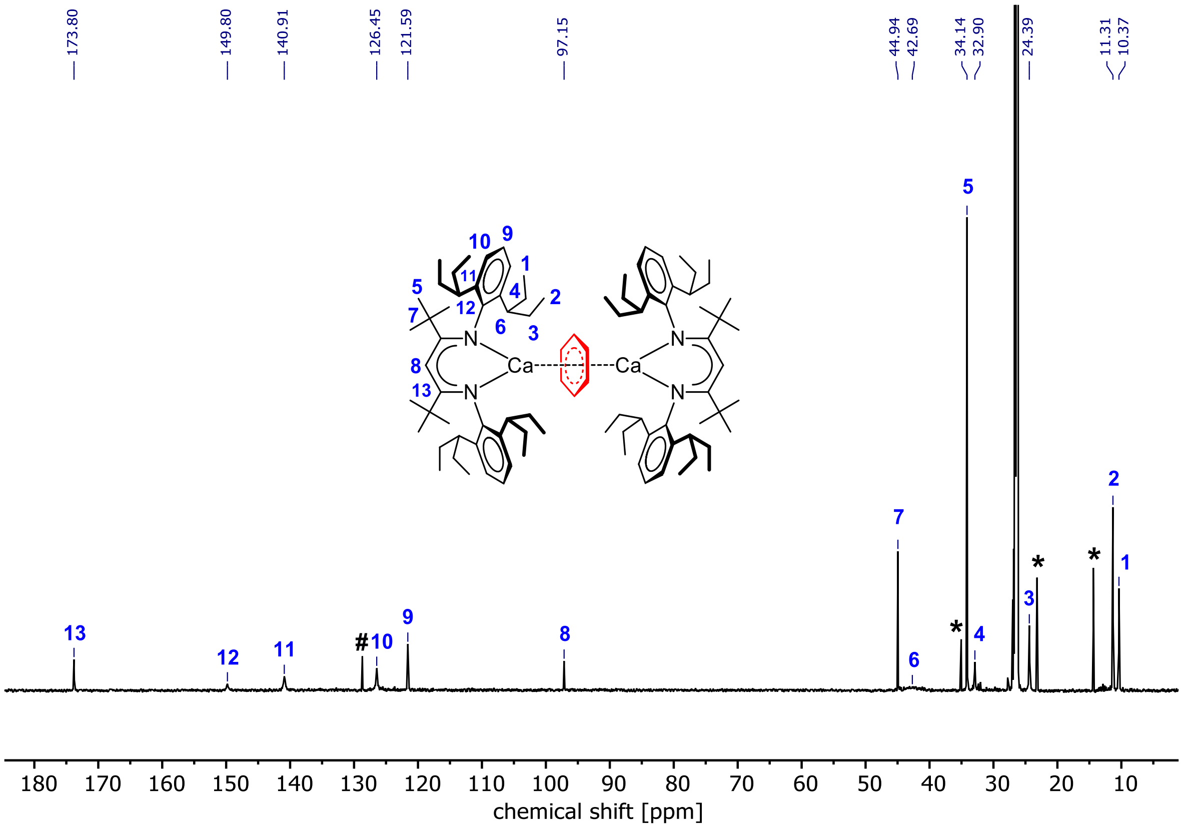

Figure S26. ^{13}C NMR (600.13 MHz, 298 K, C_6D_{12}) of [{(^{DIPeP}BDI*)Ca}$_2$(η^6:η^6-C_6H_6)]. * denotes co-crystallised *n*-pentane, # denotes benzene. The $C_6H_6^{2-}$ anion is NMR silent.

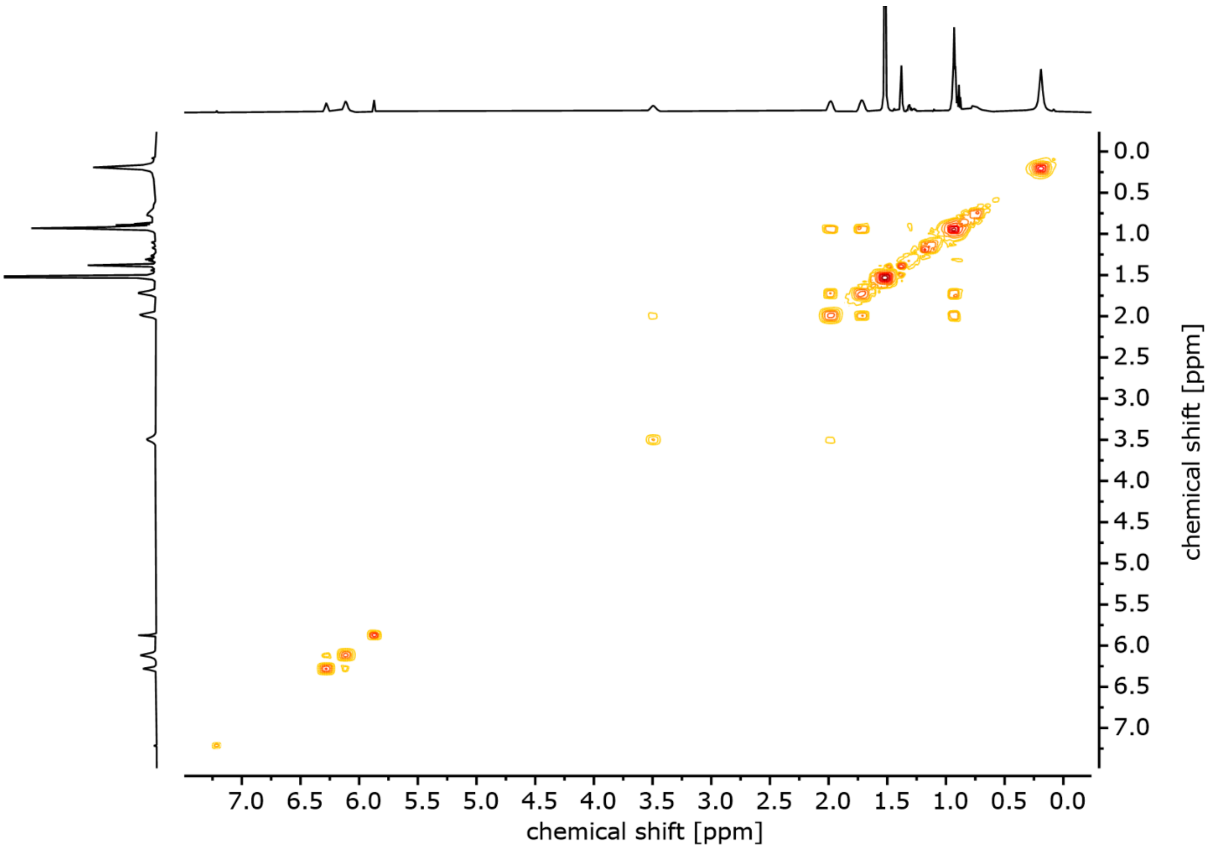

Figure S27. ^{1}H-^{1}H COSY NMR (600.13 MHz, 298 K, C_6D_{12}) of [{(^{DIPeP}BDI*)Ca}$_2$(η^6:η^6-C_6H_6)].

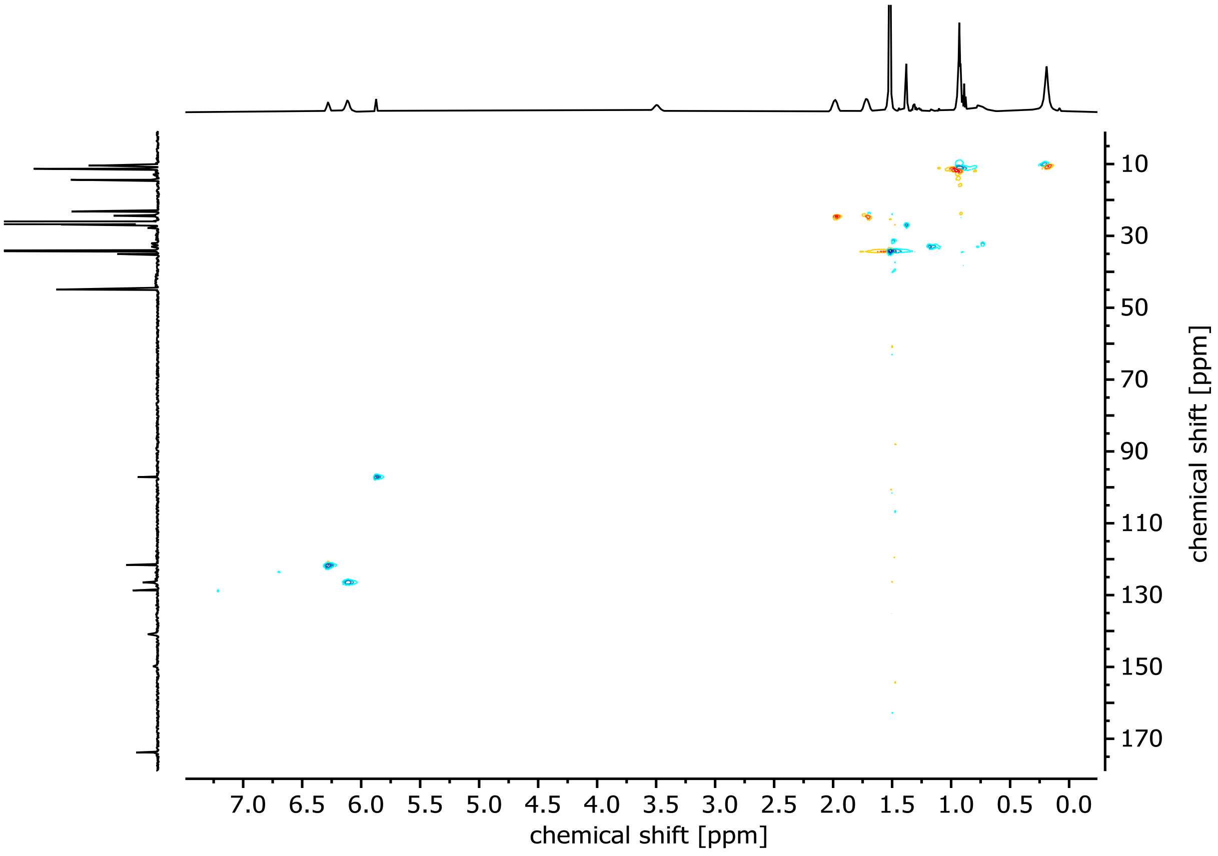

Figure S28. ^{1}H-^{13}C HSQC NMR (600.13/150.92 MHz, 298 K, C_6D_{12}) of [{(^{DIPeP}BDI*)Ca}$_2$(η^6:η^6-C_6H_6)].

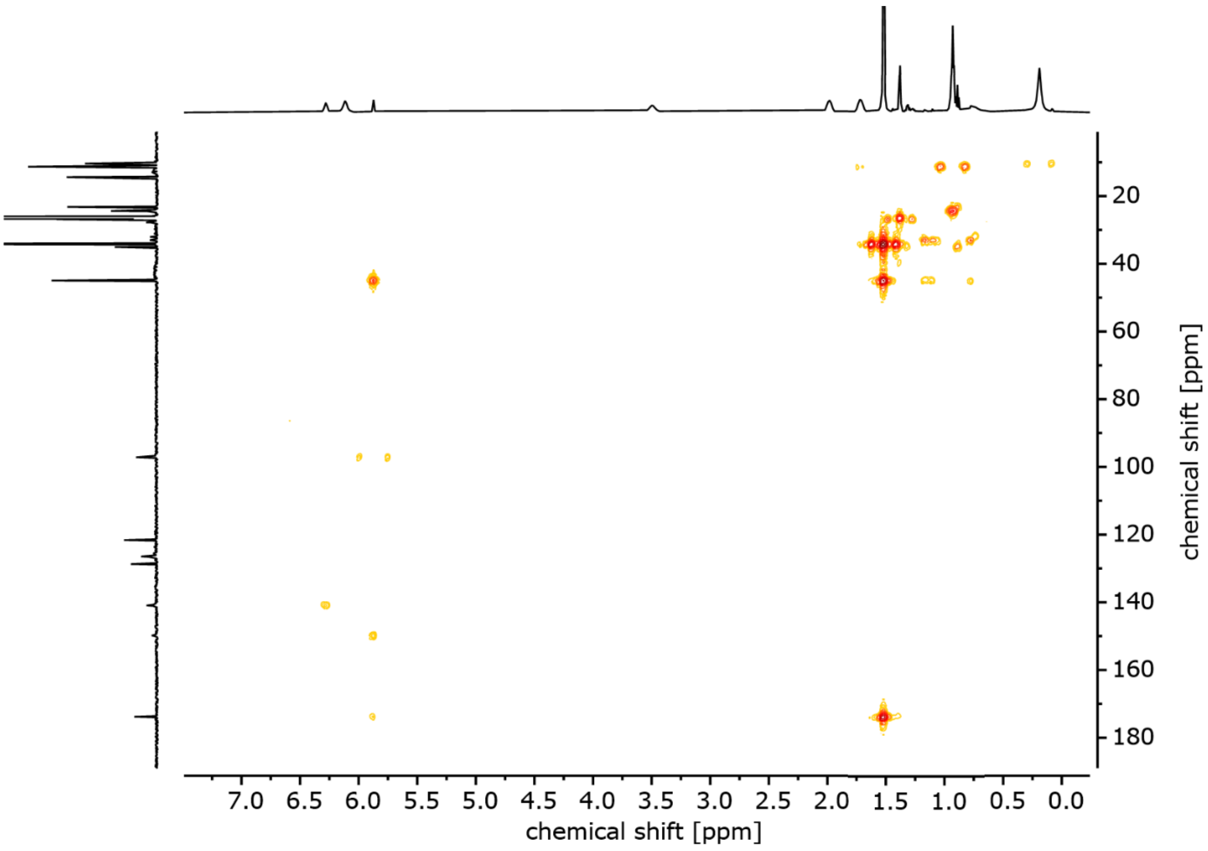

Figure S29. ^{1}H-^{13}C HMBC NMR (600.13/150.92 MHz, 298 K, C_6D_{12}) of [{(^{DIPeP}BDI*)Ca}$_2$(η^6:η^6-C_6H_6)].

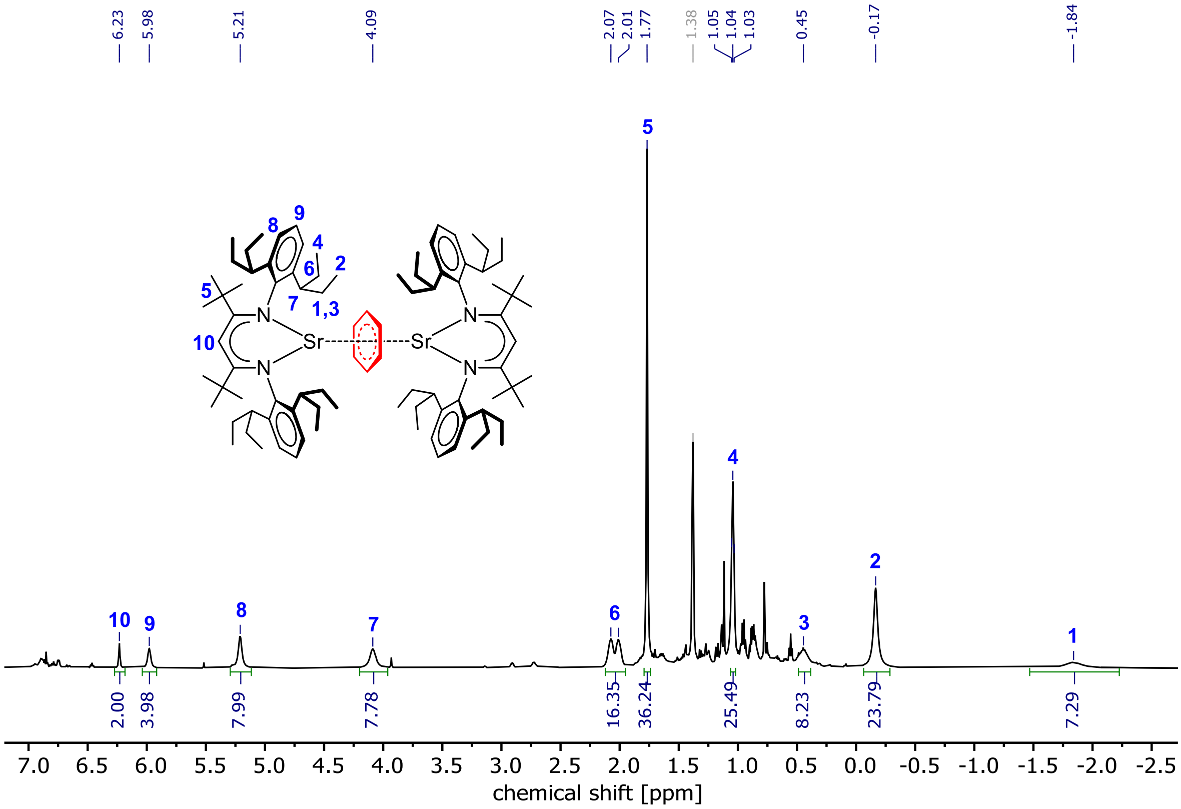

Figure S30. ^{1}H NMR (600.13 MHz, 298 K, C_6D_{12}) of [{(^{DIPeP}BDI*)Sr}$_2$(η^6:η^6-C_6H_6)]. The $C_6H_6^{2-}$ anion is NMR silent.

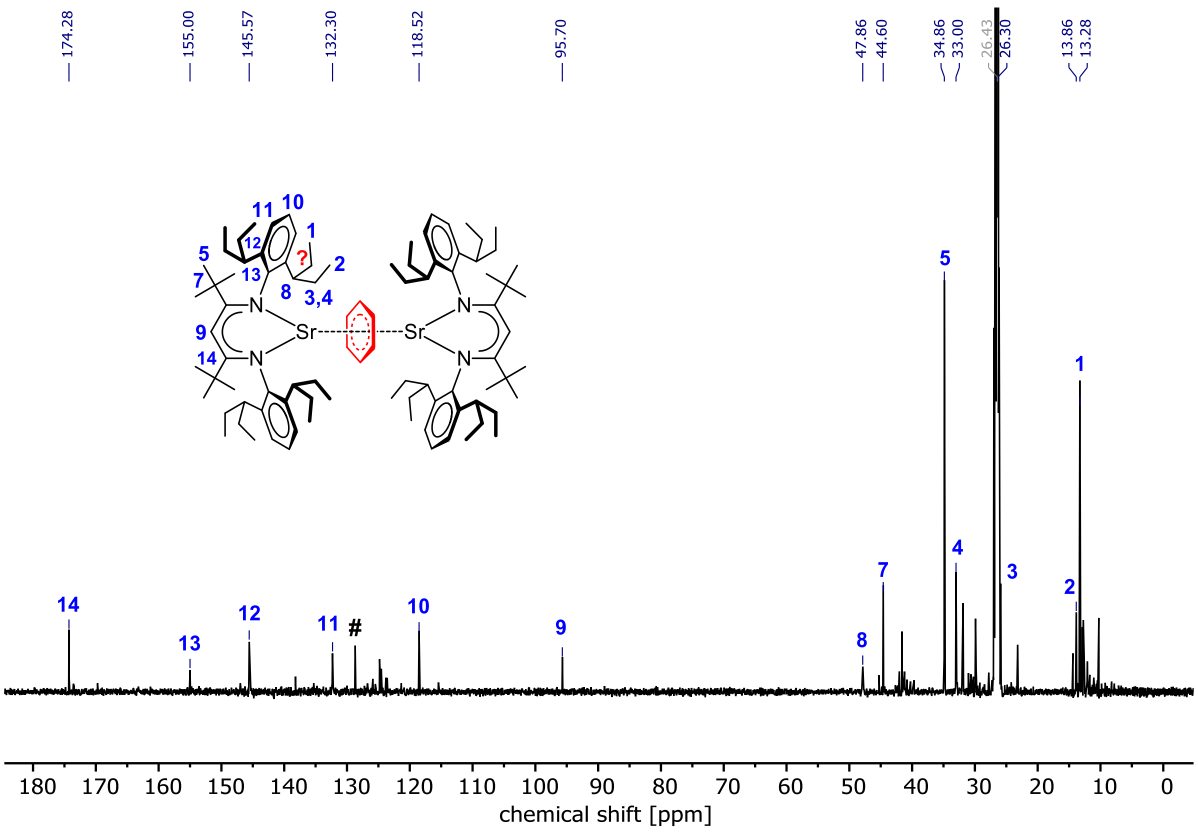

Figure S31. ^{13}C NMR (150.92 MHz, 298 K, C_6D_{12}) of [{(^{DIPeP}BDI*)Sr}$_2$(η^6:η^6-C_6H_6)]. # denotes benzene. The $C_6H_6^{2-}$ anion is NMR silent.

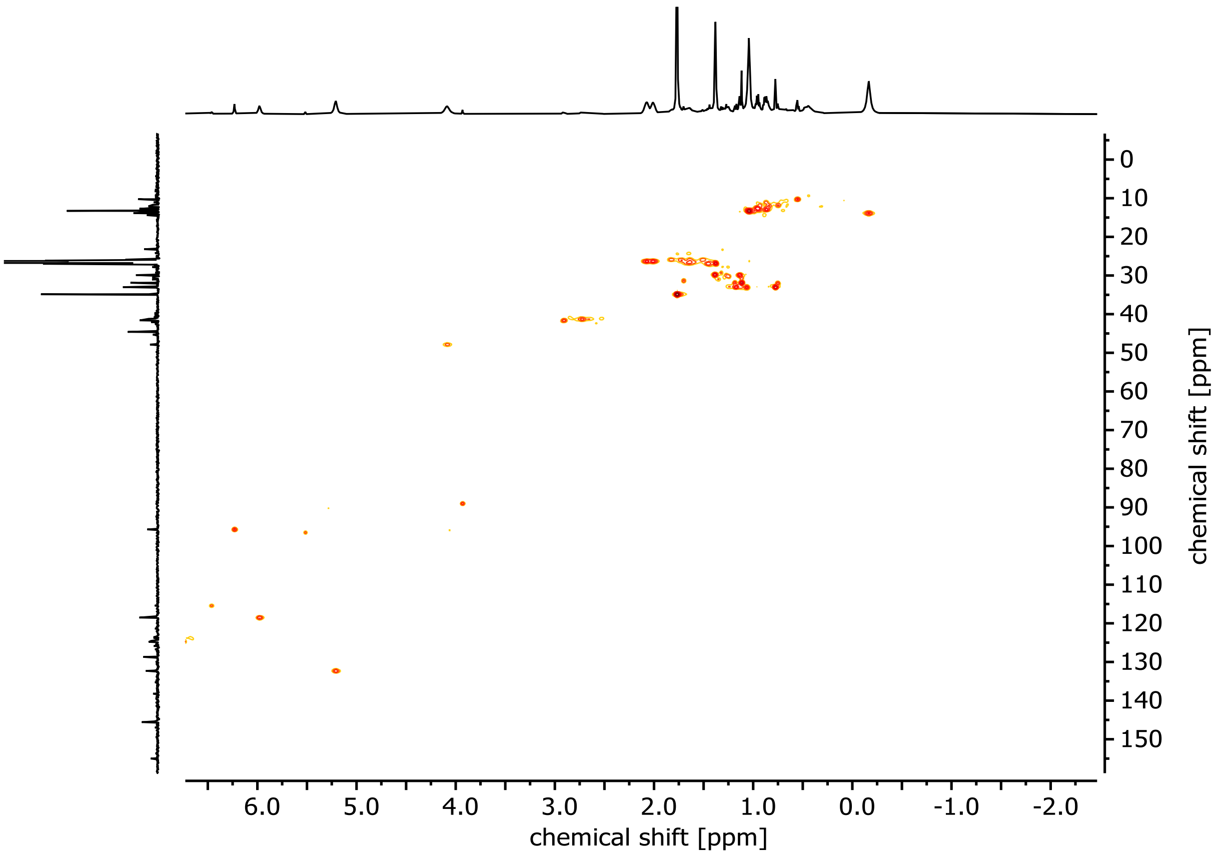

Figure S32. ^{1}H-^{13}C HSQC NMR (600.13/150.92 MHz, 298 K, C_6D_{12}) of [(^{DIPeP}BDI*)Sr]$_2$(η^6:η^6-C_6H_6)].

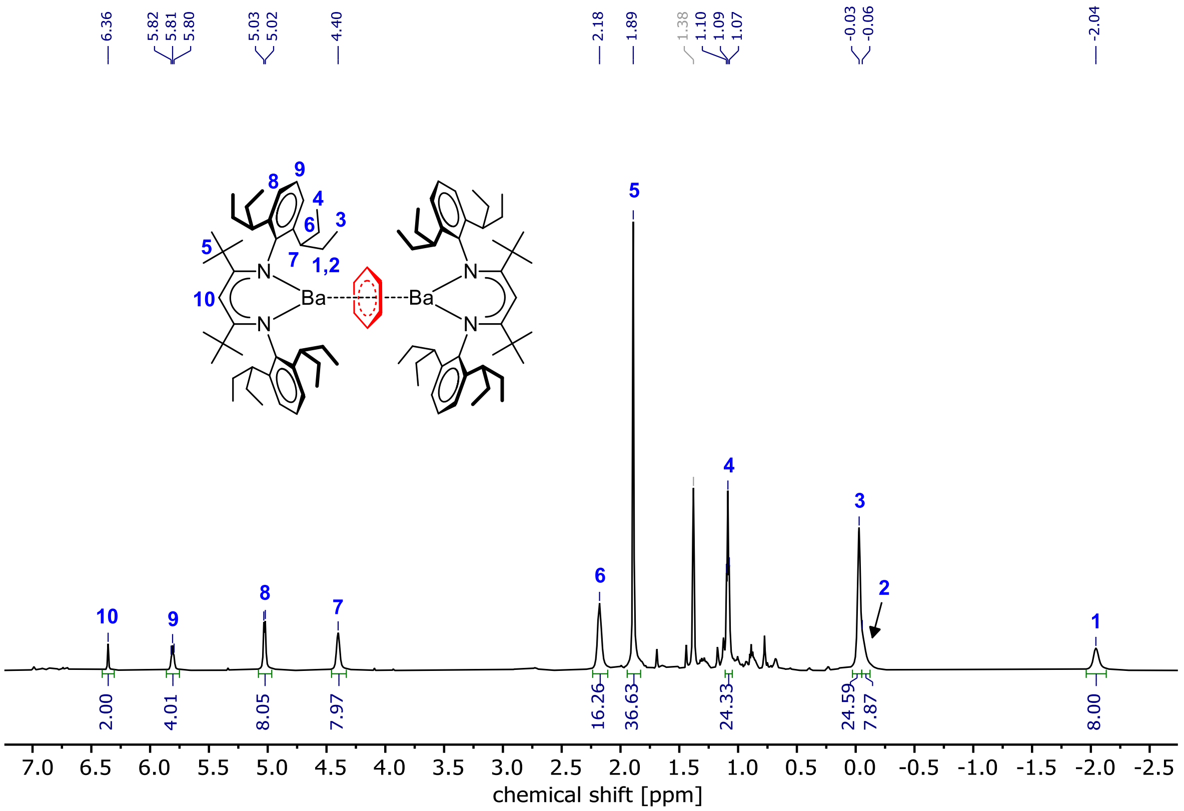

Figure 33. ^{1}H NMR (600.13 MHz, 298 K, C_6D_{12}) of [{(^{DIPeP}BDI*)Ba}$_2$(η^6:η^6-C_6H_6)]. The $C_6H_6^{2-}$ anion is NMR silent.

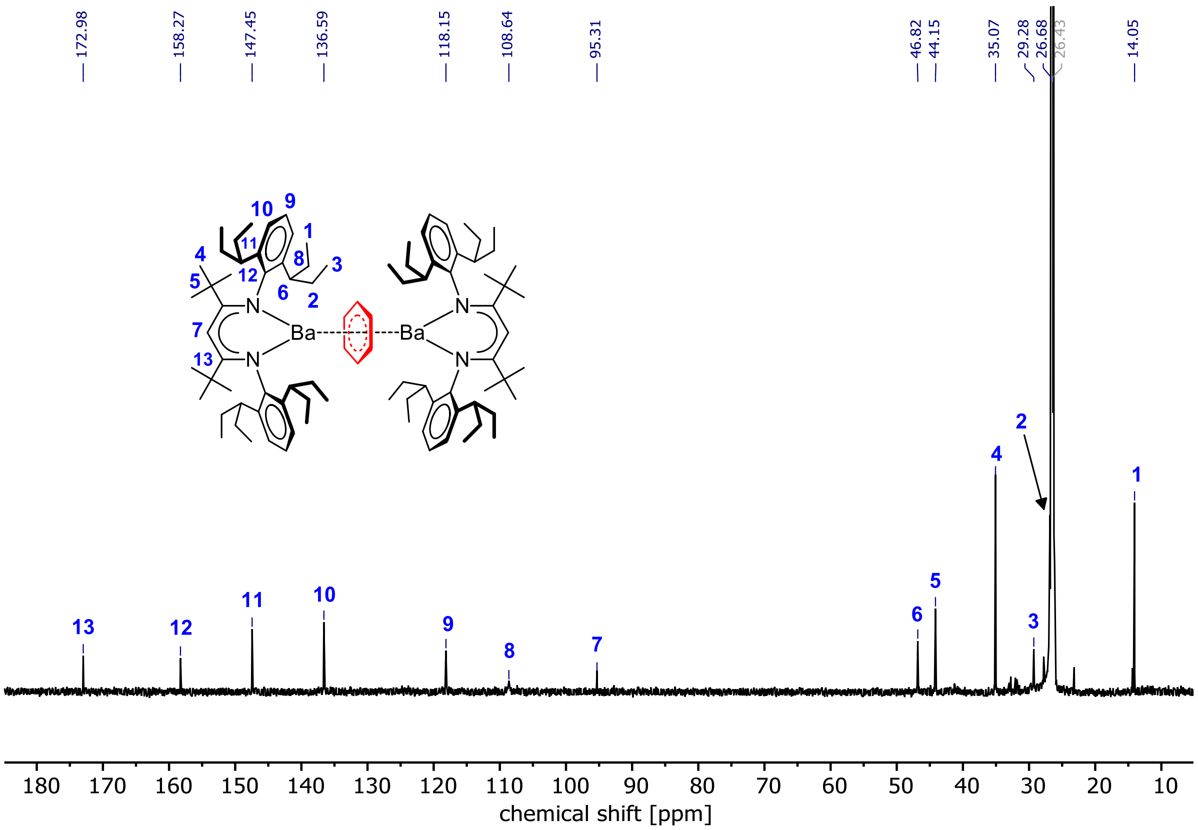

Figure S34. ^{13}C NMR (150.92 MHz, 298 K, C_6D_{12}) of [{(^{DIPeP}BDI*)Ba}$_2$(η^6:η^6-C_6H_6)]. The $C_6H_6^{2-}$ anion is NMR silent.

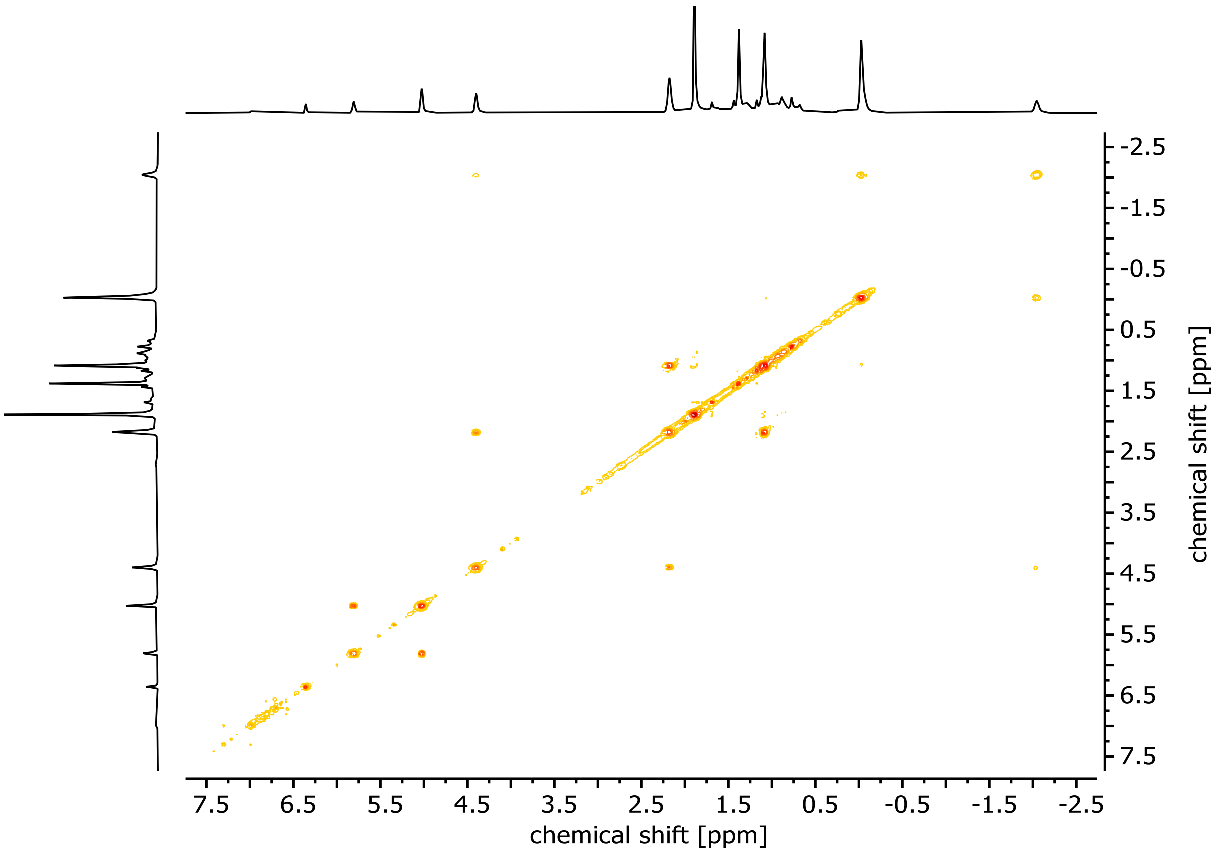

Figure S35. ^{1}H-^{1}H COSY NMR (600.13 MHz, 298 K, C_6D_{12}) of [{(^{DIPeP}BDI*)Ba}$_2$(η^6:η^6-C_6H_6)].

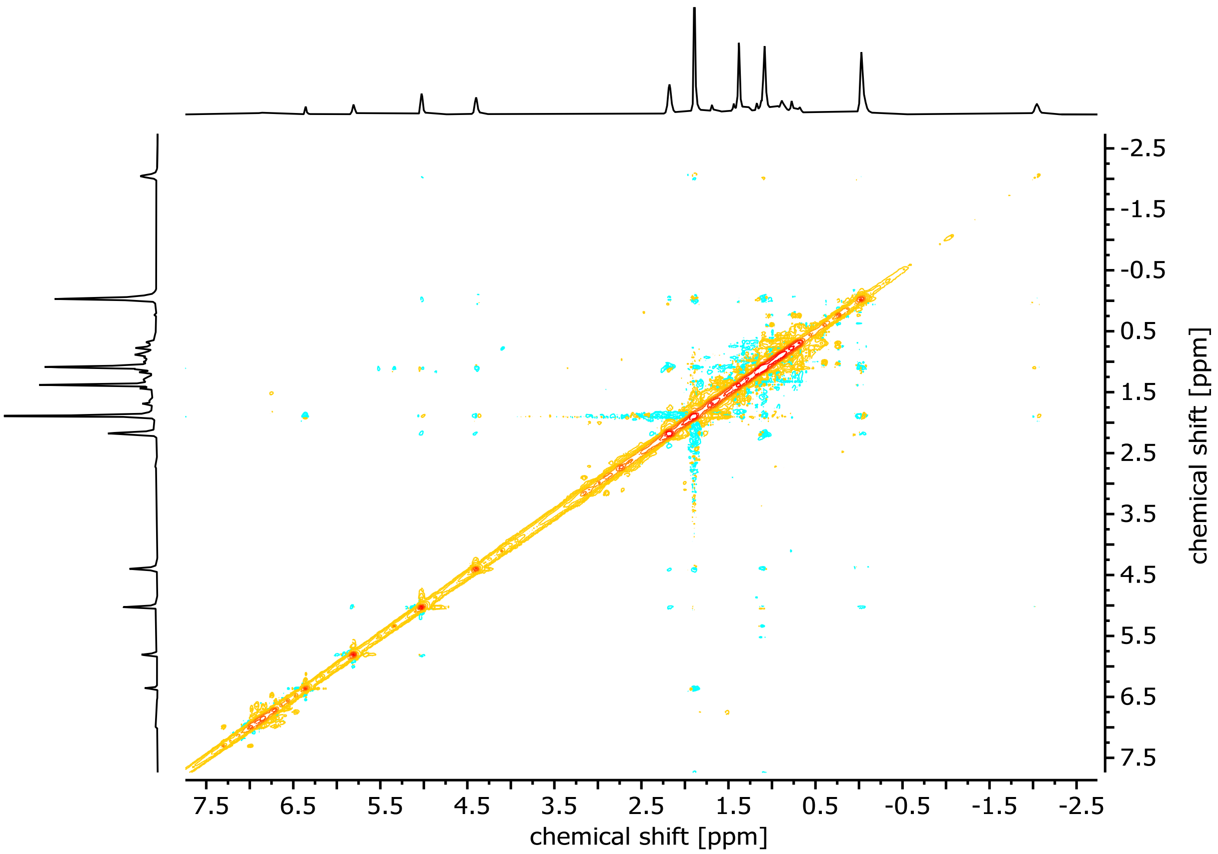

Figure S36. ^{1}H-^{1}H NOESY (400.13 MHz, 298 K, C_6D_{12}) of [{(^{DIPeP}BDI*)Ba}$_2$(η^6:η^6-C_6H_6)].

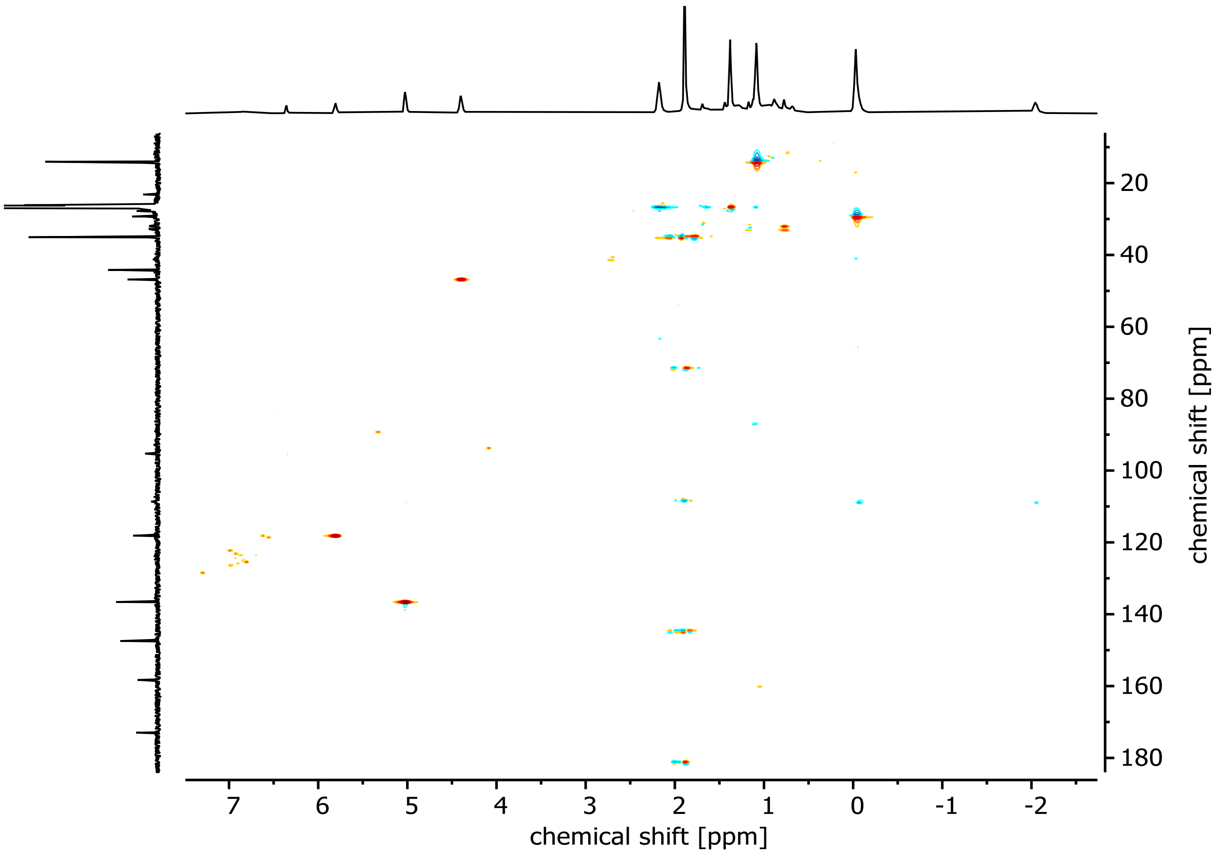

Figure S37. ^{1}H-^{13}C HSQC NMR (600.13/150.92 MHz, 298 K, C_6D_{12}) of [{(^{DIPeP}BDI*)Ba}$_2$(η^6:η^6-C_6H_6)].

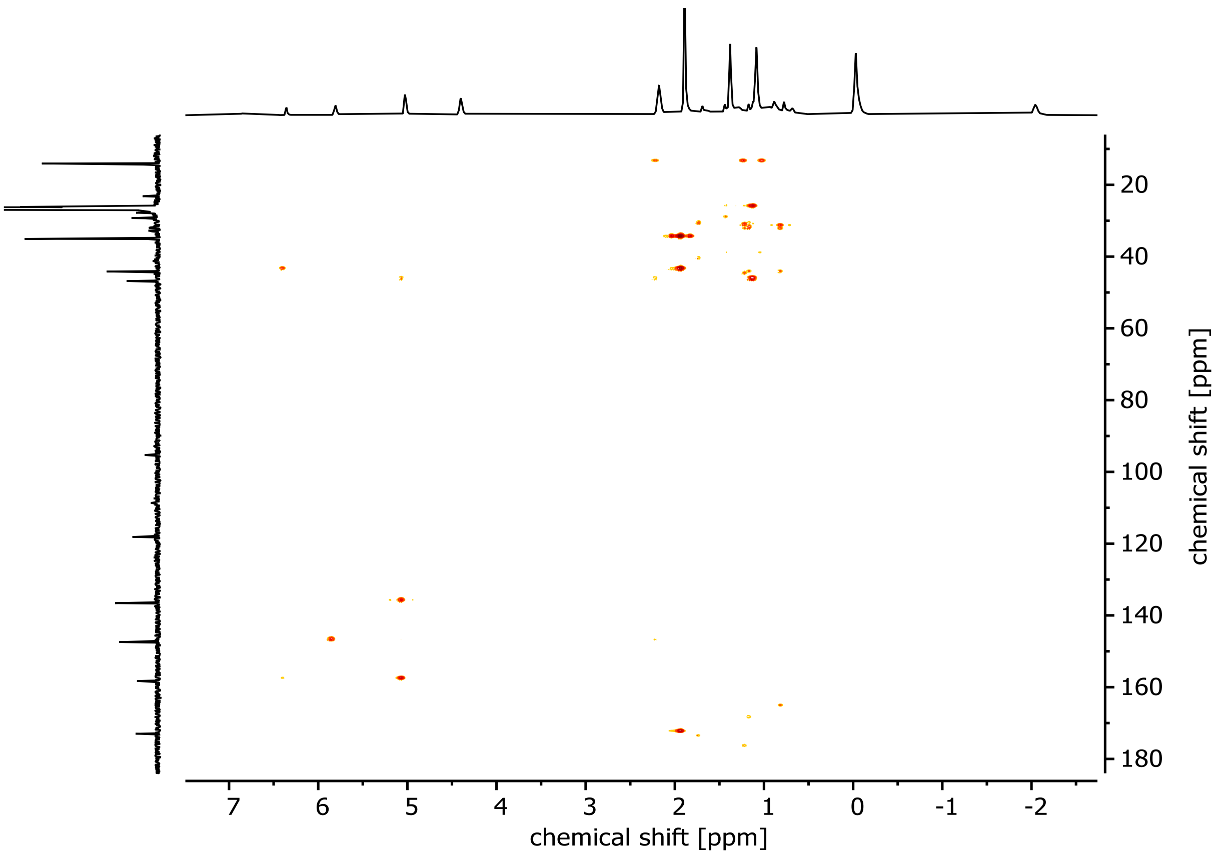

Figure S38. ^{1}H-^{13}C HMBC NMR (600.13/150.92 MHz, 298 K, C_6D_{12}) of [{(^{DIPeP}BDI*)Ba}$_2$(η^6:η^6-C_6H_6)].

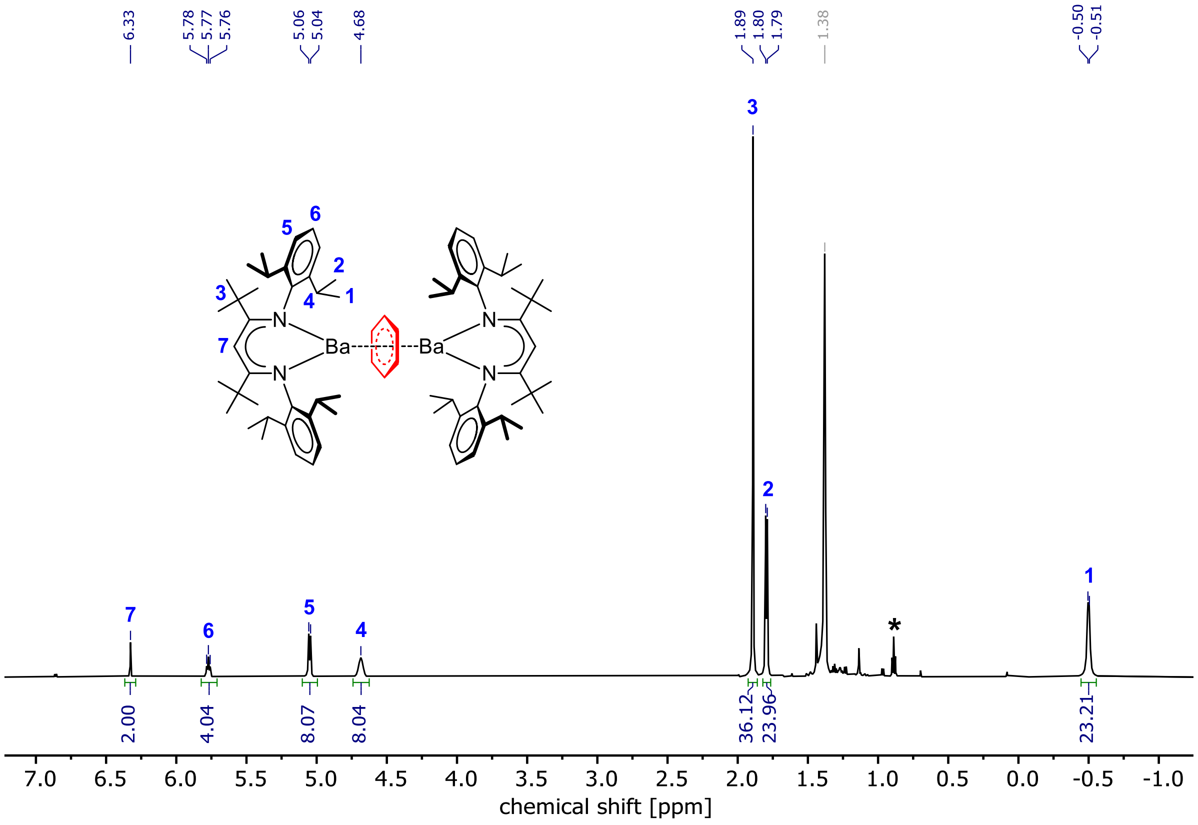

Figure S39. ^{1}H NMR (600.13 MHz, 298 K, C_6D_{12}) of [{(^{DIPP}BDI*)Ba}$_2$(η^6:η^6-C_6H_6)].* denotes co-crystallised *n*-pentane. The $C_6H_6^{2-}$ anion is NMR silent.

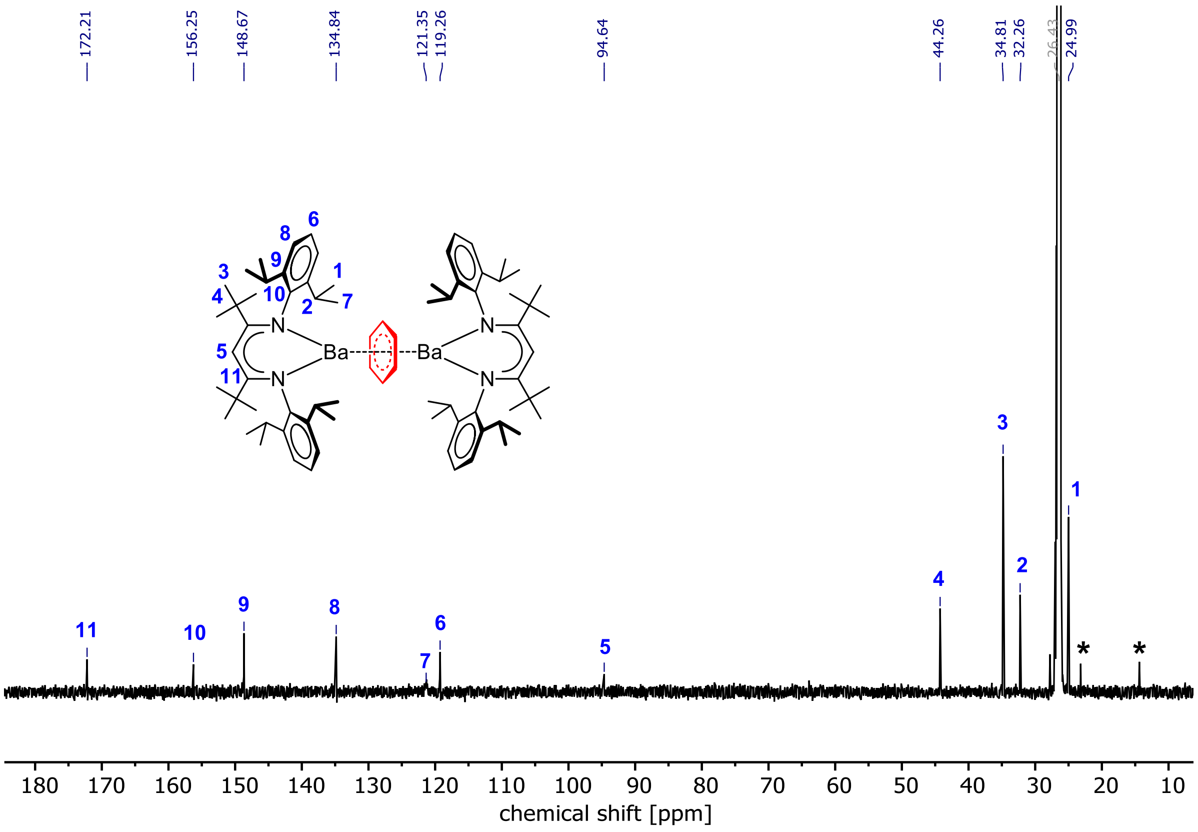

Figure S40. ^{13}C NMR (150.92 MHz, 298 K, C_6D_{12}) of [{(^{DIPP}BDI*)Ba}$_2$(η^6:η^6-C_6H_6)].* denotes co-crystallised *n*-pentane. The $C_6H_6^{2-}$ anion is NMR silent.

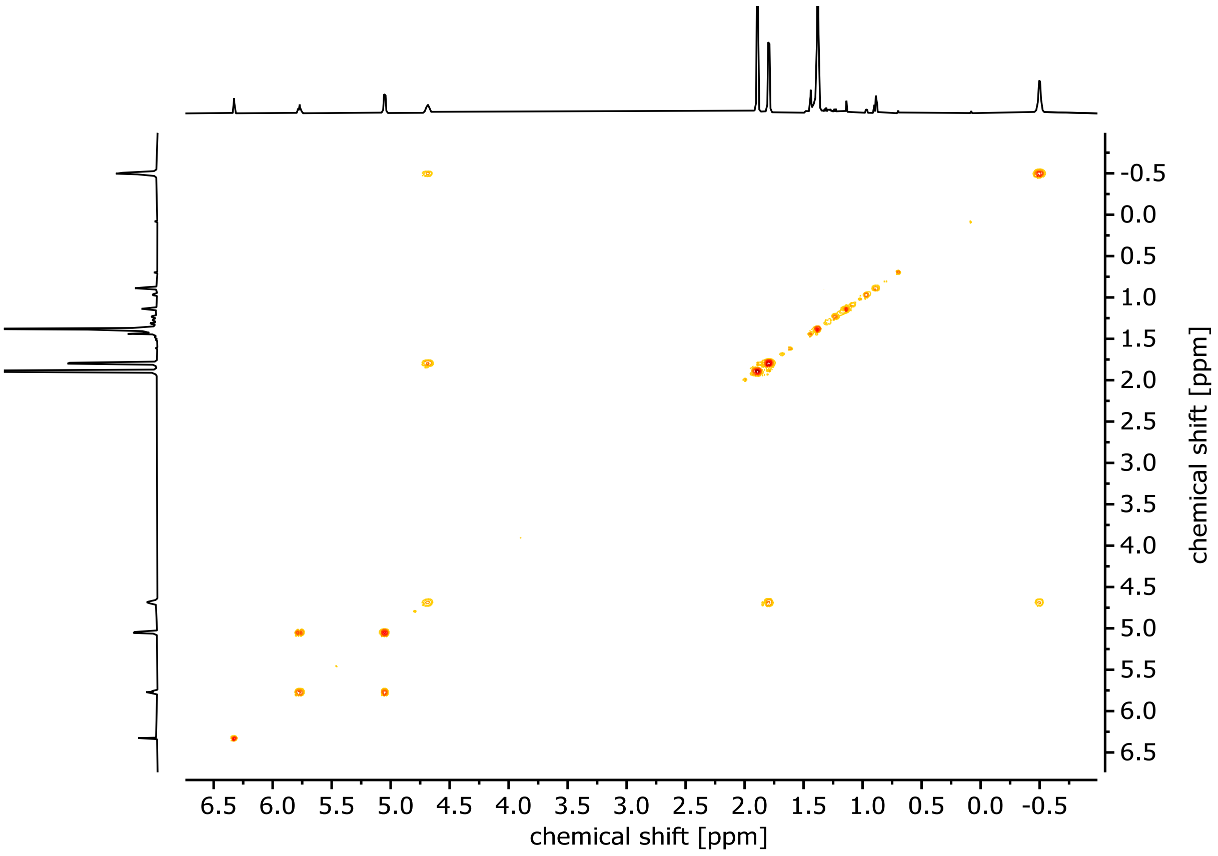

Figure S41. ^{1}H-^{1}H COSY NMR (600.13 MHz, 298 K, C_6D_{12}) of [{(^{DIPP}BDI*)Ba}$_2$(η^6-η^6-C_6H_6)].

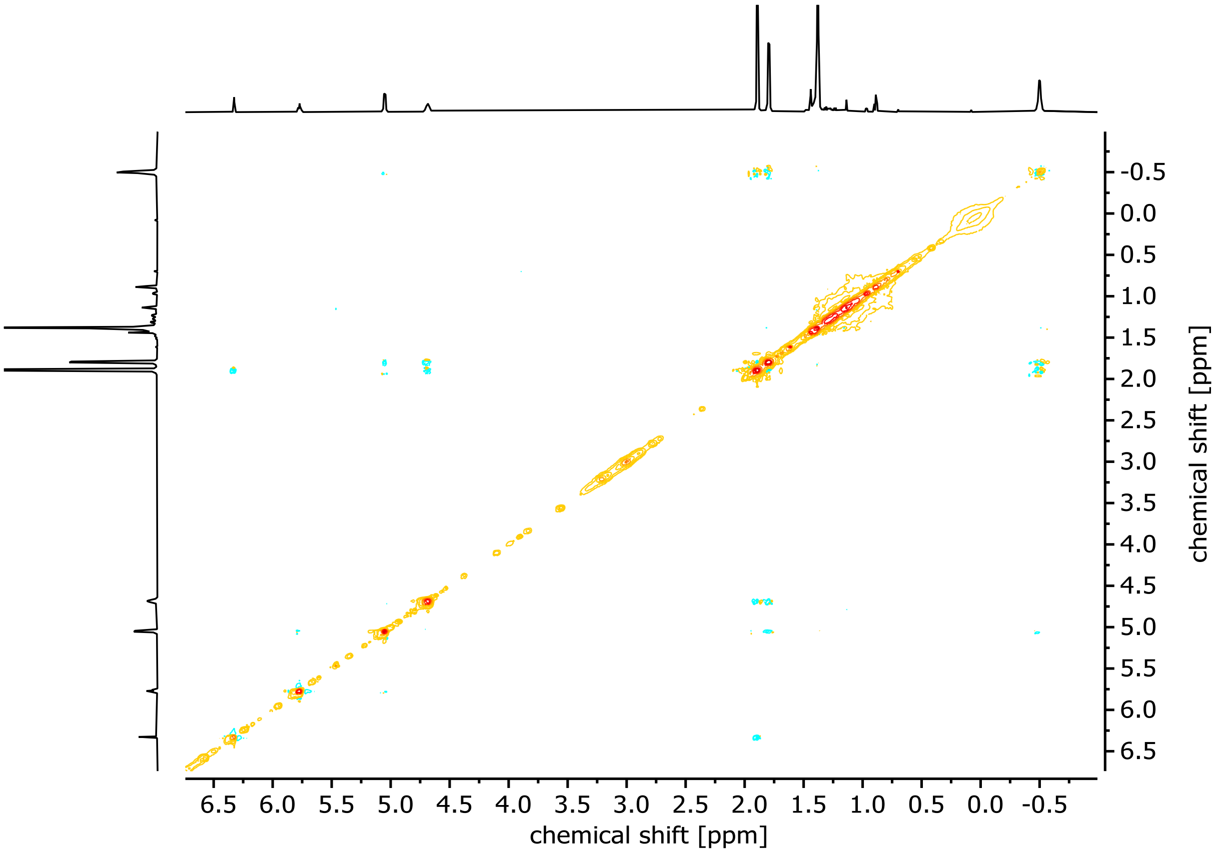

Figure S42. ^{1}H-^{1}H NOESY (400.13 MHz, 298 K, C_6D_{12}) of [{(^{DIPP}BDI*)Ba}$_2$(η^6:η^6-C_6H_6)].

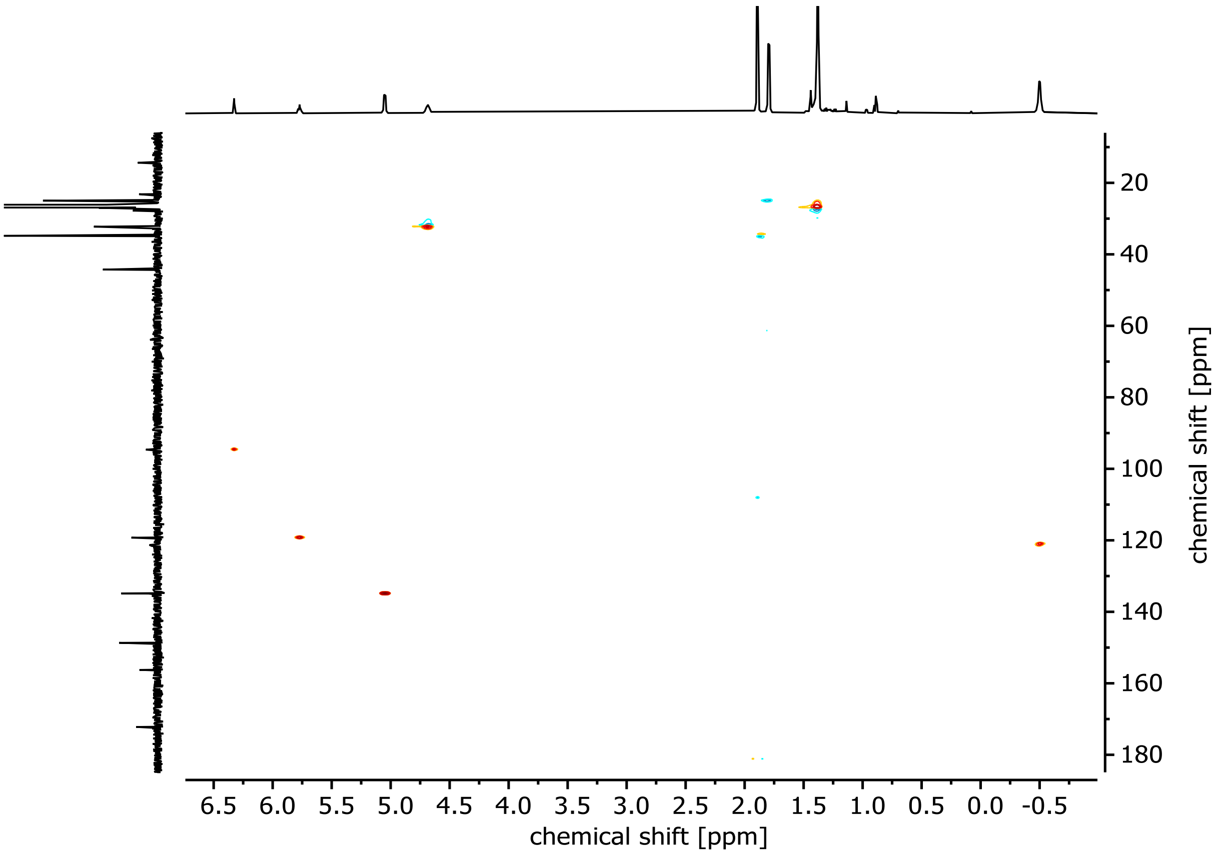

Figure S43. ^{1}H-^{13}C HSQC NMR (600.13/150.92 MHz, 298 K, C_6D_{12}) of [{(^{DIPP}BDI*)Ba}$_2$(η^6:η^6-C_6H_6)].

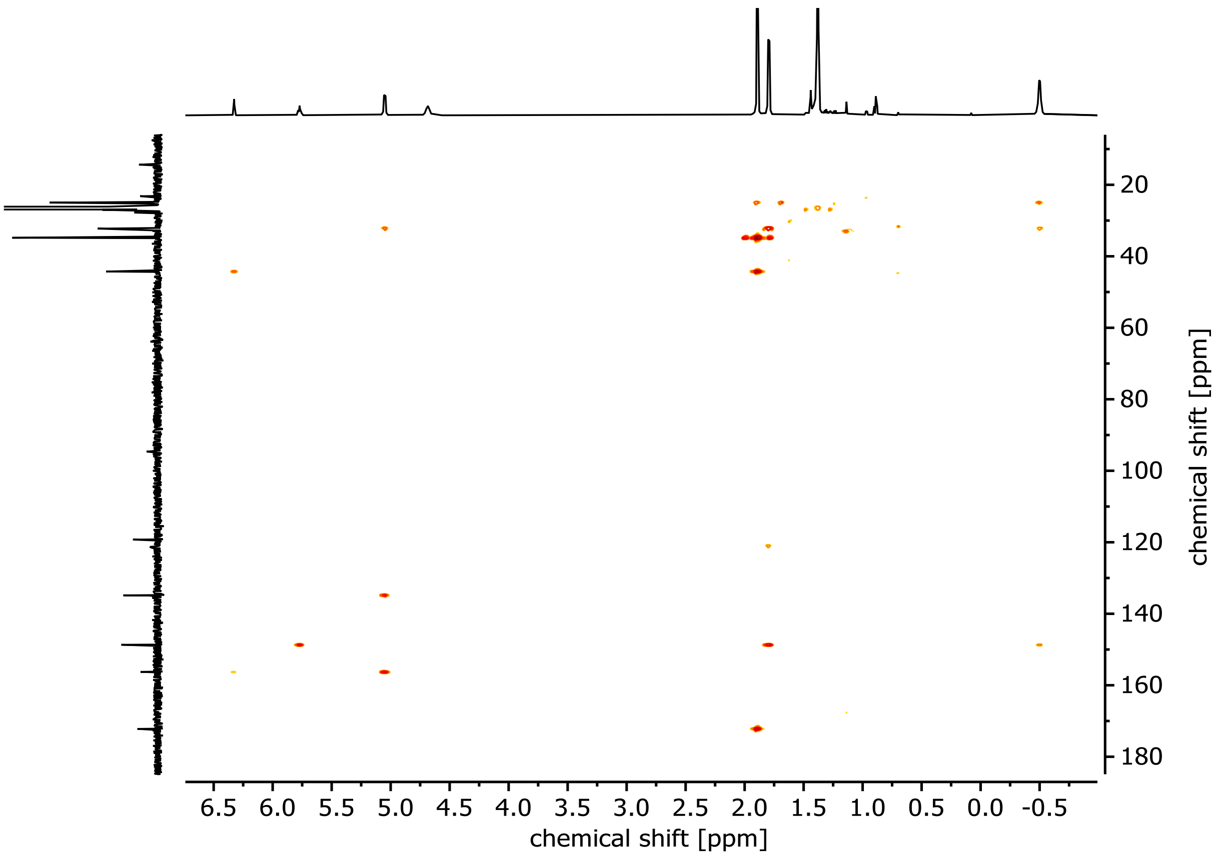

Figure S44. ^{1}H-^{13}C HMBC NMR (600.13/150.92 MHz, 298 K, C_6D_{12}) of [{(^{DIPP}BDI*)Ba}$_2$(η^6:η^6-C_6H_6)].

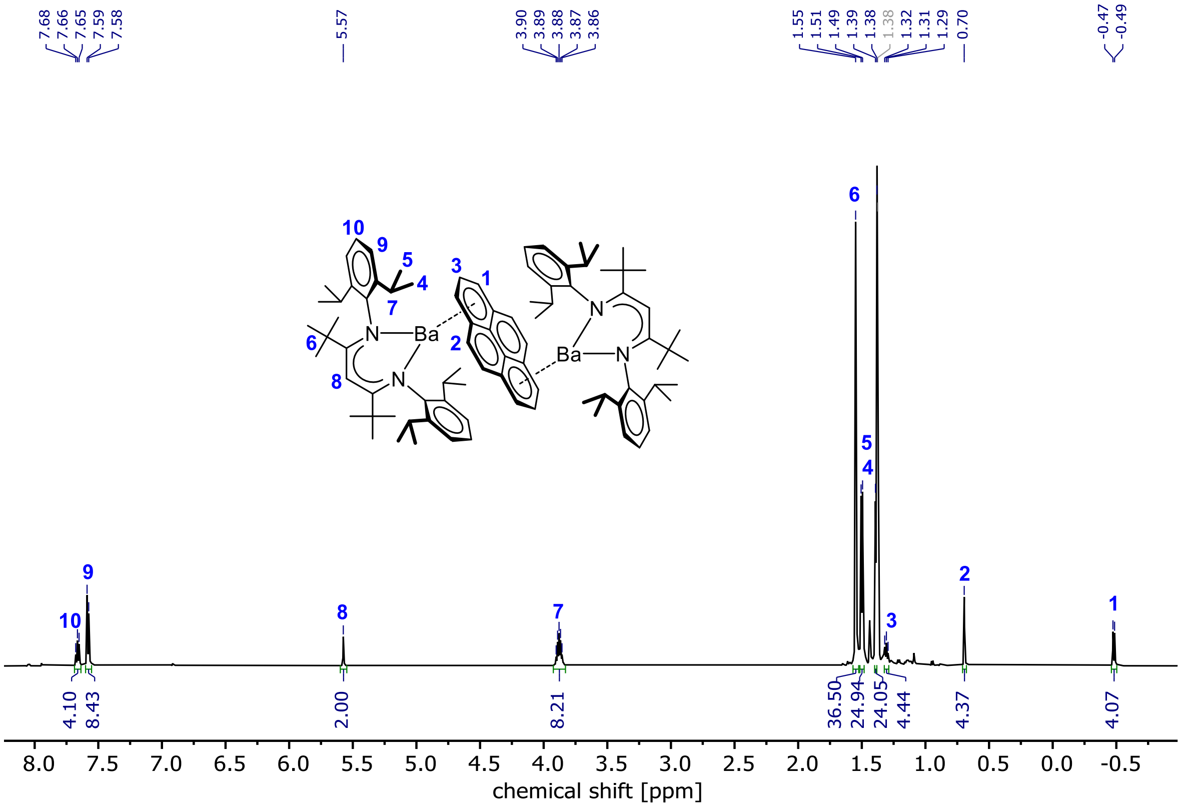

Figure S45. ^{1}H NMR (600.13 MHz, 298 K, C_6D_{12}) of [{(^{DIPP}BDI*)Ba}$_2$(η^6:η^6-$C_{16}H_{10}$)].

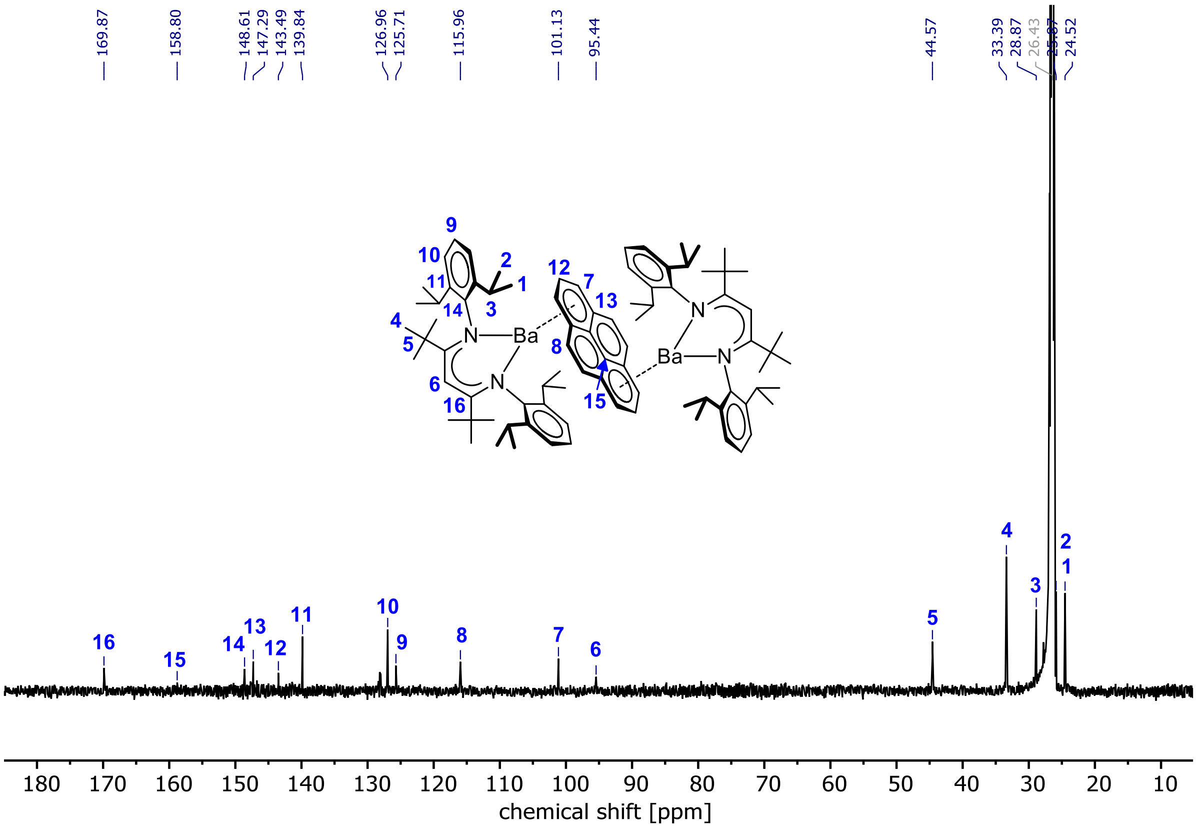

Figure S46. ^{13}C NMR (150.92 MHz, 298 K, C_6D_{12}) of [{(^{DIPP}BDI*)Ba}$_2$(η^6:η^6-$C_{16}H_{10}$)].

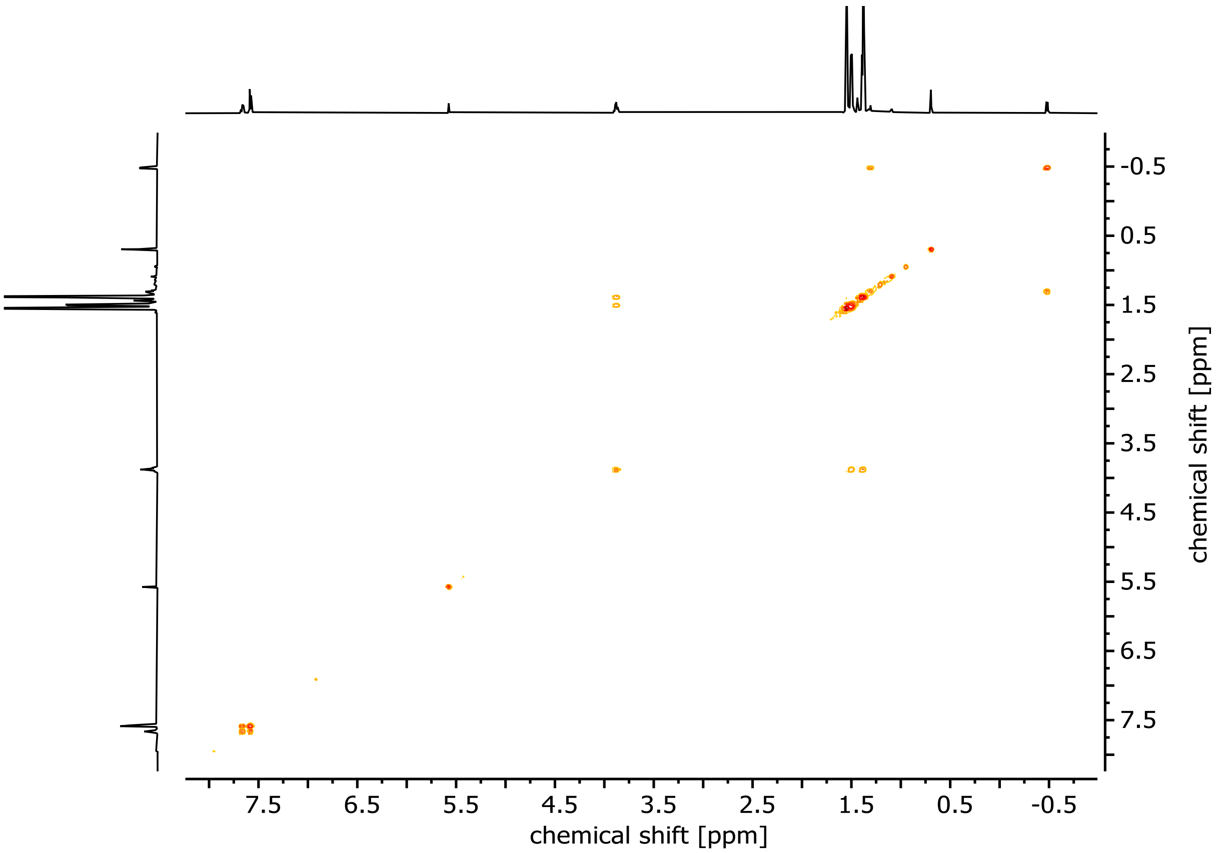

Figure S47. ^1H-^1H COSY NMR (600.13 MHz, 298 K, C_6D_{12}) of [{(^{DIPP}BDI*)Ba}$_2$(η^6:η^6-$C_{16}H_{10}$)].

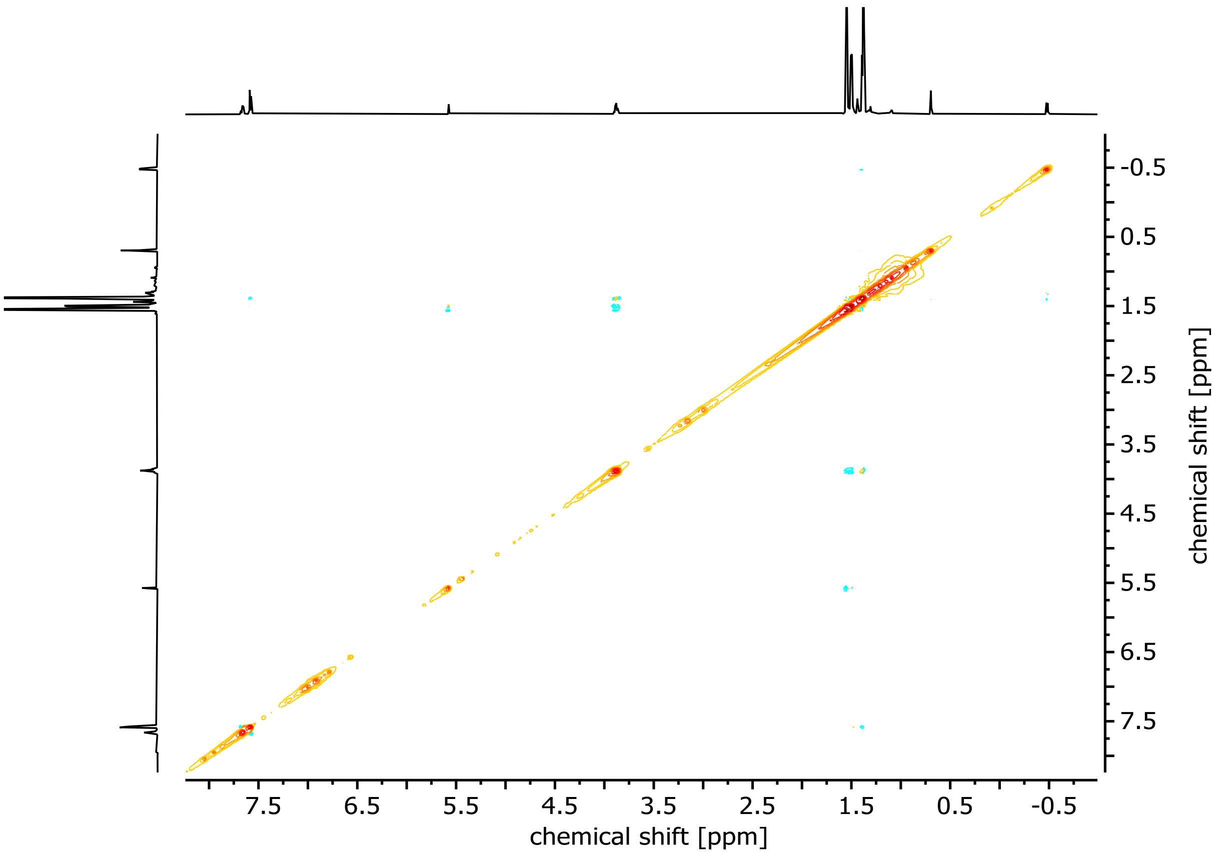

Figure S48. ^1H-^1H NOESY (400.13 MHz, 298 K, C_6D_{12}) of [{(^{DIPP}BDI*)Ba}$_2$(η^6:η^6-$C_{16}H_{10}$)].

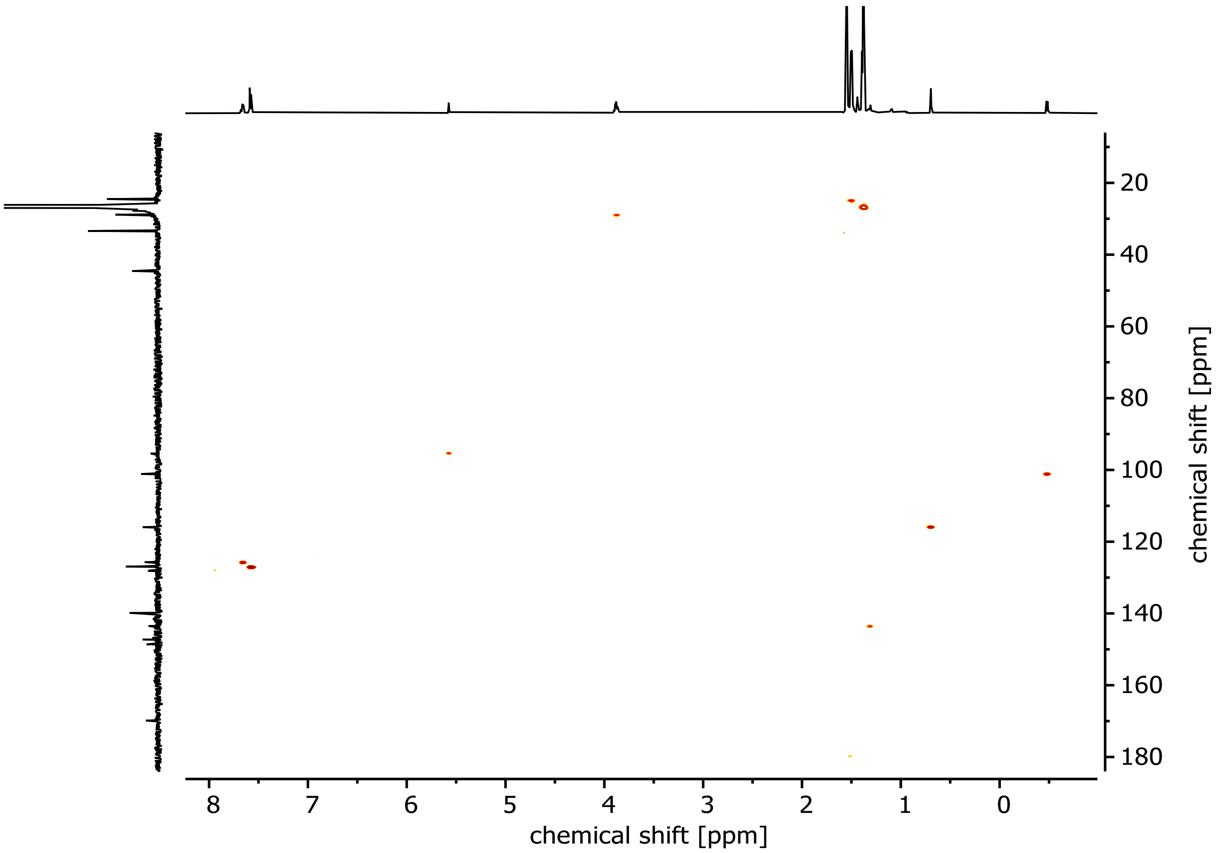

Figure S49. ^1H-^{13}C HSQC NMR (600.13/150.92 MHz, 298 K, C_6D_{12}) of [{(^{DIPP}BDI*)Ba}$_2$(η^6:η^6-$C_{16}H_{10}$)].

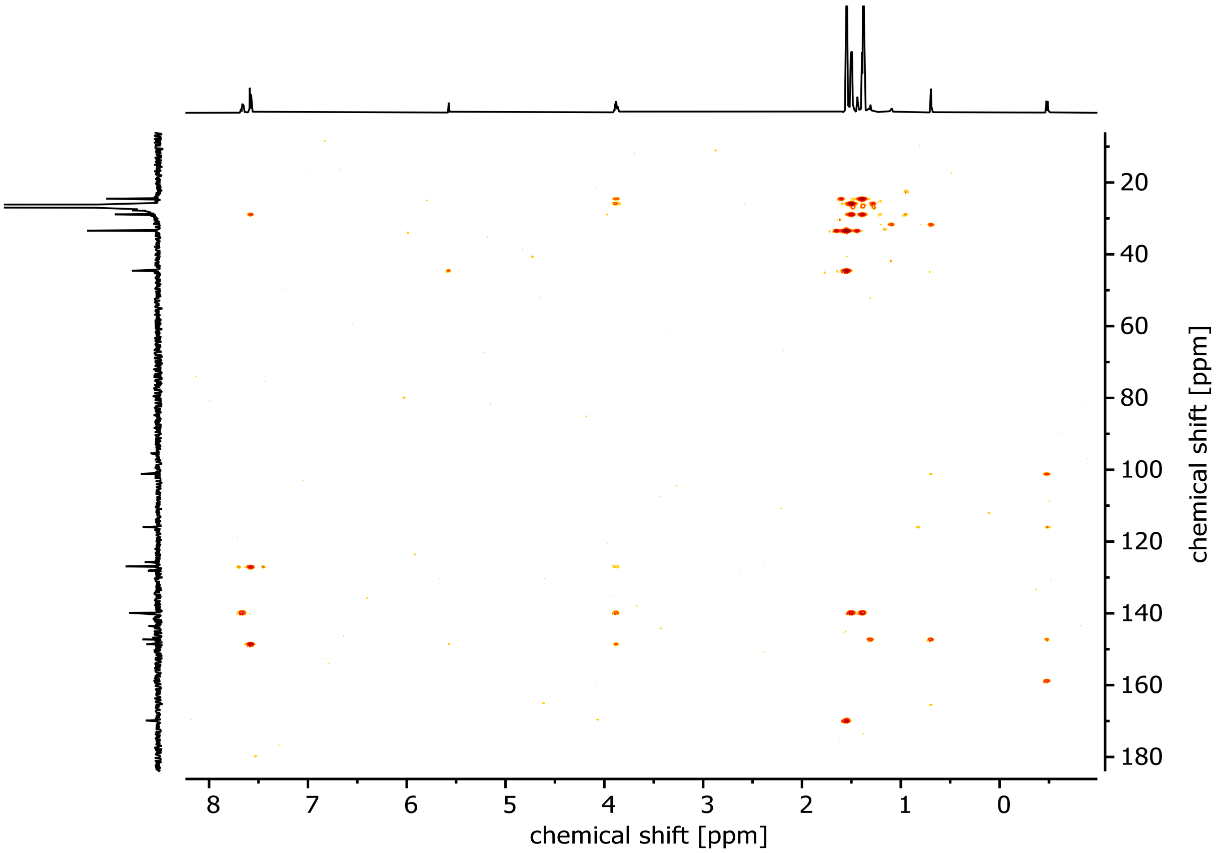

Figure 50. ^1H-^{13}C HMBC NMR (600.13/150.92 MHz, 298 K, C_6D_{12}) of [{(^{DIPP}BDI*)Ba}$_2$(η^6:η^6-$C_{16}H_{10}$)].

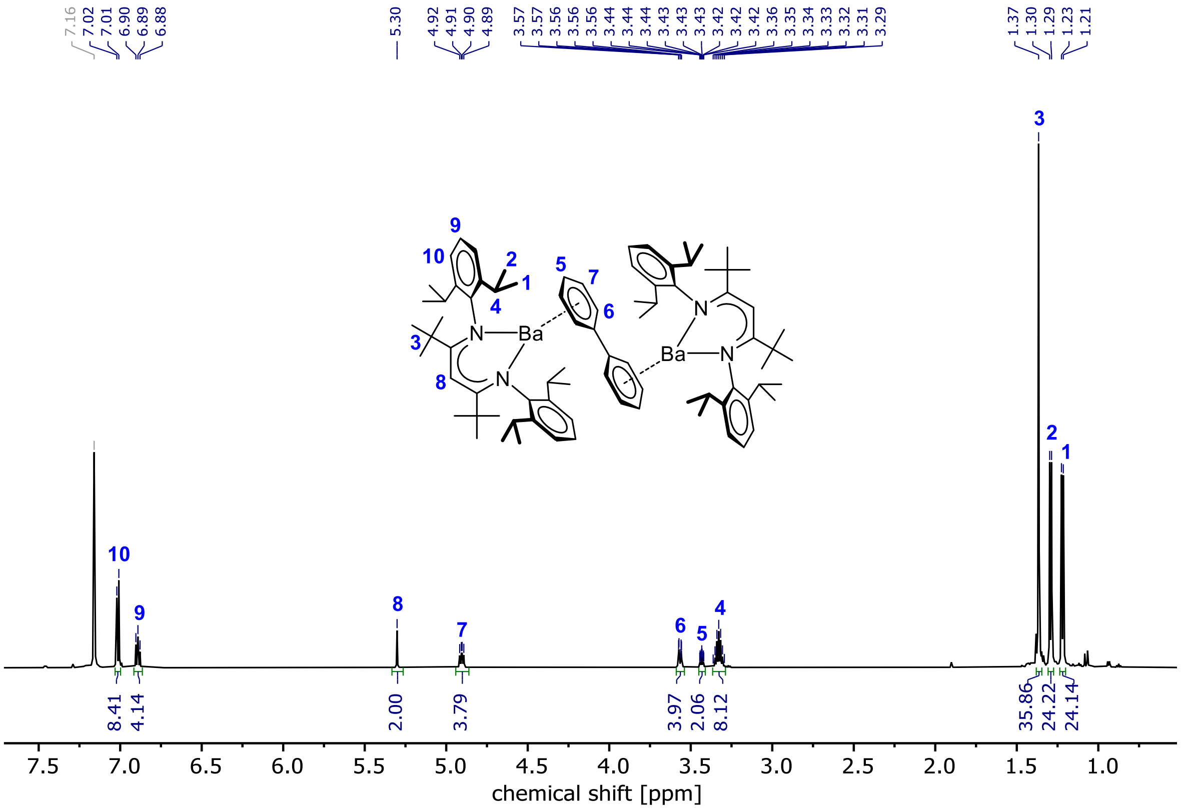

Figure S51. ^{1}H NMR (600.13 MHz, 298 K, C_6D_6) of [{(^{DIPP}BDI*)Ba}$_2$(η^6:η^6-$C_{12}H_{10}$)].

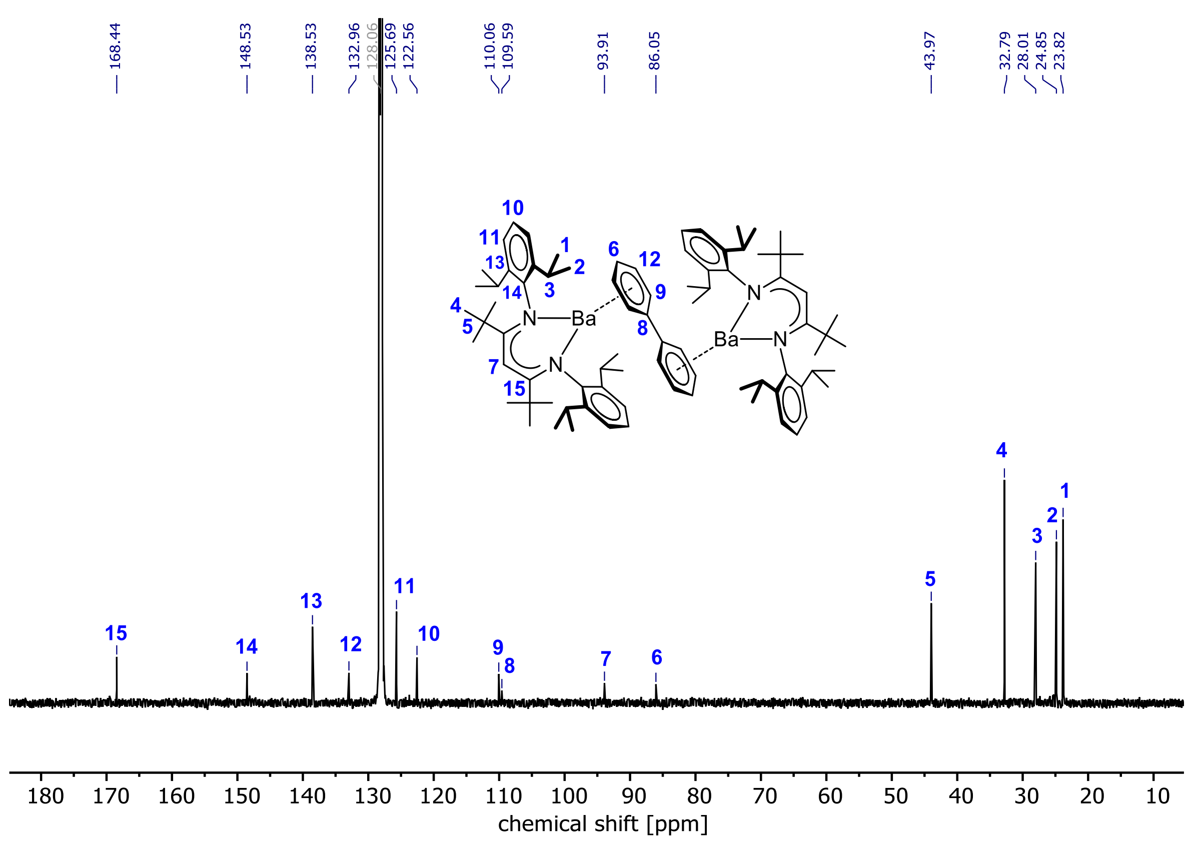

Figure S52. ^{13}C NMR (150.92 MHz, 298 K, C_6D_6) of [{(^{DIPP}BDI*)Ba}$_2$(η^6:η^6-$C_{12}H_{10}$)].

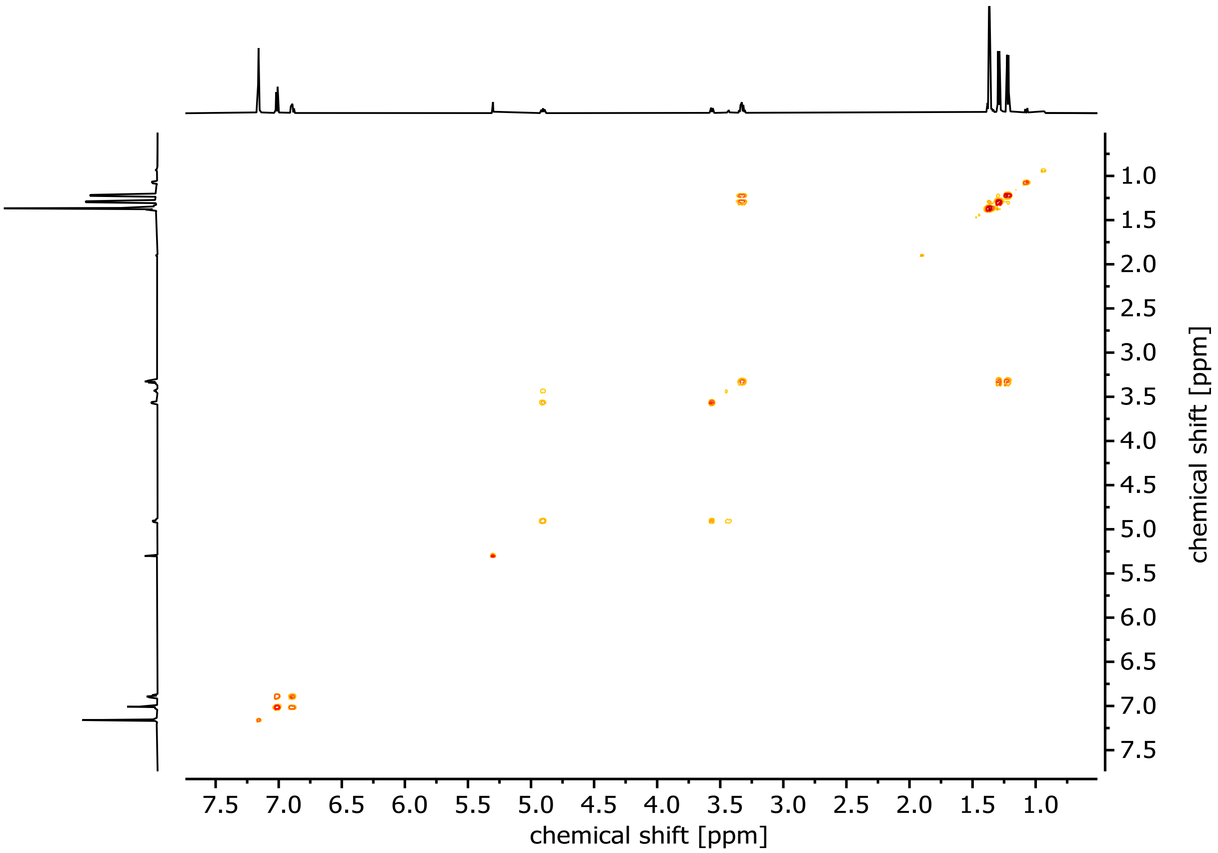

Figure S53. ^{1}H-^{1}H COSY NMR (600.13 MHz, 298 K, C_6D_6) of [{(^{DIPP}BDI*)Ba}$_2$(η^6:η^6-$C_{12}H_{10}$)].

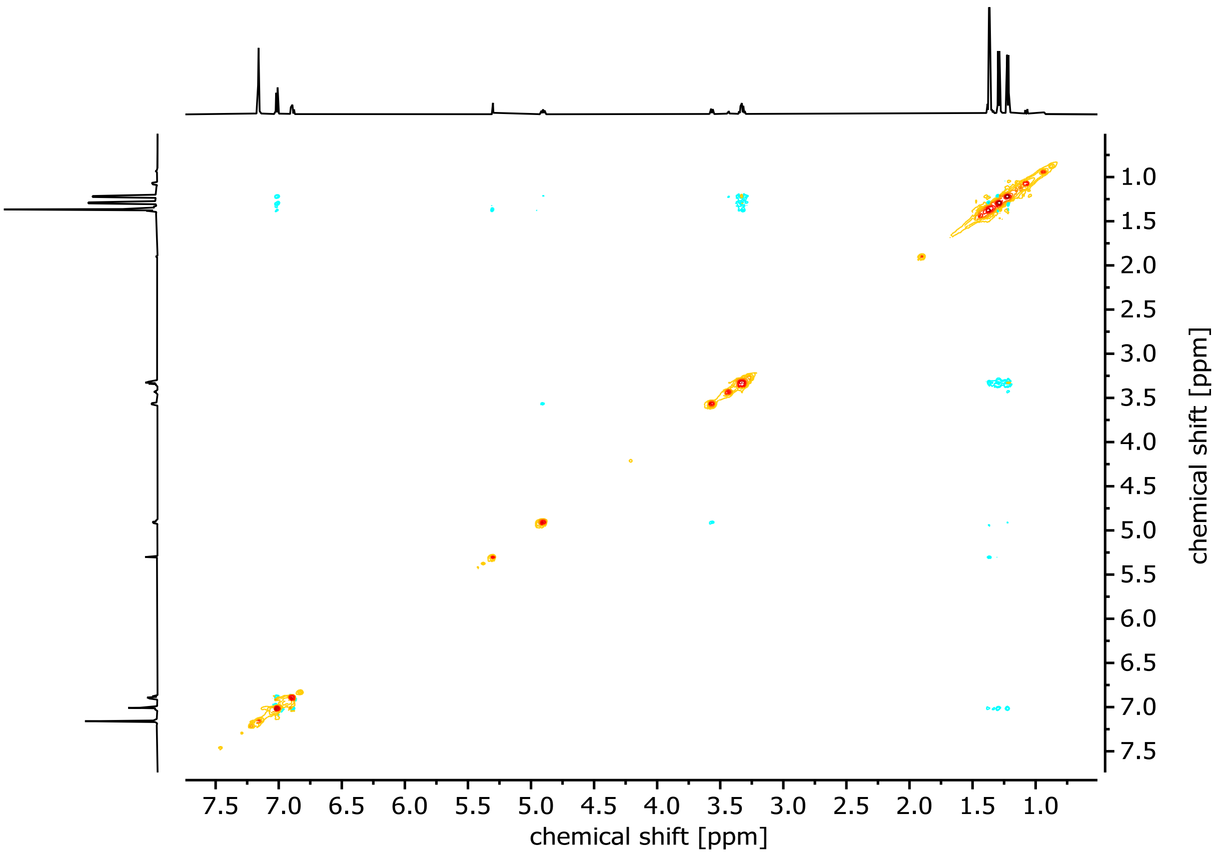

Figure S54. ^{1}H-^{1}H NOESY (400.13 MHz, 298 K, C_6D_6) of [{(^{DIPP}BDI*)Ba}$_2$(η^6:η^6-$C_{12}H_{10}$)].

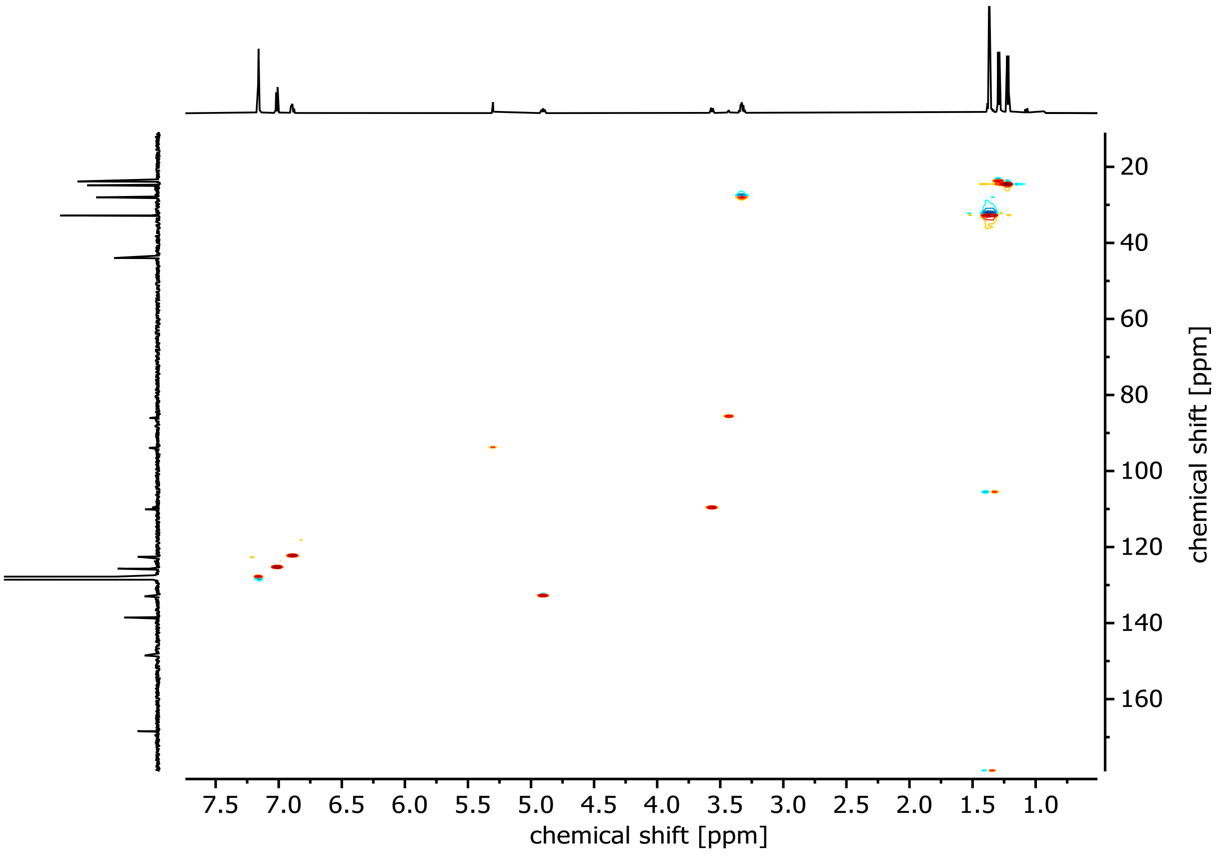

Figure S55. ^{1}H-^{13}C HSQC NMR (600.13/150.92 MHz, 298 K, C_6D_6) of [{(^{DIPP}BDI*)Ba}$_2$(η^6:η^6-$C_{12}H_{10}$)].

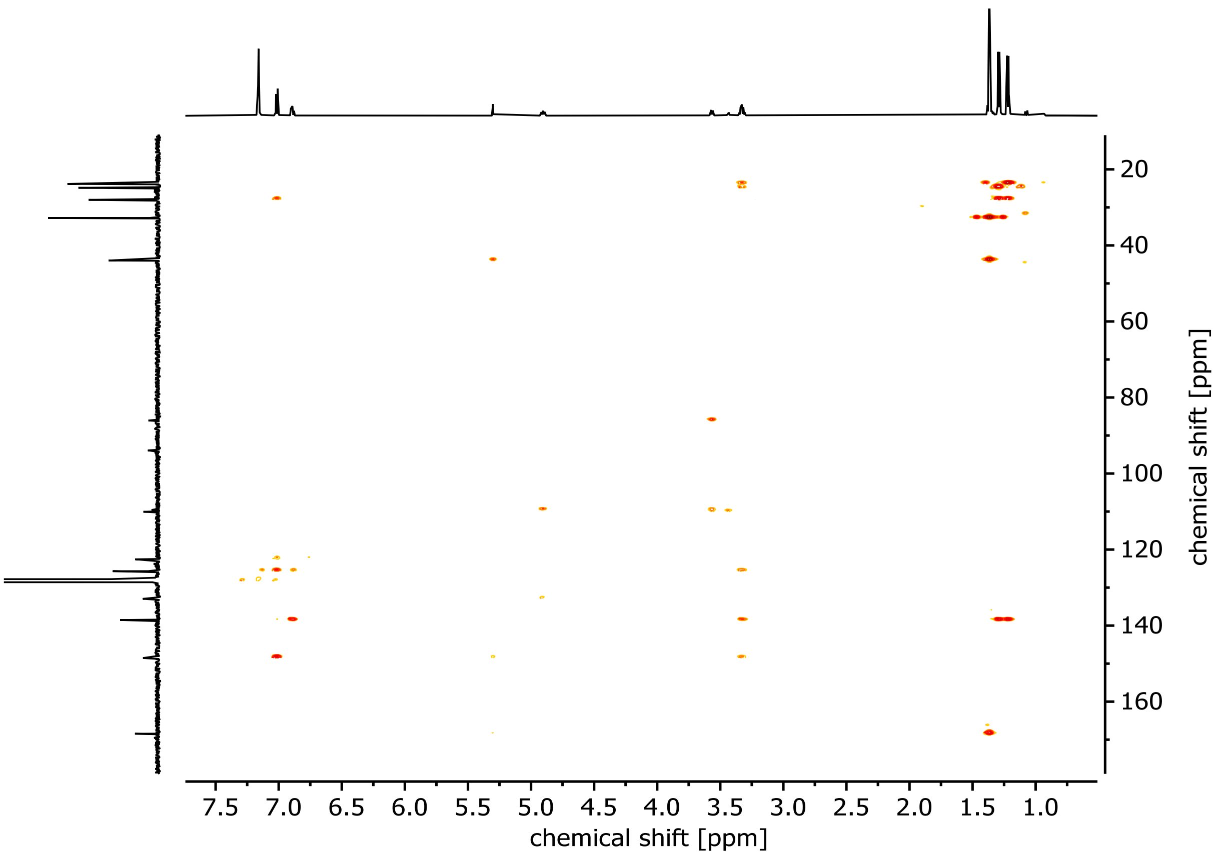

Figure S56. ^{1}H-^{13}C HMBC NMR (600.13/150.92 MHz, 298 K, C_6D_6) of [{(^{DIPP}BDI*)Ba}$_2$(η^6:η^6-$C_{12}H_{10}$)].

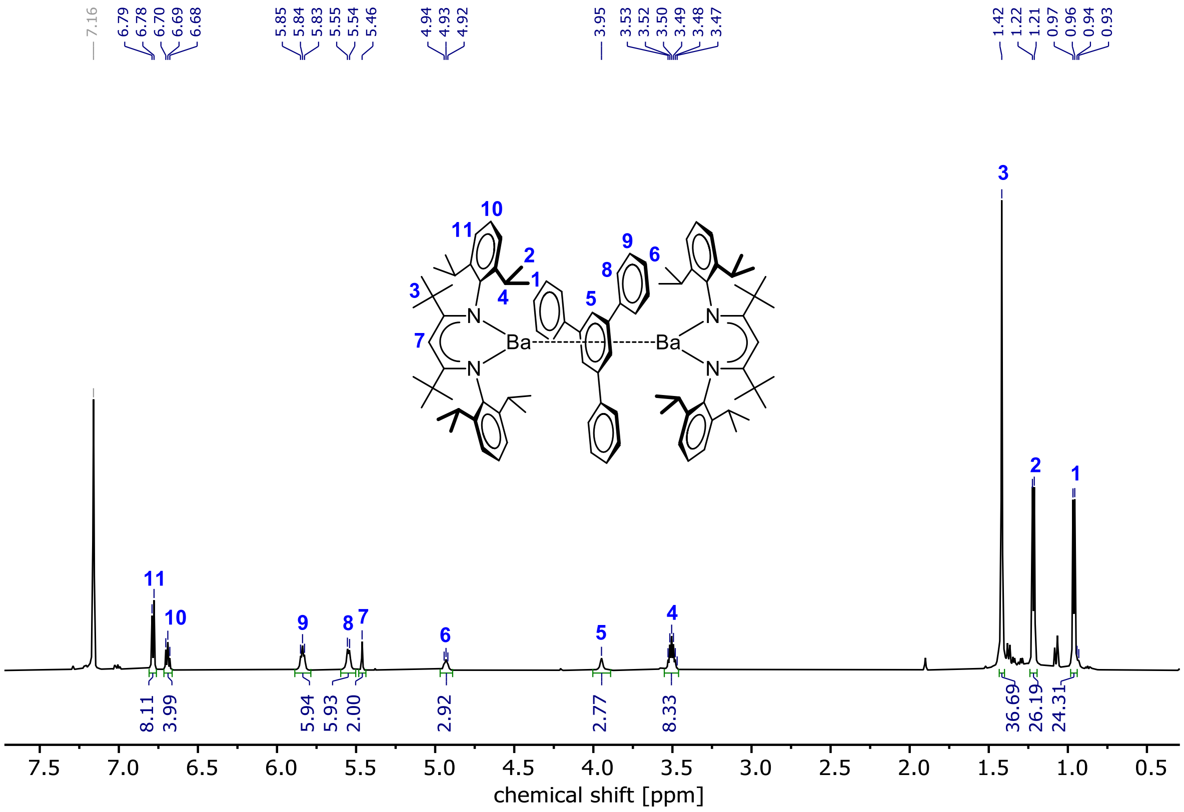

Figure S57. ^{1}H NMR (600.13 MHz, 298 K, C_6D_6) of [{(^{DIPP}BDI*)Ba}$_2$(η^6:η^6-1,3,5-$Ph_3C_6H_3$)].

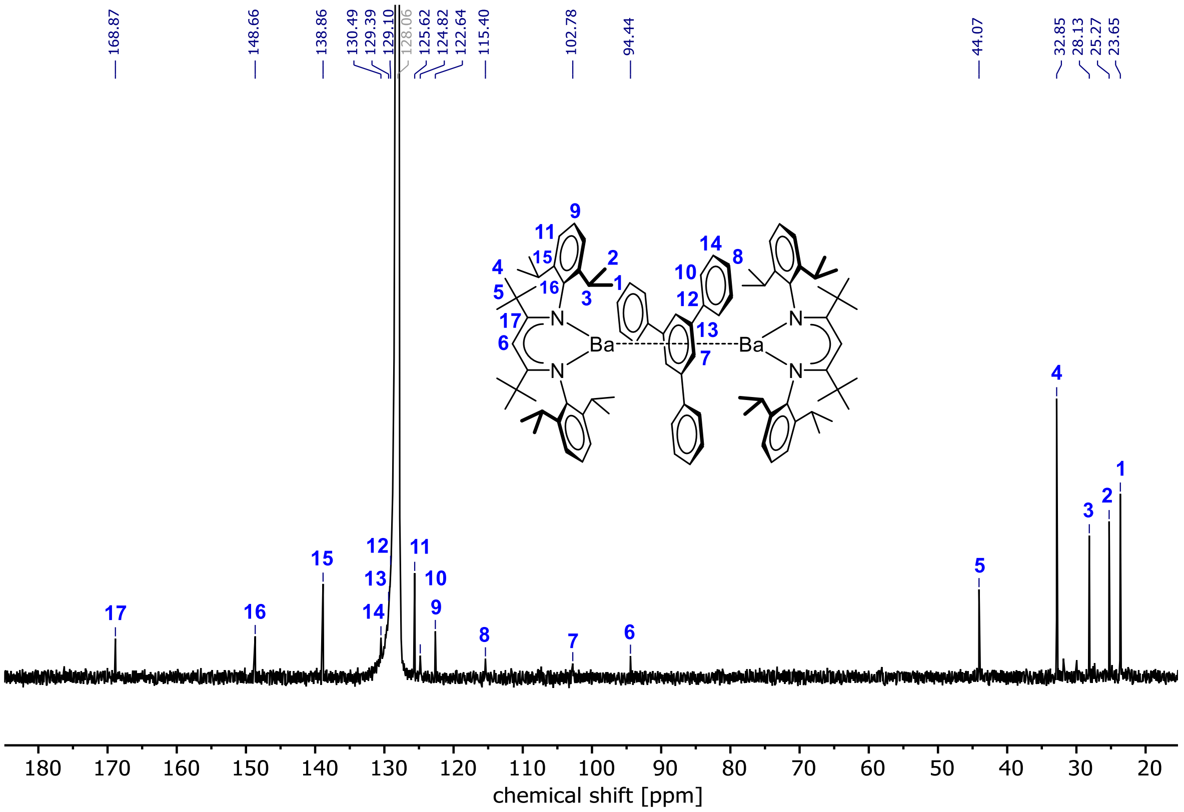

Figure S58. ^{13}C NMR (150.92 MHz, 298 K, C_6D_6) of [{(^{DIPP}BDI*)Ba}$_2$(η^6:η^6-1,3,5-$Ph_3C_6H_3$)].

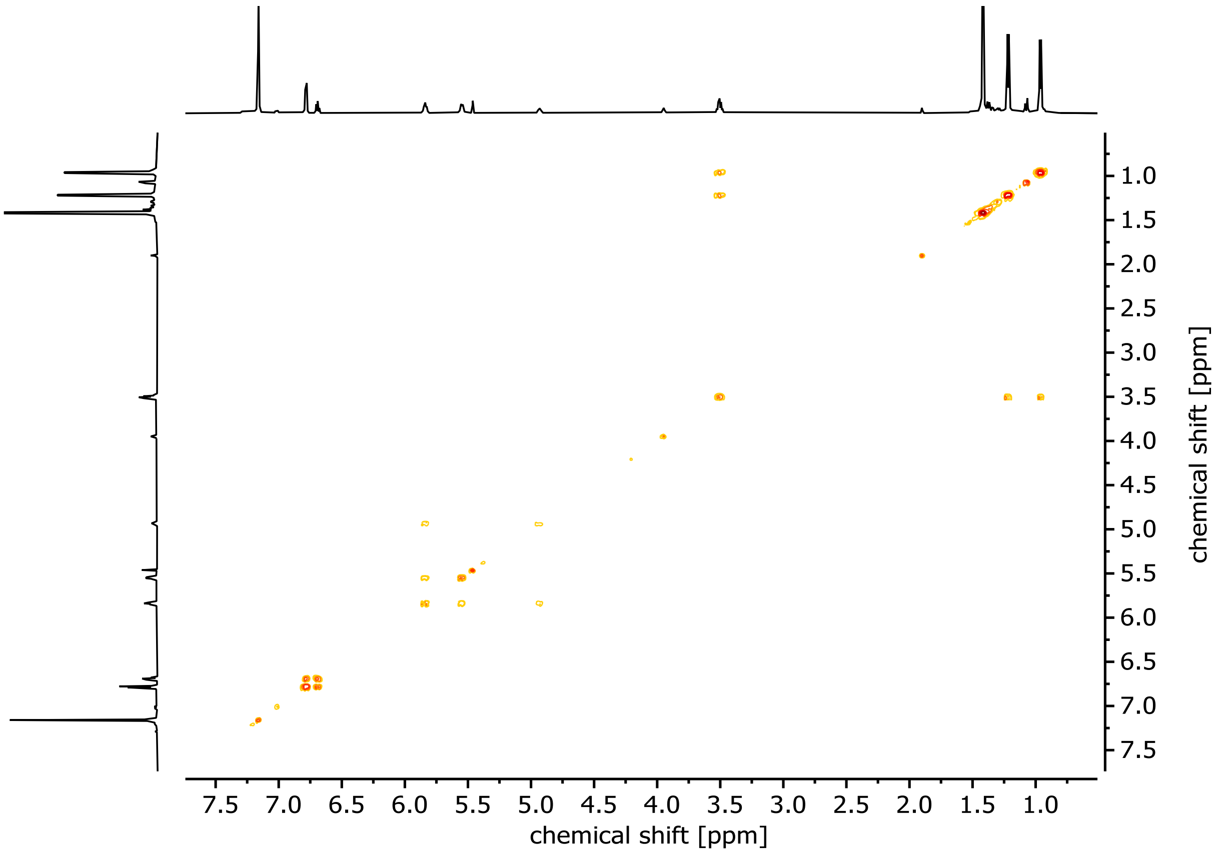

Figure S59. ^1H-^1H COSY NMR (600.13 MHz, 298 K, C_6D_6) of [{(^{DIPP}BDI*)Ba}$_2$(η^6:η^6-1,3,5-$Ph_3C_6H_3$)].

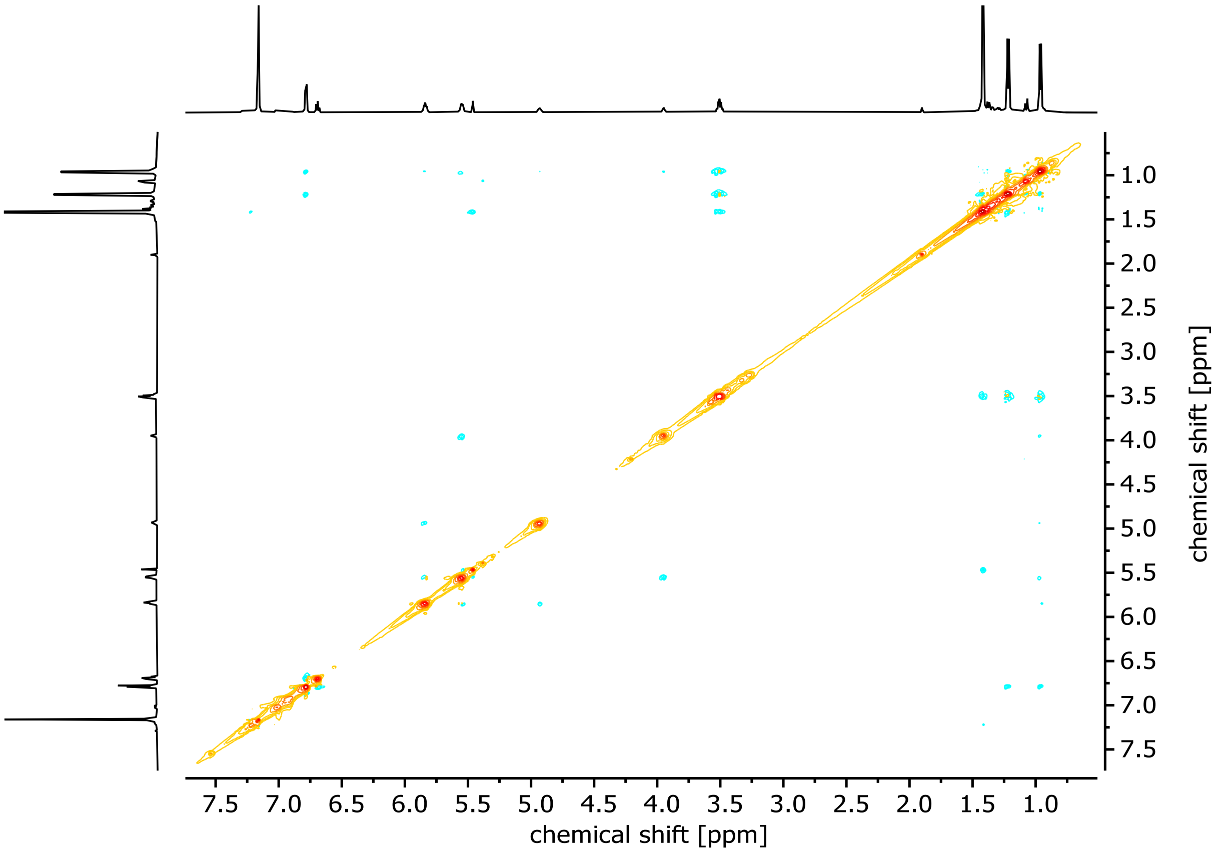

Figure S60. ^1H-^1H NOESY (400.13 MHz, 298 K, C_6D_6) of [{(^{DIPP}BDI*)Ba}$_2$(η^6:η^6-1,3,5-$Ph_3C_6H_3$)].

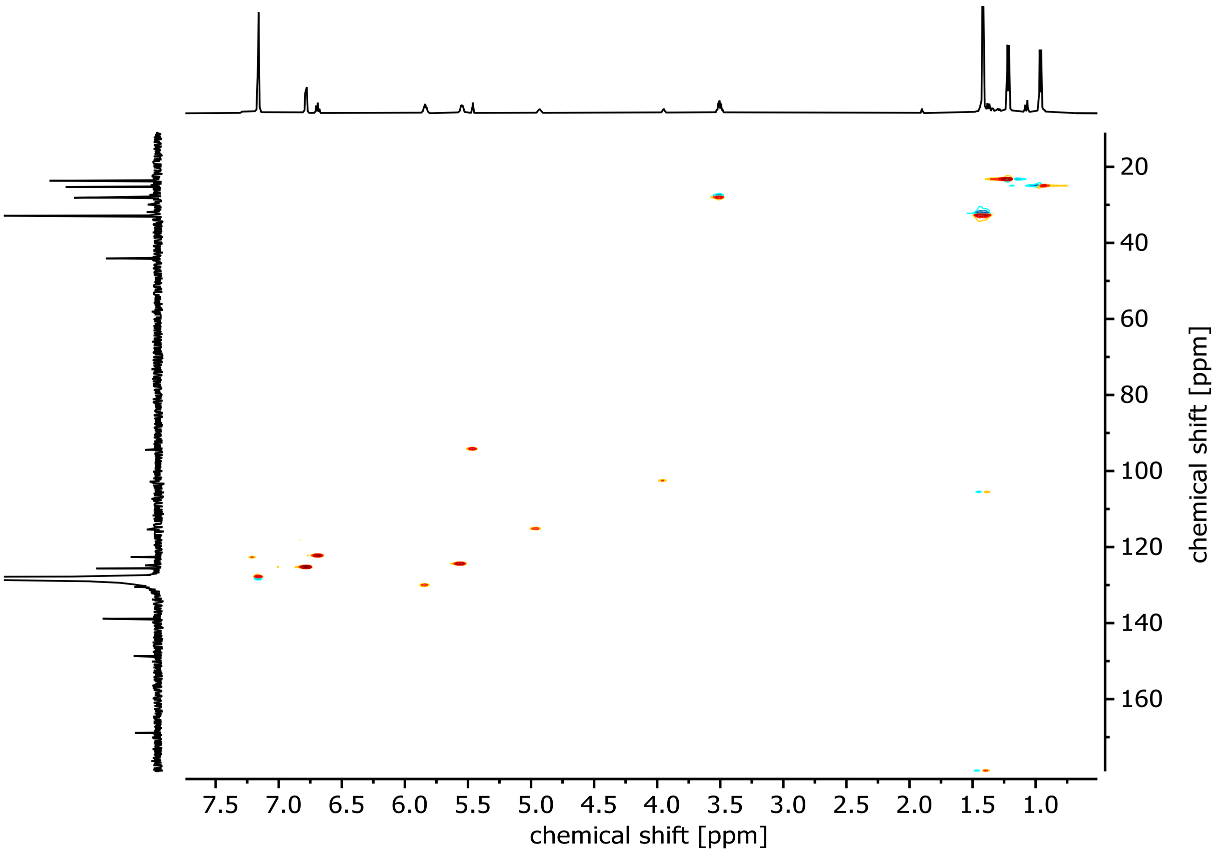

Figure S61. ^{1}H-^{13}C HSQC NMR (600.13/150.92 MHz, 298 K, C_6D_6) of [{(^{DIPP}BDI*)Ba}$_2$(η^6:η^6-1,3,5-$Ph_3C_6H_3$)].

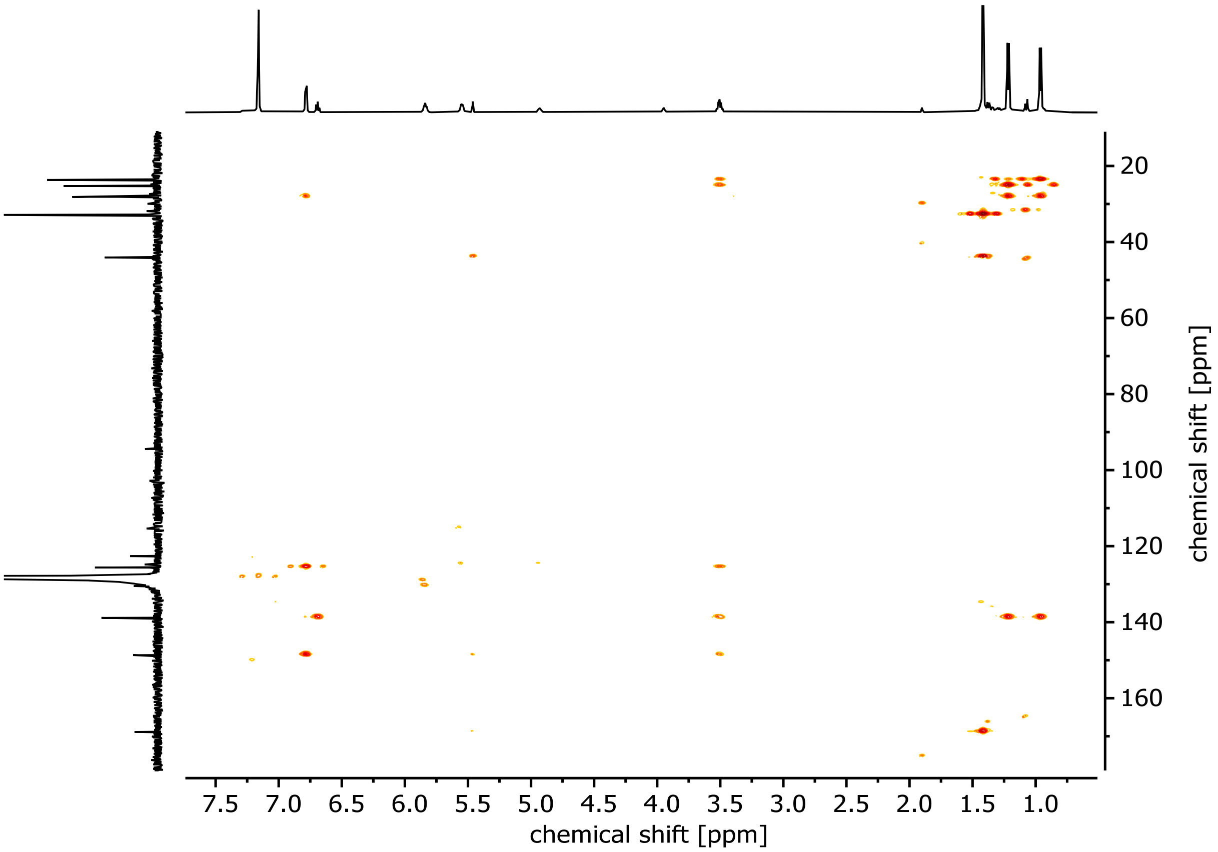

Figure S62. ^{1}H-^{13}C HMBC NMR (600.13/150.92 MHz, 298 K, C_6D_6) of [{(^{DIPP}BDI*)Ba}$_2$(η^6:η^6-1,3,5-$Ph_3C_6H_3$)].

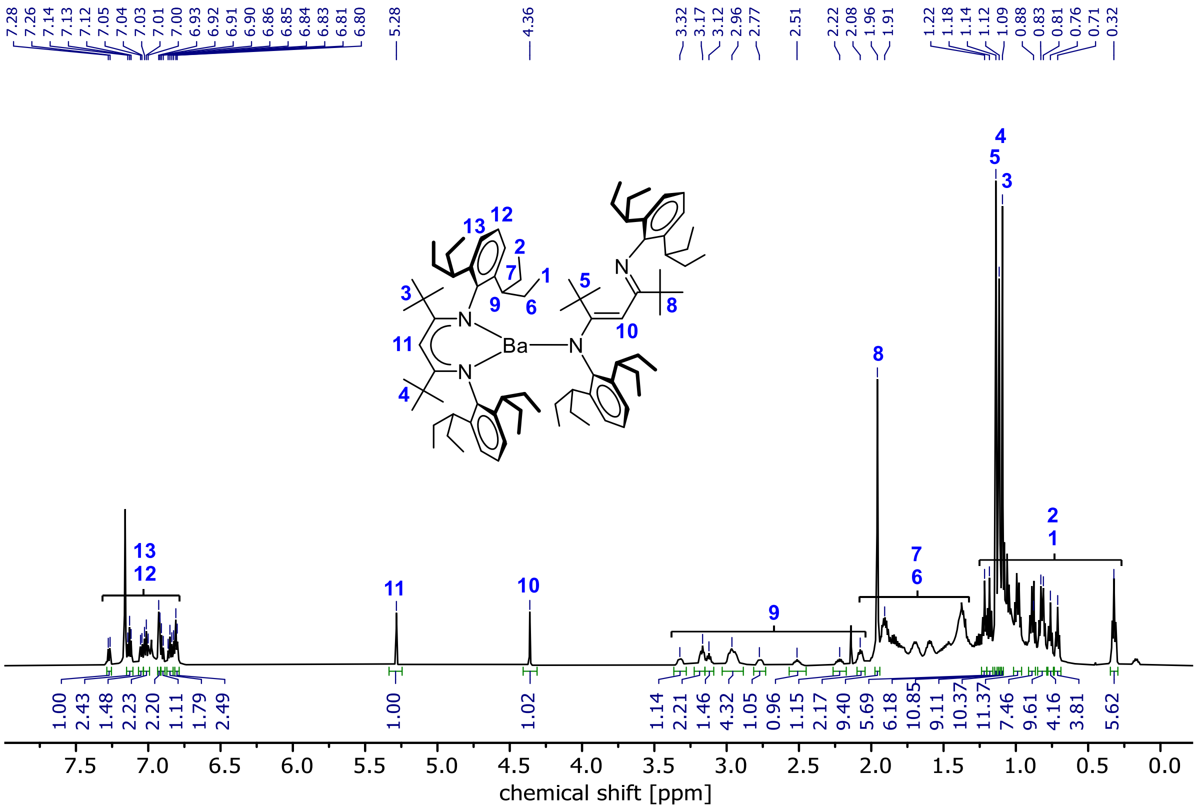

Figure S63. ^{1}H NMR (600.13 MHz, 298 K, C_6D_6) of $[(\kappa^2,\kappa^1\text{-}^{DIPeP}BDI^*)_2Ba]$.

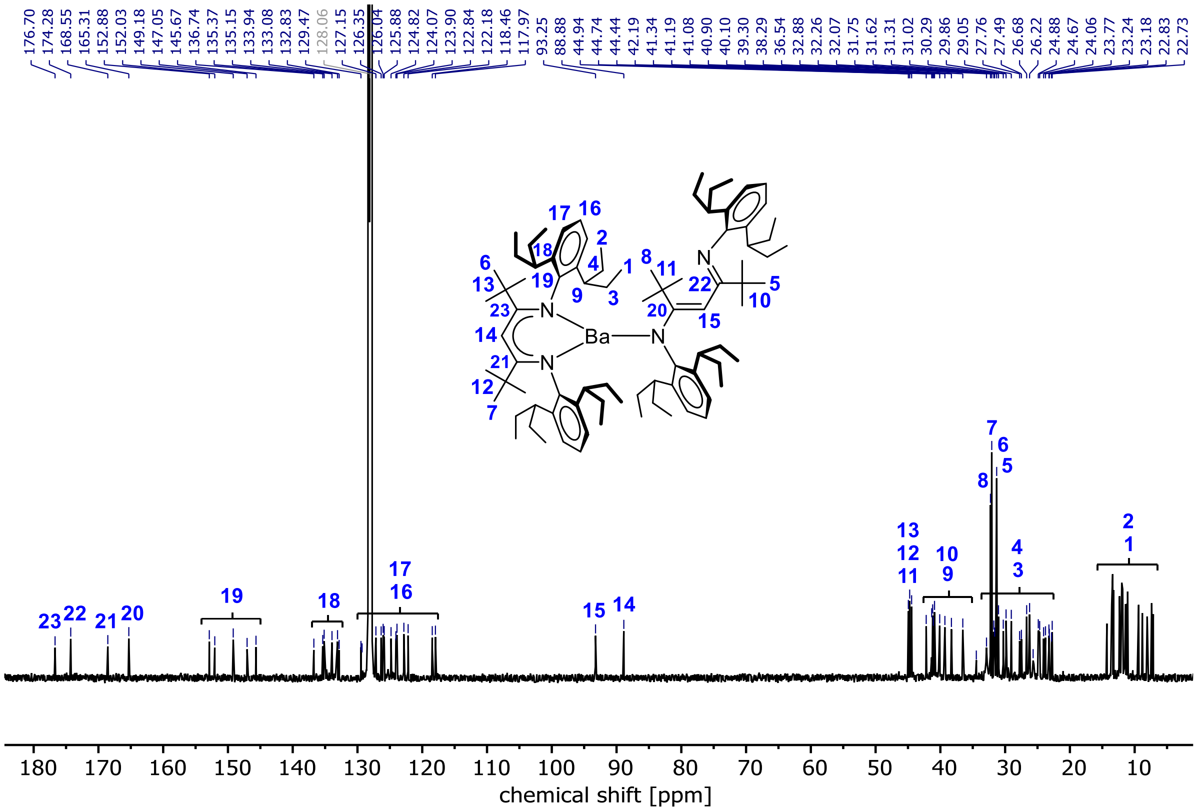

Figure S64. ^{13}C NMR (150.92 MHz, 298 K, C_6D_6) of of $[(\kappa^2,\kappa^1\text{-}^{DIPeP}BDI^*)_2Ba]$.

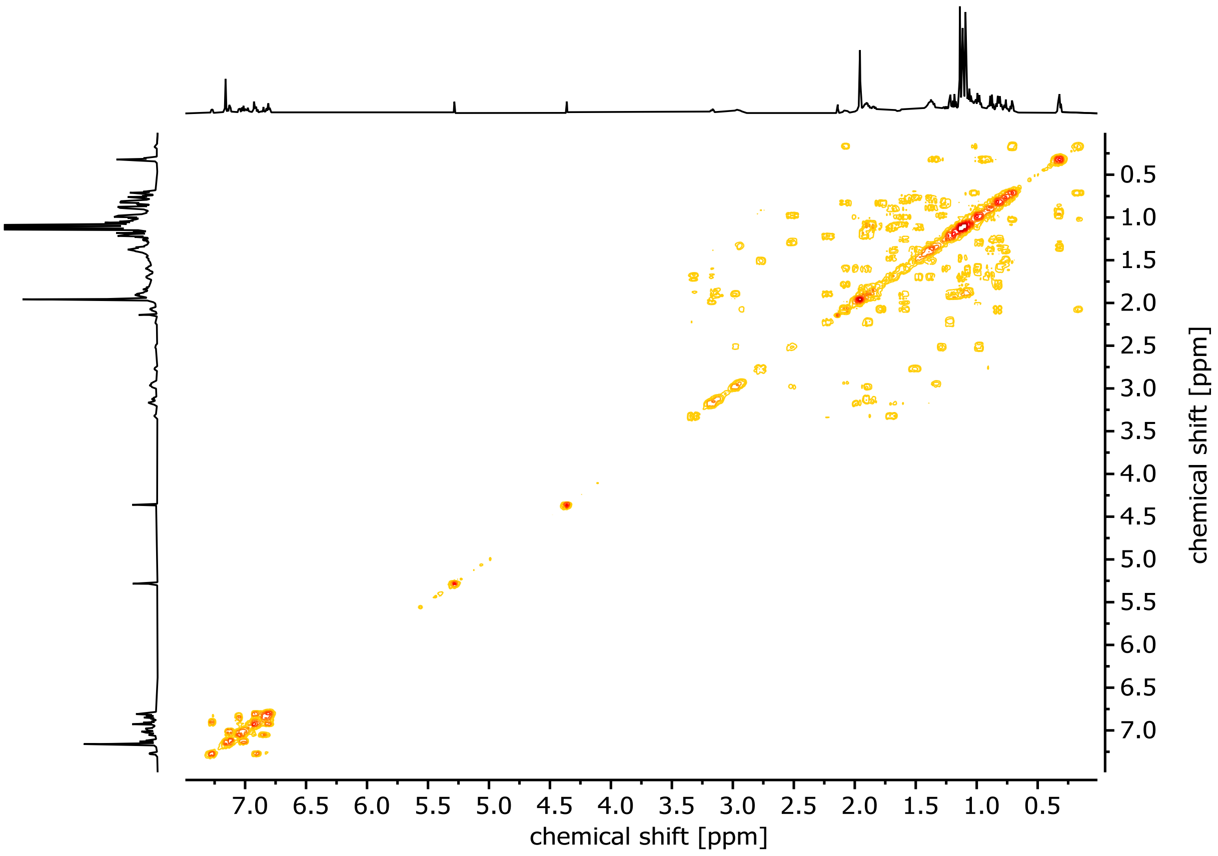

Figure S65. ^{1}H-^{1}H COSY NMR (600.13 MHz, 298 K, C_6D_6) of [(κ^2,κ^1-^{DIPeP}BDI*)$_2$Ba].

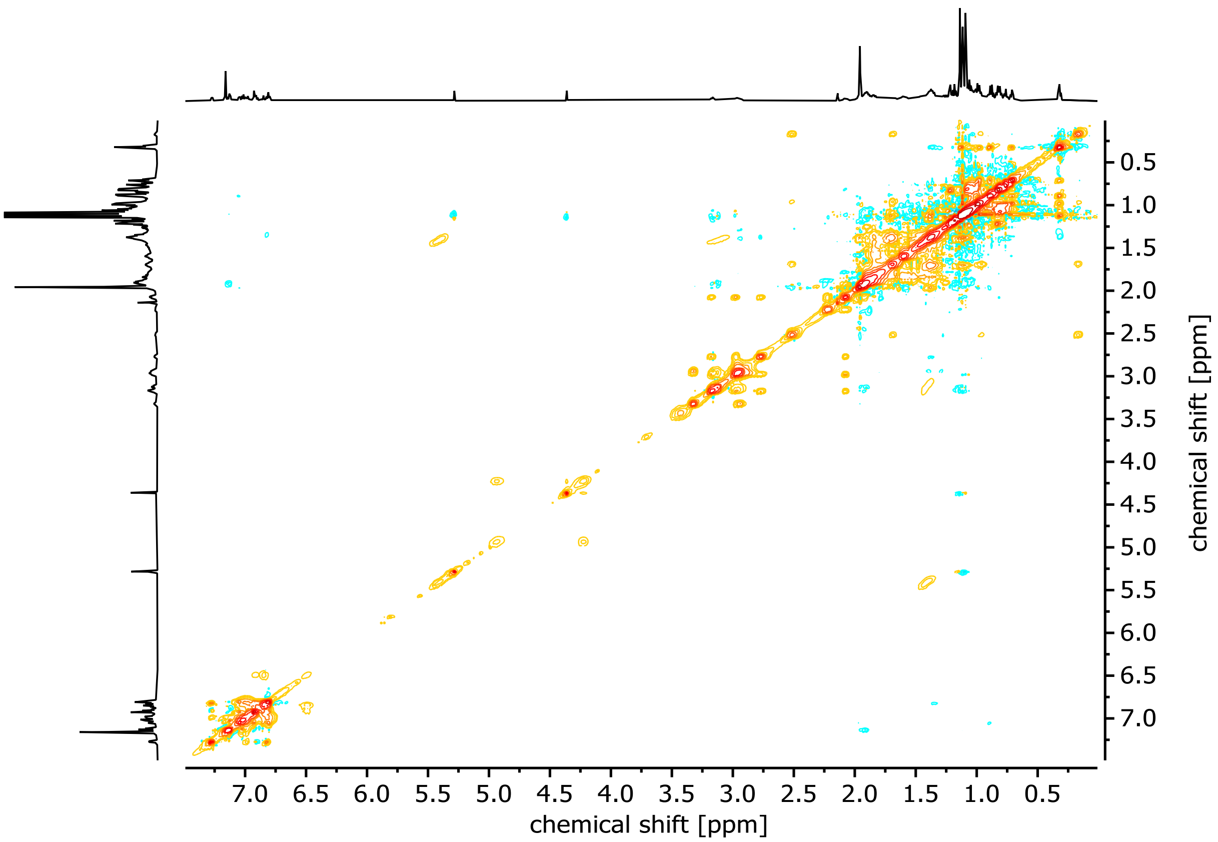

Figure S66. ^{1}H-^{1}H NOESY (400.13 MHz, 298 K, C_6D_6) of [(κ^2,κ^1-^{DIPeP}BDI *)$_2$Ba].

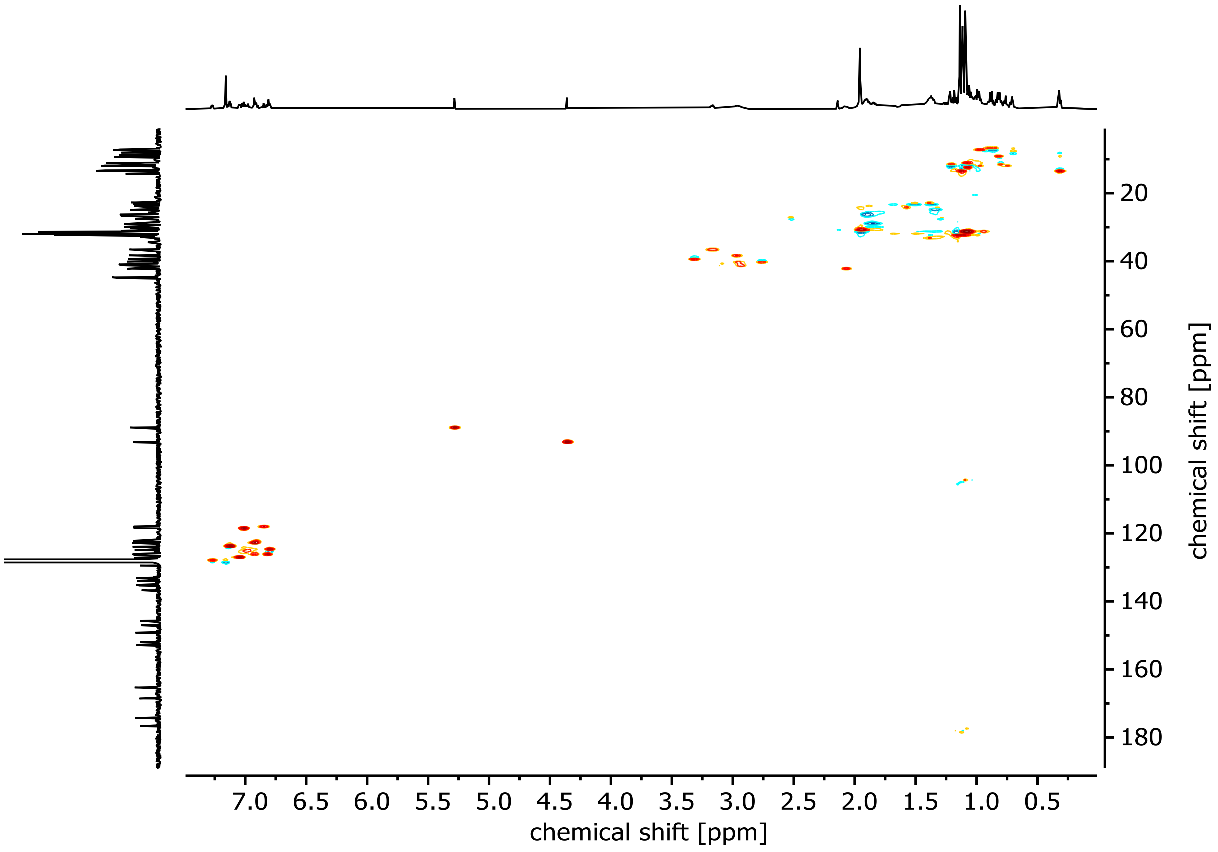

Figure S67. ^{1}H-^{13}C HSQC NMR (600.13/150.92 MHz, 298 K, C_6D_6) of [(κ^2,κ^1-^{DIPeP}BDI *)$_2$Ba].

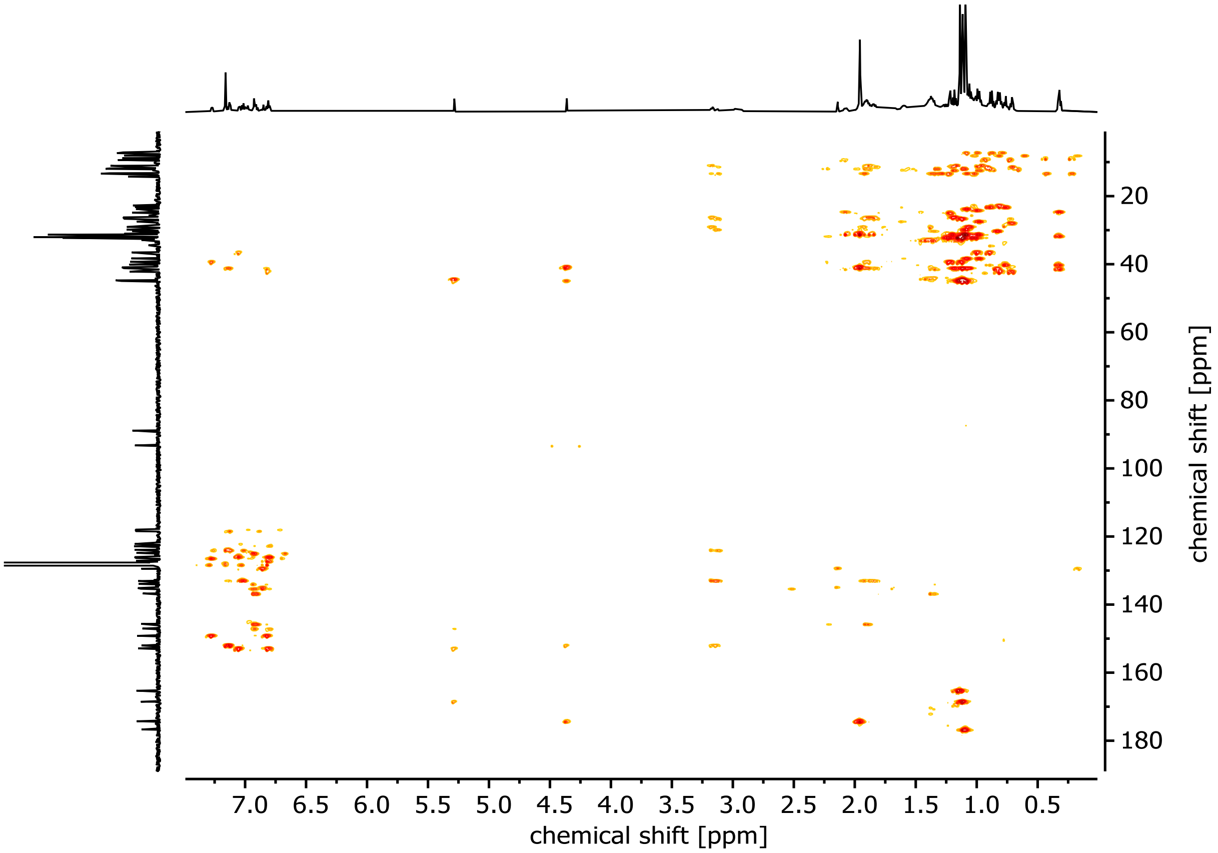

Figure S68. ^{1}H-^{13}C HMBC NMR (600.13/150.92 MHz, 298 K, C_6D_6) of [(κ^2,κ^1-^{DIPeP}BDI *)$_2$Ba].

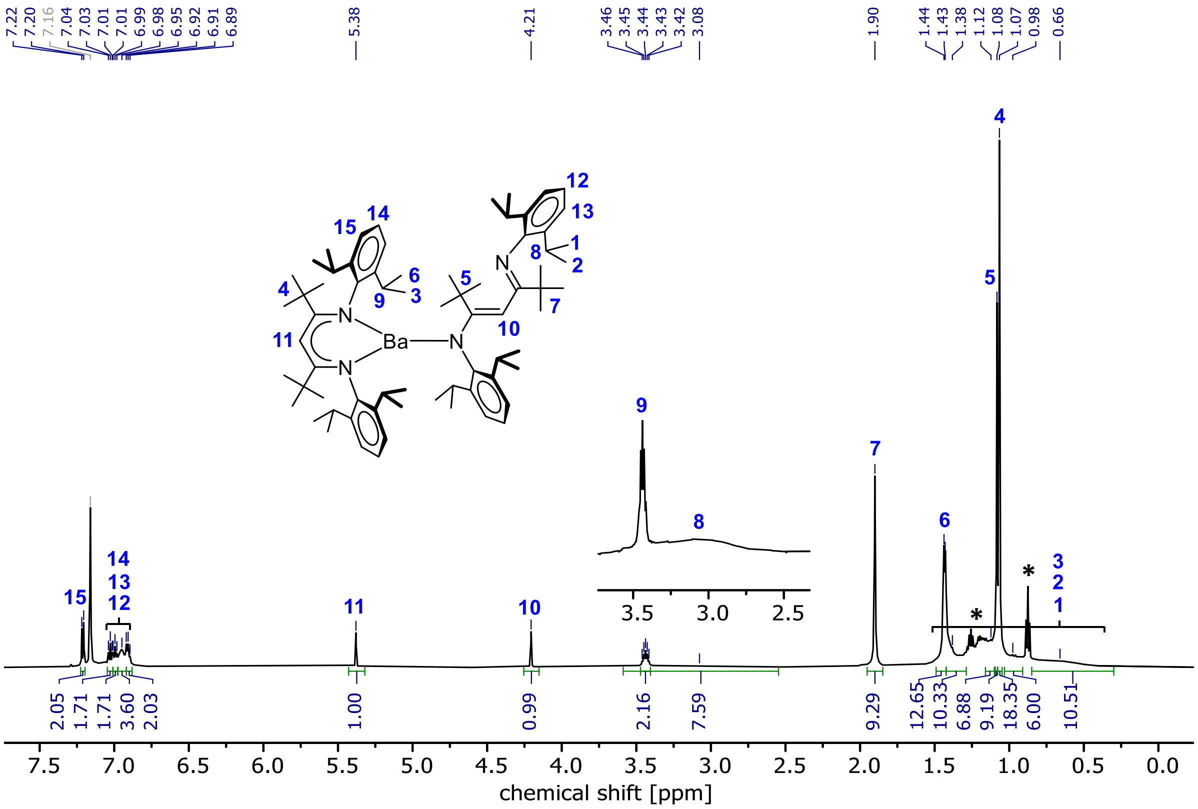

Figure S69. ^{1}H NMR (600.13 MHz, 298 K, C_6D_6) of [(κ^2,κ^1-^{DIPP}BDI*)$_2$Ba]. * denotes *n*-pentane.

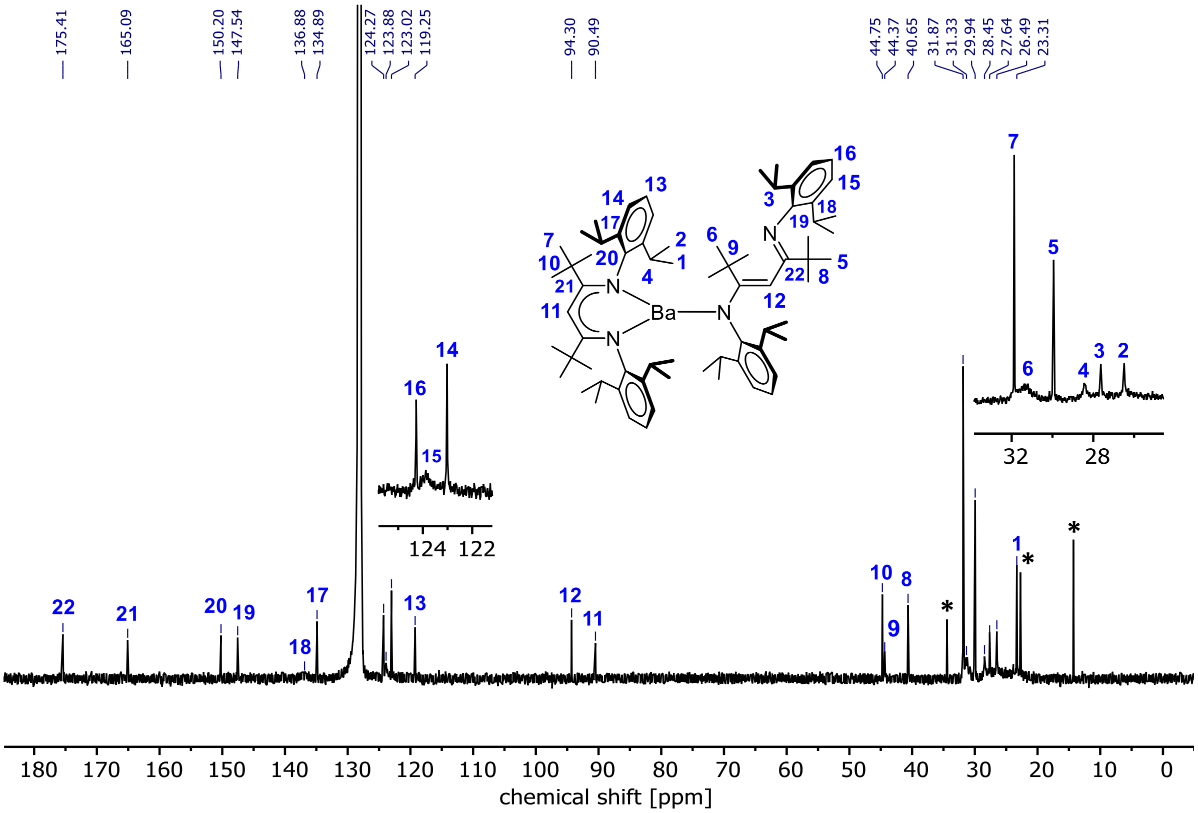

Figure S70. ^{1}H NMR (600.13 MHz, 298 K, C_6D_6) of [(κ^2,κ^1-^{DIPP}BDI*)$_2$Ba]. * denotes *n*-pentane.

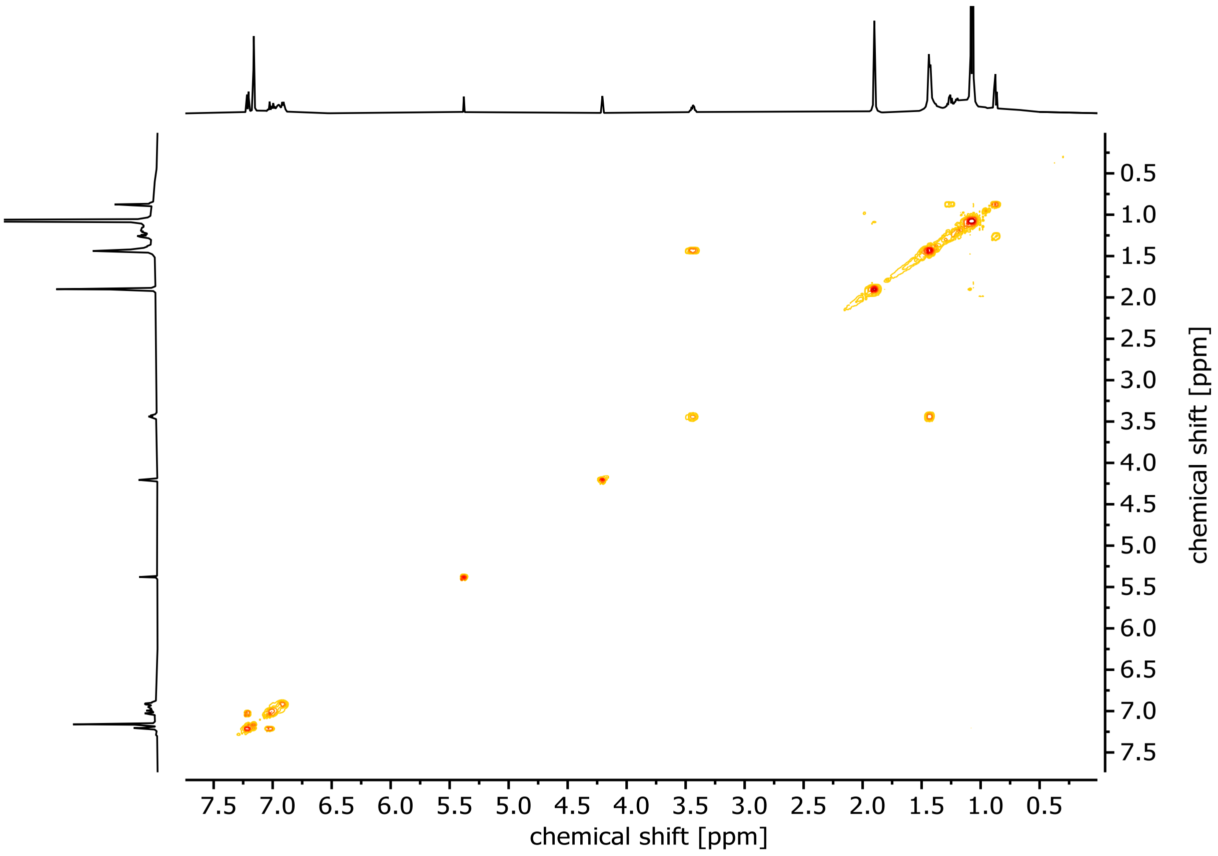

Figure S71. ^{1}H-^{1}H COSY NMR (600.13 MHz, 298 K, C_6D_6) of [(κ^2,κ^1-^{DIPP}BDI*)$_2$Ba].

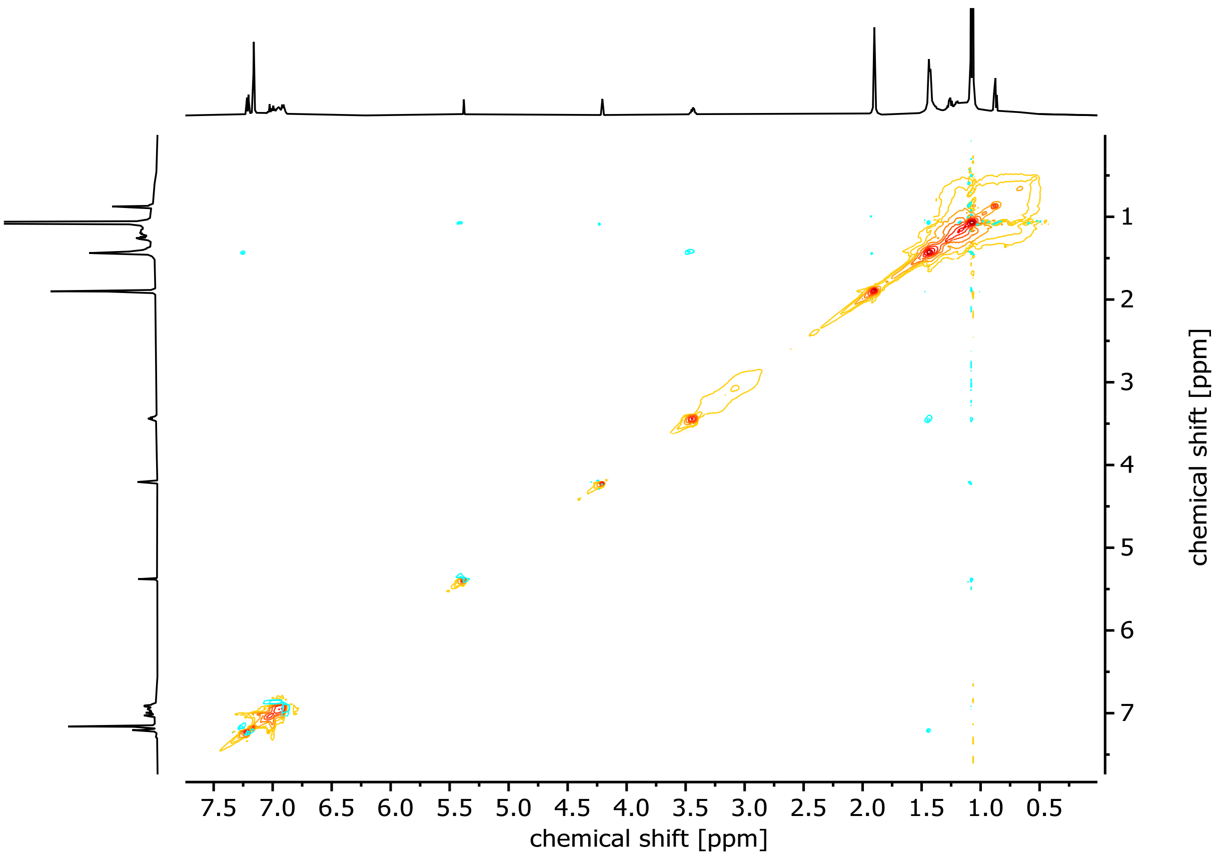

Figure S72. ^{1}H-^{1}H NOESY (400.13 MHz, 298 K, C_6D_6) of [(κ^2,κ^1-^{DIPP}BDI*)$_2$Ba].

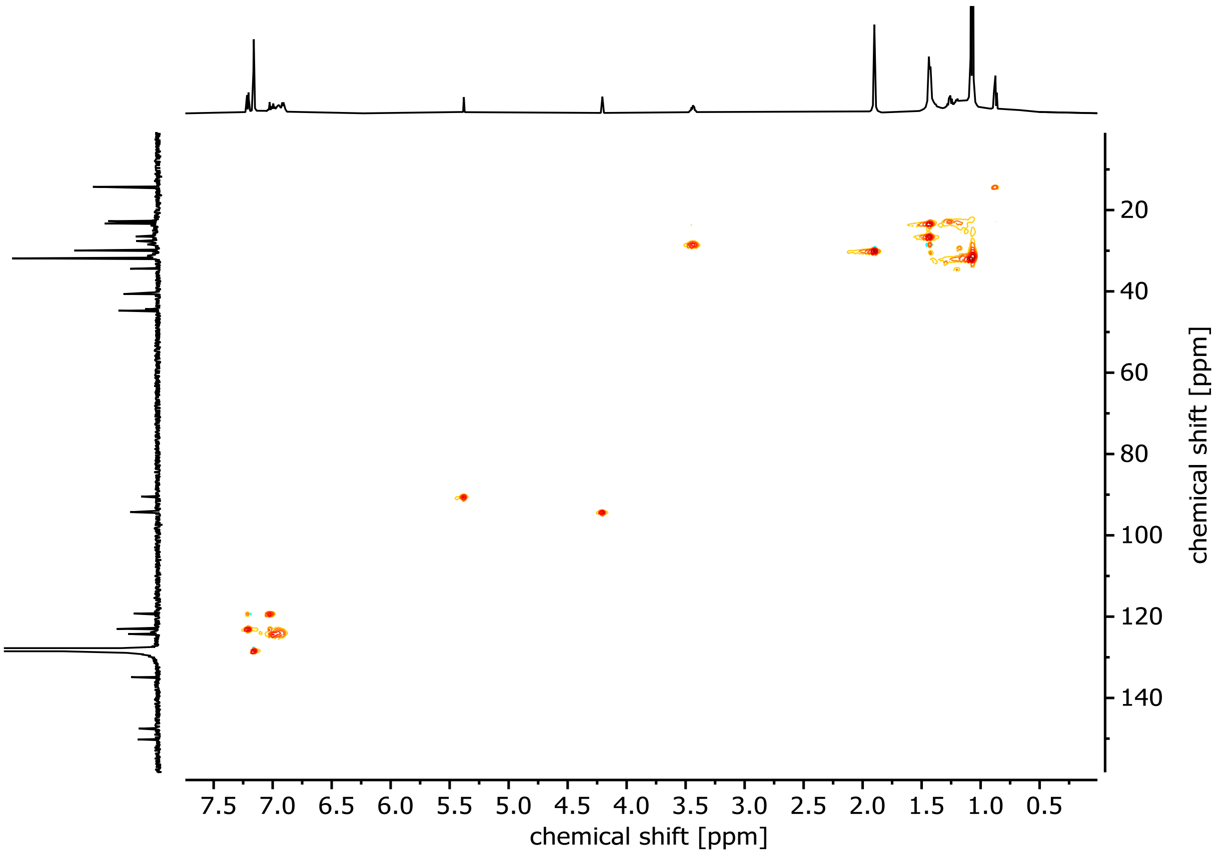

Figure S73. ^{1}H-^{13}C HSQC NMR (600.13/150.92 MHz, 298 K, C_6D_6) of [(κ^2,κ^1-^{DIPP}BDI*)$_2$Ba].

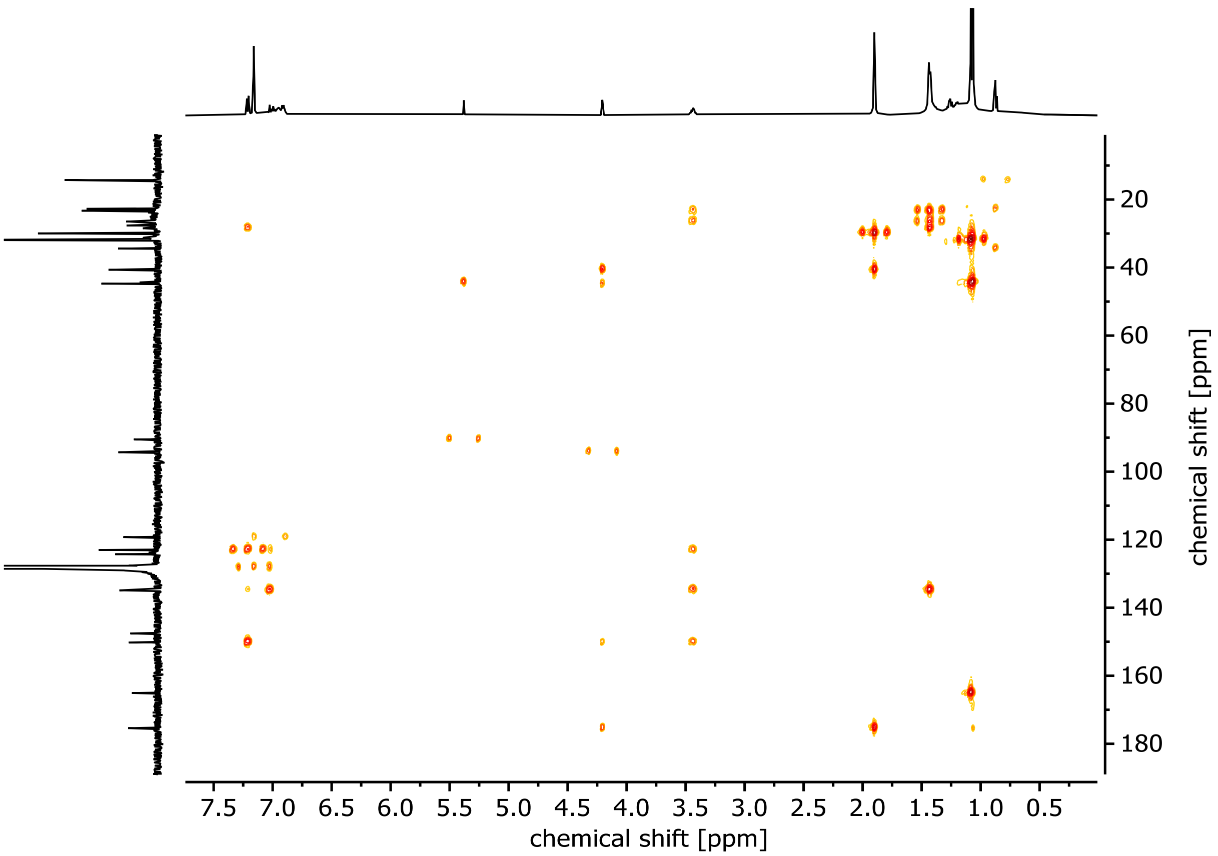

Figure S74. ^{1}H-^{13}C HMBC NMR (600.13/150.92 MHz, 298 K, C_6D_6) of [(κ^2,κ^1-^{DIPP}BDI*)$_2$Ba].

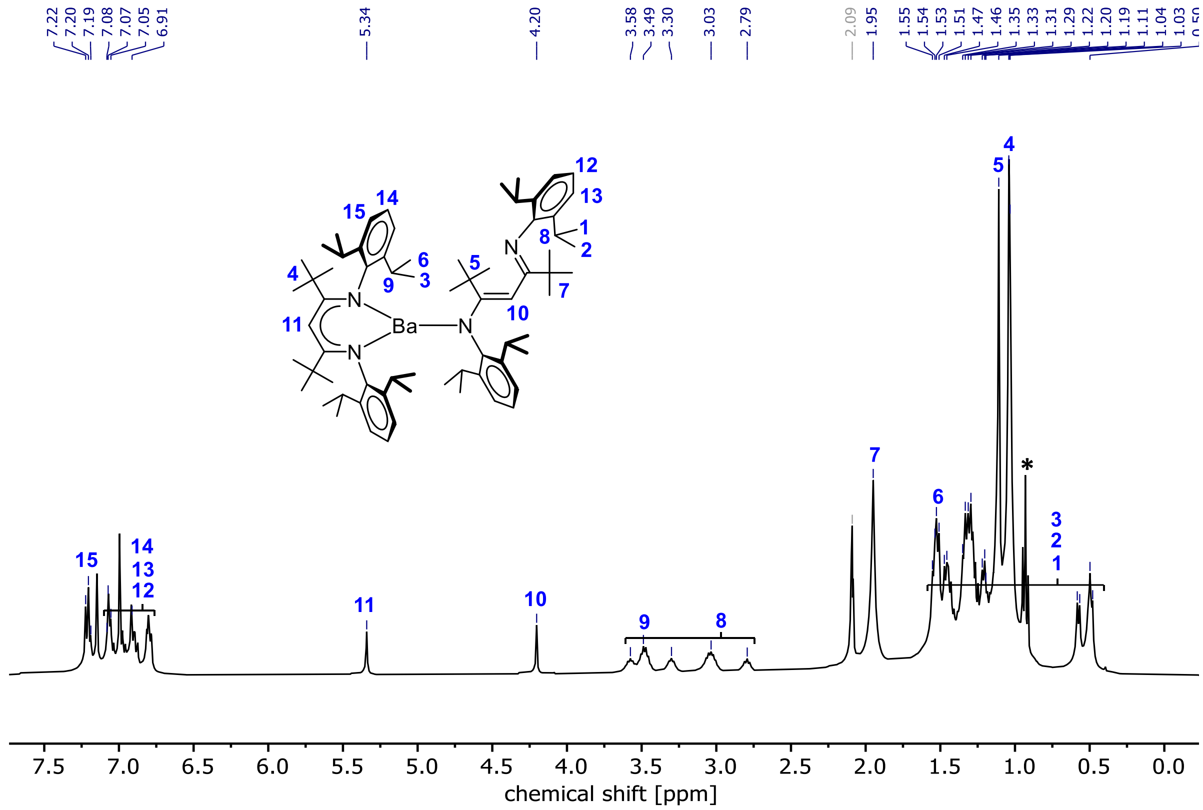

Figure S75. ^{1}H NMR (600.13 MHz, 223 K, C_6D_6) of [(*κ*^{2},*κ*^{1}-^{DIPP}BDI*)$_2$Ba]. * denotes *n*-pentane.

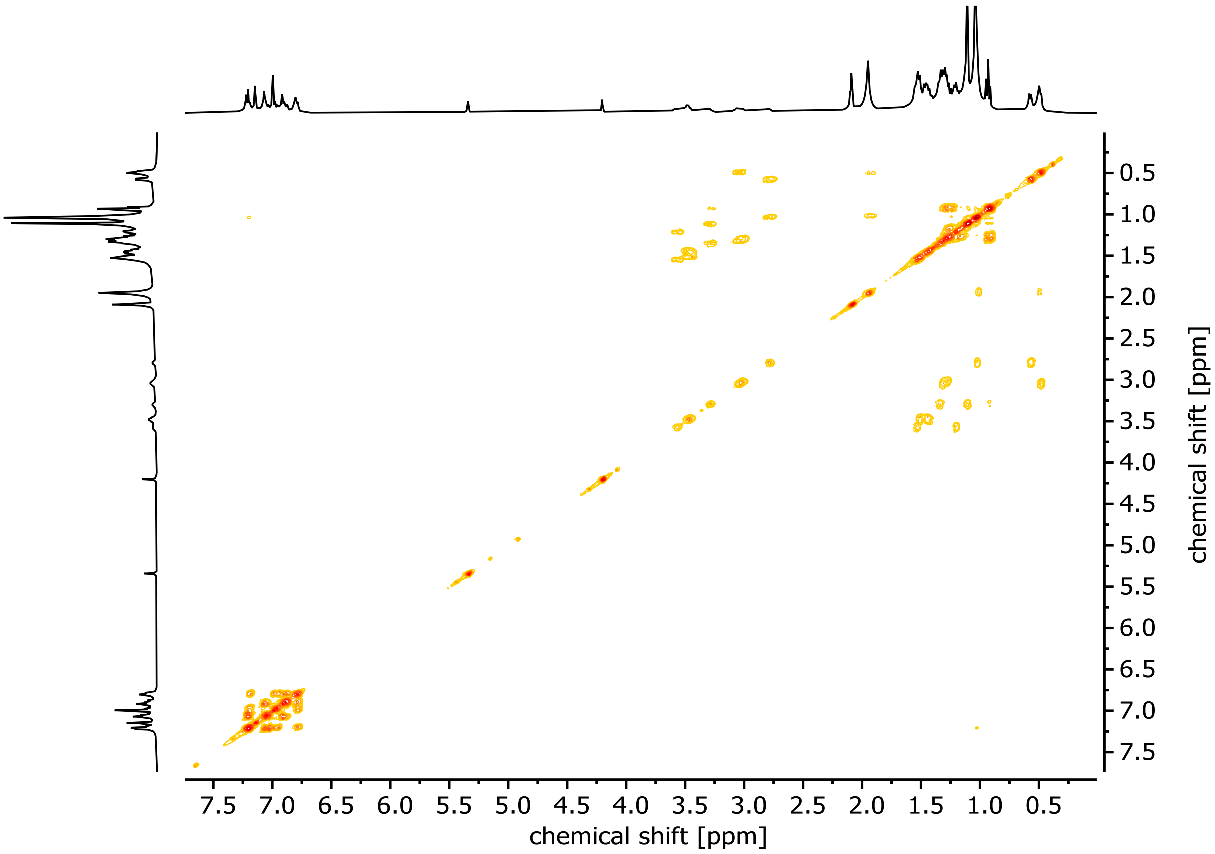

Figure S76. ^{1}H-^{1}H COSY NMR (400.13 MHz, 223 K, C_7D_8) of [(*κ*^{2},*κ*^{1}-^{DIPP}BDI*)$_2$Ba].

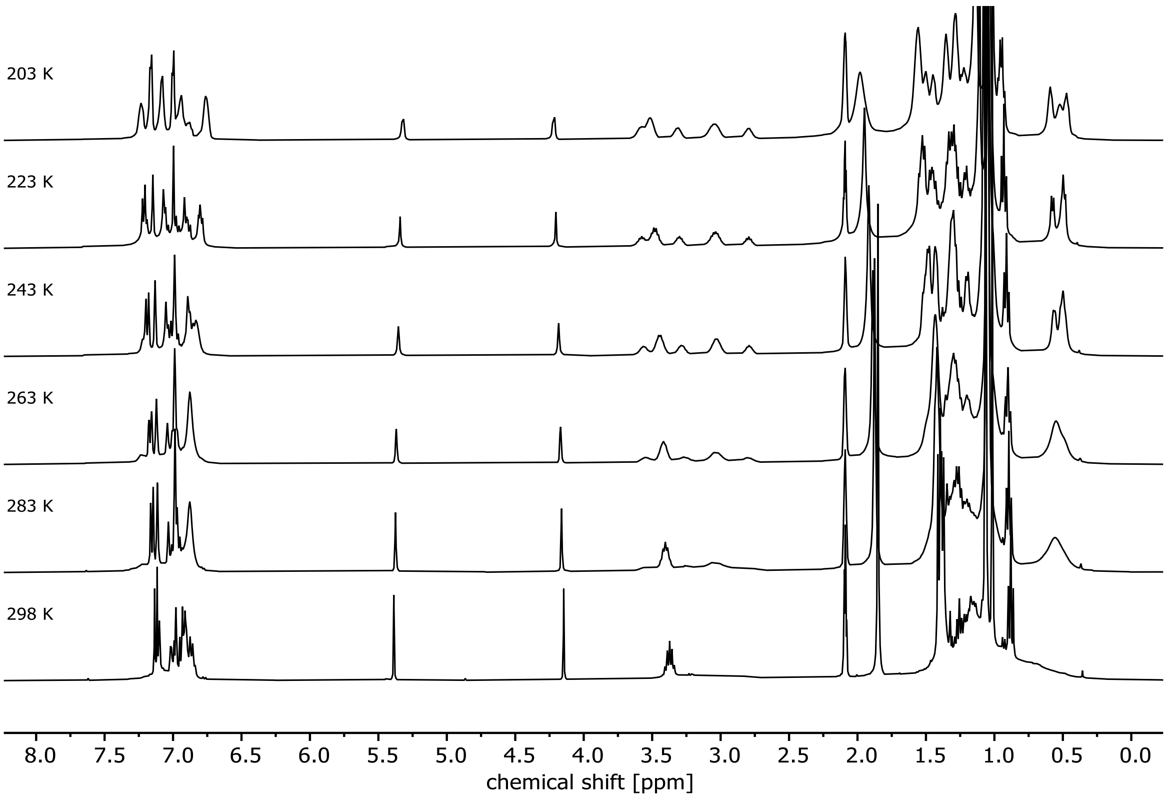

Figure S77 ^{1}H NMR variable temperature spectra (400.13 MHz, C_7D_8) of [(κ^2,κ^1-^{DIPP}BDI*)$_2$Ba].

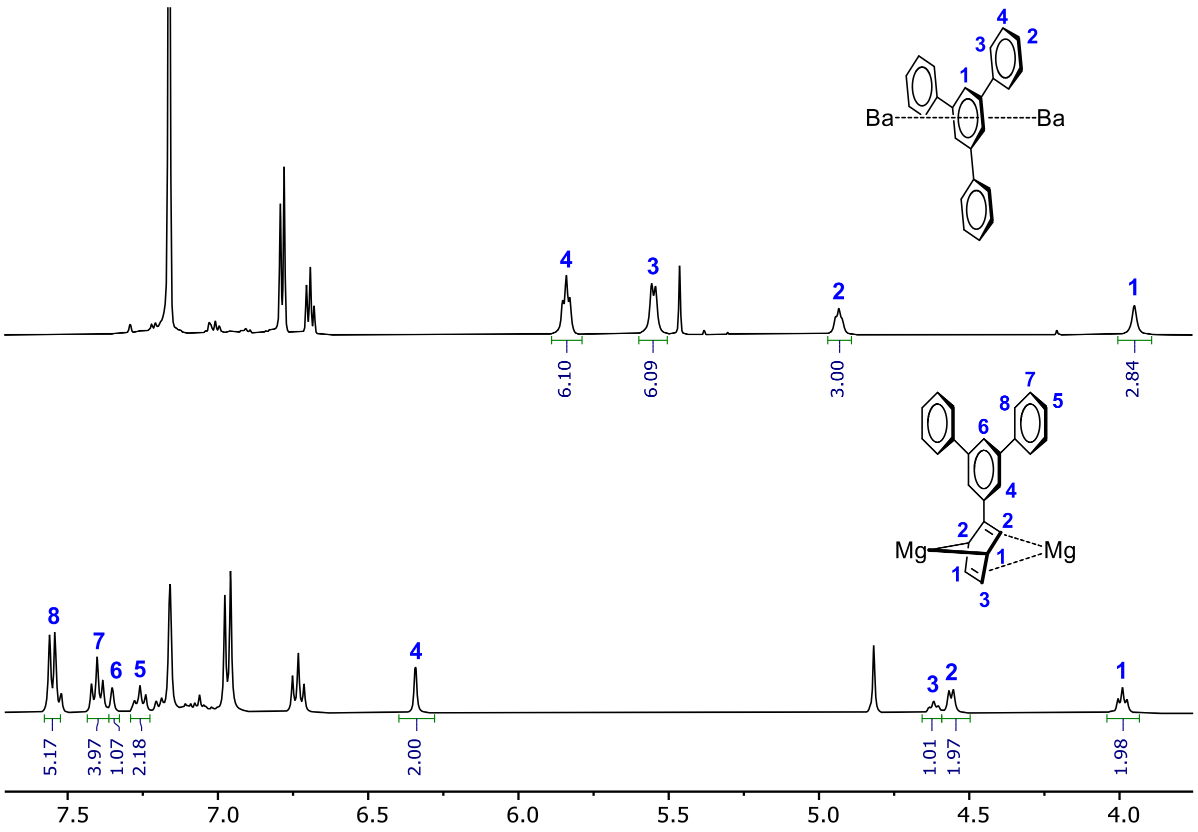

Figure S78. Fragments of ^{1}H NMR (600.13 MHz, 298 K, C_6D_6) of [{(^{DIPP}BDI)Mg}$_2$(η^2:η^4-1,3,5-$Ph_3C_6H_3$)] and [{(^{DIPP}BDI*)Ba}$_2$(η^2:η^4-1,3,5-$Ph_3C_6H_3$)] showing different symmetries of [1,3,5-$Ph_3C_6H_3$]$^{2-}$.

1.4 NMR spectra of Ae benzene compounds

Unusual values of BDI chemical shifts, *i.e*. down-field shifted CH backbone, up-field shifted DIPP/DIPeP aromatic proton signals and strong shifts of some *iso*-propyl CH_3 or 3-pentyl CH_2/CH_3 ^{1}H and ^{13}C signals, are observed in NMR spectra of Ae benzene complexes. Based on in-depth structural analysis, a possible explanation could be the hyperfine contribution to NMR chemical shifts, mainly pseudocontact effect, since the source of spin density is open shell benzene dianion isolated from ligand moieties.[S10] A simplified pseudocontact effect field shown on **Figure S79** corresponds to the observed trend of chemical shifts.[S11] Since the observed shifts are the largest for Ba homologues, the metal influence on resulting chemical shifts cannot be omitted and requires further study. A detailed investigation into magnetic ground state is in progress and will be reported in a separate study.

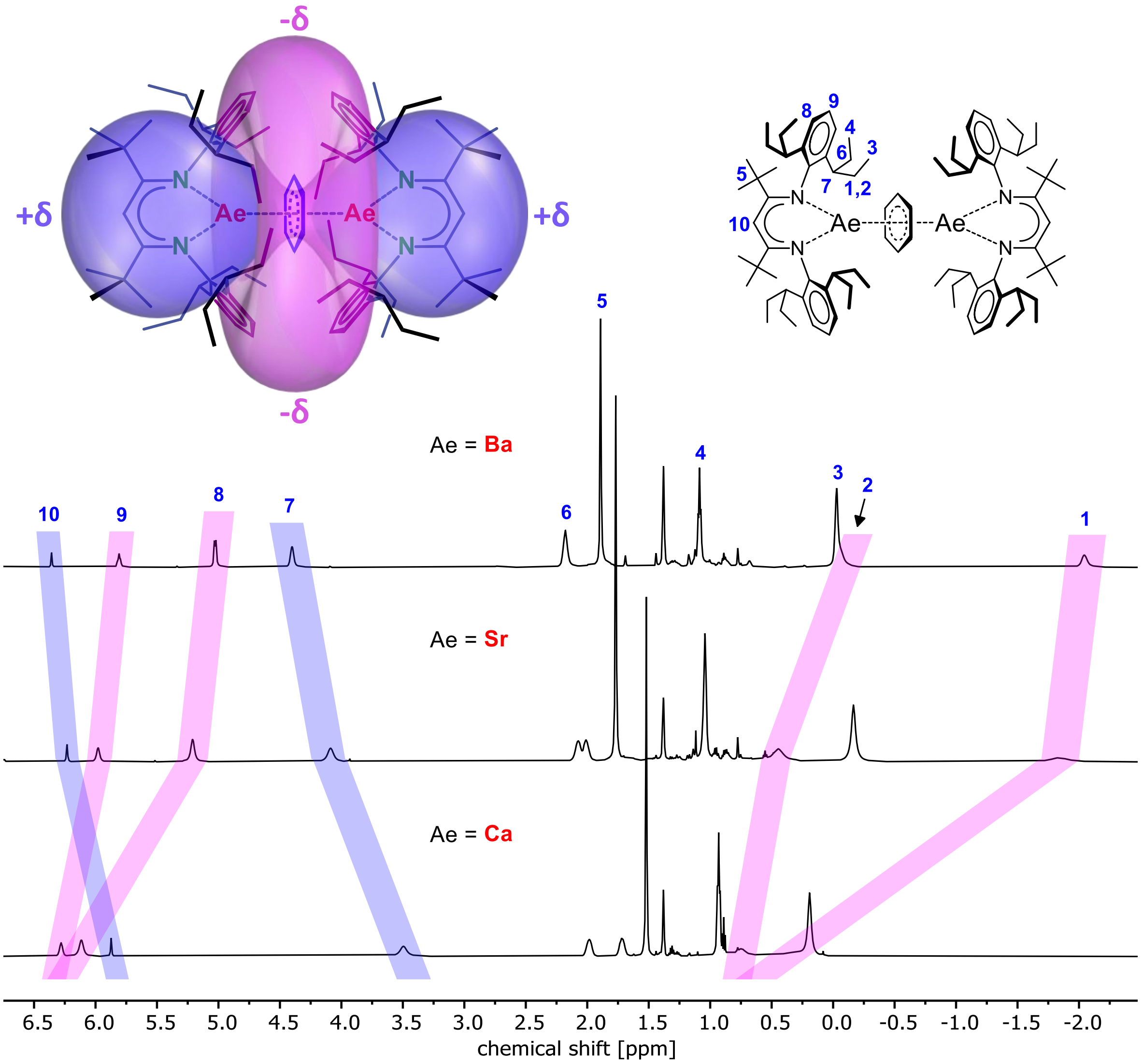

Figure S79. ^{1}H NMR spectra (600.13 MHz, 298 K, C_6D_{12}) [{(^{DIPeP}BDI*)Ae}$_2$(η^6:η^6-C_6H_6)] complexes showing the hyperfine contribution to the ligand chemical shifts following the pseudocontact effect field (positive shift in violet, negative shift in pink).

1.5 NMR Experiments

Reactivity/decomposition studies were performed using ^{1}H NMR spectroscopy. Solutions of [{(^{DIPeP}BDI*)Ba}$_2$(*η*^{6}:*η*^{6}-C_6H_6)] and [{(^{DIPP}BDI*)Ba}$_2$(*η*^{6}:*η*^{6}-C_6H_6)] samples were prepared in J.-Young tubes by dissolving *ca*. 5 mg in 0.4 – 0.5 mL of deuterated benzene, cyclohexane or methylcyclohexane. Reactants were added in appropriate stoichiometry. Prior to pressurize with 1.5 bar of H_2, samples were frozen in liquid nitrogen. Prior to addition of iodobenzene or (BDI*)Ae iodides, samples were cooled to -35 °C. Addition of polycyclic hydrocarbons to the samples was performed with prior cooling to -35 °C or without prior cooling.

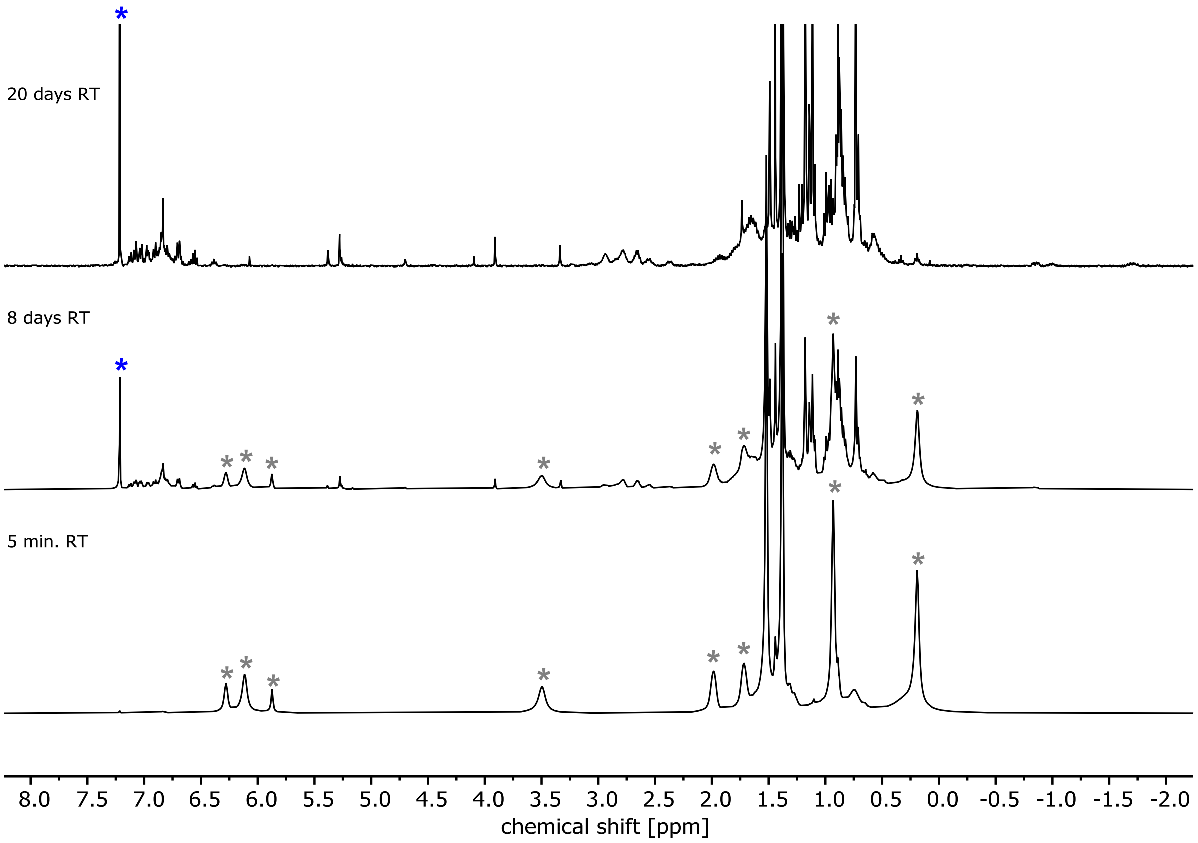

Figure S80. ^{1}H NMR (600.13 MHz, 298 K, C_6D_{12}) spectra showing full decomposition of [{(^{DIPeP}BDI*)Ca}$_2$(C_6H_6)] (grey) at RT over 20 days into free benzene (blue) and unidentified species.

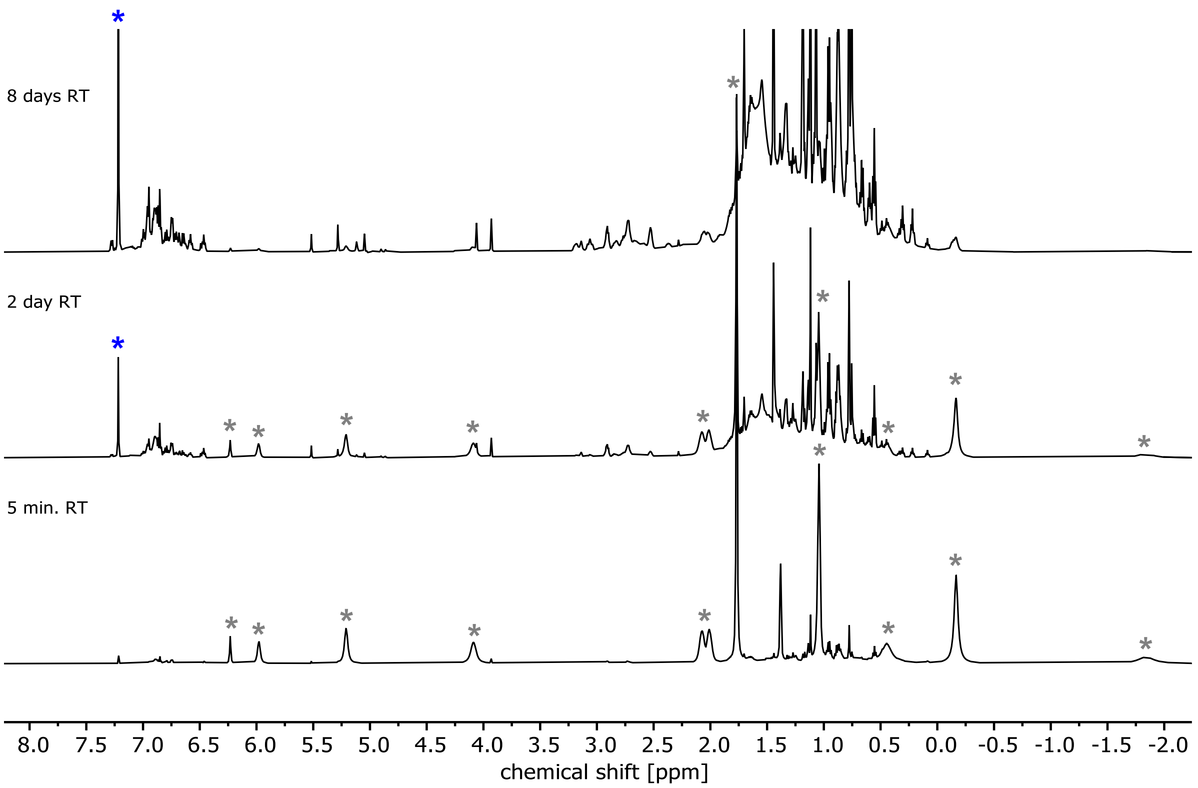

Figure S81. ^{1}H NMR (600.13 MHz, 298 K, C_6D_{12}) spectra showing full decomposition of [{(^{DIPeP}BDI*)Sr}$_2$(C_6H_6)] (grey) at RT over 8 days into free molecule (blue) and unidentified species.

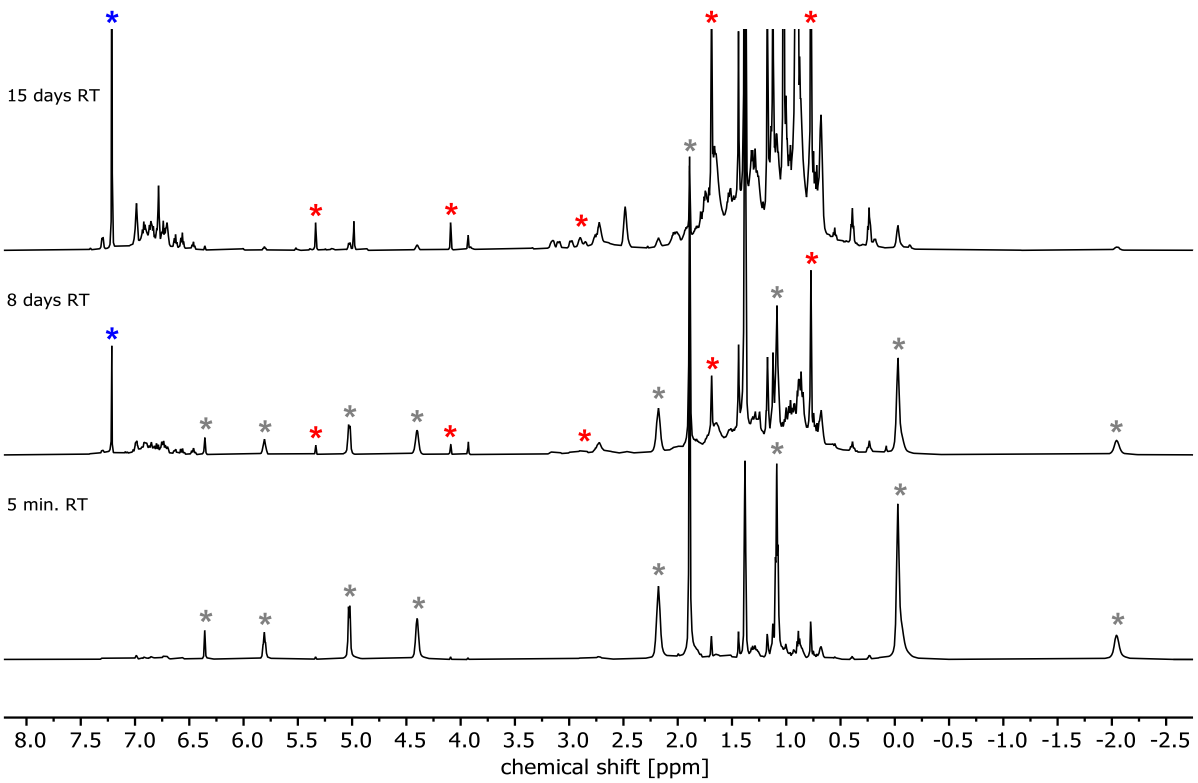

Figure S82. ^{1}H NMR (600.13 MHz, 298 K, C_6D_{12}) spectra showing full decomposition of [{(^{DIPeP}BDI*)Ba}$_2$(C_6H_6)] (grey) at RT over 15 days into free molecule (blue), homoleptic complex (red) and unidentified species.

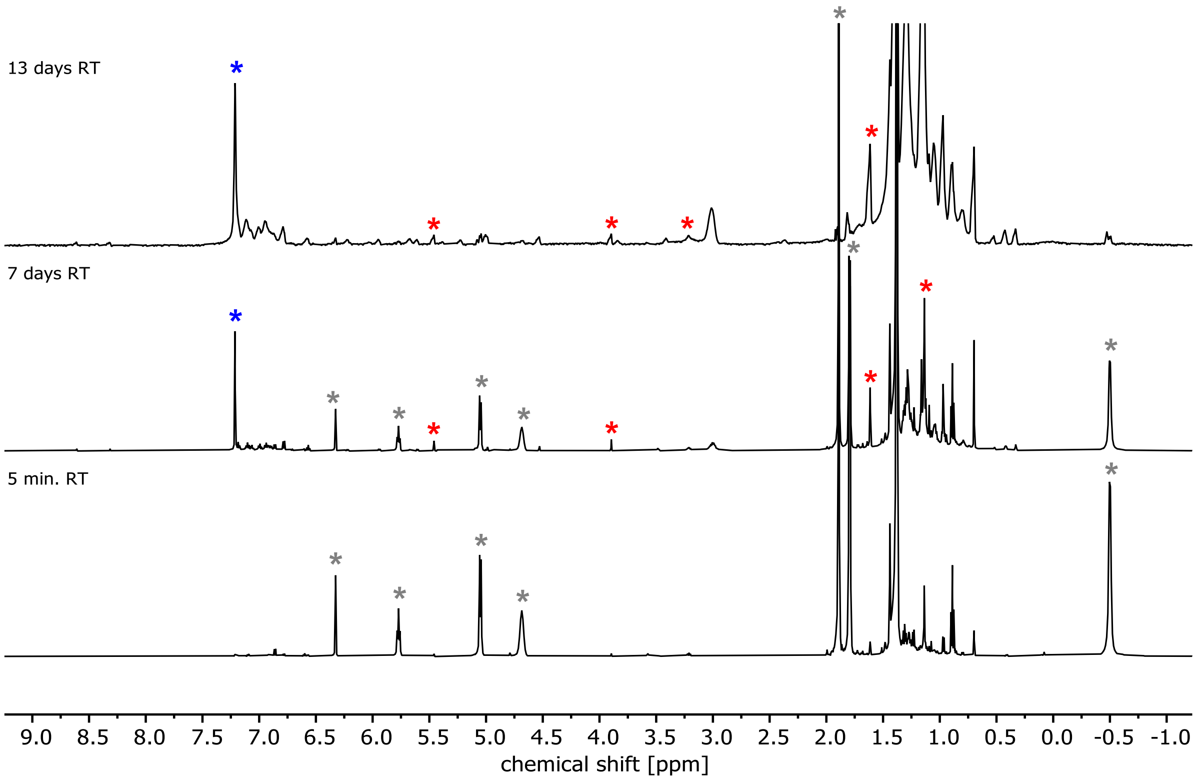

Figure S83. ^{1}H NMR (600.13 MHz, 298 K, C_6D_{12}) spectra showing full decomposition of [{(^{DIPP}BDI*)Ba}$_2$(C_6H_6)] (grey) at RT over 13 days into free molecule (blue), homoleptic complex (red) and unidentified species.

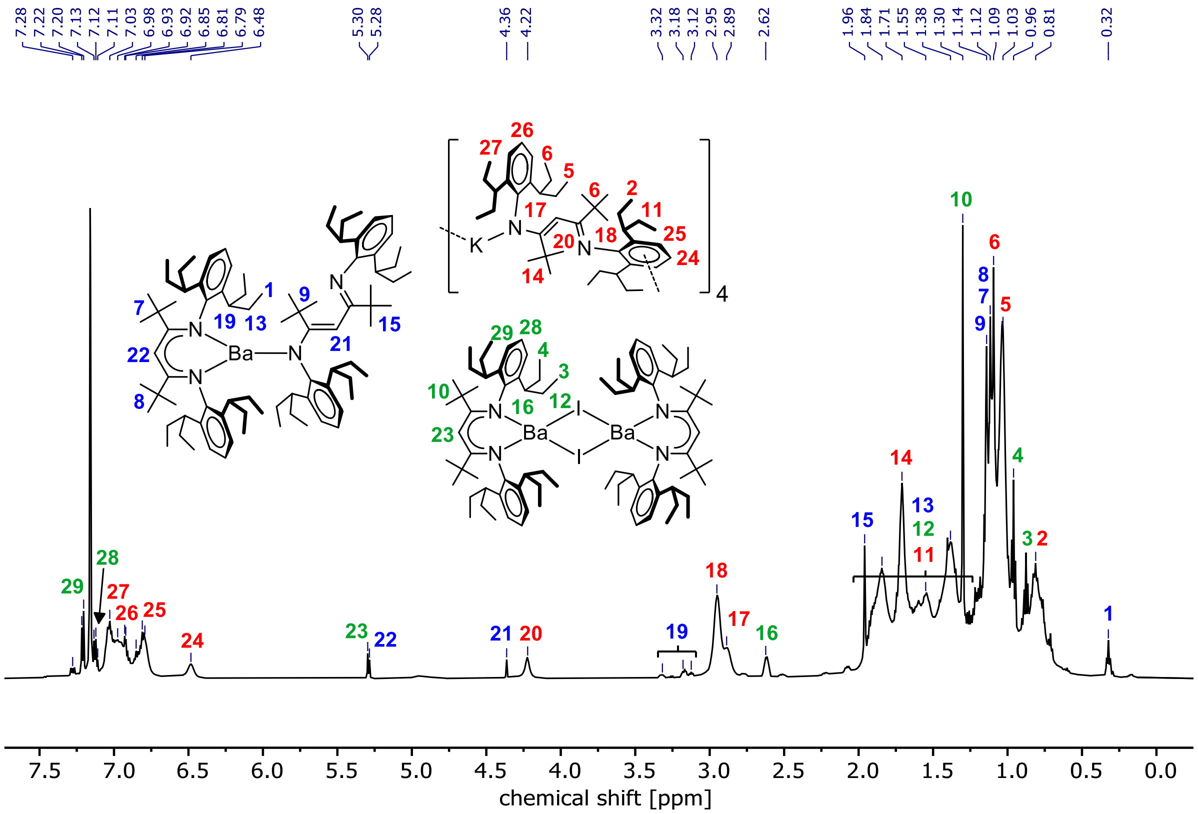

Figure S84. ^{1}H NMR (600.13 MHz, 298 K, C_6D_{12}) showing a mixture of [(κ^2,κ^1-^{DIPeP}BDI*)$_2$Ba] (blue) and [{(^{DIPeP}BDI*)Ba(μ-I)}$_2$ (green) obtained in the reaction of [{(^{DIPeP}BDI*)K]$_4$ (red) with BaI_2 (2 eq.)

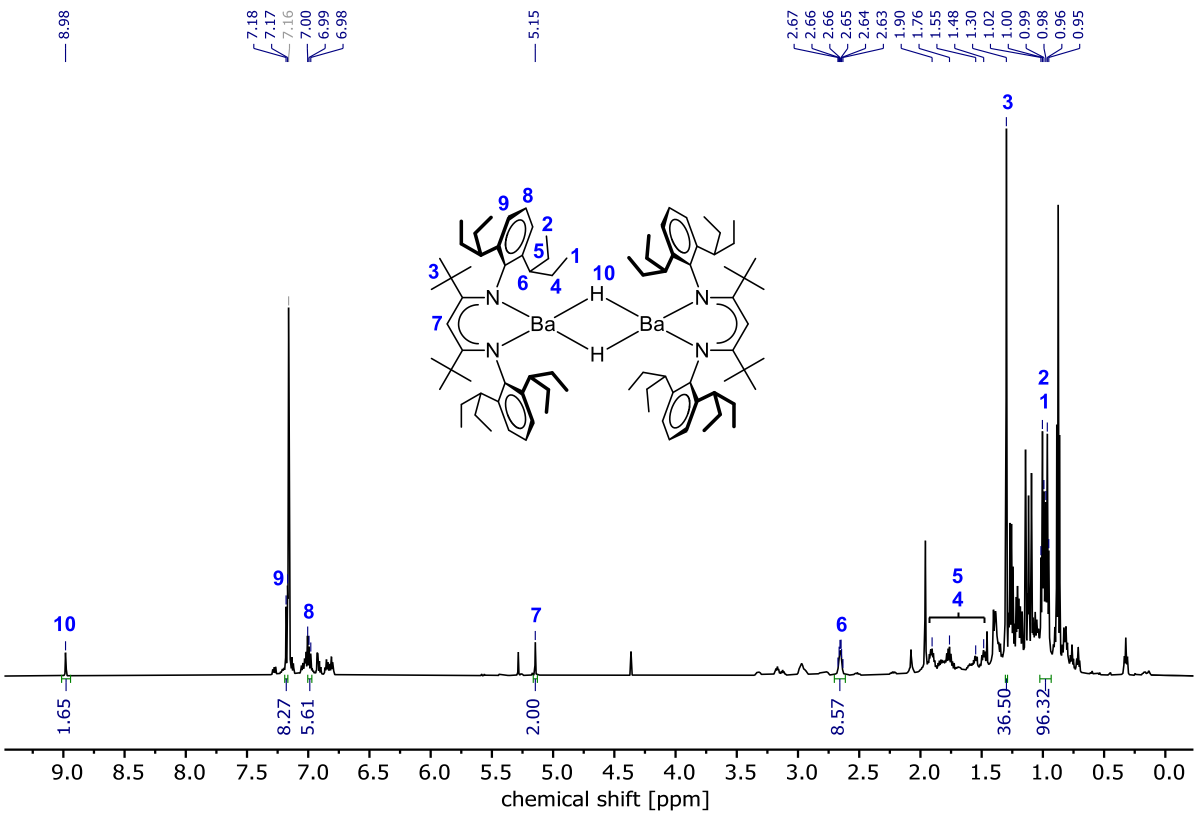

Figure S85. ^{1}H NMR (600.13 MHz, 298 K, C_6D_6) of [(^{DIPeP}BDI*)Ba(μ-H)]$_2$ generated *in situ* from the frozen solution of [{(^{DIPeP}BDI*)Ba}$_2$(η^6:η^6-C_6H_6)] pressurised with 1.5 bar of H_2.

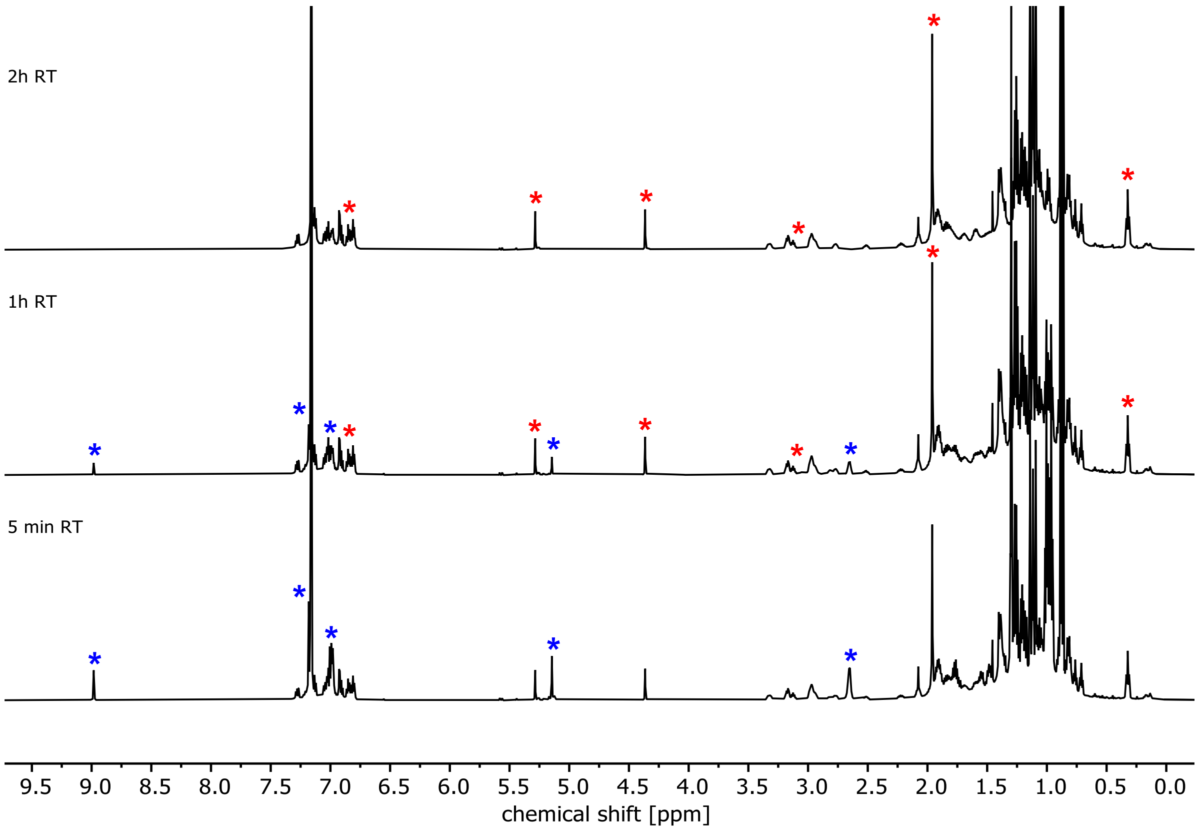

Figure S86. ^{1}H NMR (600.13 MHz, 298 K, C_6D_6) spectra showing the decomposition of *in situ* generated [(^{DIPeP}BDI*)Ba(μ-H)]$_2$ into [(η^2,η^1-^{DIPeP}BDI*)$_2$Ba] over 2h.

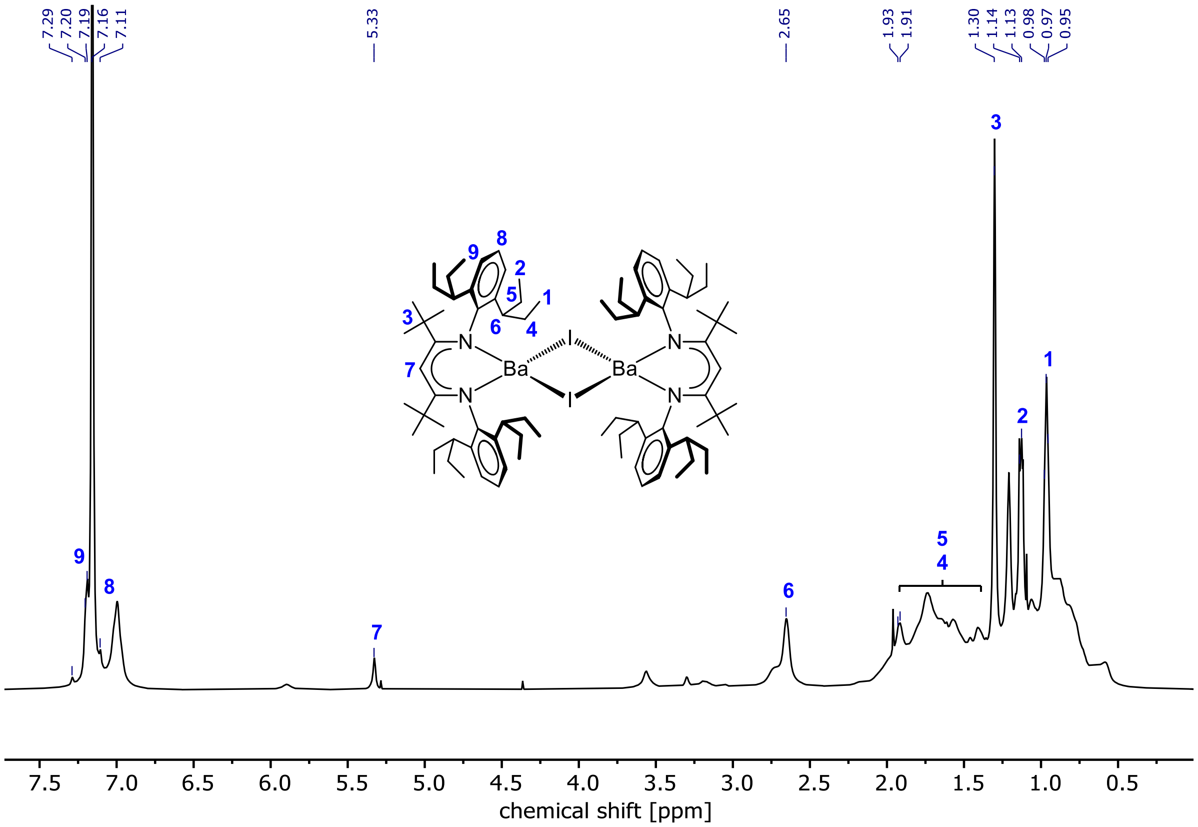

Figure S87. ^{1}H NMR (600.13 MHz, 298 K, C_6D_6) of [(^{DIPeP}BDI*)Ba(μ-I)]$_2$ formed after addition of iodine to the frozen solution of [{(^{DIPeP}BDI*)Ba}$_2$(η^6:η^6-C_6H_6)].

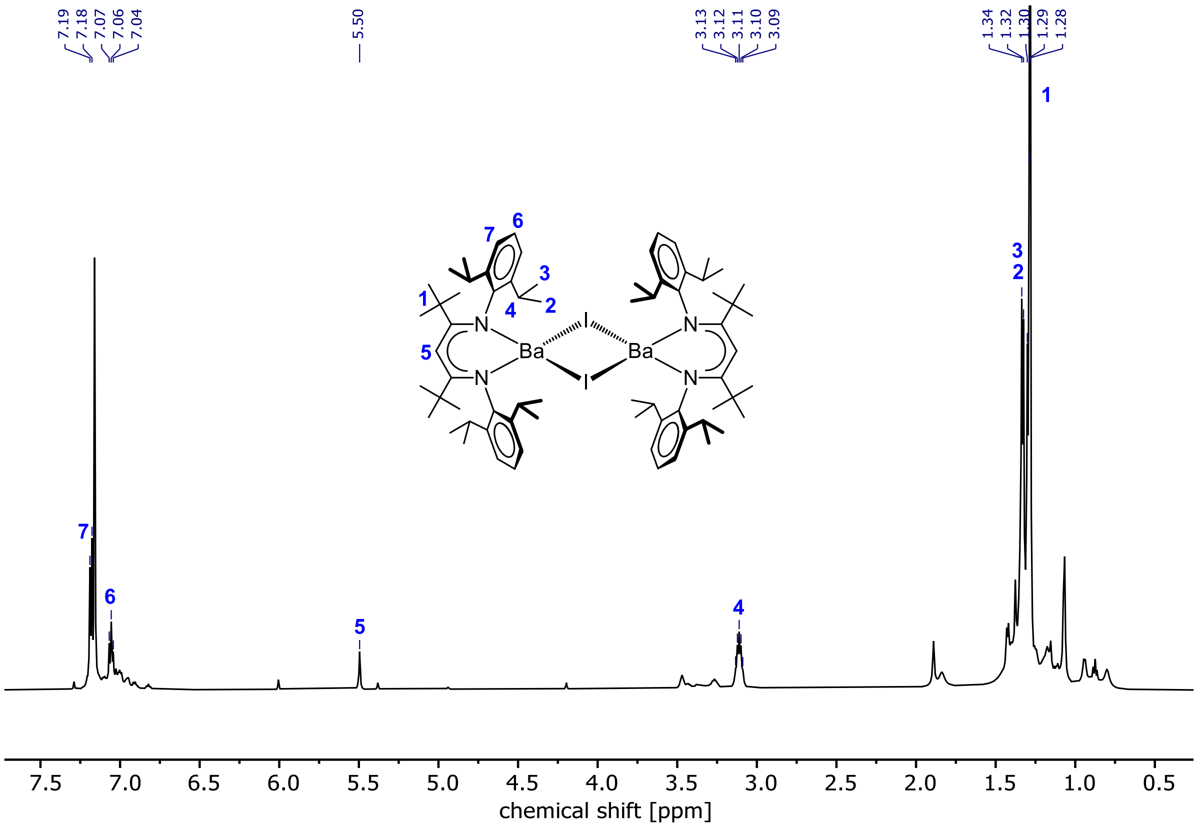

Figure S88. ^{1}H NMR (600.13 MHz, 298 K, C_6D_6) of [(^{DIPP}BDI*)Ba(μ-I)]$_2$ formed after addition of iodine to the frozen solution of [{(^{DIPP}BDI*)Ba}$_2$(η^6:η^6-C_6H_6)].

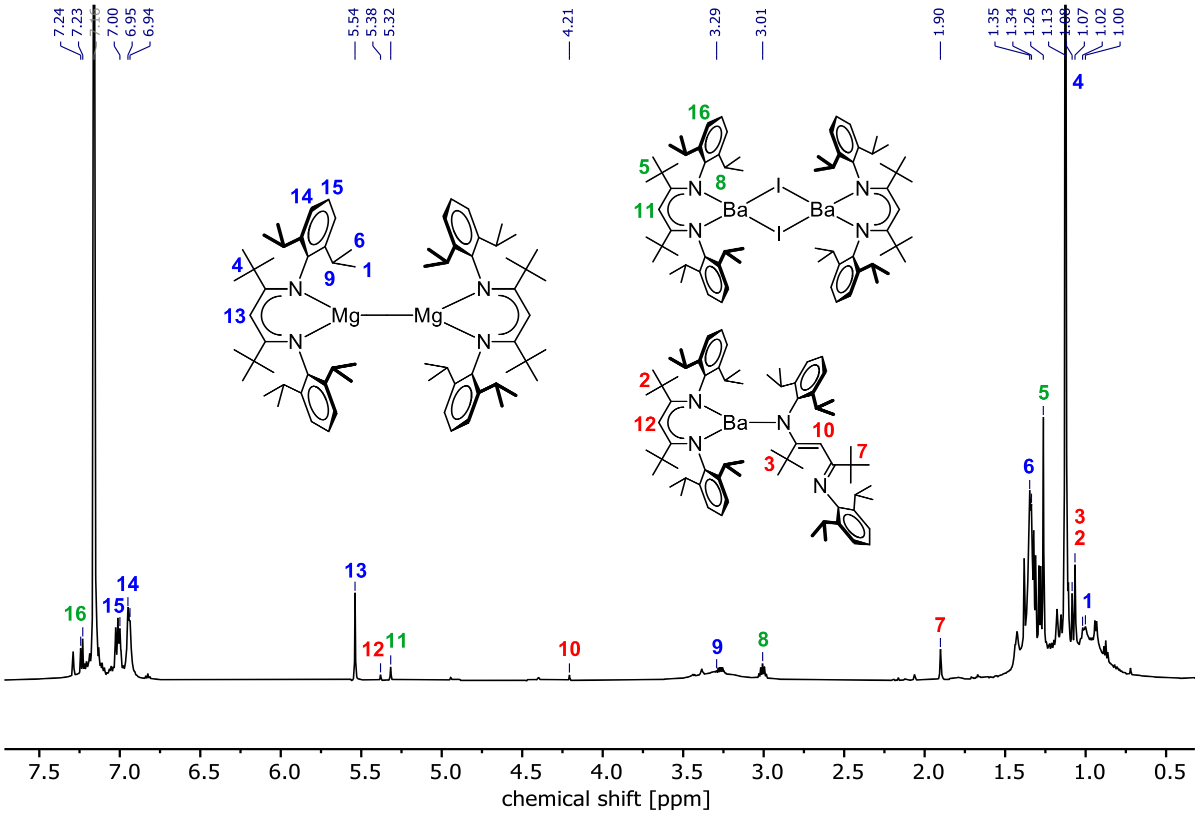

Figure S89. ^{1}H NMR (600.13 MHz, 298 K, C_6D_6) of [(^{DIPP}BDI*)Mg]$_2$ (blue) and [(^{DIPP}BDI*)Ba(μ-I)]$_2$ (green) obtained in reduction of [(^{DIPP}BDI*)Mg(μ-I)]$_2$ by [{(^{DIPP}BDI*)Ba}$_2$(η^6:η^6-C_6H_6)] in 1:1 ratio.

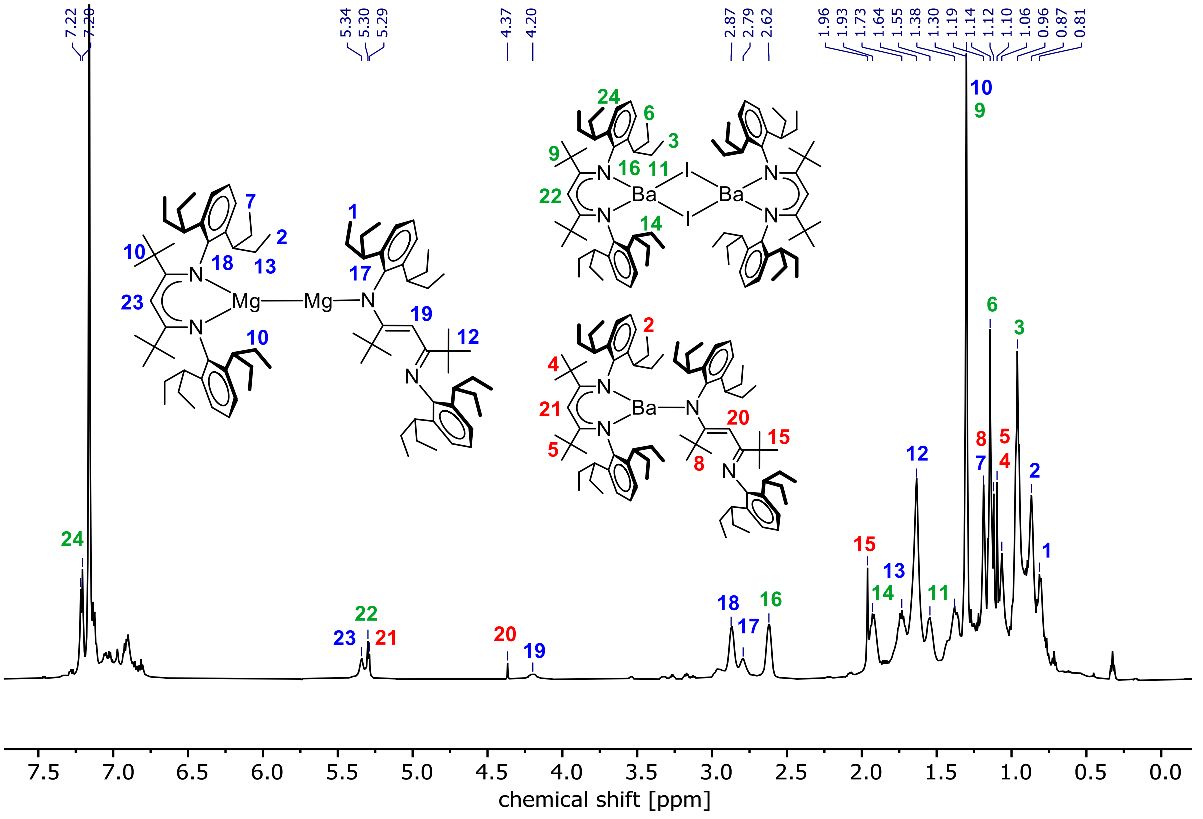

Figure S90. ^{1}H NMR (600.13 MHz, 298 K, C_6D_6) of [{(^{DIPeP}BDI*)Ca}$_2$(η^6:η^6-C_6H_6)] and [(^{DIPeP}BDI*)Ba(μ-I)]$_2$ obtained in reaction of [(^{DIPeP}BDI*)Ca(μ-I)]$_2$ with [{(^{DIPeP}BDI*)Ba}$_2$(η^6:η^6-C_6H_6)] in 1:1 ratio.

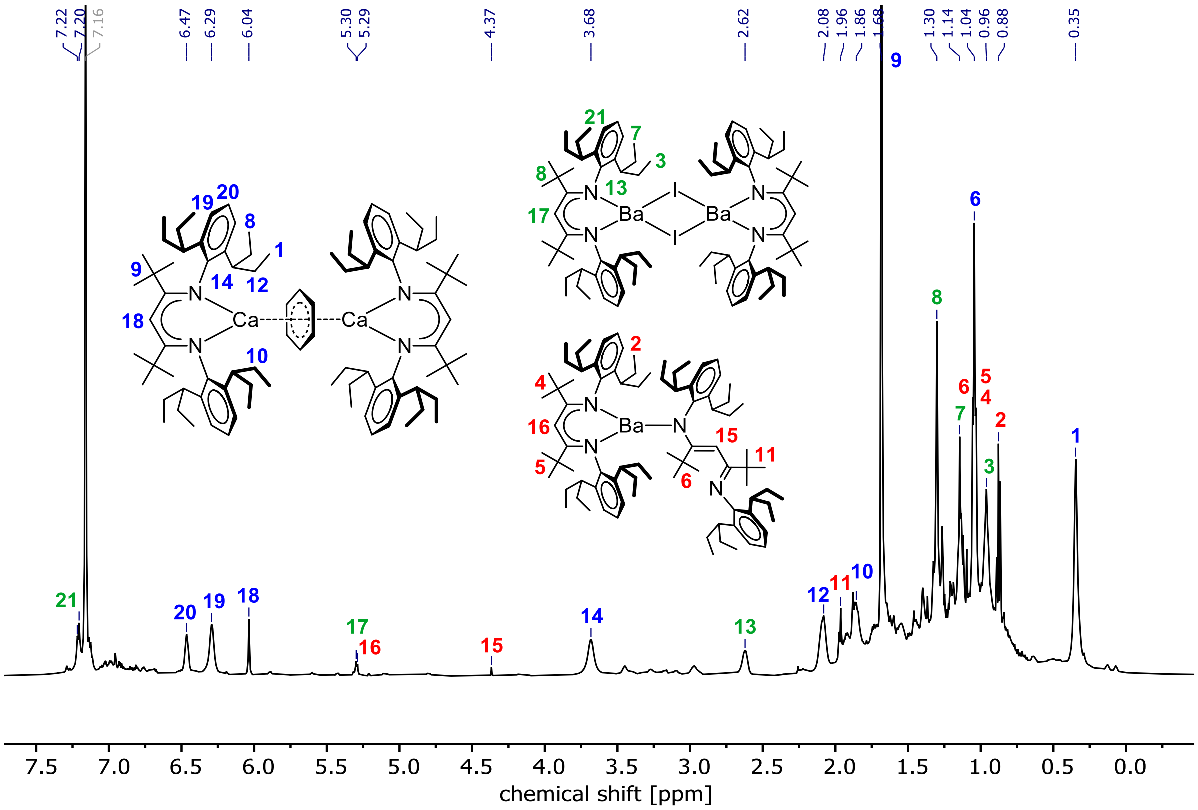

Figure S91. ^{1}H NMR (600.13 MHz, 298 K, C_6D_6) of [{(^{DIPeP}BDI*)Ca}$_2$(η^6:η^6-C_6H_6)] and [(^{DIPeP}BDI*)Ba(μ-I)]$_2$ obtained in reaction of [(^{DIPeP}BDI*)Ca(μ-I)]$_2$ with [{(^{DIPeP}BDI*)Ba}$_2$(η^6:η^6-C_6H_6)] in 1:1 ratio.

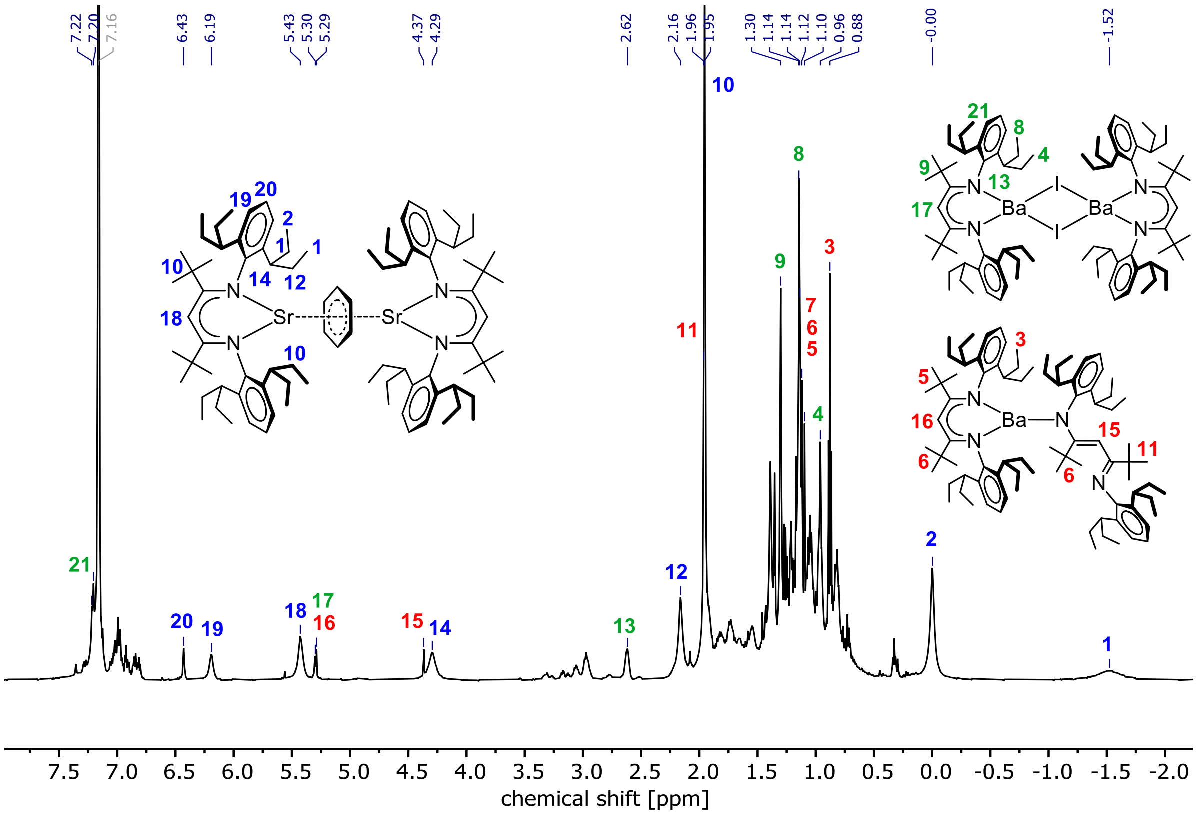

Figure S92. ^{1}H NMR (600.13 MHz, 298 K, C_6D_6) of [{(^{DIPeP}BDI*)Sr}$_2$(η^6:η^6-C_6H_6)] and [(^{DIPeP}BDI*)Ba(μ-I)]$_2$ obtained in reaction of [(^{DIPeP}BDI*)Ca(μ-I)]$_2$ with [{(^{DIPeP}BDI*)Ba}$_2$(η^6:η^6-C_6H_6)] in 1:1 ratio.

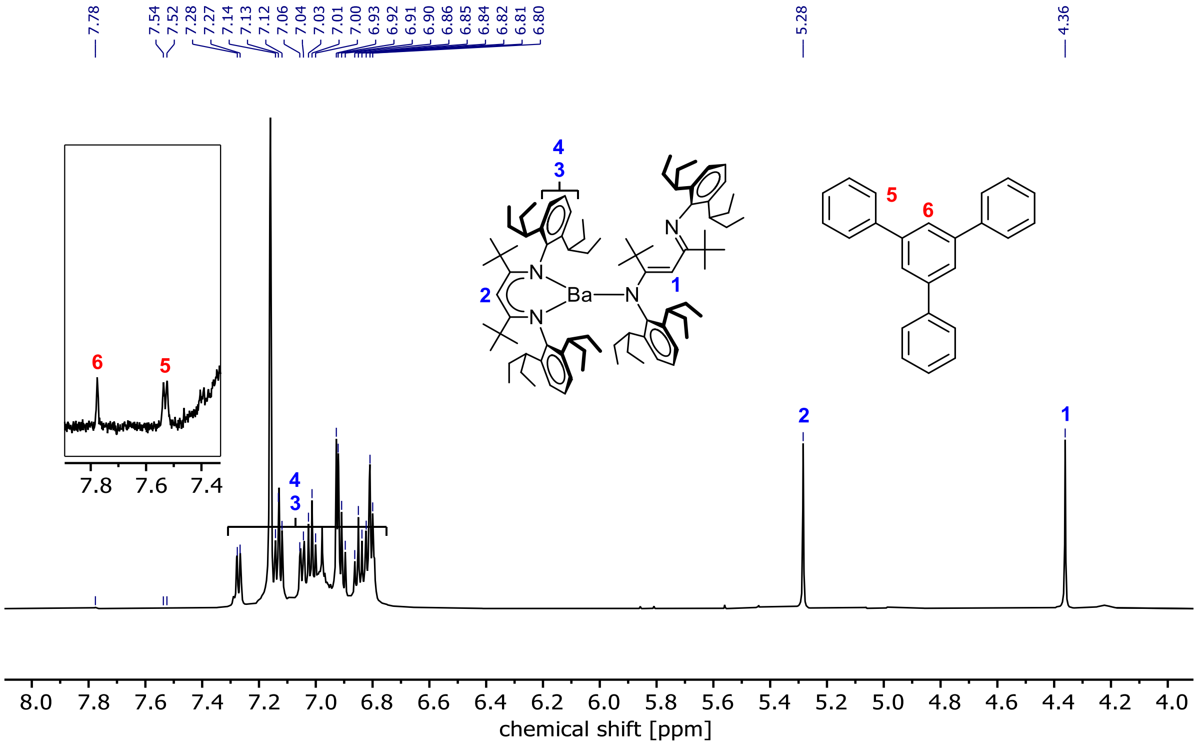

Figure S93. ^{1}H NMR (600.13 MHz, 298 K, C_6D_6) of [(^{DIPeP}BDI*)$_2$Ba] formed after addition of 1,3,5-$Ph_3C_6H_3$ to the cooled solution of [{(^{DIPeP}BDI*)Ba}$_2$(η^6:η^6-C_6H_6)]. Free residual 1,3,5-$Ph_3C_6H_3$ marked in red.

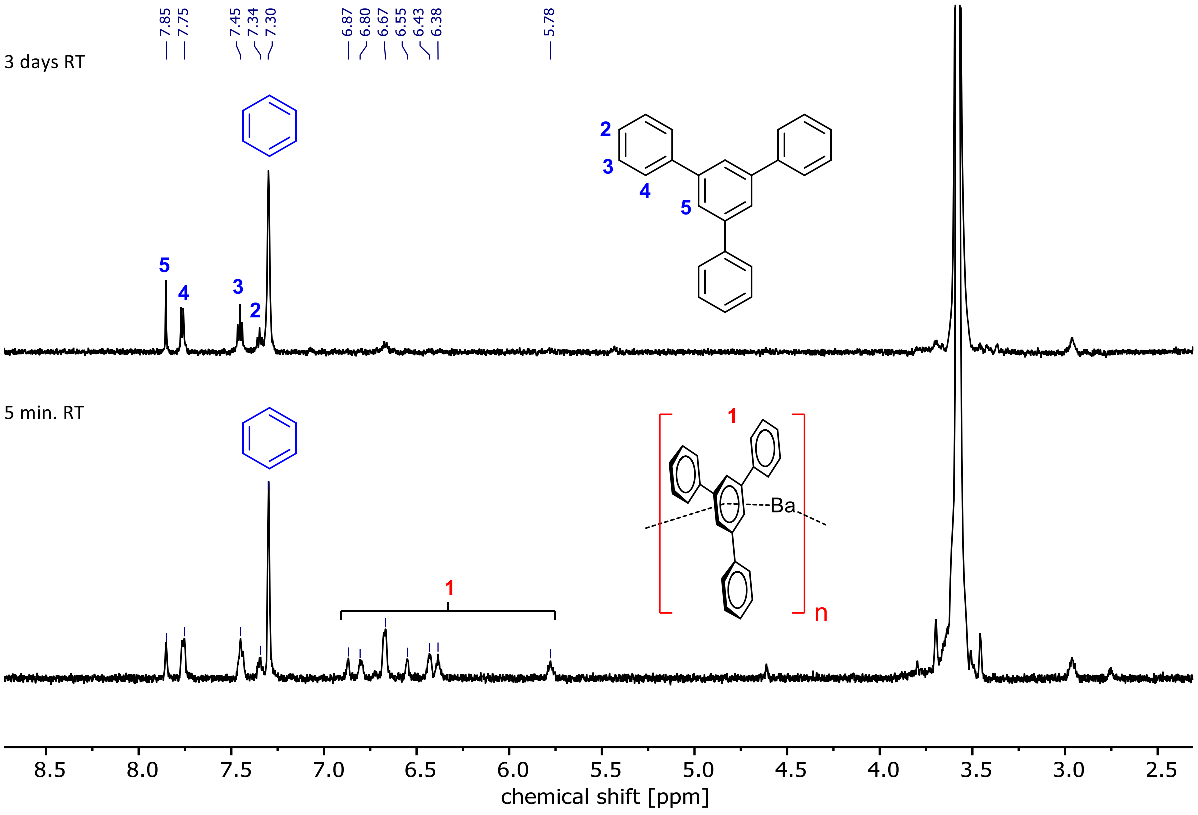

Figure S94. ^{1}H NMR (600.13 MHz, 298 K, THF-d_8) spectra of the dark mirror deposited after addition of 1,3,5-$Ph_3C_6H_3$ to the cooled solution of [{(^{DIPeP}BDI*)Ba}$_2$(η^6:η^6-C_6H_6)], showing decomposition of the proposed side-product [Ba(1,3,5-$Ph_3C_6H_3$)]$_n$ (red) into free 1,3,5-$Ph_3C_6H_3$ (blue) and an undefined Ba species over 3 days.

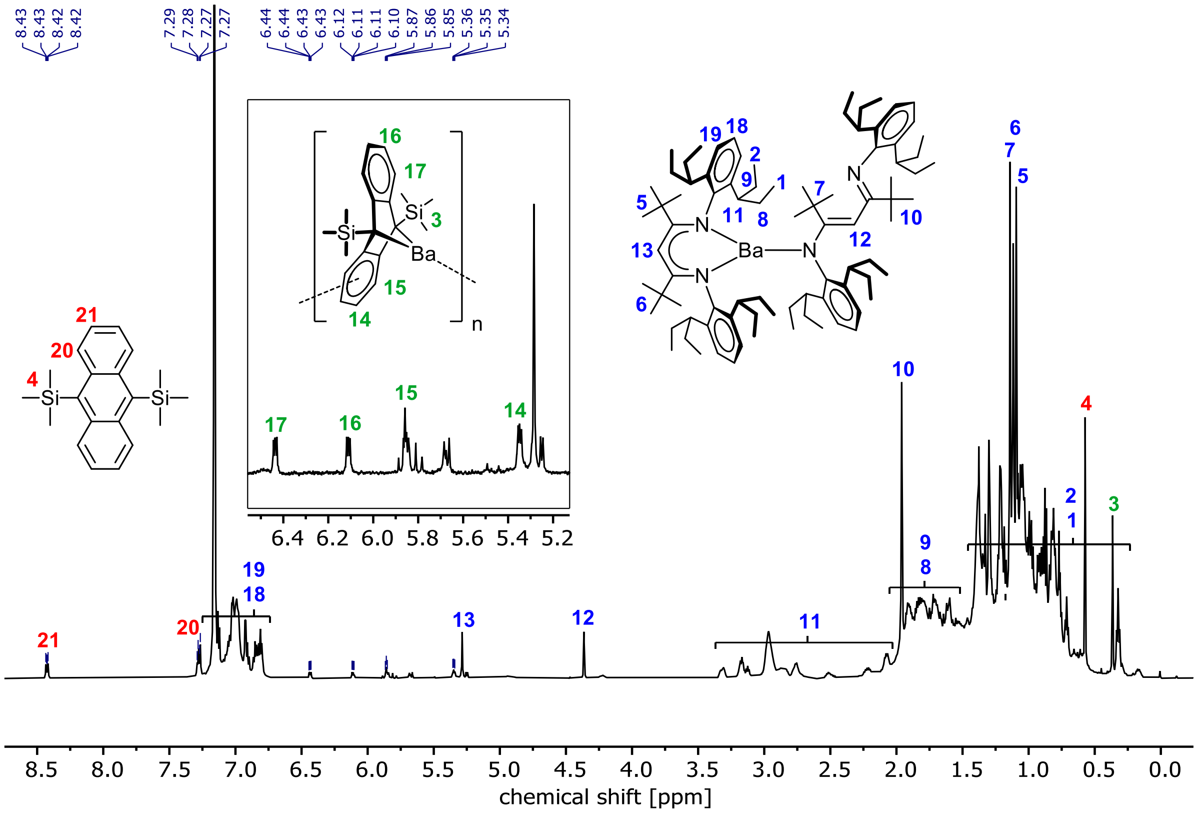

Figure S95. ^{1}H NMR (600.13 MHz, 298 K, C_6D_6) of [(^{DIPeP}BDI*)$_2$Ba] (blue) and [Ba(*bis*(trimethylsilyl)anthracene)]$_n$ (green) formed after addition of *bis*(trimethylsilyl)anthracene (red) to the cooled solution of [{(^{DIPeP}BDI*)Ba}$_2$(η^6:η^6-C_6H_6)].

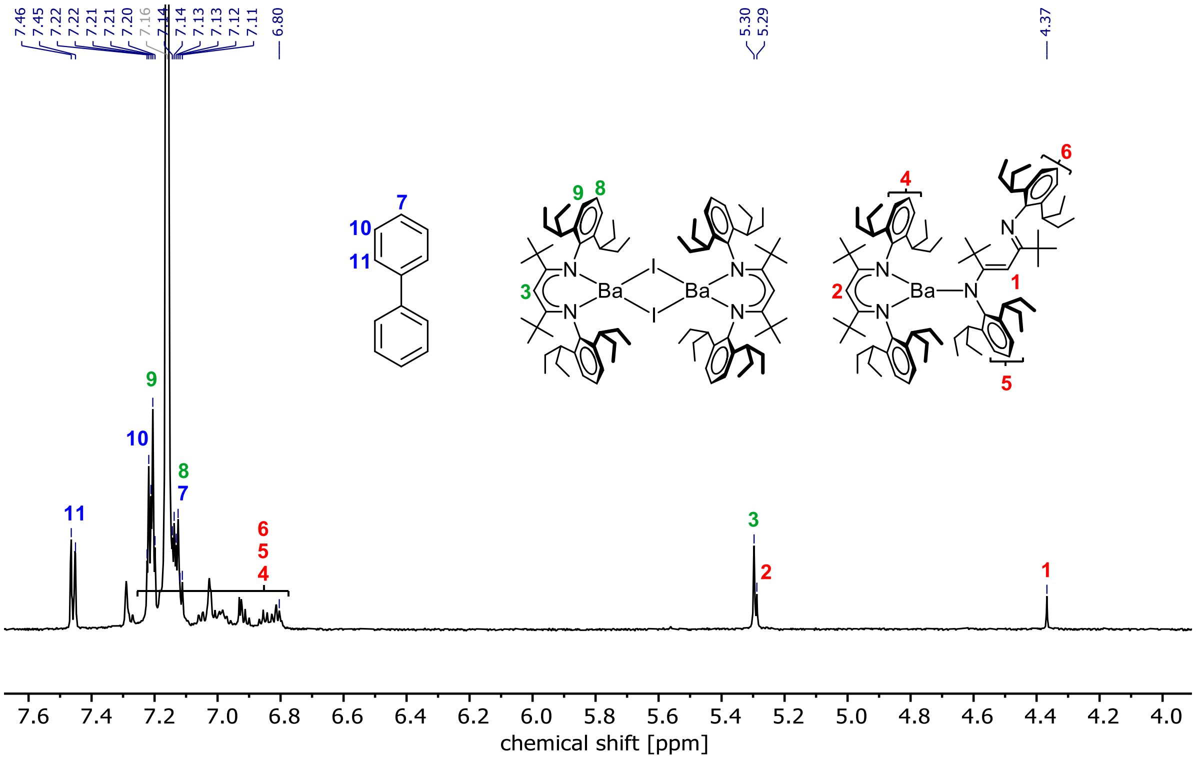

Figure S96. Fragment of ^{1}H NMR (600.13 MHz, 298 K, C_6D_6) spectrum showing biphenyl and [(^{DIPeP}BDI*)Ba(μ-I)]$_2$ formation upon adding 2 equiv. of iodobenzene to [{(^{DIPeP}BDI*)Ba}$_2$(η^6:η^6-C_6H_6)] solution.

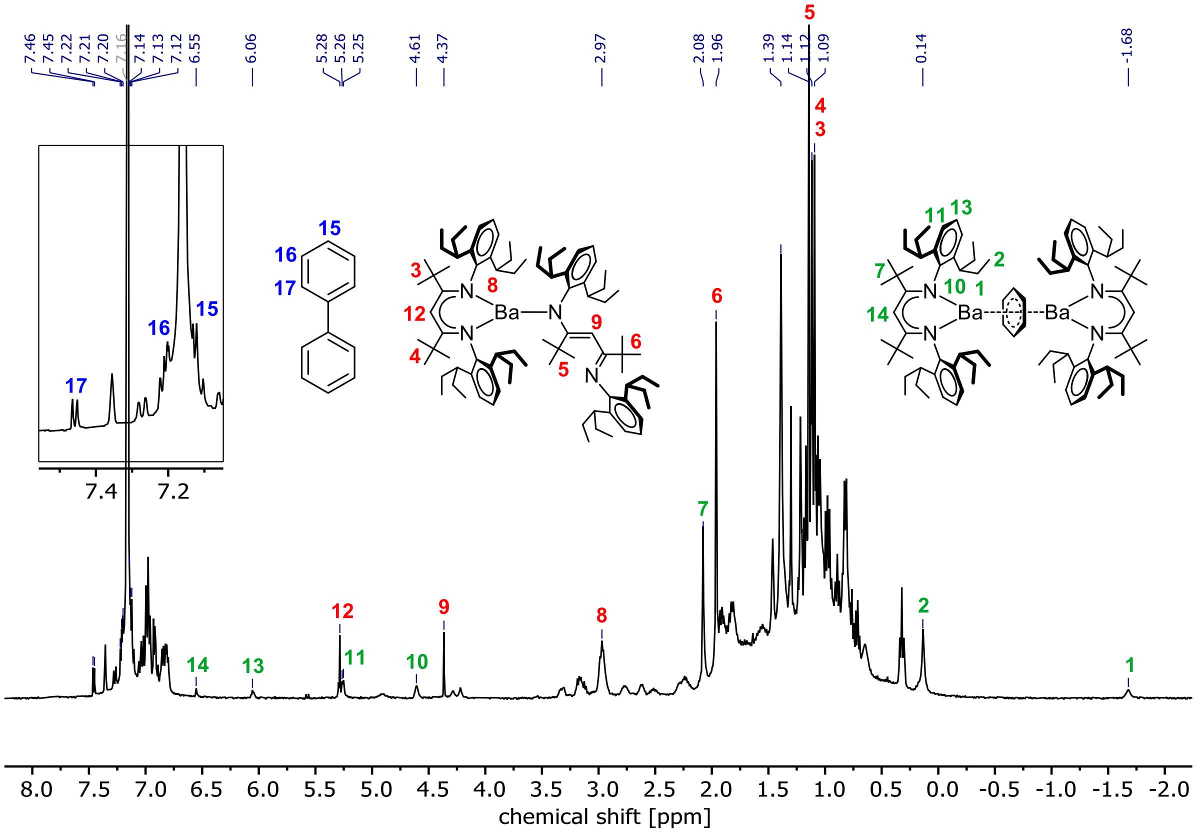

Figure S97. ^{1}H NMR (600.13 MHz, 298 K, C_6D_6) of the sample obtained after sublimation of the frozen [{(^{DIPP}BDI*)Ba}$_2$(η^6:η^6-C_6H_6)] benzene solution at -15 °C, showing [{(^{DIPP}BDI*)Ba}$_2$(η^6:η^6-C_6H_6)] (green) decomposition into biphenyl (blue) and [(^{DIPeP}BDI*)$_2$Ba] (red).

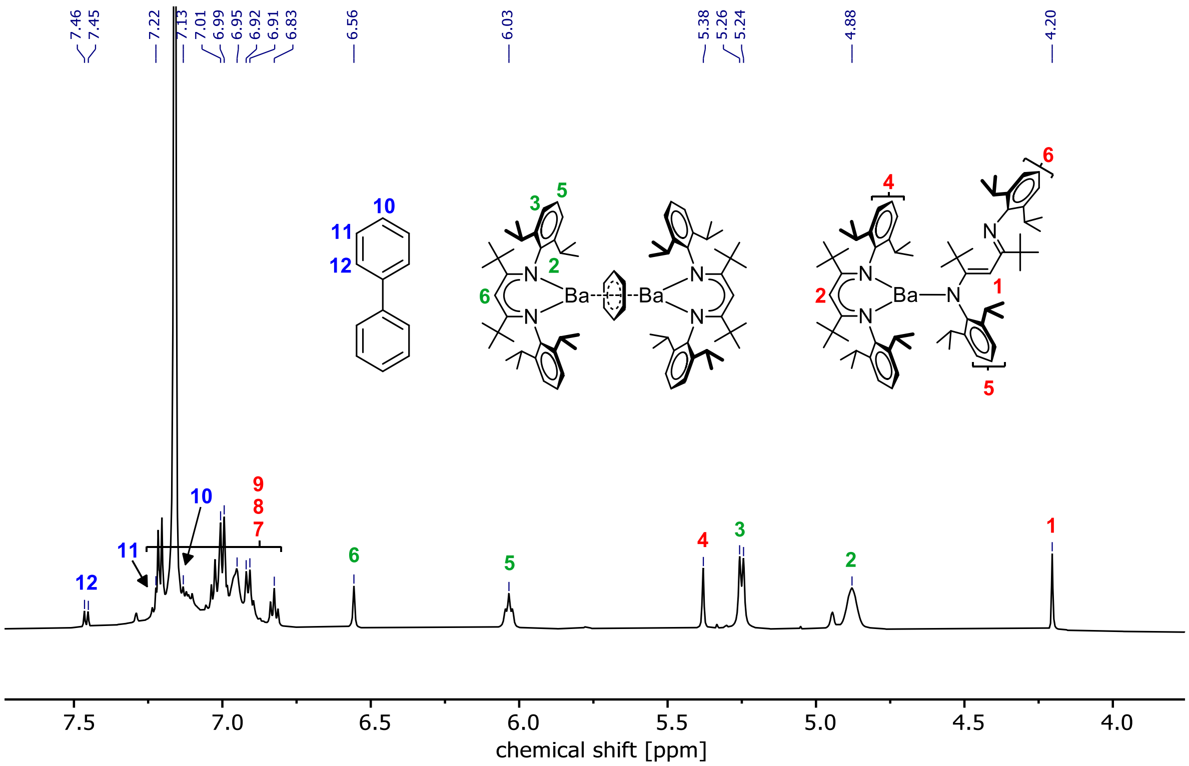

Figure S98. Fragment of ^{1}H NMR (600.13 MHz, 298 K, C_6D_6) spectrum showing biphenyl formation in the benzene solution of [{(^{DIPP}BDI*)Ba}$_2$(η^6:η^6-C_6H_6)] after 1 days at RT.

1.6 Crystal Structure Determination

Suitable single crystals of compounds [(^{DIPeP}BDI*)K]$_4$, [(^{DIPeP}BDI*)Ca(μ-I)]$_2$, [(^{DIPeP}BDI*)CaI·THF], [(^{DIPeP}BDI*)Sr(μ-I)]$_2$, [(^{DIPeP}BDI*)Ba(μ-I)]$_2$, [{(^{DIPeP}BDI*)Ca}$_2$(η^6:η^6-C_6H_6)], [{(^{DIPeP}BDI*)Sr}$_2$(η^6:η^6-C_6H_6)], [{(^{DIPeP}BDI*)Ba}$_2$(η^6:η^6-C_6H_6)], [{(^{DIPP}BDI*)Ba}$_2$(η^6:η^6-$C_{16}H_{10}$)] were embedded in protective perfluoropolyalkylether oil (viscosity 1800 cSt; ABCR GmbH) on a microscope slide and a single specimen was selected and subsequently transferred to the cold nitrogen gas stream of the diffractometer.

The intensity data was collected at 100 K using CuK_α radiation (λ = 1.54184 Å) on an Agilent SuperNova dual radiation diffractometer with microfocus X-ray sources and mirror optics. The measured data were processed with the CrysAlisPro software package.[S12] Data were corrected for Lorentz and polarisation effects, and an empirical absorption correction using spherical harmonics was performed. In most cases, either an additional numerical absorption correction based on gaussian integration over a multifaceted crystal model (compounds [(^{DIPeP}BDI*)K]$_4$, [(^{DIPeP}BDI*)Ca(μ-I)]$_2$, [(^{DIPeP}BDI*)Sr(μ-I)]$_2$, [(^{DIPeP}BDI*)Ba(μ-I)]$_2$ and [{(^{DIPeP}BDI*)Sr}$_2$(η^6:η^6-C_6H_6)]) or an analytical absorption correction[S13] ([(^{DIPeP}BDI*)CaI·THF], [{(^{DIPeP}BDI*)Ca}$_2$(η^6:η^6-C_6H_6)], [{(^{DIPeP}BDI*)Ba}$_2$(η^6:η^6-C_6H_6)], [{(^{DIPP}BDI*)Ba}$_2$(η^6:η^6-$C_{16}H_{10}$)] were also applied. Using Olex2,[S14] the structures were solved by dual-space methods (SHELXT)[S15] and refined by full-matrix least-squares procedures on F^2 using SHELXL.[S16] All non-hydrogen atoms were refined with anisotropic displacement parameters. Most H-atoms were placed in geometrically calculated positions and refined by using a riding model where each H-atom was assigned a fixed isotropic displacement parameter with a value equal to 1.2U_{eq} (CH or CH_2) or 1.5U_{eq} (CH_3) of its parent C-atom.

Disordered fragments of the structures were modeled using similarity restraints (SADI, SAME, SIMU), geometric restraints (DANG, FLAT, AFIX), rigid bond restraints (RIGU) or isotropic restrains (ISOR),[S17] while the heavily disordered/unidentified solvent contribution to the structure factors was secured by back-Fourier transformation using the solvent mask routine[S18] of the program Olex2,[S13] as stated below:

[(^{DIPeP}BDI*)K]$_4$ – Significant disorder affecting one potassium atom, a N-DIPeP moiety, two more DIPeP groups and six additional 3-pentyl moieties. The disorder was modeled using SADI, SIMU, DANG, FLAT and RIGU restraints. The relative occupancies of the two alternative orientations of each group were refined to 0.802(3)/0.198(3) (K + N-DIPeP + DIPeP 1), 0.805(3)/0.195(3) (DIPeP 2), 0.638(6)/0.362(6) (3-pentyl 1), 0.646(6)/0.354(6) (3-pentyl 2),

0.669(4)/0.331(4) (3-Pentyl 3), 0.765(4)/0.235(4) (3-pentyl 4), 0.818(5)/0.182(5) (3-pentyl 5) and 0.770(6)/0.230(6) (3-pentyl 6), respectively. Co-crystallised benzene and *n*-hexane were the main occupants of the solvent cavities, with a significant contribution of other, unidentified molecules (an isomeric mixture of hexanes + benzene + small amounts of THF were used for crystallisation). Therefore, the solvent contribution to the structure factors was secured by back-Fourier transformation using the solvent mask routine· The solvent accessible voids treated this way had a size of 978.0 Å^3 (11.2% of the unit cell) and contained 188.5 electrons/unit cell.

[(^{DIPeP}BDI*)Ca(μ-I)]$_2$ – Disorder affecting one DIPeP group and the co-crystallised benzene solvent. The disorder was modeled using SADI, SIMU, FLAT and RIGU restains. Site occupancy factors of 0.511(4) and 0.489(4) were observed for the two alternative orientations of the DIPeP moiety. The benzene moiety was disordered about a two-fold rotation axis and its site occupancy factor was therefore constrained to 0.5. The ligand backbone hydrogen atom H2 deviated significantly from the position calculated *via* the riding model. This H atom was placed as indicated by a difference electron density map and its position was refined together with an isotropic displacement parameter.

[(^{DIPeP}BDI*)CaI·THF] – Disorder affecting one *tert*-butyl and two 3-pentyl groups was modeled using SIMU restrains. The relative occupancies of the two alternative orientations of each group were refined to 0.74(1)/0.26(1) (*tert*-butyl), 0.518(7)/0.482(7) (3-pentyl 1) and 0.902(7)/0.098(7) (3-pentyl 2). Isotropic restrain (ISOR) was used on atom C41A. The ligand backbone hydrogen atom H2 deviated significantly from the position calculated *via* the riding model. This H atom was placed as indicated by a difference electron density map and its position was refined together with an isotropic displacement parameter.

[(^{DIPeP}BDI*)Ba(μ-I)]$_2$ – Disorder of co-crystallised solvent. Since it was not clear, which solvents were present (a mixture of benzene, hexanes (isomeric mixture) and THF was used for crystallisation), their contribution to the structure factors was secured by back-Fourier transformation using the solvent mask routine. The solvent accessible voids treated this way had a size of 2255.6 Å^3 (23.0% of the unit cell) and contained 378.8 electrons/unit cell.

[{(^{DIPeP}BDI*)Ca}$_2$(η^6:η^6-C_6H_6)]) – Co-crystallised *n*-pentane molecule (0.5 per asymmetric unit) disordered about an inversion center. Its site occupancy factor was constrained to 0.5. Hydrogen atoms of the reduced benzene moiety were placed as indicated by a difference electron density map and their positions were refined together with isotropic displacement

parameters. The ligand backbone hydrogen atoms H8, H51 were placed as indicated by a difference electron density map and its position was refined together with an isotropic displacement parameter.

[{(^{DIPeP}BDI*)Sr}$_2$(η^6:η^6-C_6H_6)]) – Co-crystallised *n*-pentane molecule (0.5 per asymmetric unit) disordered about an inversion center. Its site occupancy factor was constrained to 0.5. SADI and RIGU restraints were applied to ensure a reasonable geometry of *n*-pentane molecule. Hydrogen atoms of the reduced benzene moiety were placed as indicated by a difference electron density map and their positions were refined together with isotropic displacement parameters. SADI restraints were necessary to obtain a reasonable position of H5.

[{(^{DIPeP}BDI*)Ba}$_2$(η^6:η^6-C_6H_6)]) – Significant disorder affecting two DIPeP moieties and three 3-pentyl groups of two compound molecules in asymmetric unit and heavily disordered solvent. The disorder was modeled using SADI and SIMU restrains. For two 3-Pentyl group, the relative occupancies of each of two position were refined to 0.701(19)/0.299(19) (3-pentyl 1) and 0.47(3)/0.53(3) (3-pentyl 2). Next two 3-Pentyl groups were split into 3 parts with free variables related linearly by SUMP command and their occupancy sites refined to 0.469(3)/0.320(3)/0.211(3) (3-pentyl 2) and 0.19(3)/0.620(3)/0.190(3) (3-pentyl 3). The relative occupancies of DIPeP moieties were refined to 0.537(4)/0.463(4) (DIPeP 1) and 0.810(3)/0.190(3) (DIPeP 2). Isotropic restrain (ISOR) was used on C27A and C27C. The ligand backbone hydrogen atoms H8, H51, H8A, H51A were placed as indicated by a difference electron density map and its position was refined together with an isotropic displacement parameter. Co-crystallised *n*-pentane and cyclohexane were the main occupants of the solvent cavities (an mixture of *n*-pentane and cyclohexane were used for crystallisation and their contribution to the structure factors was secured by back-Fourier transformation using the solvent mask routine· The solvent accessible voids treated this way had a size of 1867.0 Å^3 (19.2% of the unit cell) and contained 335 electrons/unit cell.

[{(^{DIPP}BDI*)Ba}$_2$(η^6:η^6-$C_{16}H_{10}$)] – Disorder of one co-crystallised benzene molecule and *iso*-propyl group. The disordered benzene molecule was modeled using SIMU and SAME restraints and refined to relative occupancies 0.83(2)/0.17(2). The disordered *iso*-propyl group was modeled using similarity restraints SIMU. The relative occupancies of each position were refined to 0.77(3)/0.23(3). The ligand backbone hydrogen atoms H18, H53, H96 were placed as indicated by a difference electron density map and its position was refined together with an isotropic displacement parameter.

The crystal structure data has been deposited with the Cambridge Crystallographic Data Centre. CCDC 2435047-2435054, 2435056 contain the supplementary crystallographic data for the complexes. This data can be obtained free of charge from The Cambridge Crystallographic Data Centre via www.ccdc.cam.ac.uk/data_request/cif.

Crystallographic and refinement data are summarised in **Tables S1-3**.

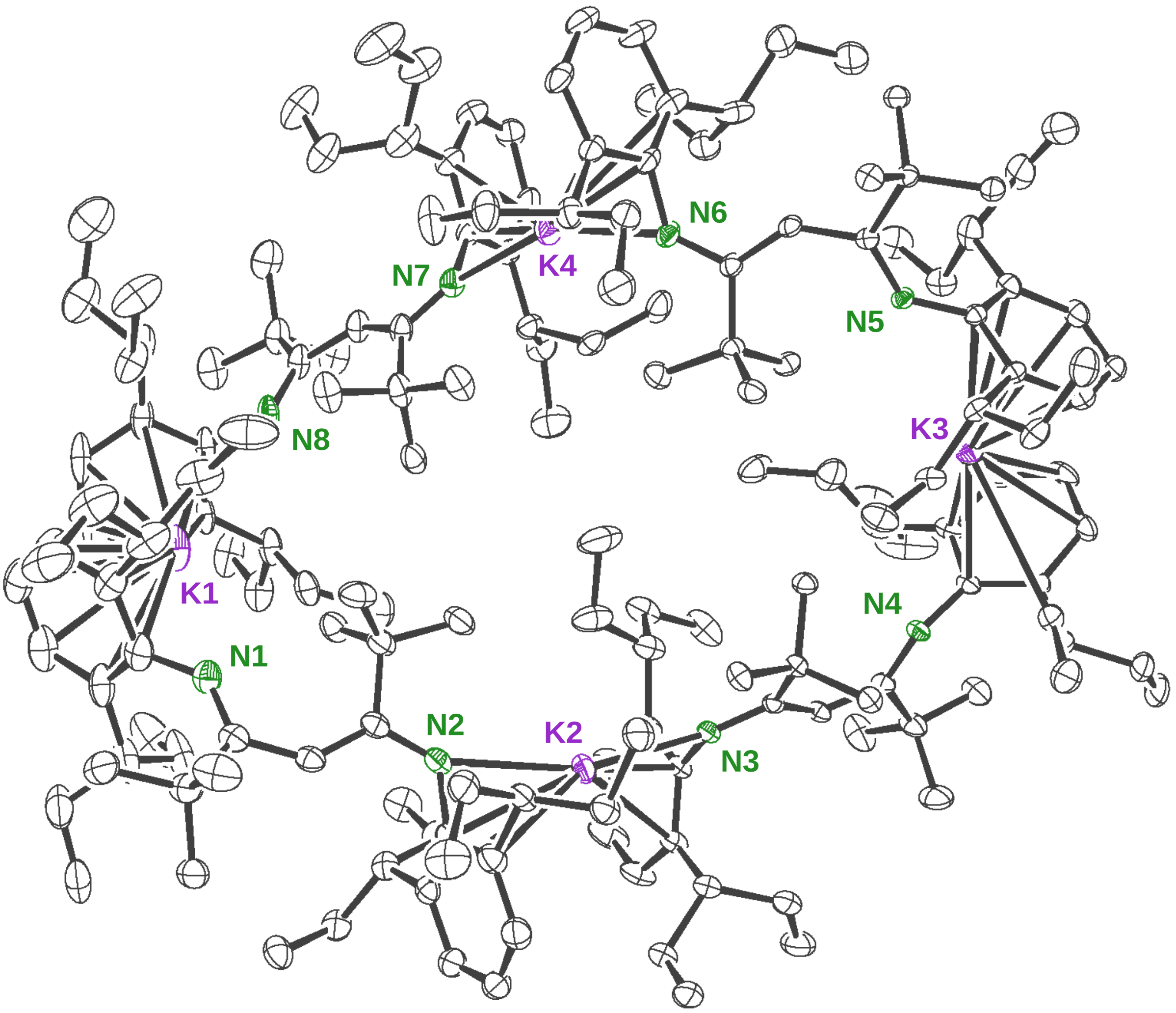

Figure S99. Molecular structure of [(^{DIPeP}BDI*)K]$_2$ (30% ellipsoid probability). Hydrogen atoms and disorder on DIPEP moieties are omitted for clarity.

Table S1. Crystal data and structure refinement for compounds [(^{DIPeP}BDI*)K]$_4$, [(^{DIPeP}BDI*)Ca(μ-I)]$_2$, [(^{DIPeP}BDI*)CaI·THF].

Compound	[(^{DIPeP}BDI*)K]$_4$ · solvent	[(^{DIPeP}BDI*)Ca(μ-I)]$_2$ · 2(C_6H_6)	[(^{DIPeP}BDI*)CaI·THF] · C_5H_{12}
Identification code	hasj190821a	hasj200228a	hasj220505b
Empirical formula	$C_{172}H_{276}K_4N_8$ [a)]	$C_{92}H_{144}Ca_2I_2N_4$	$C_{52}H_{89}CaIN_2O$
Formula weight	2612.39 [a)]	1640.06	925.23
Temperature/K	100.0(2)	99.9(4)	100.01(10)
Crystal system	triclinic	orthorhombic	orthorhombic
Space group	P-1	Pbcn	Pbca
a/Å	18.0046(3)	21.0407(3)	17.1405(3)
b/Å	19.7746(4)	20.0279(2)	16.1273(3)
c/Å	27.2387(5)	20.3948(2)	37.6114(9)
α/°	70.6596(17)	90	90
β/°	73.3726(16)	90	90
γ/°	85.7978(15)	90	90
Volume/Å^3	8765.5(3)	8594.39(18)	10396.9(4)
Z	2	4	8
ρ_{calc}g/cm^3	0.990 [a)]	1.268	1.182
μ/mm^{-1}	1.246 [a)]	7.124	0.750
F(000)	2880.0 [a)]	3480.0	3968.0
Crystal size/mm^3	0.189 × 0.169 × 0.116	0.196 × 0.17 × 0.098	0.323 × 0.258 × 0.188
Radiation	Cu Kα (λ = 1.54184)	Cu Kα (λ = 1.54184)	Mo Kα (λ = 0.71073)
2Θ range for data collection/°	6.804 to 145.608	7.478 to 145.628	4.088 to 51.362
Index ranges	-22 ≤ h ≤ 22, -23 ≤ k ≤ 21, -33 ≤ l ≤ 31	-25 ≤ h ≤ 21, -24 ≤ k ≤ 24, -22 ≤ l ≤ 24	-17 ≤ h ≤ 20, -19 ≤ k ≤ 19, -37 ≤ l ≤ 45
Reflections collected	98411	33862	45714
Independent reflections	33930 [R_{int} = 0.0316, R_{sigma} = 0.0299]	8374 [R_{int} = 0.0386, R_{sigma} = 0.0264]	9860 [R_{int} = 0.0534, R_{sigma} = 0.0393]
Data/restraints/parameters	33930/9244/2437	8374/685/577	9860/54/624
Goodness-of-fit on F^2	1.042	1.111	1.062
Final R indexes [I>=2σ (I)]	R_1 = 0.0584, wR_2 = 0.1564	R_1 = 0.0280, wR_2 = 0.0702	R_1 = 0.0475, wR_2 = 0.1129
Final R indexes [all data]	R_1 = 0.0689, wR_2 = 0.1689	R_1 = 0.0302, wR_2 = 0.0717	R_1 = 0.0644, wR_2 = 0.1263
Largest diff. peak/hole / e Å^{-3}	0.73/-0.53	0.33/-0.52	0.67/-1.25
CCDC number	2435047	2435048	2435049

a) Contribution of masked disordered solvent neglected.

Table S2. Crystal data and structure refinement for $[(^{DIPeP}BDI^*)Sr(\mu\text{-}I)]_2$, $[(^{DIPeP}BDI^*)Ba(\mu\text{-}I)]_2$, $[\{(^{DIPeP}BDI^*)Ca\}_2(\eta^6\text{:}\eta^6\text{-}C_6H_6)]$.

Compound	$[(^{DIPeP}BDI^*)Sr(\mu\text{-}I)]_2$	$[(^{DIPeP}BDI^*)Ba(\mu\text{-}I)]_2$ · solvent	$[\{(^{DIPeP}BDI^*)Ca\}_2(\eta^6\text{:}\eta^6\text{-}C_6H_6)]$ · $0.5(C_5H_{12})$
Identification code	hasj221212a	hasj190909a	hasj220622a
Empirical formula	$C_{86}H_{138}I_2N_4Sr_2$	$C_{86}H_{138}Ba_2I_2N_4$ a)	$C_{189}H_{300}Ca_4N_8$
Formula weight	1657.04	1756.48 a)	2844.67
Temperature/K	100.01(10)	99.99(13)	100.00(10)
Crystal system	tetragonal	tetragonal	monoclinic
Space group	$I4_1/acd$	I-4c2 b)	$P2_1/n$
a/Å	19.8990(2)	19.97390(10)	12.4360(14)
b/Å	19.8990(2)	19.97390(10)	22.839(2)
c/Å	42.0248(10)	24.5351(4)	30.727(3)
α/°	90	90	90
β/°	90	90	97.930(10)
γ/°	90	90	90
Volume/Å^3	16640.6(6)	9788.44(19)	8643.8(15)
Z	8	4	2
ρ_{calc}g/cm^3	1.323	1.192 a)	1.093
μ/mm^{-1}	7.832	1.468 a)	1.476
F(000)	6912.0	3600.0 a)	3140.0
Crystal size/mm^3	0.305 × 0.096 × 0.026	0.193 × 0.132 × 0.085	0.218 × 0.143 × 0.1
Radiation	Cu Kα (λ = 1.54184)	Mo Kα (λ = 0.71073)	Cu Kα (λ = 1.54184)
2Θ range for data collection/°	7.562 to 145.168	6.45 to 59.504	5.808 to 145.364
Index ranges	-17 ≤ h ≤ 22, -24 ≤ k ≤ 18, -28 ≤ l ≤ 50	-27 ≤ h ≤ 26, -27 ≤ k ≤ 25, -33 ≤ l ≤ 34	-15 ≤ h ≤ 10, -27 ≤ k ≤ 21, -37 ≤ l ≤ 37
Reflections collected	15954	83800	32013
Independent reflections	4084 [R_{int} = 0.0265, R_{sigma} = 0.0210]	6694 [R_{int} = 0.0391, R_{sigma} = 0.0190]	16601 [R_{int} = 0.0185, R_{sigma} = 0.0265]
Data/restraints/parameters	4084/0/221	6694/0/221	16601/0/991
Goodness-of-fit on F^2	1.054	1.041	1.017
Final R indexes [I>=2σ (I)]	R_1 = 0.0299, wR_2 = 0.0736	R_1 = 0.0199, wR_2 = 0.0425	R_1 = 0.0330, wR_2 = 0.0796
Final R indexes [all data]	R_1 = 0.0373, wR_2 = 0.0782	R_1 = 0.0239, wR_2 = 0.0440	R_1 = 0.0394, wR_2 = 0.0833
Largest diff. peak/hole / e Å^{-3}	0.92/-0.58	0.51/-0.31	0.46/-0.22
CCDC number	2435050	2435051	2435052

a) Contribution of masked disordered solvent neglected. b) Flack parameter -0.015(5)

Table S3. Crystal data and structure refinement for [{(^{DIPeP}BDI*)Sr}$_2$(η^6:η^6-C_6H_6)], [{(^{DIPeP}BDI*)Ba}$_2$(η^6:η^6-C_6H_6)], [{(^{DIPeP}BDI*)Ba}$_2$(η^6:η^6-$C_{16}H_{10}$)].

Compound	[{(^{DIPeP}BDI*)Sr}$_2$(η^6:η^6-C_6H_6)] · 0.5(C_5H_{12})	[{(^{DIPeP}BDI*)Ba}$_2$(η^6:η^6-C_6H_6)] · solvent	[{(^{DIPP}BDI*)Ba}$_2$(η^6:η^6-$C_{16}H_{10}$)] · 3(C_5H_{12})
Identification code	hasj230320a	hasj230815a	hasj240412b
Empirical formula	$C_{94.5}H_{150}N_4Sr_2$	$C_{92}H_{144}Ba_2N_4$	$C_{104}H_{134}Ba_2N_4$
Formula weight	1517.42	1580.78	1714.82
Temperature/K	100.00(10)	99.97(11)	99.97(13)
Crystal system	monoclinic	triclinic	triclinic
Space group	P2$_1$/n	P-1	P-1
a/Å	12.21855(8)	15.84065(18)	9.64881(13)
b/Å	22.92842(17)	23.0138(3)	26.2579(3)
c/Å	31.5562(3)	30.6235(3)	27.1109(3)
α/°	90	108.8315(10)	78.9461(10)
β/°	97.7853(7)	94.1080(9)	85.4731(10)
γ/°	90	109.8516(11)	79.5197(11)
Volume/Å^3	8759.05(12)	9730.2(2)	6621.95(14)
Z	4	4	3
ρ_{calc}g/cm^3	1.151	1.079	1.290
μ/mm^{-1}	1.931	6.539	0.936
F(000)	3284.0	3344.0	2694.0
Crystal size/mm^3	0.299 × 0.231 × 0.031	0.209 × 0.189 × 0.124	0.29 × 0.24 × 0.07
Radiation	Cu Kα (λ = 1.54184)	CuKα (λ = 1.54184)	Mo Kα (λ = 0.71073)
2Θ range for data collection/°	5.654 to 146.168	4.394 to 145.326	3.064 to 52.044
Index ranges	-15 ≤ h ≤ 14, -28 ≤ k ≤ 27, -23 ≤ l ≤ 38	-17 ≤ h ≤ 19, -28 ≤ k ≤ 27, -37 ≤ l ≤ 37	-11 ≤ h ≤ 11, -32 ≤ k ≤ 32, -33 ≤ l ≤ 33
Reflections collected	66423	127173	108756
Independent reflections	17090 [R_{int} = 0.0404, R_{sigma} = 0.0327]	37786 [R_{int} = 0.0427, R_{sigma} = 0.0395]	26072 [R_{int} = 0.0443, R_{sigma} = 0.0428]
Data/restraints/parameters	17090/31/978	37786/1605/2382	26072/210/1677
Goodness-of-fit on F^2	1.019	1.006	1.085
Final R indexes [I>=2σ (I)]	R_1 = 0.0325, wR_2 = 0.0812	R_1 = 0.0372, wR_2 = 0.0908	R_1 = 0.0358, wR_2 = 0.0721
Final R indexes [all data]	R_1 = 0.0374, wR_2 = 0.0846	R_1 = 0.0419, wR_2 = 0.0940	R_1 = 0.0477, wR_2 = 0.0770
Largest diff. peak/hole / e Å^{-3}	1.40/-0.80	1.29/-1.62	1.72/-0.69
CCDC number	2435053	2435054	2435056

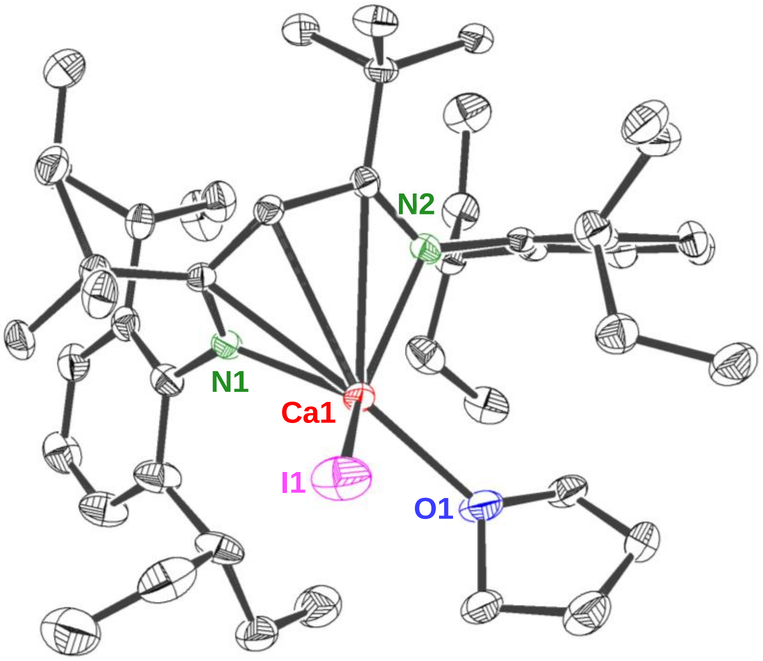

Figure S100. Molecular structure of [(^{DIPeP}BDI*)Ca·THF)] (50% ellipsoid probability). Hydrogen atoms and disorder are omitted for clarity.

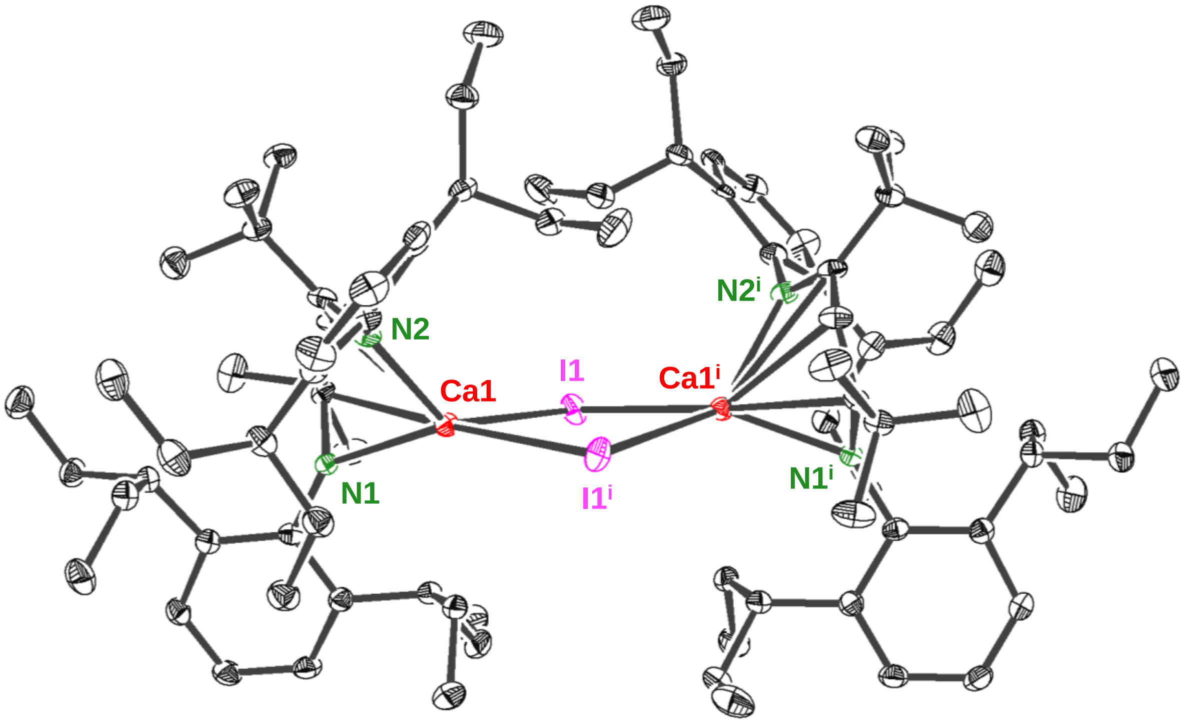

.

Figure S101. Molecular structure of [(^{DIPeP}BDI*)Ba(μ-)]$_2$ (50% ellipsoid probability). Hydrogen atoms and disorder are omitted for clarity. ^{i}1-X,+Y,1/2-Z.

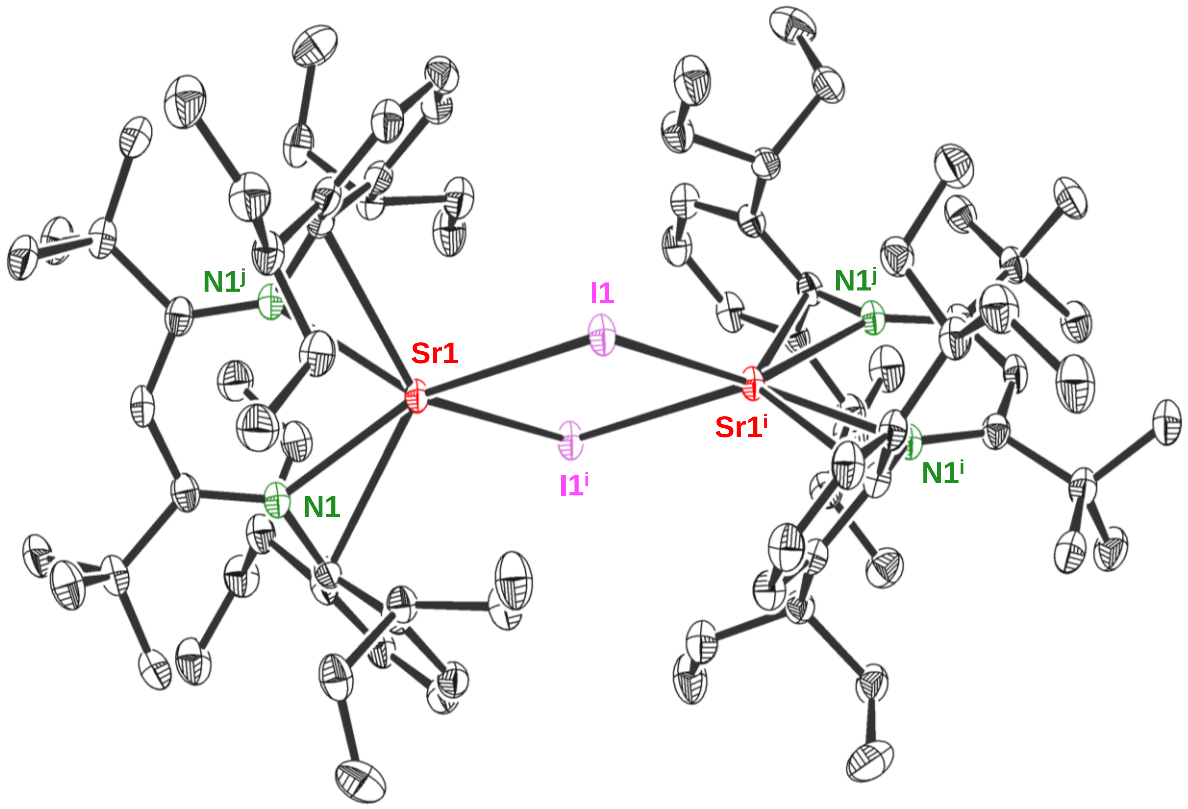

Figure S102. Molecular structure of [(^{DIPeP}BDI*)Sr(μ-)]$_2$ (50% ellipsoid probability). Hydrogen atoms are omitted for clarity. ^{i}2-X,1/2-Y,+Z; ^{j}5/4-Y,5/4-X,5/4-Z.

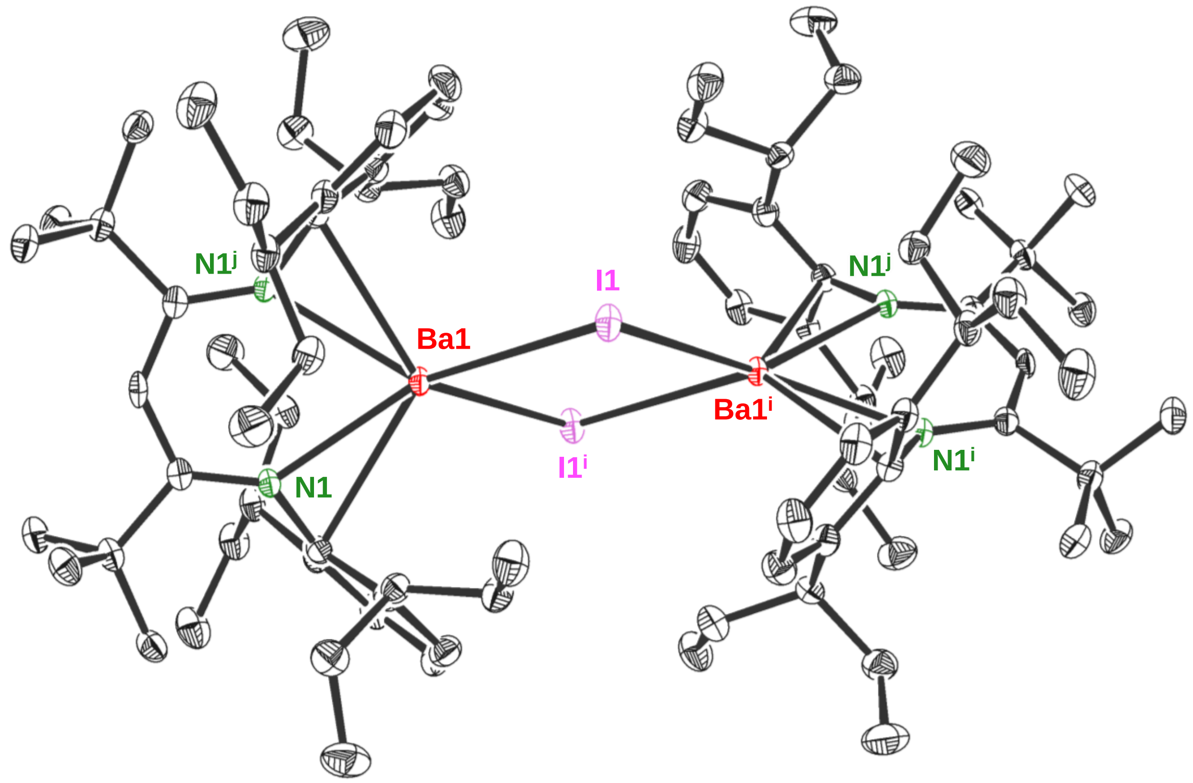

Figure S103. Molecular structure of [(^{DIPeP}BDI*)Ba(μ-)]$_2$ (50% ellipsoid probability). Hydrogen atoms are omitted for clarity. ^{i}1-X,1-Y,+Z; ^{j}1-Y,1-X,3/2-Z.

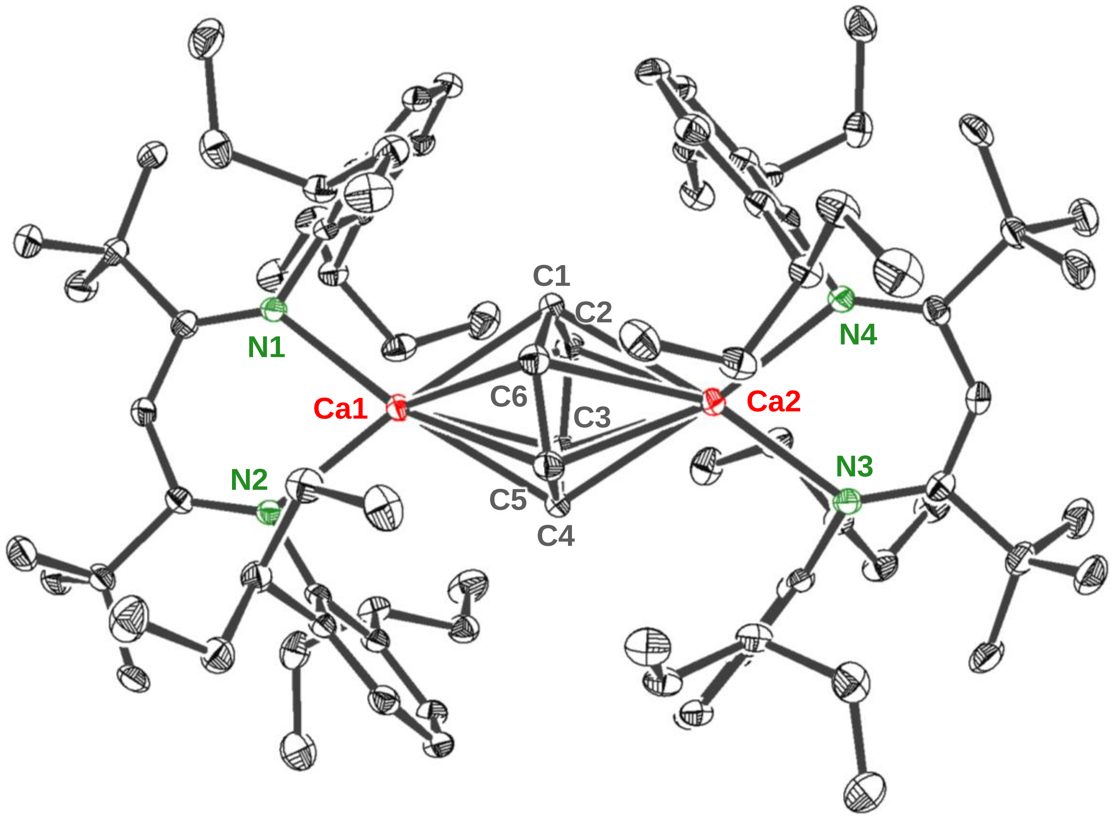

Figure S104. Molecular structure of [{(^{DIPeP}BDI*)Ca}$_2$(η^6:η^6-C_6H_6)] (50% ellipsoid probability). Hydrogen atoms are omitted for clarity.

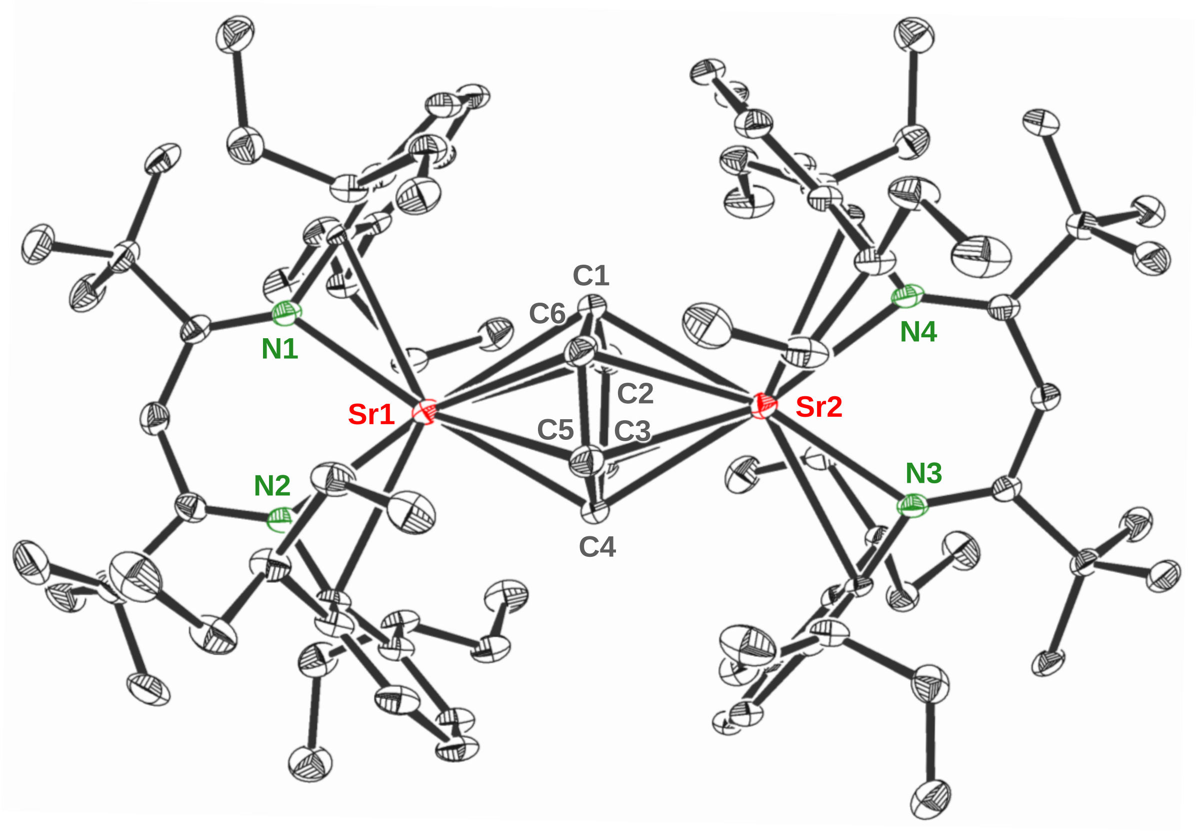

Figure S105. Molecular structure of [{(^{DIPeP}BDI*)Sr}$_2$(η^6:η^6-C_6H_6)] (50% ellipsoid probability). Hydrogen atoms are omitted for clarity.

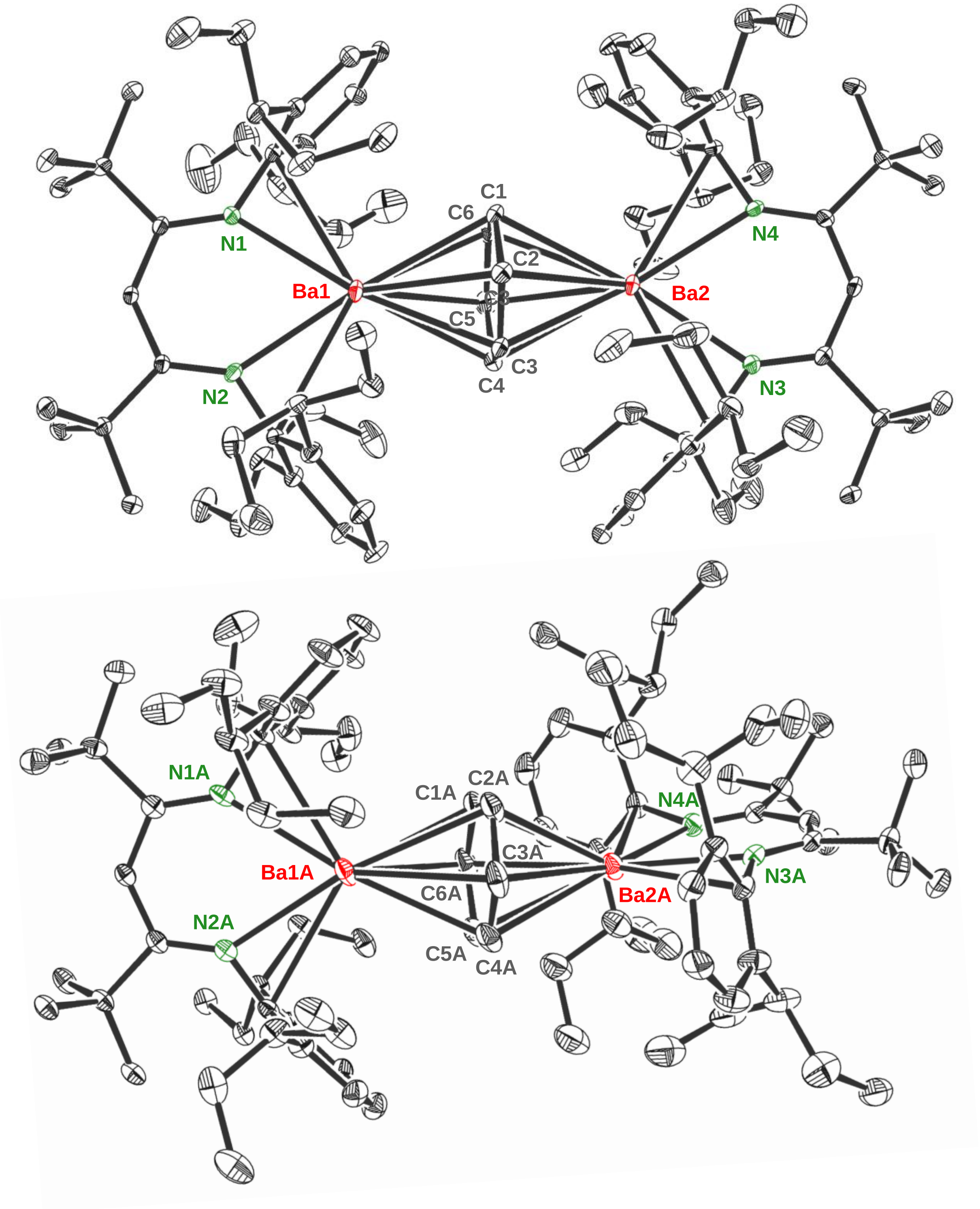

Figure S106. Molecular structures of [{(^{DIPeP}BDI*)Ba}$_2$(η^6:η^6-C_6H_6)] (50% ellipsoid probability). Hydrogen atoms and disorder are omitted for clarity.

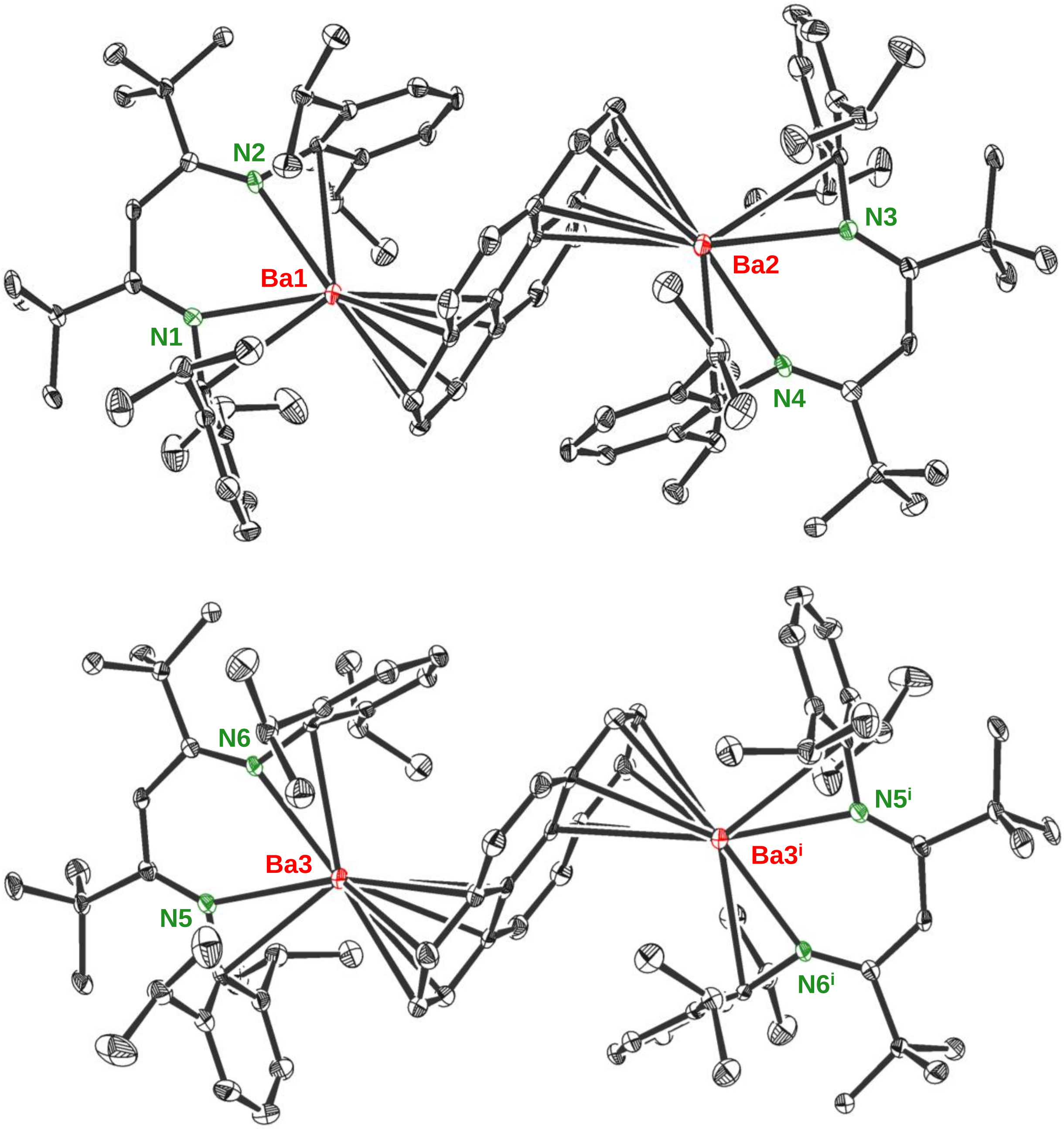

Figure S107. Molecular structures of [{(^{DIPP}BDI*)Ba}$_2$(η^6:η^6-$C_{16}H_{10}$)] (50% ellipsoid probability). Hydrogen atoms and disorder of *iso*-propyl group are omitted for clarity. ^{i}-X,2-Y,-Z.

1.7 EPR Measurements

EPR samples of ^{13}C-labeled Ae benzene complexes were prepared *via* benzene dianion exchange. $[\{(^{DIPeP}BDI)Ca\}_2(\eta^6{:}\eta^6\text{-}C_6H_6)]$[S29] (5 mg, 4.1 μmol) or $[\{(^{DIPeP}BDI)Sr\}_2(\eta^6{:}\eta^6\text{-}C_6H_6)]$[S30] (5 mg, 3.8 μmol) or $[\{(^{DIPeP}BDI^*)Ba\}_2(\eta^6{:}\eta^6\text{-}C_6H_6)]$ (5 mg, 3.2 μmol) was dissolved in 0.5 mL of methylcyclohexane and an excess of $^{13}C_6H_6$ was added (5 μL, 55 μmol) resulting in formation of $[\{(^{DIPeP}BDI)Ca\}_2(\eta^6{:}\eta^6\text{-}^{13}C_6H_6)]$, $[\{(^{DIPeP}BDI)Sr\}_2(\eta^6{:}\eta^6\text{-}^{13}C_6H_6)]$, $[\{(^{DIPeP}BDI^*)Ba\}_2(\eta^6{:}\eta^6\text{-}^{13}C_6H_6)]$. Solutions were transferred to quartz EPR tubes that were immediately frozen, flame-sealed and stored under liquid nitrogen. The spectra were measured as frozen solutions at 110 K with a microwave frequency of about 9.5 GHz.

The three broad triplet spectra, presented in Figure S108, resemble the lineshape of a typical doublet with small g-anisotropy, which indicates that zero-field splitting (ZFS) is quite small (<< 0.01 cm^{-1}). The small ZFS implies that the unpaired electrons are not localized to the alkali earth nuclei, and are delocalized to a large average interelectron distance. Hyperfine coupling to six equivalent ^{13}C nuclei is clearly visible for $[\{(^{DIPeP}BDI)Ca\}_2(\eta^6{:}\eta^6\text{-}^{13}C_6H_6)]$ and $[\{(^{DIPeP}BDI)Sr\}_2(\eta^6{:}\eta^6\text{-}^{13}C_6H_6)]$, with anisotropic hyperfine coupling constants of approximately 10-15 MHz (0.4–0.5 mT), somewhat larger than that previously reported for the doublet benzene anion (0.28 mT).[S19] Further EPR investigations of these complexes, including spin Hamiltonian simulations, are ongoing. Overall, the EPR spectroscopy is consistent with the benzene-dianion and triplet character assigned to these complexes.

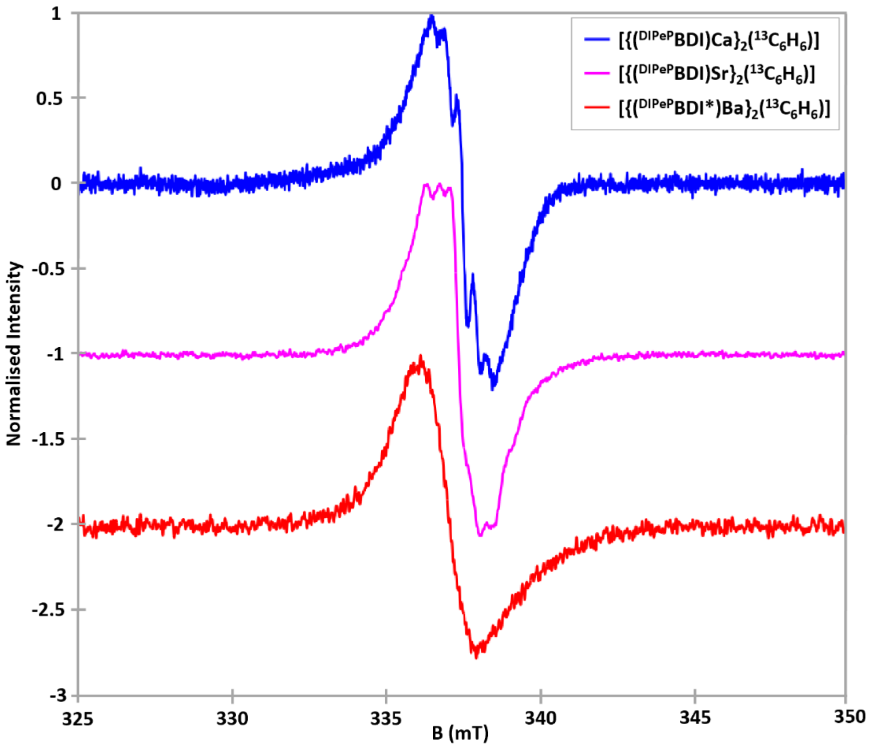

Figure S108. X-band EPR of complexes $[\{(^{DIPeP}BDI)Ae\}_2(^{13}C_6H_6)]$ (Ae = Ca, Sr) and $[\{(^{DIPeP}BDI^*)Ba\}_2(^{13}C_6H_6)]$ normalized to a maximum derivative intensity of 1. For $[\{(^{DIPeP}BDI)Ca\}_2(^{13}C_6H_6)]$, a microwave power of 5 mW and modulation amplitude of 0.02 mT were used. For $[\{(^{DIPeP}BDI)Sr\}_2(^{13}C_6H_6)]$ and $[\{(^{DIPeP}BDI^*)Ba\}_2(^{13}C_6H_6)]$, a microwave power of 0.2 mW and a modulation amplitude of 0.4 mT were used.

1.8 Computational Details

Geometry optimisations, frequency calculations and PCM solvent corrections were run with Gaussian 16 Revision A.03[S20] using the (U)B3PW91[S21-S22] functional. For geometry optimisations, all atoms were described with def2-SVP basis sets of Ahlrichs and Weigand and incorporating a correction for dispersion effects using Grimme's D3 parameter set with Becke-Johnson dampening[S23-S24] (*i.e.* (U)B3PW91-D3BJ).[S25] Single point energy calculations were performed on the optimised geometries, at the (U)B3PW91/def2-TZVP level of theory. Stationary points were fully characterised using analytical frequency calculations as either minima (all positive eigenvalues) or transition states (one negative eigenvalue). IRC calculations and subsequent geometry optimisations were used to confirm the minima linked by the transition states. Energies are given in atomic units (a.u.) unless otherwise stated. Natural Bond Orbital (NBO) and Natural Population Analysis (NPA) was performed using NBO-7 using the single point calculations performed at the (U)B3PW91/def2-TZVP level of theory.[26] Molecular orbitals and spin densities isosurfaces were visualised by VESTA.[27]

Table S4. Relative energies calculated for [{(^{DIPeP}BDI*)Ae}$_2$((η^6:η^6-C_6H_6)] using restricted singlet (Ae-Bz_s), unrestricted singlet (Ae-Bz_oss), and unrestricted triplet (Ae-Bz_t) wavefunctions (Ae = Ca, Sr, Ba).

Compound	Spin	Functional	Basis Set	E (SCF)	H (0 K)	G (298 K)	ΔE (SCF) Relative to Closed Shell Singlet (kcal/mol)
Ca-Bz_s	0	B3PW91	def2-TZVP	-5166.5612	-5164.3884	-5164.5370	0
Ca-Bz_oss	0	UB3PW91	def2-TZVP	-5166.5612	-5164.3884	-5164.5370	0
Ca-Bz_t	1	UB3PW91	def2-TZVP	-5166.5716	-5164.3994	-5164.5494	-6.52
Sr-Bz_s	0	B3PW91	def2-TZVP	-3872.9653	-3870.7963	-3870.9483	0
Sr-Bz_oss	0	UB3PW91	def2-TZVP	-3872.9653	-3870.7963	-3870.9483	0
Sr-Bz_t	1	UB3PW91	def2-TZVP	-3872.9674	-3870.7976	-3870.9507	-1.30
Ba-Bz_s	0	B3PW91	def2-TZVP	-3862.5377	-3860.3702	-3860.5243	0
Ba-Bz_oss	0	UB3PW91	def2-TZVP	-3862.5377	-3860.3702	-3860.5243	-0.01
Ba-Bz_t	1	UB3PW91	def2-TZVP	-3862.5397	-3860.3727	-3860.5258	-1.28

Table S5. Relative energies calculated for [(κ^2,κ^1-^{DIPP}BDI*)$_2$Ba] (Ba-Ho_open) and [(κ^2,κ^2-^{DIPP}BDI*)$_2$Ba] (Ba-Ho_closed) using restricted singlet wavefunction.

Compound	Functional	Basis Set	E (SCF)	H (0 K)	G (298 K)	ΔE (SCF) Relative to Ba Homo_open (kcal/mol)
Ba-Ho_open	B3PW91	def2-TZVP	-2975.80296361	-2974.732347	-2974.857812	0.00
Ba-Ho_closed	B3PW91	def2-TZVP	-2975.71881809	-2974.683064	-2974.797954	+52.80

Table S6. Natural Population Analysis (NPA) charges calculated for [{(^{DIPeP}BDI*)Ae}$_2$((η^6:η^6-C_6H_6)] and [{(^{DIPP}BDI)Mg}$_2$((η^2:η^4-C_6H_6)] using restricted singlet (Ae-Bz_s, Mg-Bz_s) and unrestricted triplet (Ae-Bz_t) wavefunctions (Ae = Ca, Sr, Ba).

Compound	[BDI]	[Ae1]	[C_6H_6]	[Ae2]	[BDI]
Mg-Bz_s	-0.90	+1.69	-1.63	+1.75	-0.91
Ca-Bz_s	-0.93	+1.71	-1.56	+1.74	-0.96
Ca-Bz_t	-0.51 (α)<br>-0.43 (β)	+0.83 (α)<br>+0.90 (β)	-1.64 (α)<br>+0.06 (β)	+0.83 (α)<br>+0.90 (β)	-0.52 (α)<br>-0.43 (β)
Sr-Bz_s	-0.95	+1.74	-1.59	+1.75	-0.96
Sr-Bz_t	-0.52 (α)<br>-0.43 (β)	+0.85 (α)<br>+0.91 (β)	-1.67 (α)<br>+0.05 (β)	+0.86 (α)<br>+0.91 (β)	-0.52 (α)<br>-0.43 (β)
Ba-Bz_s	-0.96	+1.72	-1.58	+1.75	-0.94
Ba-Bz_t	-0.50 (α)<br>-0.44 (β)	+0.83 (α)<br>+0.91 (β)	-1.65 (α)<br>+0.06 (β)	+0.84 (α)<br>+0.91 (β)	-0.52 (α)<br>-0.44 (β)

Table S7. Natural Electron Configuration calculated for [{(^{DIPeP}BDI*)Ae}$_2$((η^6:η^6-C_6H_6)] and [{(^{DIPP}BDI)Mg}$_2$((η^2:η^4-C_6H_6)] using restricted singlet (Ae-Bz_s, Mg-Bz_s) and unrestricted triplet (Ae-Bz_t) wavefunctions (Ae = Ca, Sr, Ba).

Compound	[Ae1]	[Ae2]
Mg-Bz_s	[core]3s(0.28)3p(0.01)3d(0.01)	[core]3s(0.22)3p(0.01)3d(0.01)
Ca-Bz_s	[core]4s(0.11)3d(0.16)4d(0.01)	[core]4s(0.10)3d(0.14)4d(0.01)
Ca-Bz_t	[core]4s(0.10)3d(0.15)4d(0.01)	[core]4s(0.10)3d(0.15)4d(0.01)
Sr-Bz_s	[core]5s(0.10)4d(0.14)5d(0.01)	[core]5s(0.09)4d(0.12)5p(0.01)5d(0.01)
Sr-Bz_t	[core]5s(0.10)4d(0.13)5d(0.01)	[core]5s(0.09)4d(0.12)5d(0.01)
Ba-Bz_s	[core]6s(0.09)5d(0.17)6d(0.01)	[core]6s(0.09)5d(0.14)6d(0.01)
Ba-Bz_t	[core]6s(0.09)5d(0.14)6d(0.01)	[core]6s(0.09)5d(0.14)6d(0.01)

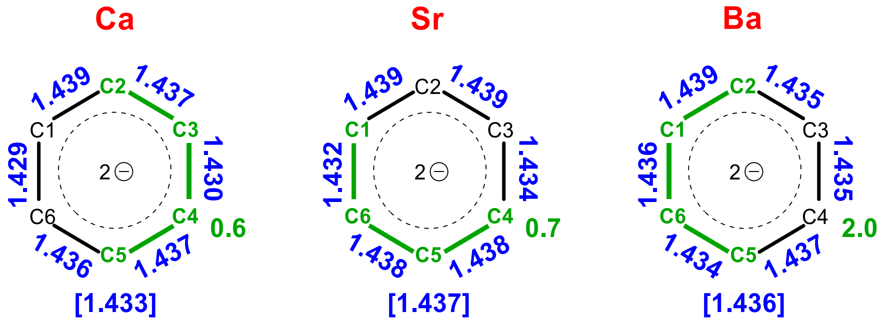

Figure S109. Calculated C-C interatomic distances in Å (blue) and the largest C-C-C-C torsion angles (green) within the $(C_6H_6)^{2-}$ rings for optimised Ae-Bz (Ae = Ca, Sr, Ba) in their singlet (top) and triplet (bottom) states; optimised at the (U)B3PW91-D3BJ/Def2-SVP level of theory. Carbon labeling C1 = 147, C2 = 148, C3 = 149, C4 = 150, C5 = 152, C6 = 151.

Table S8. Selected experimental (X-ray) and calculated (DFT) bond lengths/distances (Å) for crystal structures of [{(^{DIPeP}BDI*)Ae}$_2$((η^6:η^6-C_6H_6)] and optimised geometries (closed-shell singlet state). Labeling: Ae1 = 57, Ae2 = 58, N1 = 1, N2 = 59, N3 = 60, N4 =2, C18 = 5, C34 = 65, C61 = 94, C77 = 32, C1 = 147, C2 = 148, C3 = 149, C4 = 150, C5 = 152, C6 = 151.

	X-ray			DFT		
	Ca	Sr	Ba	Ca	Sr	Ba
Ae1–N1	2.388(1)	2.528(1)	2.706(2)	2.386	2.527	2.712
Ae1–N2	2.381(1)	2.521(2)	2.714(2)	2.409	2.540	2.729
Ae1–C18	2.941(1)	2.962(1)	3.036(3)	2.918	2.981	3.084
Ae1–C34	2.919(1)	2.939(2)	3.079(3)	2.915	3.006	3.199
Ae1–C1	2.670(1)	2.766(2)	2.951(4)	2.681	2.806	2.994
Ae1–C2	2.623(1)	2.746(2)	2.950(4)	2.596	2.796	3.001
Ae1–C3	2.754(1)	2.802(2)	2.958(3)	2.657	2.784	3.001
Ae1–C4	2.675(1)	2.771(2)	2.962(3)	2.668	2.775	2.966
Ae1–C5	2.593(1)	2.743(2)	2.947(3)	2.585	2.755	2.932
Ae1–C6	2.731(1)	2.792(2)	2.932(3)	2.680	2.793	2.949
Ae1–cent.[a]	2.272(1)	2.381(1)	2.579(1)	2.222	2.385	2.604
Ae2–N3	2.380(1)	2.517(1)	2.711(3)	2.391	2.539	2.752
Ae2–N4	2.387(1)	2.521(2)	2.697(3)	2.417	2.546	2.759
Ae2–C61	2.947(1)	2.971(2)	3.083(3)	2.925	3.019	3.142
Ae2–C77	2.950(1)	2.977(2)	3.020(3)	2.983	3.014	3.155
Ae2–C1	2.667(1)	2.765(2)	2.897(3)	2.629	2.781	2.996
Ae2–C2	2.743(1)	2.805(2)	2.918(3)	2.710	2.801	3.081
Ae2–C3	2.610(1)	2.740(2)	2.966(3)	2.659	2.788	3.031
Ae2–C4	2.678(1)	2.768(2)	3.026(4)	2.686	2.806	2.992
Ae2–C5	2.782(1)	2.833(2)	2.971(4)	2.806	2.846	2.967
Ae2–C6	2.634(1)	2.774(2)	2.915(3)	2.674	2.794	2.948
Ae2–cent.[a]	2.258(1)	2.369(1)	2.578(2)	2.281	2.405	2.637

[a] centroid: C1,C2,C3,C4,C5,C6

Table S9. Selected experimental (X-ray) and calculated (DFT) bond lengths/distances (Å) for the crystal structure of [{(^{DIPP}BDI)Mg}$_2$((η^2:η^4-C_6H_6)][S28] and its optimised geometry (closed-shell singlet state). Labeling: Mg1 = 1, Mg2 = 2, N1 = 3, N2 = 4, N3 = 5, N4 = 6, , C1 = 7, C2 = 9, C3 = 11, C4 = 13, C5 = 15, C6 = 17.

X-ray				DFT			
Mg1–N1	2.050(2)	Mg2–N3	2.043(2)	Mg1–N1	2.0531	Mg2–N3	2.0504
Mg1–N2	2.044(5)	Mg2–N4	2.037(5)	Mg1–N2	2.0563	Mg2–N4	2.0529
Mg1–C1	2.297(5)	Mg2–C1	2.828(3)	Mg1–C1	2.2540	Mg2–C1	2.8721
Mg1–C2	2.792(4)	Mg2–C2	2.371(4)	Mg1–C2	2.7284	Mg2–C2	2.4153
Mg1–C3	2.667(3)	Mg2–C3	2.500(5)	Mg1–C3	2.6972	Mg2–C3	2.4453
Mg1–C4	2.294(3)	Mg2–C4	2.793(5)	Mg1–C4	2.2826	Mg2–C4	2.8169
Mg1–C5	2.723(4)	Mg2–C5	2.362(4)	Mg1–C5	2.7475	Mg2–C5	2.4094
Mg1–C6	2.635(5)	Mg2–C6	2.486(3)	Mg1–C6	2.7320	Mg2–C6	2.4489
Mg1–cent.[a]	2.167(3)	Mg2–cent.[a]	2.152(3)	Mg1–cent.[a]	2.166	Mg2–cent.[a]	2.158

[a] centroid: C1,C2,C3,C4,C5,C6

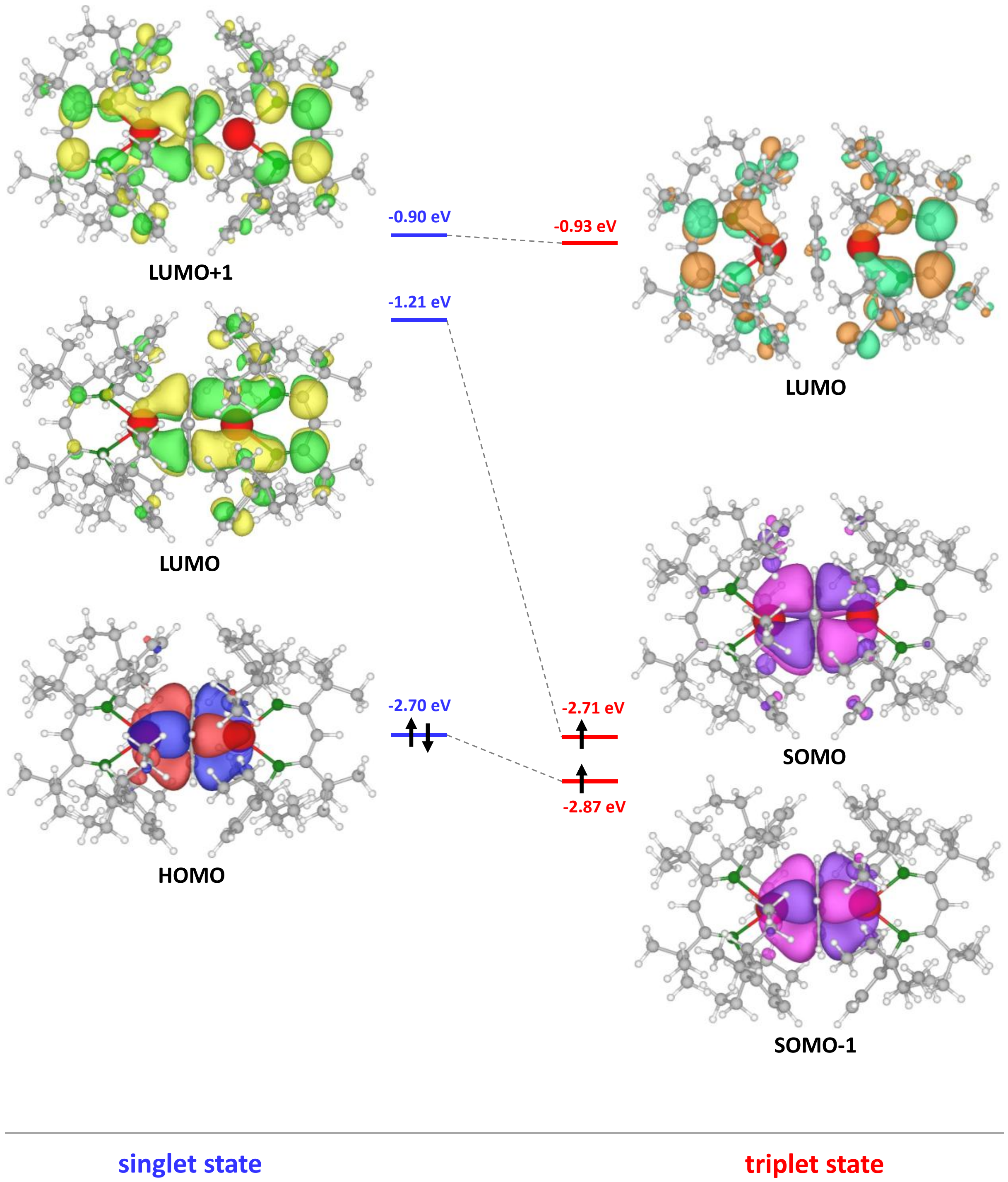

Figure S110. Comparison of significant molecular orbitals for singlet and triplet state calculated for [{(^{DIPeP}BDI*)Ca}$_2$(η^6:η^6-C_6H_6)]. Calculated at (U)B3PW91-D3BJ/Def2-TZVP level of theory

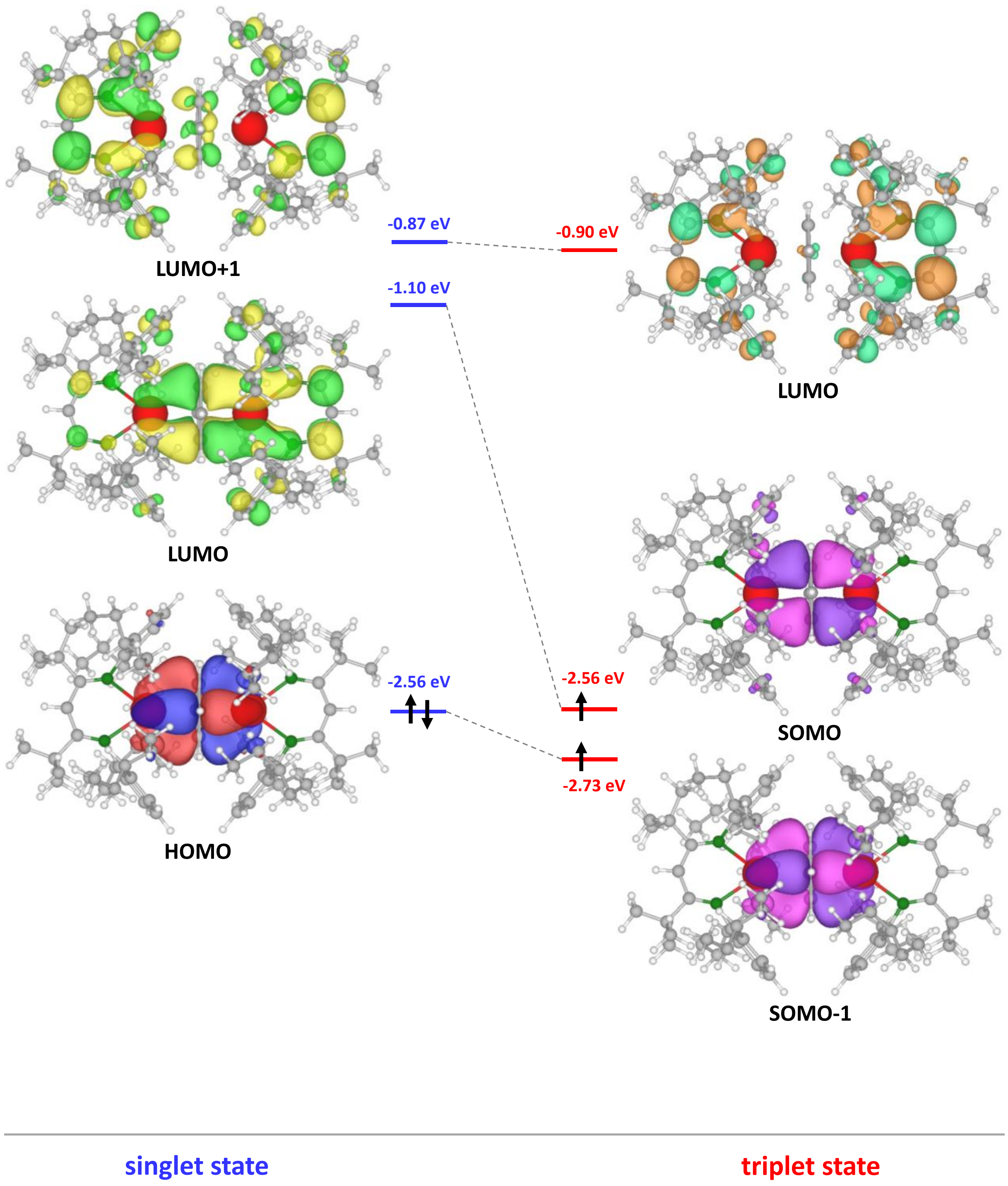

Figure S111. Comparison of significant molecular orbitals for singlet and triplet state calculated for $[\{(^{DIPeP}BDI^{*})Sr\}_2(\eta^6{:}\eta^6\text{-}C_6H_6)$. Calculated at (U)B3PW91-D3BJ/Def2-TZVP level of theory.

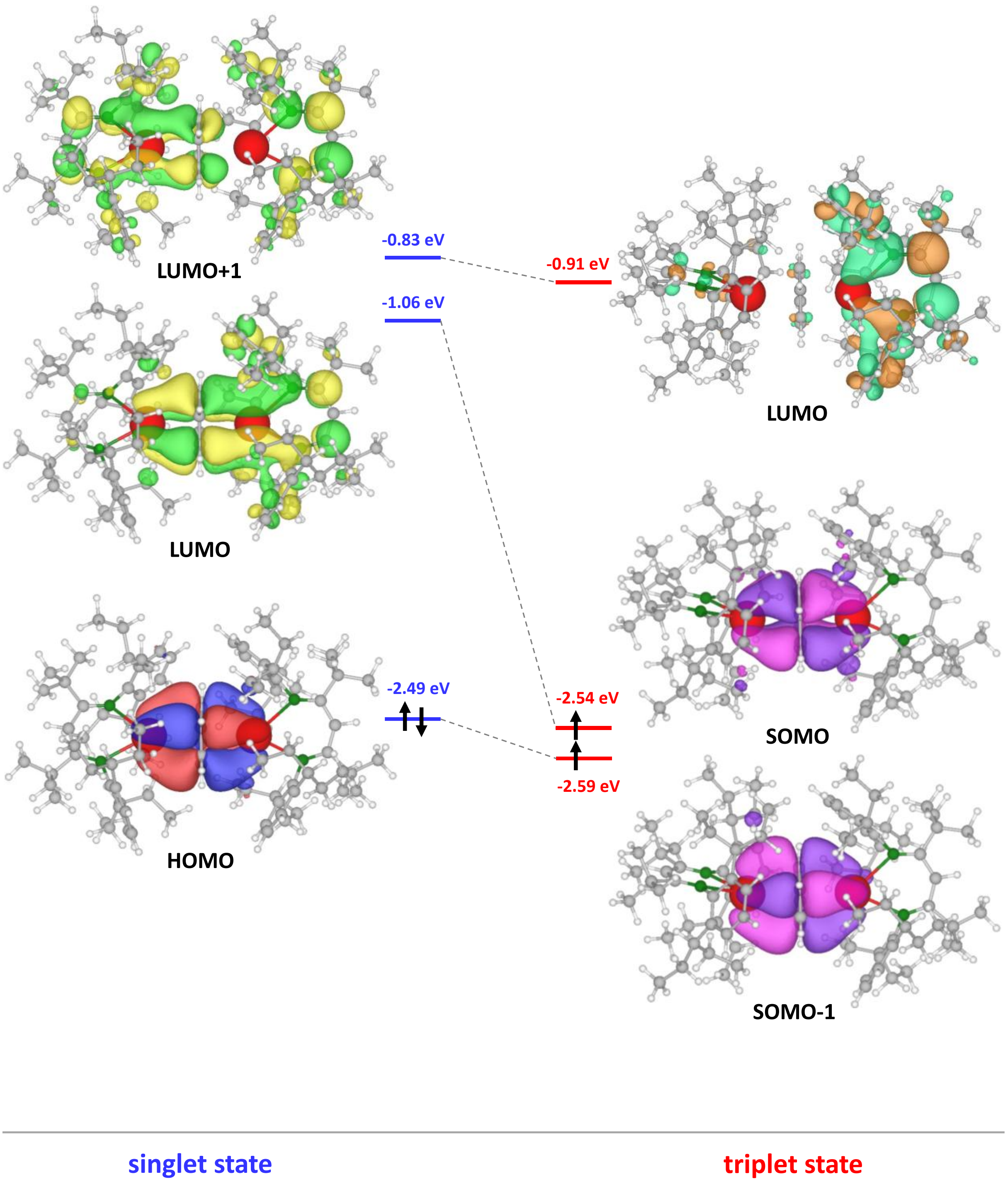

Figure S112. Comparison of significant molecular orbitals of [{(^{DIPeP}BDI*)Ba}$_2$(η^6:η^6-C_6H_6)] in their singlet and triplet state. Calculated at (U)B3PW91-D3BJ/Def2-TZVP level of theory.

Table S10. Kohn-Sham alpha spin densities calculated for [{(^{DIPeP}BDI*)Ae}$_2$((η^6:η^6-C_6H_6)] using triplet wavefunctions (isosurface 0.0015 e/Å^3). Spin density values for [C_6H_6] and Ae cations with their s, p, d orbitals percentage share. Calculated at UB3PW91-D3BJ/Def2-TZVP level of theory.

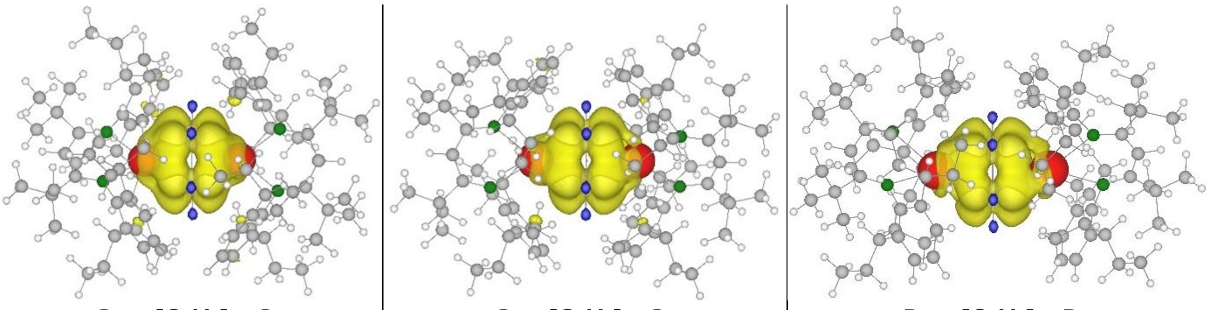

Ca	[C_6H_6]	Ca	Sr	[C_6H_6]	Sr	Ba	[C_6H_6]	Ba
**0.25**	**1.51**	**0.26**	**0.17**	**1.59**	**0.15**	**0.22**	**1.53**	**0.21**
6.7% s		7.7% s	14.3% s		11.4% s	4.2% s		8.3% s
11.8% p		12.5% p	24.9% p		26.1% p	30.7% p		31.0% p
81.5% d		79.8% d	60.8% d		62.5% d	64.5% d		60.1% d

Table S11. Natural spin densities values for [C_6H_6] and Ae cations calculated for [{(^{DIPeP}BDI*)Ae}$_2$((η^6:η^6-C_6H_6)] using triplet wavefunctions. Calculated at UB3PW91-D3BJ/Def2-TZVP level of theory.

Ca	[C_6H_6]	Ca	Sr	[C_6H_6]	Sr	Ba	[C_6H_6]	Ba
**0.067**	**1.693**	**0.065**	**0.058**	**1.719**	**0.053**	**0.074**	**1.707**	**0.072**

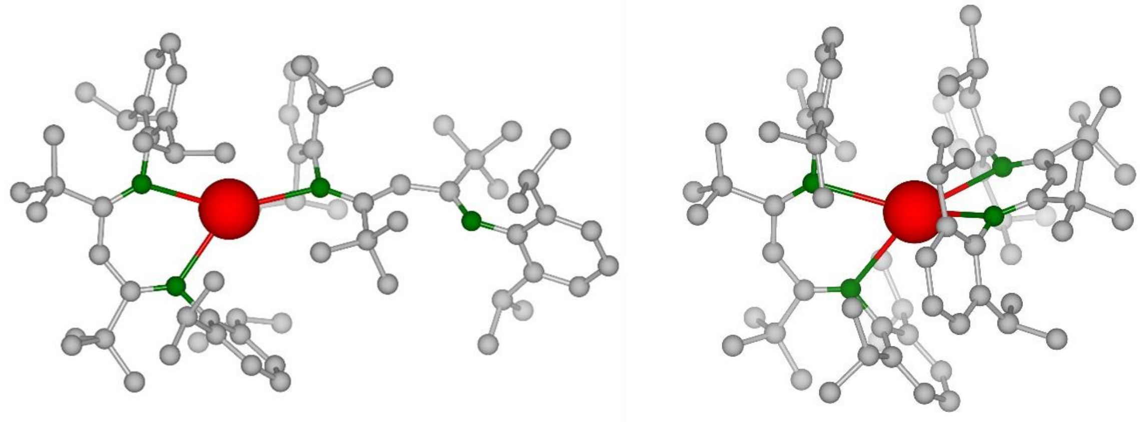

Ba-Ho_open **Ba-Ho_closed**

Figure S113. Optimised structures for [(κ^2,κ^1-^{DIPP}BDI*)$_2$Ba] (Ba-Ho_open) and [(κ^2,κ^2-^{DIPP}BDI*)$_2$Ba] (Ba-Ho_closed), modeled based on structures [(κ^2,κ^1-^{DIPeP}BDI)$_2$Ca][S29] and [(κ^2,κ^2-^{DIPP}BDI)$_2$Ba].[S31]

XYZ-coordinates

242
Ca-Bz_s

N	4.014400	1.379800	0.502100
N	-4.154700	1.500000	-0.417800
C	5.320600	1.263900	0.351000
C	6.363500	2.396700	0.621200
C	3.252900	2.480200	0.926800
C	2.818300	2.569800	2.277500
C	1.905000	3.573700	2.620100
H	1.581100	3.664200	3.659400
C	1.397700	4.458200	1.674800
H	0.674600	5.222200	1.967000
C	1.834500	4.368900	0.356500
H	1.452100	5.071500	-0.386700
C	2.767600	3.408300	-0.034900
C	3.368300	1.672600	3.378800
H	3.980800	0.893900	2.893000
C	2.209600	0.979800	4.116600
H	1.494800	1.748900	4.451900
H	1.669200	0.387900	3.363300
C	4.297100	2.491500	4.302900
H	3.680200	3.098900	4.988900
C	5.330300	1.695100	5.095000
C	3.279000	3.413200	-1.466200
H	4.193700	2.800000	-1.492500
C	2.258300	2.745700	-2.398900
H	2.064600	1.714100	-2.054200
H	1.294000	3.268400	-2.283900
C	3.644400	4.843100	-1.932200

H	3.822900	5.473800	-1.046900
C	4.869100	4.930100	-2.834700
C	-5.469800	1.305700	-0.383100
C	-6.504600	2.466000	-0.580600
C	-3.441700	2.717300	-0.410000
C	-2.856400	3.237600	-1.600900
C	-1.937400	4.288300	-1.490100
H	-1.440700	4.654200	-2.388500
C	-1.636700	4.876200	-0.267900
H	-0.933700	5.709400	-0.215700
C	-2.243200	4.395100	0.887900
H	-2.017500	4.866000	1.846000
C	-3.121000	3.310200	0.850000
C	-3.171100	2.664100	-2.973400
H	-3.986500	1.940300	-2.822500
C	-1.986000	1.859000	-3.519200
H	-1.083700	2.493600	-3.559700
H	-1.746000	1.075500	-2.787000
C	-3.695800	3.691300	-4.005600
C	-2.698300	4.643400	-4.659800
H	-4.188900	3.110100	-4.802600
C	-3.692500	2.750300	2.140700
H	-4.565200	2.143300	1.866800
C	-2.723800	1.774200	2.834100
H	-2.308000	1.075700	2.090300
C	-1.551600	2.391000	3.576700
C	-4.181400	3.838100	3.107200
H	-4.810800	4.549400	2.551200
C	-4.963000	3.286200	4.291900
Ca	2.190600	-0.047700	-0.071200
Ca	-2.310800	-0.019900	-0.052000
N	4.111900	-1.406700	-0.584100
N	-4.204400	-1.444900	0.263800
C	5.932100	0.054400	-0.062100
H	7.009500	0.113500	-0.076000
C	5.418700	-1.198000	-0.448700
C	6.493200	-2.283100	-0.796600
C	3.400600	-2.596200	-0.806600
C	2.883300	-2.912500	-2.091300
C	1.961000	-3.962400	-2.207800
H	1.557300	-4.202100	-3.194600
C	1.553100	-4.708100	-1.111300
H	0.834700	-5.520500	-1.234500
C	2.088600	-4.422000	0.143400
H	1.792300	-5.027600	1.002100
C	3.002900	-3.384400	0.322200
C	3.276600	-2.175400	-3.363700
H	4.099300	-1.487800	-3.115600
C	2.089000	-1.327300	-3.843200
H	1.194400	-1.969300	-3.853100
H	1.883900	-0.571100	-3.068600
C	3.768400	-3.176700	-4.437300
H	4.207300	-4.051300	-3.930200
H	2.894500	-3.572800	-4.984300
C	3.598500	-3.106000	1.689400
H	4.498500	-2.501600	1.517300
C	2.702400	-2.232000	2.582800
H	2.310900	-1.373400	2.010600
C	1.526100	-2.923800	3.248600
C	4.046700	-4.386500	2.408200
C	4.878500	-4.118900	3.655400
H	3.171000	-4.998900	2.677900
C	-6.044000	0.033400	-0.165100
H	-7.117400	0.041000	-0.212400
C	-5.510000	-1.240400	0.124000
C	-6.572000	-2.369900	0.351800
C	-3.500700	-2.660900	0.350300
C	-2.999200	-3.138500	1.588900
C	-2.106200	-4.219500	1.582500
H	-1.715200	-4.586700	2.534500
C	-1.705400	-4.833400	0.405100
H	-1.006100	-5.671000	0.431600
C	-2.217100	-4.379100	-0.809200
H	-1.920700	-4.877500	-1.733800
C	-3.107000	-3.307100	-0.865600
C	-3.355800	-2.527800	2.936700
H	-4.154500	-1.787900	2.773300
C	-2.129200	-1.782000	3.481300
H	-1.272800	-2.474000	3.468200
H	-1.862600	-0.995200	2.760500
C	-3.868000	-3.610400	3.917400
H	-4.328200	-4.427600	3.339100
H	-3.001600	-4.070800	4.424400
C	-3.675700	-2.845400	-2.195800
H	-4.576400	-2.261800	-1.964800
C	-2.753100	-1.871000	-2.951700
H	-2.364200	-1.105100	-2.259300
C	-1.568000	-2.487300	-3.673200
C	-4.116300	-4.017200	-3.085000
H	-4.710300	-4.715300	-2.475800
C	-4.925900	-3.584600	-4.299800
H	2.772800	5.294800	-2.438200
H	4.835000	3.222500	3.680200
C	2.550000	0.099100	5.311200
H	4.626700	-5.001200	1.702900
H	3.334600	-1.772800	3.356400
C	4.788900	-2.636500	-5.433200
C	2.212200	-0.642600	-5.194800
C	-4.872100	-3.132300	4.960100
C	-2.237000	-1.163800	4.866300
H	-3.238800	-4.594600	-3.418100
C	2.627100	2.684500	-3.871200
H	-3.361900	-1.303300	-3.671800
C	-2.220800	1.205900	-4.873200
H	-4.496700	4.287900	-3.547400
H	-3.328600	4.429600	3.477500
H	-3.300700	1.136200	3.519600
C	7.926800	-1.856000	-0.443100
C	6.258400	-3.616300	-0.076400
C	6.450700	-2.536000	-2.306700
C	7.244700	2.573100	-0.631800
C	5.786400	3.777000	0.949200
C	7.247300	1.976700	1.808400
C	-7.956000	1.976800	-0.713100
C	-6.469500	3.435100	0.608100
C	-6.191700	3.256500	-1.852200
C	-8.016900	-1.913500	0.095200
C	-6.349700	-3.597300	-0.540700
C	-6.493000	-2.811600	1.816400
C	-0.085800	1.243800	-0.653600
C	0.001000	0.017600	-1.464700
C	-0.069600	-1.265500	-0.754600
C	-0.054600	-1.314500	0.615300
C	-0.052600	1.190700	0.714500
C	0.078400	-0.088700	1.418600
H	-0.067100	0.054900	-2.545700
H	-0.109700	-2.199400	-1.313800
H	-0.116600	-2.285100	1.104900
H	-0.073900	2.122500	1.278300
H	-0.031900	-0.126900	2.496800
H	-0.177200	2.214600	-1.136100
H	-0.857300	1.610500	3.917000
H	-0.978100	3.077200	2.938200
H	-1.879600	2.950700	4.465400
H	-5.367000	4.098100	4.915600
H	-5.812400	2.670000	3.955100
H	-4.338400	2.654300	4.942200
H	6.081600	2.367500	5.537800
H	4.890000	1.113000	5.913800
H	5.866300	0.984600	4.446200
H	1.666300	-0.482700	5.617100
H	3.352700	-0.619400	5.086000
H	2.866200	0.685700	6.186100
H	5.248000	-5.056600	4.097900
H	5.754900	-3.492300	3.421600
H	4.302200	-3.594200	4.433100
H	0.897200	-2.187800	3.768800
H	0.890300	-3.446900	2.519500
H	1.852400	-3.661700	3.997000
H	-5.189600	-3.966300	5.605400
H	-4.464400	-2.348900	5.612300
H	-5.774700	-2.723500	4.483200
H	-1.341400	-0.561000	5.083200
H	-3.110300	-0.499600	4.957600
H	-2.312400	-1.925300	5.656700
H	-1.394300	0.524300	-5.126400
H	-3.149700	0.613800	-4.873900
H	-2.299600	1.940200	-5.688700
H	-3.165900	5.142300	-5.523000
H	-2.367400	5.439000	-3.977800
H	-1.800400	4.126600	-5.031800
H	-5.289200	-4.455100	-4.866900
H	-5.805300	-2.990800	-4.000900
H	-4.335300	-2.965700	-4.992900
H	-0.928700	-1.702000	-4.098600
H	-0.944000	-3.091800	-2.999200
H	-1.887100	-3.134200	-4.504000
H	5.094900	-3.423700	-6.140200
H	4.401900	-1.796500	-6.024200
H	5.695900	-2.284600	-4.920800
H	1.334700	-0.002000	-5.375800
H	3.104200	-0.001200	-5.254800
H	2.258500	-1.364100	-6.024300
H	5.038400	5.964600	-3.172100
H	5.773800	4.608400	-2.297200
H	4.779700	4.299100	-3.729700
H	2.717700	3.686300	-4.316600
H	3.580800	2.156200	-4.027100
H	1.853200	2.145400	-4.435400
H	-8.600600	2.847100	-0.909200
H	-8.084600	1.274200	-1.549200
H	-8.328300	1.499800	0.204700
H	-7.261000	4.191500	0.487200
H	-6.646500	2.913300	1.559700
H	-5.513300	3.966700	0.666000
H	-6.974600	4.012200	-2.021800
H	-5.238800	3.786300	-1.765000
H	-6.156000	2.603800	-2.736400
H	-8.687600	-2.772600	0.248700
H	-8.339300	-1.122800	0.787500
H	-8.165800	-1.558300	-0.934900
H	-7.115400	-4.353400	-0.306400
H	-6.454100	-3.342400	-1.604300
H	-5.371200	-4.062500	-0.381200
H	-7.316500	-3.505900	2.046700
H	-5.554600	-3.334000	2.022300
H	-6.575000	-1.948200	2.492900
H	8.011900	2.747000	1.998800
H	6.647300	1.866700	2.720800
H	7.762000	1.023500	1.627600
H	6.624200	4.463400	1.148700
H	5.208700	4.198500	0.120700
H	5.142500	3.775000	1.834300
H	7.901500	3.448200	-0.503900
H	7.885200	1.703500	-0.827500
H	6.628800	2.738500	-1.527700
H	8.606800	-2.695100	-0.656600
H	8.273500	-1.000800	-1.040700
H	8.032500	-1.604100	0.622200
H	7.286800	-3.188200	-2.605700

H	5.522200	-3.036900	-2.596000
H	6.534400	-1.593100	-2.867300
H	7.023700	-4.338700	-0.401100
H	6.352300	-3.505100	1.012500
H	5.279600	-4.051500	-0.305200

242

Ca-Bz_oss

N	4.014400	1.379900	0.502100
N	-4.154700	1.500000	-0.417800
C	5.320600	1.263900	0.351100
C	6.363400	2.396800	0.621200
C	3.252800	2.480200	0.926900
C	2.818400	2.569800	2.277500
C	1.905000	3.573800	2.620200
H	1.581100	3.664200	3.659500
C	1.397700	4.458200	1.674900
H	0.674600	5.222200	1.967200
C	1.834500	4.368900	0.356600
H	1.452000	5.071500	-0.386500
C	2.767600	3.408300	-0.034800
C	3.368300	1.672600	3.378900
H	3.980800	0.893900	2.893100
C	2.209600	0.979800	4.116700
H	1.494900	1.749000	4.452000
H	1.669300	0.387900	3.363400
C	4.297100	2.491500	4.303000
H	3.680300	3.098900	4.988900
C	5.330300	1.695200	5.095100
C	3.278900	3.413200	-1.466100
H	4.193500	2.800000	-1.492500
C	2.258100	2.745700	-2.398800
H	2.064400	1.714200	-2.054000
H	1.293700	3.268400	-2.283700
C	3.644200	4.843100	-1.932200
H	3.822800	5.473800	-1.046900
C	4.868900	4.930100	-2.834700
C	-5.469800	1.305600	-0.383100
C	-6.504600	2.465900	-0.580600
C	-3.441700	2.717300	-0.410000
C	-2.856500	3.237600	-1.600900
C	-1.937400	4.288300	-1.490100
H	-1.440800	4.654200	-2.388500
C	-1.636700	4.876200	-0.267900
H	-0.933800	5.709400	-0.215600
C	-2.243200	4.395100	0.887900
H	-2.017600	4.866000	1.846000
C	-3.121000	3.310200	0.850000
C	-3.171100	2.664000	-2.973400
H	-3.986500	1.940200	-2.822500
C	-1.986000	1.859000	-3.519200
H	-1.083800	2.493700	-3.559800
H	-1.745900	1.075500	-2.787000
C	-3.695900	3.691300	-4.005600
C	-2.698500	4.643400	-4.659900
H	-4.188900	3.110000	-4.802600
C	-3.692600	2.750300	2.140700
H	-4.565200	2.143300	1.866800
C	-2.723800	1.774100	2.834100
H	-2.308000	1.075700	2.090300
C	-1.551700	2.391000	3.576600
C	-4.181400	3.838000	3.107200
H	-4.810800	4.549300	2.551200
C	-4.963000	3.286100	4.291800
Ca	2.190600	-0.047600	-0.071200
Ca	-2.310700	-0.019900	-0.052000
N	4.111900	-1.406700	-0.584000
N	-4.204400	-1.444900	0.263700
C	5.932100	0.054500	-0.062100
H	7.009500	0.113600	-0.076000
C	5.418700	-1.197900	-0.448600
C	6.493300	-2.283100	-0.796500
C	3.400600	-2.596100	-0.806600
C	2.883300	-2.912500	-2.091200
C	1.961000	-3.962400	-2.207800
H	1.557400	-4.202100	-3.194600
C	1.553100	-4.708100	-1.111300
H	0.834800	-5.520500	-1.234500
C	2.088600	-4.422000	0.143500
H	1.792400	-5.027500	1.002100
C	3.003000	-3.384400	0.322200
C	3.276700	-2.175400	-3.363800
H	4.099300	-1.487900	-3.115700
C	2.089100	-1.327300	-3.843200
H	1.194500	-1.969200	-3.852900
H	1.884000	-0.570900	-3.068600
C	3.768300	-3.176800	-4.437400
H	4.207200	-4.051400	-3.930200
H	2.894300	-3.572800	-4.984300
C	3.598600	-3.105900	1.689400
H	4.498500	-2.501500	1.517300
C	2.702400	-2.232000	2.582800
H	2.310900	-1.373400	2.010600
C	1.526100	-2.923800	3.248500
C	4.046800	-4.386400	2.408200
C	4.878700	-4.118800	3.655300
H	3.171100	-4.998800	2.678000
C	-6.044000	0.033300	-0.165100
H	-7.117400	0.040900	-0.212500
C	-5.510000	-1.240400	0.124000
C	-6.571900	-2.370000	0.351800
C	-3.500700	-2.660900	0.350200
C	-2.999100	-3.138500	1.588900
C	-2.106200	-4.219500	1.582400
H	-1.715200	-4.586700	2.534400
C	-1.705300	-4.833400	0.405000
H	-1.006000	-5.671000	0.431500
C	-2.217100	-4.379100	-0.809300
H	-1.920700	-4.877500	-1.733900
C	-3.107000	-3.307100	-0.865700
C	-3.355600	-2.527800	2.936700
H	-4.154300	-1.787800	2.773200
C	-2.129000	-1.782200	3.481200
H	-1.272700	-2.474300	3.468200
H	-1.862300	-0.995500	2.760400
C	-3.868000	-3.610300	3.917300
H	-4.328400	-4.427400	3.339000
H	-3.001700	-4.070900	4.424200
C	-3.675700	-2.845400	-2.195900
H	-4.576300	-2.261800	-1.964900
C	-2.753100	-1.871000	-2.951700
H	-2.364200	-1.105100	-2.259300
C	-1.568000	-2.487300	-3.673200
C	-4.116200	-4.017200	-3.085100
H	-4.710300	-4.715200	-2.475900
C	-4.925800	-3.584500	-4.299900
H	2.772600	5.294800	-2.438100
H	4.835100	3.222600	3.680300
C	2.550100	0.099100	5.311300
H	4.626800	-5.001200	1.702900
H	3.334600	-1.772800	3.356400
C	4.788900	-2.636700	-5.433200
C	2.212200	-0.642700	-5.194800
C	-4.872000	-3.132100	4.960100
C	-2.236800	-1.163900	4.866200
H	-3.238800	-4.594600	-3.418100
C	2.626700	2.684400	-3.871100
H	-3.361800	-1.303300	-3.671800
C	-2.220800	1.205900	-4.873200
H	-4.496800	4.287800	-3.547400
H	-3.328700	4.429500	3.477500
H	-3.300700	1.136100	3.519600
C	7.926800	-1.855800	-0.443200
C	6.258600	-3.616200	-0.076200
C	6.450700	-2.536000	-2.306600
C	7.244500	2.573300	-0.631800
C	5.786300	3.777100	0.949200
C	7.247300	1.976900	1.808300
C	-7.956000	1.976700	-0.713000
C	-6.469400	3.435000	0.608000
C	-6.191800	3.256400	-1.852200
C	-8.016900	-1.913700	0.095000
C	-6.349600	-3.597400	-0.540600
C	-6.493000	-2.811600	1.816400
C	-0.085800	1.243800	-0.653600
C	0.001100	0.017700	-1.464700
C	-0.069500	-1.265500	-0.754600
C	-0.054600	-1.314500	0.615300
C	-0.052600	1.190700	0.714500
C	0.078400	-0.088700	1.418600
H	-0.067100	0.054900	-2.545700
H	-0.109700	-2.199300	-1.313800
H	-0.116600	-2.285100	1.104900
H	-0.073900	2.122500	1.278300
H	-0.031900	-0.126900	2.496800
H	-0.177200	2.214600	-1.136100
H	-0.857400	1.610400	3.917000
H	-0.978200	3.077100	2.938200
H	-1.879700	2.950700	4.465400
H	-5.367100	4.098000	4.915600
H	-5.812400	2.669900	3.955100
H	-4.338500	2.654200	4.942100
H	6.081700	2.367500	5.537900
H	4.889900	1.113200	5.913900
H	5.866300	0.984600	4.446300
H	1.666400	-0.482700	5.617200
H	3.352700	-0.619400	5.086100
H	2.866200	0.685800	6.186200
H	5.248300	-5.056500	4.097800
H	5.755100	-3.492300	3.421400
H	4.302500	-3.594100	4.433000
H	0.897100	-2.187900	3.768700
H	0.890300	-3.447000	2.519400
H	1.852500	-3.661700	3.997000
H	-5.189600	-3.966000	5.605400
H	-4.464200	-2.348800	5.612300
H	-5.774500	-2.723200	4.483200
H	-1.341100	-0.561200	5.083200
H	-3.110000	-0.499500	4.957400
H	-2.312400	-1.925300	5.656700
H	-1.394200	0.524300	-5.126400
H	-3.149600	0.613700	-4.873900
H	-2.299600	1.940200	-5.688800
H	-3.166100	5.142300	-5.523000
H	-2.367500	5.439000	-3.977800
H	-1.800500	4.126600	-5.031900
H	-5.289100	-4.455000	-4.867000
H	-5.805200	-2.990700	-4.001000
H	-4.335200	-2.965700	-4.993000
H	-0.928600	-1.702000	-4.098600
H	-0.943900	-3.091800	-2.999300
H	-1.887100	-3.134200	-4.504000
H	5.094800	-3.423800	-6.140200
H	4.402000	-1.796700	-6.024300
H	5.695900	-2.284800	-4.920900
H	1.334700	-0.002000	-5.375800
H	3.104300	-0.001300	-5.255000
H	2.258400	-1.364200	-6.024300
H	5.038100	5.964600	-3.172200

H	5.773600	4.608300	-2.297300
H	4.779400	4.299000	-3.729700
H	2.717200	3.686200	-4.316500
H	3.580400	2.156200	-4.027000
H	1.852700	2.145400	-4.435200
H	-8.600600	2.847100	-0.909100
H	-8.084600	1.274100	-1.549100
H	-8.328300	1.499800	0.204800
H	-7.261000	4.191500	0.487200
H	-6.646400	2.913300	1.559700
H	-5.513300	3.966600	0.665900
H	-6.974700	4.012100	-2.021800
H	-5.238900	3.786200	-1.765100
H	-6.156100	2.603700	-2.736400
H	-8.687500	-2.772800	0.248600
H	-8.339300	-1.122900	0.787300
H	-8.165800	-1.558500	-0.935100
H	-7.115200	-4.353600	-0.306300
H	-6.453800	-3.342600	-1.604300
H	-5.371000	-4.062600	-0.381000
H	-7.316500	-3.505900	2.046700
H	-5.554600	-3.333900	2.022400
H	-6.575100	-1.948100	2.492800
H	8.011900	2.747200	1.998700
H	6.647300	1.866800	2.720700
H	7.762000	1.023600	1.627500
H	6.624000	4.463600	1.148600
H	5.208500	4.198500	0.120700
H	5.142400	3.775000	1.834300
H	7.901400	3.448400	-0.503900
H	7.885100	1.703600	-0.827600
H	6.628600	2.738600	-1.527800
H	8.606900	-2.694900	-0.656600
H	8.273500	-1.000700	-1.040900
H	8.032600	-1.603700	0.622100
H	7.286800	-3.188200	-2.605600
H	5.522200	-3.037000	-2.595800
H	6.534300	-1.593200	-2.867300
H	7.023900	-4.338600	-0.400800
H	6.352500	-3.504900	1.012700
H	5.279800	-4.051400	-0.304900

242

Ca-Bz_t

N	4.020900	1.369100	0.416600
N	-4.111000	1.570400	-0.261200
C	5.329000	1.216500	0.308500
C	6.390200	2.336400	0.558100
C	3.285900	2.480400	0.872100
C	2.928500	2.570100	2.245000
C	2.028700	3.562000	2.648300
H	1.761500	3.640600	3.704800
C	1.455300	4.439100	1.735300
H	0.736900	5.190500	2.067800
C	1.823300	4.358500	0.397000
H	1.397300	5.063400	-0.319800
C	2.748200	3.413800	-0.054500
C	3.516600	1.656400	3.310800
H	4.196900	0.947200	2.810100
C	2.382200	0.846600	3.962400
H	1.602100	1.546700	4.303600
H	1.900100	0.259800	3.165200
C	4.355500	2.483800	4.309800
H	3.678100	3.014700	5.001600
C	5.411700	1.721100	5.104400
C	3.218100	3.506100	-1.499000
H	4.072600	2.819700	-1.616400
C	2.116300	3.052000	-2.473100
H	1.911000	1.984700	-2.294100
H	1.182600	3.575000	-2.203400
C	3.702600	4.946600	-1.809400
H	3.958800	5.450600	-0.864400
C	4.902400	5.037800	-2.743400
C	-5.426400	1.358900	-0.271100
C	-6.469600	2.519700	-0.405000
C	-3.401200	2.785100	-0.189100
C	-2.812400	3.358400	-1.355300
C	-1.902600	4.410900	-1.200200
H	-1.407700	4.818100	-2.081400
C	-1.610200	4.949800	0.046100
H	-0.917700	5.788900	0.134300
C	-2.210400	4.412300	1.179500
H	-1.988400	4.843400	2.157000
C	-3.081700	3.323900	1.095600
C	-3.127300	2.843100	-2.750900
H	-3.956300	2.129300	-2.632600
C	-1.955800	2.034100	-3.318100
H	-1.037700	2.646400	-3.319900
H	-1.743300	1.219400	-2.612700
C	-3.626700	3.916400	-3.747400
C	-2.607700	4.868100	-4.369300
H	-4.131400	3.374100	-4.564300
C	-3.664100	2.721300	2.362600
H	-4.529600	2.117900	2.057500
C	-2.704300	1.728000	3.042000
H	-2.313800	1.031500	2.283500
C	-1.514900	2.320000	3.778400
C	-4.171400	3.780800	3.351600
H	-4.795500	4.505100	2.806500
C	-4.966400	3.196300	4.511500
Ca	2.194300	-0.130900	0.059500
Ca	-2.255200	0.054900	-0.014200
N	4.097900	-1.509100	-0.431400
N	-4.120400	-1.430900	0.090400
C	5.927300	-0.014600	-0.058200
H	7.004500	0.038400	-0.083800
C	5.408400	-1.282200	-0.382300
C	6.475800	-2.384500	-0.701400
C	3.403300	-2.690900	-0.744000
C	2.962100	-2.930000	-2.073000
C	2.072100	-3.983400	-2.313900
H	1.722900	-4.160600	-3.333700
C	1.619500	-4.804700	-1.289700
H	0.926800	-5.620700	-1.504500
C	2.070200	-4.585800	0.009200
H	1.734900	-5.247900	0.809400
C	2.951700	-3.545000	0.309300
C	3.375800	-2.054300	-3.246100
H	4.258200	-1.472800	-2.944000
C	2.246700	-1.052300	-3.520800
H	1.295900	-1.605300	-3.562700
H	2.153900	-0.401500	-2.636800
C	3.746400	-2.901500	-4.483500
H	4.125600	-3.878000	-4.141700
H	2.830100	-3.131100	-5.055800
C	3.452200	-3.347500	1.728500
H	4.330500	-2.691800	1.655500
C	2.462700	-2.588400	2.628200
H	2.103300	-1.693400	2.095100
C	1.249800	-3.361600	3.114900
C	3.914700	-4.661400	2.376100
C	4.644500	-4.464100	3.697900
H	3.053000	-5.330800	2.531500
C	-5.978600	0.067400	-0.173300
H	-7.055600	0.048200	-0.230500
C	-5.420600	-1.225100	-0.020800
C	-6.511700	-2.345100	0.003200
C	-3.416200	-2.648600	0.116800
C	-3.015200	-3.218900	1.348600
C	-2.124000	-4.296200	1.336200
H	-1.810000	-4.738300	2.285100
C	-1.644300	-4.825000	0.143400
H	-0.945400	-5.663000	0.155900
C	-2.091500	-4.301100	-1.067400
H	-1.752900	-4.753900	-2.001000
C	-2.980500	-3.223900	-1.109700
C	-3.577800	-2.738900	2.673100
H	-4.409400	-2.051600	2.446800
C	-2.511200	-1.937200	3.432500
H	-1.612800	-2.568500	3.532200
H	-2.197700	-1.098000	2.793600
C	-4.136400	-3.918500	3.504600
H	-4.363900	-4.758100	2.828400
H	-3.346500	-4.295900	4.177600
C	-3.545500	-2.722600	-2.426300
H	-4.400100	-2.080000	-2.170400
C	-2.585700	-1.812400	-3.212300
H	-2.205000	-1.028800	-2.536000
C	-1.401000	-2.492000	-3.878400
C	-4.103000	-3.873700	-3.278100
H	-4.793900	-4.459900	-2.650200
C	-4.826300	-3.417100	-4.538100
H	2.860200	5.528700	-2.222100
H	4.863400	3.278500	3.741200
C	2.747100	-0.077800	5.115200
H	4.570900	-5.191500	1.669300
H	3.015100	-2.202300	3.495800
C	4.785600	-2.284400	-5.412700
C	2.365500	-0.171200	-4.751200
C	-5.390300	-3.595400	4.307500
C	-2.909700	-1.405500	4.800300
H	-3.295900	-4.572500	-3.552400
C	2.388400	3.270400	-3.953300
H	-3.170200	-1.275900	-3.975300
C	-2.191000	1.438900	-4.698000
H	-4.415400	4.513600	-3.268300
H	-3.325900	4.365200	3.749400
H	-3.285900	1.100800	3.732600
C	7.919500	-1.923700	-0.443900
C	6.267100	-3.641100	0.150700
C	6.393400	-2.784300	-2.179000
C	7.234200	2.500200	-0.721900
C	5.835200	3.719800	0.904700
C	7.303900	1.914200	1.721600
C	-7.919400	2.028900	-0.543100
C	-6.428500	3.438900	0.821900
C	-6.169400	3.364200	-1.645400
C	-7.267500	-2.337600	-1.338100
C	-6.006300	-3.773900	0.225400
C	-7.496900	-2.041600	1.149700
C	0.026100	1.267500	-0.496300
C	0.001000	0.119800	-1.363500
C	-0.054700	-1.202100	-0.803200
C	-0.094600	-1.374700	0.615300
C	-0.008600	1.093000	0.921900
C	-0.065100	-0.228700	1.481000
H	0.042700	0.249700	-2.442200
H	-0.043600	-2.082300	-1.441200
H	-0.177100	-2.378800	1.021600
H	0.046400	1.969100	1.562700
H	-0.095800	-0.362400	2.561200
H	0.027500	2.275300	-0.900900
H	-0.845900	1.521800	4.132600
H	-0.919800	2.980200	3.131800
H	-1.826500	2.901200	4.659800
H	-5.385000	3.990900	5.147900
H	-5.807000	2.582900	4.148100
H	-4.347200	2.553400	5.156100
H	6.097900	2.421500	5.605500

H	4.983900	1.073700	5.879800
H	6.019700	1.079700	4.447400
H	1.906300	-0.749800	5.347400
H	3.619300	-0.706900	4.880900
H	2.977900	0.479900	6.034600
H	5.033600	-5.418500	4.084300
H	5.500000	-3.778800	3.581500
H	3.989400	-4.039500	4.474300
H	0.576400	-2.701000	3.680400
H	0.670100	-3.785800	2.282900
H	1.532800	-4.188400	3.784100
H	-5.716000	-4.464200	4.900600
H	-5.241300	-2.756600	5.001600
H	-6.221900	-3.323500	3.639500
H	-2.117500	-0.760200	5.210700
H	-3.832400	-0.806200	4.750800
H	-3.078300	-2.215500	5.526100
H	-1.386600	0.735300	-4.962900
H	-3.141100	0.883200	-4.733700
H	-2.228300	2.205300	-5.486500
H	-3.058200	5.394700	-5.225100
H	-2.270300	5.642000	-3.666100
H	-1.714200	4.345800	-4.744400
H	-5.301100	-4.266600	-5.052300
H	-5.617400	-2.686500	-4.303400
H	-4.143700	-2.939300	-5.257400
H	-0.777900	-1.753300	-4.404000
H	-0.757900	-3.006400	-3.150200
H	-1.721300	-3.235900	-4.624000
H	4.995600	-2.953900	-6.261500
H	4.465500	-1.319200	-5.826600
H	5.736000	-2.115100	-4.885600
H	1.528800	0.542100	-4.787100
H	3.297400	0.413600	-4.750700
H	2.331600	-0.756400	-5.682200
H	5.156400	6.087700	-2.957700
H	5.788900	4.572700	-2.286900
H	4.728400	4.535500	-3.704800
H	2.493800	4.337200	-4.200900
H	3.303600	2.756500	-4.283900
H	1.555100	2.880700	-4.557000
H	-8.574400	2.904300	-0.671000
H	-8.058800	1.381500	-1.421100
H	-8.268800	1.490000	0.349300
H	-7.219200	4.201000	0.736000
H	-6.602200	2.878500	1.751900
H	-5.471400	3.966200	0.897900
H	-6.957400	4.121900	-1.779400
H	-5.218500	3.895300	-1.541000
H	-6.134400	2.748500	-2.555900
H	-8.051800	-3.111200	-1.329700
H	-7.745800	-1.371000	-1.544900
H	-6.587100	-2.560600	-2.172600
H	-6.876000	-4.449400	0.231400
H	-5.326800	-4.116300	-0.562200
H	-5.491600	-3.893500	1.183900
H	-8.218700	-2.867700	1.249800
H	-6.963900	-1.938700	2.106400
H	-8.067700	-1.118100	0.987400
H	8.068900	2.687700	1.896100
H	6.725500	1.800800	2.648100
H	7.820000	0.964300	1.529200
H	6.683300	4.405700	1.056200
H	5.216000	4.136000	0.105700
H	5.238400	3.722200	1.822600
H	7.915100	3.359300	-0.612600
H	7.845700	1.615500	-0.942100
H	6.592000	2.684300	-1.595500
H	8.598100	-2.767600	-0.642200
H	8.224800	-1.101000	-1.106300
H	8.075700	-1.610400	0.598600
H	7.215400	-3.475900	-2.423000
H	5.453300	-3.297200	-2.408700
H	6.488100	-1.904000	-2.830600
H	7.055600	-4.373700	-0.082500
H	6.331300	-3.414200	1.224300
H	5.304300	-4.120700	-0.054200

242

Sr-Bz_s

N	4.293000	1.481700	0.250800
N	-4.481300	1.513600	-0.254700
C	5.596900	1.344500	0.123800
C	6.630000	2.510200	0.242400
C	3.493400	2.603600	0.504500
C	3.013900	2.855900	1.822300
C	2.080500	3.881100	2.013200
H	1.734800	4.102200	3.025300
C	1.574600	4.624500	0.953100
H	0.836700	5.408300	1.133100
C	2.022700	4.355900	-0.335700
H	1.626700	4.933600	-1.174700
C	2.981100	3.370500	-0.582400
C	3.520000	2.090400	3.037600
H	4.103300	1.231800	2.662800
C	2.323100	1.550200	3.842800
H	1.620700	2.377700	4.027500
H	1.779400	0.856000	3.182800
C	4.481000	2.976100	3.861100
H	3.890600	3.673600	4.481600
C	5.494500	2.229900	4.723500
C	3.471700	3.171000	-2.006900
H	4.361200	2.521800	-1.966800
C	2.402300	2.444100	-2.840100
H	2.148600	1.474000	-2.374500
H	1.466300	3.024800	-2.784600
C	3.885000	4.516400	-2.653900
H	4.094300	5.246300	-1.856000
C	5.101000	4.444000	-3.568700
C	-5.784600	1.294700	-0.150200
C	-6.831500	2.449100	-0.283400
C	-3.736600	2.701600	-0.345700
C	-3.203600	3.135400	-1.600200
C	-2.232000	4.143400	-1.609200
H	-1.780500	4.432100	-2.558300
C	-1.815800	4.778800	-0.447200
H	-1.070200	5.574600	-0.490200
C	-2.356000	4.380700	0.770500
H	-2.035600	4.881800	1.685400
C	-3.282700	3.338300	0.855000
C	-3.601000	2.494100	-2.920400
H	-4.381700	1.752600	-2.686000
C	-2.419300	1.715700	-3.519600
H	-1.544900	2.380700	-3.621700
H	-2.094700	0.950700	-2.795200
C	-4.229100	3.462000	-3.952000
C	-3.320100	4.469900	-4.648200
H	-4.707600	2.834700	-4.722400
C	-3.750500	2.866700	2.220200
H	-4.629200	2.228400	2.054000
C	-2.716100	1.961500	2.919100
H	-2.369800	1.176800	2.225700
C	-1.478700	2.631400	3.492900
C	-4.190400	4.022200	3.132400
H	-4.875200	4.674900	2.570700
C	-4.867400	3.561900	4.416300
Sr	2.337100	-0.049200	-0.212400
Sr	-2.449600	-0.006900	-0.045900
N	4.425000	-1.485100	-0.393300
N	-4.474200	-1.507100	0.264500
C	6.215500	0.089900	-0.122800
H	7.292200	0.160200	-0.156300
C	5.724200	-1.217600	-0.312700
C	6.807000	-2.332500	-0.486300
C	3.735000	-2.704800	-0.384400
C	3.169800	-3.235000	-1.579800
C	2.291900	-4.323800	-1.497000
H	1.858300	-4.721800	-2.418000
C	1.965400	-4.914000	-0.284200
H	1.286200	-5.767800	-0.248300
C	2.520400	-4.402700	0.886600
H	2.273200	-4.869300	1.841900
C	3.382600	-3.305400	0.868700
C	3.451600	-2.660700	-2.960300
H	4.246100	-1.905000	-2.854100
C	2.186500	-1.951800	-3.472200
H	1.338900	-2.651800	-3.390800
H	1.931200	-1.129300	-2.780800
C	3.941600	-3.761200	-3.932000
H	4.436200	-4.554400	-3.349300
H	3.064700	-4.247800	-4.394800
C	3.904200	-2.730000	2.170200
H	4.758100	-2.087400	1.915700
C	2.883300	-1.793900	2.842300
H	2.498300	-1.063600	2.111300
C	1.686100	-2.444500	3.513500
C	4.417100	-3.801500	3.142700
C	5.143400	-3.223500	4.350000
H	3.583700	-4.434900	3.487800
C	-6.331700	0.008000	0.069400
H	-7.406300	0.012400	0.105600
C	-5.780500	-1.282700	0.249500
C	-6.820900	-2.431100	0.468000
C	-3.748400	-2.708600	0.311900
C	-3.174200	-3.166200	1.532400
C	-2.271200	-4.236300	1.503700
H	-1.829800	-4.578500	2.443000
C	-1.922800	-4.872100	0.320400
H	-1.219900	-5.707100	0.325900
C	-2.484900	-4.430000	-0.873900
H	-2.224500	-4.937400	-1.804300
C	-3.376900	-3.356200	-0.911300
C	-3.455100	-2.522200	2.882300
H	-4.274700	-1.798500	2.749600
C	-2.202900	-1.746200	3.322100
H	-1.337600	-2.426600	3.273100
H	-1.981900	-0.971000	2.569900
C	-3.888300	-3.575600	3.929600
H	-4.373800	-4.416200	3.409400
H	-2.986300	-4.007900	4.397600
C	-3.922900	-2.878400	-2.244000
H	-4.781000	-2.229200	-2.020700
C	-2.924700	-1.986000	-3.007600
H	-2.529100	-1.202800	-2.338900
C	-1.734200	-2.675600	-3.653600
C	-4.437900	-4.028500	-3.122900
H	-5.086800	-4.674800	-2.512900
C	-5.201400	-3.558800	-4.353700
H	3.024200	4.932700	-3.206400
H	5.036100	3.617300	3.159300
C	2.604100	0.879500	5.180600
H	5.091900	-4.478200	2.596800
H	3.421000	-1.179200	3.577000
C	4.901000	-3.296300	-5.021500
C	2.225500	-1.388200	-4.883200
C	-4.832300	-3.071500	5.015100
C	-2.231200	-1.086500	4.691500
H	-3.599400	-4.670700	-3.437500
C	2.749600	2.182200	-4.296300

H	-3.480700	-1.432600	-3.778100
C	-2.704600	1.040800	-4.853300
H	-5.052700	4.010300	-3.476200
H	-3.325000	4.656400	3.384400
H	-3.231600	1.409800	3.719000
C	8.248400	-1.804400	-0.442900
C	6.694900	-3.398000	0.610900
C	6.614300	-3.004100	-1.849200
C	7.522300	2.523000	-1.014600
C	6.023300	3.909900	0.370800
C	7.506300	2.269300	1.483400
C	-8.290800	1.971600	-0.258300
C	-6.669400	3.460000	0.857500
C	-6.619200	3.179600	-1.610800
C	-8.282000	-1.959000	0.427400
C	-6.681400	-3.520100	-0.601900
C	-6.591700	-3.060000	1.845800
C	-0.084000	1.232300	-0.822200
C	-0.104100	-0.046900	-1.575600
C	-0.088700	-1.282100	-0.801700
C	-0.024700	-1.275800	0.571800
C	-0.021000	1.238700	0.549000
C	0.048200	0.003300	1.319000
H	-0.134700	-0.059300	-2.661700
H	-0.122800	-2.245100	-1.313400
H	-0.025400	-2.226800	1.104000
H	0.006500	2.198600	1.066300
H	0.029500	0.020200	2.404400
H	-0.136500	2.182400	-1.352100
H	-0.796200	1.875800	3.906900
H	-0.918600	3.187200	2.728000
H	-1.727600	3.330900	4.305200
H	-5.247300	4.418200	4.994100
H	-5.722000	2.900400	4.200100
H	-4.179900	3.005200	5.071700
H	6.257900	2.920800	5.113800
H	5.037700	1.726100	5.584300
H	6.017300	1.457500	4.137400
H	1.688100	0.403400	5.564300
H	3.374000	0.096800	5.109800
H	2.935000	1.600600	5.942700
H	5.576200	-4.020100	4.974300
H	5.966100	-2.559500	4.038000
H	4.473100	-2.632200	4.993000
H	0.998500	-1.675400	3.893600
H	1.119600	-3.075300	2.813700
H	1.982400	-3.070700	4.368700
H	-5.103600	-3.886000	5.704800
H	-4.394200	-2.263800	5.615900
H	-5.765600	-2.685700	4.579400
H	-1.342200	-0.450600	4.824300
H	-3.117100	-0.447000	4.823200
H	-2.226600	-1.824900	5.506900
H	-1.889700	0.352500	-5.125000
H	-3.635700	0.453800	-4.814300
H	-2.809700	1.765200	-5.674700
H	-3.852600	4.936700	-5.491500
H	-3.011000	5.284100	-3.977900
H	-2.408500	4.007100	-5.057300
H	-5.626300	-4.410100	-4.907100
H	-6.034900	-2.893600	-4.074500
H	-4.559000	-3.002100	-5.053400
H	-1.056200	-1.931200	-4.095900
H	-1.150700	-3.256100	-2.925000
H	-2.041500	-3.359800	-4.459000
H	5.208400	-4.141700	-5.656900
H	4.460300	-2.534500	-5.678000
H	5.813600	-2.864300	-4.585700
H	1.309300	-0.811700	-5.086000
H	3.080300	-0.711800	-5.031500
H	2.284600	-2.179800	-5.644800
H	5.304200	5.421500	-4.033300
H	5.999400	4.157300	-3.002000
H	4.977800	3.710800	-4.377800
H	2.874100	3.116000	-4.863900
H	3.681200	1.602500	-4.390900
H	1.948400	1.607600	-4.782400
H	-8.947800	2.844700	-0.392700
H	-8.512100	1.263600	-1.070000
H	-8.563900	1.502600	0.697900
H	-7.454400	4.229300	0.784000
H	-6.763600	2.976300	1.840800
H	-5.702300	3.971800	0.805100
H	-7.402400	3.941100	-1.751500
H	-5.653200	3.694200	-1.625400
H	-6.663000	2.485500	-2.463000
H	-8.936700	-2.829200	0.589000
H	-8.508800	-1.228000	1.216800
H	-8.552800	-1.519500	-0.543500
H	-7.453700	-4.289500	-0.444600
H	-6.821100	-3.108600	-1.611900
H	-5.707400	-4.018800	-0.554100
H	-7.370200	-3.811400	2.051500
H	-5.622400	-3.565500	1.901400
H	-6.639300	-2.297200	2.636000
H	8.262800	3.065700	1.570500
H	6.899500	2.280100	2.398000
H	8.030200	1.304800	1.439400
H	6.842700	4.638000	0.475900
H	5.441500	4.195400	-0.511600
H	5.371900	4.015900	1.244900
H	8.159900	3.421300	-1.009500
H	8.183000	1.648400	-1.075400
H	6.914200	2.542000	-1.930600
H	8.938700	-2.651100	-0.579800
H	8.452900	-1.081500	-1.245900
H	8.491800	-1.334600	0.521200
H	7.418200	-3.735400	-2.028700
H	5.661700	-3.541200	-1.900900
H	6.644400	-2.260000	-2.658200
H	7.491700	-4.146800	0.477300
H	6.816100	-2.956100	1.610400
H	5.736600	-3.927700	0.572000

242

Sr-Bz_oss

N	4.293100	1.481800	0.251100
N	-4.481400	1.513600	-0.254500
C	5.596900	1.344600	0.123600
C	6.630100	2.510300	0.242300
C	3.493500	2.603400	0.505200
C	3.014000	2.855200	1.823200
C	2.080600	3.880400	2.014500
H	1.735100	4.101100	3.026700
C	1.574600	4.624100	0.954700
H	0.836800	5.407900	1.135100
C	2.022600	4.356000	-0.334300
H	1.626500	4.934000	-1.173000
C	2.981000	3.370700	-0.581400
C	3.520200	2.089200	3.038100
H	4.103200	1.230500	2.662800
C	2.323500	1.549100	3.843600
H	1.621300	2.376800	4.028500
H	1.779500	0.855000	3.183600
C	4.481900	2.974200	3.861500
H	3.892000	3.671500	4.482800
C	5.495700	2.227200	4.722900
C	3.471300	3.171600	-2.006100
H	4.360800	2.522500	-1.966400
C	2.401600	2.444800	-2.839100
H	2.148200	1.474600	-2.373500
H	1.465600	3.025500	-2.783200
C	3.884200	4.517300	-2.652800
H	4.093700	5.247000	-1.854800
C	5.100000	4.445300	-3.568000
C	-5.784700	1.294600	-0.150400
C	-6.831600	2.449000	-0.283500
C	-3.736700	2.701700	-0.345100
C	-3.203600	3.135800	-1.599400
C	-2.232100	4.143900	-1.608000
H	-1.780500	4.432900	-2.556900
C	-1.816100	4.779100	-0.445700
H	-1.070500	5.575000	-0.488400
C	-2.356400	4.380600	0.771800
H	-2.036300	4.881500	1.686900
C	-3.283000	3.338000	0.855900
C	-3.600800	2.494900	-2.919800
H	-4.381500	1.753500	-2.685800
C	-2.419000	1.716600	-3.519100
H	-1.544600	2.381700	-3.620800
H	-2.094500	0.951400	-2.794800
C	-4.228600	3.463100	-3.951300
C	-3.319500	4.471200	-4.647200
H	-4.707100	2.836100	-4.721900
C	-3.750900	2.866000	2.220900
H	-4.629600	2.227700	2.054400
C	-2.716400	1.960400	2.919300
H	-2.370200	1.176000	2.225500
C	-1.479100	2.630200	3.493300
C	-4.190800	4.021000	3.133600
H	-4.875700	4.673900	2.572100
C	-4.867700	3.560000	4.417300
Sr	2.337100	-0.049000	-0.212400
Sr	-2.449800	-0.007000	-0.045800
N	4.425100	-1.485000	-0.393800
N	-4.474300	-1.507200	0.264200
C	6.215500	0.090100	-0.123600
H	7.292200	0.160500	-0.157500
C	5.724300	-1.217400	-0.313400
C	6.807100	-2.332300	-0.487200
C	3.735100	-2.704700	-0.384900
C	3.169700	-3.234600	-1.580400
C	2.291800	-4.323500	-1.497700
H	1.858000	-4.721200	-2.418700
C	1.965400	-4.913800	-0.284900
H	1.286100	-5.767600	-0.249000
C	2.520500	-4.402700	0.885900
H	2.273400	-4.869500	1.841100
C	3.382800	-3.305400	0.868100
C	3.451400	-2.660100	-2.960700
H	4.246000	-1.904500	-2.854500
C	2.186400	-1.951000	-3.472400
H	1.338800	-2.651000	-3.391200
H	1.931100	-1.128600	-2.780700
C	3.941200	-3.760500	-3.932700
H	4.435800	-4.553800	-3.350300
H	3.064100	-4.246900	-4.395500
C	3.904400	-2.730200	2.169700
H	4.758300	-2.087600	1.915200
C	2.883500	-1.794200	2.841900
H	2.498700	-1.063700	2.111000
C	1.686400	-2.445000	3.513000
C	4.417400	-3.801800	3.142000
C	5.143500	-3.223900	4.349500
H	3.584100	-4.435400	3.487000
C	-6.331800	0.007900	0.068800
H	-7.406400	0.012200	0.104700
C	-5.780600	-1.282900	0.248900
C	-6.821000	-2.431300	0.467200

C	-3.748300	-2.708500	0.311400
C	-3.174000	-3.166200	1.531900
C	-2.271000	-4.236300	1.503100
H	-1.829500	-4.578500	2.442300
C	-1.922400	-4.871900	0.319800
H	-1.219400	-5.706700	0.325200
C	-2.484500	-4.429700	-0.874500
H	-2.224100	-4.937000	-1.805000
C	-3.376700	-3.356000	-0.911800
C	-3.455000	-2.522500	2.882000
H	-4.274500	-1.798600	2.749200
C	-2.202700	-1.746900	3.322200
H	-1.337600	-2.427500	3.273200
H	-1.981400	-0.971600	2.570200
C	-3.888700	-3.576100	3.928800
H	-4.374100	-4.416600	3.408400
H	-2.986800	-4.008700	4.396900
C	-3.922600	-2.878100	-2.244500
H	-4.780900	-2.229000	-2.021200
C	-2.924400	-1.985500	-3.007800
H	-2.528700	-1.202600	-2.338700
C	-1.733900	-2.674900	-3.654100
C	-4.437500	-4.028100	-3.123600
H	-5.086400	-4.674500	-2.513800
C	-5.200900	-3.558100	-4.354400
H	3.023200	4.933600	-3.205000
H	5.036700	3.615700	3.159800
C	2.604700	0.878400	5.181300
H	5.092400	-4.478300	2.596000
H	3.421300	-1.179600	3.576700
C	4.900300	-3.295500	-5.022400
C	2.225400	-1.386900	-4.883200
C	-4.832800	-3.072200	5.014300
C	-2.231300	-1.087600	4.691800
H	-3.598900	-4.670200	-3.438300
C	2.748500	2.183300	-4.295400
H	-3.480400	-1.431700	-3.778100
C	-2.704100	1.042200	-4.853000
H	-5.052300	4.011400	-3.475500
H	-3.325500	4.655200	3.385900
H	-3.231900	1.408300	3.719000
C	8.248500	-1.804100	-0.444400
C	6.695400	-3.397500	0.610300
C	6.614000	-3.004400	-1.849800
C	7.522100	2.523500	-1.014800
C	6.023300	3.909900	0.371200
C	7.506600	2.269200	1.483000
C	-8.290900	1.971500	-0.259400
C	-6.670100	3.459200	0.858100
C	-6.618800	3.180300	-1.610400
C	-8.282100	-1.959400	0.426300
C	-6.681200	-3.520400	-0.602600
C	-6.591900	-3.060000	1.845100
C	-0.084200	1.232700	-0.821400
C	-0.104100	-0.046300	-1.575300
C	-0.088700	-1.281800	-0.801900
C	-0.024700	-1.276000	0.571600
C	-0.021100	1.238500	0.549700
C	0.048300	0.002900	1.319300
H	-0.134700	-0.058300	-2.661400
H	-0.122900	-2.244500	-1.313900
H	-0.025200	-2.227200	1.103400
H	0.006300	2.198200	1.067400
H	0.029700	0.019400	2.404600
H	-0.136800	2.182900	-1.351000
H	-0.796400	1.874400	3.907000
H	-0.919100	3.186500	2.728800
H	-1.728200	3.329200	4.306000
H	-5.247700	4.416000	4.995500
H	-5.722300	2.898600	4.200800

H	-4.180100	3.003000	5.072400
H	6.259700	2.917700	5.112900
H	5.039400	1.723400	5.583900
H	6.017600	1.454800	4.136100
H	1.688700	0.402400	5.565100
H	3.374400	0.095600	5.110500
H	2.935800	1.599500	5.943300
H	5.576500	-4.020500	4.973700
H	5.966100	-2.559700	4.037600
H	4.473100	-2.632900	4.992600
H	0.998900	-1.675900	3.893500
H	1.119700	-3.075300	2.812900
H	1.982700	-3.071600	4.367800
H	-5.104100	-3.886800	5.703900
H	-4.394800	-2.264500	5.615200
H	-5.766200	-2.686400	4.578600
H	-1.342200	-0.451800	4.824900
H	-3.117100	-0.448000	4.823500
H	-2.226900	-1.826200	5.507000
H	-1.889200	0.353800	-5.124700
H	-3.635300	0.455300	-4.814500
H	-2.808900	1.766800	-5.674200
H	-3.851800	4.938200	-5.490400
H	-3.010400	5.285200	-3.976600
H	-2.407800	4.008400	-5.056200
H	-5.625600	-4.409400	-4.908100
H	-6.034500	-2.893100	-4.075200
H	-4.558400	-3.001200	-5.053900
H	-1.055900	-1.930500	-4.096100
H	-1.150500	-3.255700	-2.925600
H	-2.041300	-3.358800	-4.459600
H	5.207500	-4.140800	-5.658000
H	4.459600	-2.533500	-5.678600
H	5.813200	-2.863800	-4.586700
H	1.309400	-0.810100	-5.085800
H	3.080400	-0.710600	-5.031300
H	2.284400	-2.178200	-5.645100
H	5.302800	5.423000	-4.032500
H	5.998600	4.158700	-3.001700
H	4.976600	3.712300	-4.377200
H	2.872700	3.117200	-4.862900
H	3.680200	1.603700	-4.390500
H	1.947200	1.608700	-4.781400
H	-8.947900	2.844500	-0.393800
H	-8.511700	1.263600	-1.071300
H	-8.564500	1.502200	0.696600
H	-7.455100	4.228500	0.784800
H	-6.764500	2.975000	1.841000
H	-5.703000	3.971200	0.806300
H	-7.402100	3.941900	-1.750900
H	-5.652800	3.694900	-1.624400
H	-6.662400	2.486700	-2.463000
H	-8.936700	-2.829600	0.587700
H	-8.509100	-1.228400	1.215600
H	-8.552700	-1.519800	-0.544600
H	-7.453300	-4.290000	-0.445300
H	-6.820900	-3.109000	-1.612600
H	-5.707000	-4.018900	-0.554700
H	-7.370400	-3.811500	2.050800
H	-5.622600	-3.565500	1.900900
H	-6.639600	-2.297200	2.635200
H	8.263100	3.065600	1.570100
H	6.900100	2.279700	2.397800
H	8.030600	1.304800	1.438700
H	6.842600	4.638100	0.476400
H	5.441400	4.195600	-0.511200
H	5.371900	4.015700	1.245300
H	8.159800	3.421800	-1.009600
H	8.182900	1.649000	-1.076100
H	6.913900	2.542800	-1.930800

H	8.938800	-2.650800	-0.581500
H	8.452600	-1.081300	-1.247700
H	8.492300	-1.334100	0.519400
H	7.417900	-3.735700	-2.029200
H	5.661500	-3.541500	-1.901100
H	6.644000	-2.260600	-2.659000
H	7.492300	-4.146200	0.476900
H	6.816600	-2.955300	1.609700
H	5.737100	-3.927300	0.571700

242

Sr-Bz_t

N	4.311200	1.454800	0.269300
N	-4.480100	1.530900	-0.229500
C	5.616900	1.304100	0.177000
C	6.661300	2.453700	0.345600
C	3.526600	2.589100	0.520900
C	3.038900	2.844800	1.834800
C	2.125300	3.886900	2.024600
H	1.775600	4.109500	3.035300
C	1.636100	4.637800	0.962400
H	0.908500	5.431800	1.138800
C	2.089800	4.365500	-0.323200
H	1.710400	4.953800	-1.162600
C	3.042000	3.372200	-0.566400
C	3.507500	2.058700	3.052100
H	4.111600	1.211000	2.685600
C	2.280700	1.497700	3.796900
H	1.559300	2.315400	3.948400
H	1.773500	0.801300	3.111900
C	4.427600	2.934100	3.931100
H	3.808100	3.617000	4.539300
C	5.408700	2.178600	4.822300
C	3.571100	3.203200	-1.981400
H	4.447000	2.536200	-1.932600
C	2.518000	2.523500	-2.873600
H	2.250500	1.542100	-2.444000
H	1.584500	3.108600	-2.815800
C	4.029500	4.561500	-2.569900
H	4.223400	5.261900	-1.742400
C	5.274400	4.497100	-3.445700
C	-5.785100	1.314200	-0.145100
C	-6.827900	2.475100	-0.256100
C	-3.728500	2.716600	-0.273900
C	-3.176200	3.184600	-1.508100
C	-2.202300	4.189800	-1.474200
H	-1.737100	4.505200	-2.408000
C	-1.800200	4.787700	-0.287600
H	-1.051900	5.581800	-0.297100
C	-2.356300	4.354300	0.910800
H	-2.043800	4.825100	1.844400
C	-3.287600	3.313100	0.952000
C	-3.574200	2.596000	-2.853300
H	-4.367800	1.859700	-2.648800
C	-2.406600	1.818900	-3.481100
H	-1.520300	2.471000	-3.560500
H	-2.096900	1.028400	-2.780900
C	-4.182300	3.611900	-3.850700
C	-3.252000	4.619600	-4.518900
H	-4.680200	3.022500	-4.638600
C	-3.784400	2.807000	2.295400
H	-4.669900	2.188500	2.095300
C	-2.779800	1.862800	2.985500
H	-2.446300	1.094200	2.270200
C	-1.534900	2.493600	3.586400
C	-4.221800	3.942300	3.233800
H	-4.886800	4.621500	2.679500
C	-4.927200	3.457000	4.493000
Sr	2.330400	-0.067400	-0.171000

Sr	-2.446400	0.003300	-0.059400
N	4.420300	-1.496600	-0.381200
N	-4.481900	-1.496300	0.207700
C	6.228000	0.047200	-0.075700
H	7.305800	0.105300	-0.089400
C	5.722900	-1.249500	-0.296100
C	6.791900	-2.373300	-0.498100
C	3.712300	-2.704700	-0.411400
C	3.144100	-3.185400	-1.626200
C	2.245000	-4.258800	-1.582200
H	1.806400	-4.618500	-2.516500
C	1.899400	-4.877600	-0.389100
H	1.201400	-5.716600	-0.382700
C	2.457300	-4.414700	0.800300
H	2.193500	-4.905700	1.738800
C	3.342700	-3.335600	0.821600
C	3.455600	-2.575500	-2.985700
H	4.284600	-1.863100	-2.852200
C	2.230600	-1.786100	-3.477800
H	1.342000	-2.432200	-3.393100
H	2.033900	-0.960600	-2.773000
C	3.898200	-3.664100	-3.992500
H	4.354300	-4.496200	-3.433200
H	3.002500	-4.096700	-4.472600
C	3.887100	-2.822800	2.141400
H	4.762100	-2.202500	1.904900
C	2.907500	-1.874900	2.856800
H	2.567500	-1.099100	2.152700
C	1.678300	-2.505100	3.487600
C	4.366600	-3.948100	3.069400
C	5.118500	-3.446200	4.294900
H	3.513300	-4.565700	3.393700
C	-6.337200	0.023400	0.032300
H	-7.412200	0.028400	0.056600
C	-5.788200	-1.271800	0.184700
C	-6.832300	-2.423800	0.366500
C	-3.755300	-2.697800	0.227900
C	-3.190500	-3.189800	1.440000
C	-2.292100	-4.263200	1.387500
H	-1.858600	-4.633900	2.319600
C	-1.938100	-4.866900	0.189300
H	-1.240300	-5.706000	0.177000
C	-2.488300	-4.389300	-0.996800
H	-2.221600	-4.871100	-1.939000
C	-3.375900	-3.311200	-1.011000
C	-3.492100	-2.594800	2.809400
H	-4.311100	-1.868100	2.690500
C	-2.252000	-1.832200	3.304400
H	-1.381300	-2.505000	3.241200
H	-2.027400	-1.029900	2.585300
C	-3.939400	-3.689500	3.808000
H	-4.411200	-4.511300	3.246700
H	-3.043300	-4.136500	4.273700
C	-3.922900	-2.803400	-2.333500
H	-4.784500	-2.164300	-2.094800
C	-2.933300	-1.887600	-3.080900
H	-2.545300	-1.122800	-2.389500
C	-1.737900	-2.556600	-3.739200
C	-4.432500	-3.936300	-3.237900
H	-5.074800	-4.601000	-2.641200
C	-5.202700	-3.444200	-4.455800
H	3.194900	5.010800	-3.136600
H	5.007000	3.591100	3.264300
C	2.502900	0.820900	5.142200
H	5.014400	-4.625400	2.492100
H	3.469000	-1.319800	3.621500
C	4.882900	-3.209400	-5.063600
C	2.288900	-1.207000	-4.881600
C	-4.904400	-3.234600	4.896800
C	-2.309700	-1.230600	4.699400

H	-3.590500	-4.565300	-3.569200
C	2.901900	2.325400	-4.330700
H	-3.494600	-1.322500	-3.839100
C	-2.706900	1.193400	-4.835400
H	-4.989500	4.164800	-3.352500
H	-3.351400	4.555700	3.518100
H	-3.317100	1.303200	3.765600
C	8.239500	-1.866000	-0.422000
C	6.656800	-3.479000	0.555700
C	6.603900	-2.992300	-1.886900
C	7.591600	2.476100	-0.883500
C	6.072200	3.860100	0.482100
C	7.495600	2.177700	1.608000
C	-8.288000	1.999500	-0.271700
C	-6.683700	3.446700	0.920800
C	-6.593400	3.251500	-1.553200
C	-8.292700	-1.950500	0.312600
C	-6.679200	-3.496000	-0.718100
C	-6.622900	-3.075300	1.736500
C	-0.055100	1.228600	-0.815200
C	-0.089000	-0.005500	-1.554400
C	-0.097300	-1.262300	-0.853500
C	-0.047000	-1.284300	0.578900
C	-0.024100	1.207400	0.616000
C	-0.012500	-0.049100	1.315100
H	-0.108500	0.013400	-2.644400
H	-0.108100	-2.205900	-1.394600
H	-0.067400	-2.243200	1.093000
H	0.028200	2.148100	1.160400
H	0.008000	-0.064300	2.404000
H	-0.075000	2.187600	-1.329400
H	-0.875400	1.716600	3.998800
H	-0.953900	3.047500	2.836000
H	-1.779000	3.185400	4.406700
H	-5.305400	4.302800	5.087300
H	-5.787100	2.813800	4.244600
H	-4.258900	2.873300	5.144700
H	6.147400	2.868100	5.260100
H	4.919100	1.653400	5.651700
H	5.964800	1.423100	4.245200
H	1.570900	0.343800	5.483800
H	3.275300	0.039000	5.101100
H	2.799200	1.540000	5.920100
H	5.528800	-4.283000	4.880600
H	5.960800	-2.796400	4.006300
H	4.471800	-2.862900	4.968500
H	1.021900	-1.730500	3.909600
H	1.090300	-3.069200	2.750300
H	1.943600	-3.190200	4.306900
H	-5.182500	-4.078500	5.547400
H	-4.481700	-2.450500	5.538600
H	-5.832500	-2.836000	4.461800
H	-1.423300	-0.602500	4.879300
H	-3.197700	-0.595700	4.838500
H	-2.323300	-2.002600	5.483000
H	-1.903300	0.503600	-5.135400
H	-3.646200	0.618700	-4.811400
H	-2.805600	1.947100	-5.630700
H	-3.777200	5.126400	-5.343500
H	-2.919300	5.403700	-3.824500
H	-2.353900	4.148700	-4.948300
H	-5.625000	-4.285700	-5.025900
H	-6.038700	-2.789500	-4.159800
H	-4.565800	-2.869400	-5.145800
H	-1.065900	-1.801300	-4.172600
H	-1.150300	-3.142200	-3.018400
H	-2.041700	-3.230600	-4.554400
H	5.153000	-4.047600	-5.724900
H	4.481900	-2.407100	-5.697000
H	5.812900	-2.835400	-4.611200

H	1.399200	-0.587100	-5.072700
H	3.173600	-0.570100	-5.027800
H	2.306300	-1.992900	-5.651100
H	5.509100	5.485200	-3.871400
H	6.148300	4.176900	-2.859100
H	5.166700	3.792300	-4.281800
H	3.056600	3.283100	-4.848900
H	3.827100	1.736100	-4.428900
H	2.106400	1.789500	-4.868000
H	-8.941700	2.877300	-0.390800
H	-8.495200	1.316800	-1.108500
H	-8.578700	1.501800	0.664600
H	-7.466500	4.219400	0.860200
H	-6.794400	2.930800	1.885700
H	-5.715200	3.958300	0.901100
H	-7.371500	4.020900	-1.677900
H	-5.625400	3.762100	-1.534500
H	-6.627100	2.588800	-2.430400
H	-8.949800	-2.823800	0.444600
H	-8.532400	-1.236900	1.113900
H	-8.547500	-1.489700	-0.652800
H	-7.450800	-4.269800	-0.579800
H	-6.811000	-3.069700	-1.723000
H	-5.704200	-3.992700	-0.668800
H	-7.405800	-3.828000	1.920700
H	-5.655700	-3.584300	1.795600
H	-6.678100	-2.324900	2.538000
H	8.261100	2.960500	1.732500
H	6.860700	2.183200	2.503600
H	8.005200	1.205700	1.563300
H	6.899600	4.574000	0.617100
H	5.514700	4.167900	-0.408400
H	5.402900	3.962000	1.342800
H	8.237500	3.367600	-0.845300
H	8.245900	1.596200	-0.938000
H	7.012000	2.515200	-1.817200
H	8.920600	-2.716800	-0.577400
H	8.462600	-1.121000	-1.199400
H	8.477100	-1.429500	0.559100
H	7.402700	-3.724000	-2.086400
H	5.647100	-3.518800	-1.965600
H	6.648000	-2.219400	-2.667800
H	7.441300	-4.235300	0.394900
H	6.782100	-3.080000	1.572600
H	5.689900	-3.990600	0.492700

242

Ba-Bz_s

N	-4.526800	1.464800	-0.654800
N	4.700300	1.599100	-0.137700
C	-5.841800	1.460000	-0.642600
C	-6.738400	2.677600	-1.046600
C	-3.585100	2.318600	-1.221900
C	-3.013700	1.947400	-2.481400
C	-1.978200	2.727400	-3.003300
H	-1.554000	2.477900	-3.975700
C	-1.452400	3.810800	-2.305500
H	-0.640800	4.398400	-2.737500
C	-1.956400	4.118500	-1.048100
H	-1.525500	4.949200	-0.484300
C	-3.012100	3.395400	-0.486500
C	-3.540800	0.725100	-3.223500
H	-3.813900	-0.011400	-2.444200
C	-2.467300	0.088500	-4.118200
H	-2.055600	0.852900	-4.796100
H	-1.630600	-0.219000	-3.468000
C	-4.854700	1.026900	-3.977800
H	-4.619200	1.298600	-5.022100
C	-5.871600	-0.106900	-3.924100

C	-3.524700	3.807200	0.884100
H	-4.477900	3.281000	1.054700
C	-2.536500	3.354800	1.975600
H	-2.404300	2.259000	1.933700
H	-1.541300	3.759800	1.728000
C	-3.785700	5.333200	0.954700
H	-3.920300	5.719700	-0.067700
C	-4.992900	5.748700	1.786100
C	6.007400	1.514300	-0.333500
C	6.898900	2.784300	-0.540000
C	3.818800	2.663800	-0.350000
C	3.238400	3.374300	0.748600
C	2.188600	4.266500	0.498900
H	1.714900	4.774000	1.339600
C	1.725300	4.518500	-0.785100
H	0.923400	5.239400	-0.950800
C	2.286500	3.827400	-1.854300
H	1.918500	4.017400	-2.863800
C	3.296600	2.881300	-1.668200
C	3.685600	3.149300	2.186200
H	4.508900	2.416700	2.144200
C	2.555400	2.519900	3.019200
H	1.659600	3.161100	2.973700
H	2.221300	1.577200	2.552700
C	4.267300	4.398300	2.892400
C	3.305900	5.510200	3.298400
H	4.790000	4.040000	3.794800
C	3.787800	2.066300	-2.853300
H	4.740400	1.606400	-2.556500
C	2.850200	0.884500	-3.183000
H	2.646200	0.306300	-2.267800
C	1.516000	1.227100	-3.825200
C	4.060600	2.924000	-4.098700
H	4.665600	3.794100	-3.803200
C	4.769800	2.169500	-5.215000
Ba	-2.583800	-0.085300	0.430100
Ba	2.652200	-0.174000	0.386600
N	-5.012900	-1.326200	0.505600
N	5.063000	-1.432900	-0.033300
C	-6.597100	0.346900	-0.194600
H	-7.660200	0.521600	-0.255400
C	-6.262400	-0.944100	0.270400
C	-7.475900	-1.915600	0.457600
C	-4.550000	-2.535700	1.034700
C	-4.302400	-2.647100	2.436200
C	-3.826800	-3.852900	2.960000
H	-3.676600	-3.938100	4.039700
C	-3.546800	-4.940300	2.140400
H	-3.199900	-5.883900	2.567400
C	-3.675200	-4.794500	0.761600
H	-3.409200	-5.630100	0.111800
C	-4.132000	-3.605500	0.185400
C	-4.474600	-1.464500	3.372100
H	-4.878100	-0.633100	2.768600
C	-3.085700	-1.054500	3.903500
H	-2.688600	-1.882600	4.515300
H	-2.367600	-0.976600	3.067800
C	-5.468000	-1.722700	4.528700
H	-6.129600	-2.557700	4.257200
H	-4.910400	-2.067100	5.417500
C	-4.102200	-3.436700	-1.321000
H	-4.743200	-2.573900	-1.559800
C	-2.682400	-3.055700	-1.797200
H	-2.319600	-2.183200	-1.231100
C	-1.614500	-4.133600	-1.711600
C	-4.652200	-4.647500	-2.088700
C	-4.819400	-4.402500	-3.581900
H	-4.001300	-5.523500	-1.935500
C	6.707800	0.286100	-0.362600
H	7.763300	0.407900	-0.529100
C	6.316900	-1.066100	-0.245700
C	7.456400	-2.119400	-0.468900
C	4.564200	-2.667600	0.381500
C	3.823900	-3.509500	-0.498000
C	3.183400	-4.642000	0.022000
H	2.623600	-5.289000	-0.658100
C	3.239300	-4.963500	1.371400
H	2.730700	-5.852000	1.751800
C	3.947000	-4.130400	2.235800
H	3.981500	-4.374200	3.299100
C	4.602400	-2.987200	1.776800
C	3.670800	-3.229000	-1.985900
H	4.307200	-2.362800	-2.231000
C	2.206600	-2.850300	-2.269400
H	1.562200	-3.640700	-1.850600
H	1.941600	-1.945000	-1.697600
C	4.128400	-4.436600	-2.839100
H	4.872600	-5.015900	-2.270400
H	3.273700	-5.122300	-2.977000
C	5.318600	-2.075600	2.759600
H	6.119600	-1.563200	2.211100
C	4.403900	-0.949300	3.286300
H	4.333100	-0.168800	2.508900
C	3.026100	-1.386300	3.762900
C	5.987700	-2.847000	3.907900
H	6.453500	-3.755300	3.492900
C	7.043400	-2.040200	4.651900
H	-2.878900	5.836000	1.334500
H	-5.312600	1.925200	-3.542800
C	-2.911200	-1.081700	-4.984800
H	-5.623800	-4.926600	-1.654900
H	-2.748100	-2.699100	-2.833600
C	-6.324700	-0.516200	4.893900
C	-3.034500	0.240300	4.697300
C	4.725300	-4.082100	-4.195800
C	1.810700	-2.606400	-3.716900
H	5.227800	-3.202100	4.625000
C	-2.893400	3.730500	3.405500
H	4.927100	-0.435600	4.108300
C	2.906100	2.237800	4.472500
H	5.051300	4.833500	2.260600
H	3.117900	3.343000	-4.486300
H	3.401100	0.181300	-3.828300
C	-8.838800	-1.269000	0.165200
C	-7.326500	-3.092800	-0.514800
C	-7.546800	-2.453800	1.891500
C	-7.450600	3.148500	0.237200
C	-5.979200	3.873400	-1.626900
C	-7.806400	2.281900	-2.081100
C	8.407100	2.496500	-0.481100
C	6.614300	3.433800	-1.899200
C	6.597700	3.803200	0.559600
C	8.813700	-1.491300	-0.819200
C	7.696400	-3.016400	0.754900
C	7.051300	-3.000800	-1.654800
C	0.059700	1.271000	0.796700
C	0.007200	0.278300	1.900600
C	0.010200	-1.112200	1.537200
C	-0.005600	-1.537000	0.221300
C	0.043400	0.860200	-0.517600
C	-0.003100	-0.535800	-0.885600
H	0.038500	0.590500	2.943300
H	0.011900	-1.869300	2.328000
H	0.005500	-2.604300	-0.000700
H	0.058200	1.623400	-1.298600
H	-0.046700	-0.840900	-1.928500
H	0.116300	2.336500	1.015300
H	0.905000	0.322200	-3.949700
H	0.935100	1.926700	-3.209000
H	1.642200	1.678800	-4.820800
H	5.029800	2.842100	-6.046500
H	5.704000	1.710000	-4.852900
H	4.147600	1.362000	-5.631000
H	-6.789000	0.149900	-4.476400
H	-5.479400	-1.038300	-4.354100
H	-6.156300	-0.313500	-2.879600
H	-2.044100	-1.532100	-5.492600
H	-3.401400	-1.874600	-4.404000
H	-3.618900	-0.767600	-5.766700
H	-5.307800	-5.258100	-4.072600
H	-5.440100	-3.511800	-3.770000
H	-3.854400	-4.245500	-4.087700
H	-0.656900	-3.745900	-2.087300
H	-1.454300	-4.469100	-0.676700
H	-1.869900	-5.015400	-2.319200
H	5.028400	-4.990100	-4.740200
H	4.021600	-3.533200	-4.835700
H	5.620300	-3.453300	-4.079500
H	0.771400	-2.246200	-3.769000
H	2.447300	-1.846400	-4.193400
H	1.865700	-3.522700	-4.323000
H	2.095000	1.682300	4.967500
H	3.823800	1.635000	4.553800
H	3.066500	3.160800	5.048500
H	3.824500	6.239000	3.940500
H	2.923900	6.065500	2.430200
H	2.439400	5.137400	3.866700
H	7.518100	-2.640800	5.442700
H	7.838300	-1.700600	3.968500
H	6.621700	-1.144500	5.133800
H	2.440000	-0.534200	4.132500
H	2.441700	-1.877500	2.968100
H	3.102300	-2.119000	4.581100
H	-7.033400	-0.759100	5.700900
H	-5.720200	0.338700	5.228700
H	-6.911500	-0.174700	4.026400
H	-1.992900	0.518300	4.917700
H	-3.484200	1.071000	4.132900
H	-3.567800	0.161000	5.655900
H	-5.073900	6.845600	1.840000
H	-5.924600	5.372500	1.339000
H	-4.948100	5.368800	2.816200
H	-2.884300	4.819800	3.557500
H	-3.892500	3.361800	3.686300
H	-2.167700	3.295300	4.108500
H	8.949800	3.452000	-0.549600
H	8.700200	2.012900	0.462200
H	8.752400	1.869600	-1.315600
H	7.302300	4.279800	-2.056900
H	6.762200	2.721200	-2.723700
H	5.592400	3.826300	-1.949300
H	7.278000	4.665100	0.473800
H	5.574300	4.182300	0.475600
H	6.730700	3.363300	1.559000
H	9.541700	-2.298900	-0.991700
H	8.768500	-0.886400	-1.736200
H	9.207900	-0.863800	-0.006600
H	8.505600	-3.727800	0.525600
H	8.013400	-2.427800	1.627800
H	6.811900	-3.603300	1.027300
H	7.870100	-3.689900	-1.915000
H	6.171500	-3.606800	-1.418400
H	6.828300	-2.385700	-2.538800
H	-8.457900	3.147500	-2.280500
H	-7.350900	1.982200	-3.033500
H	-8.446400	1.457100	-1.742500
H	-6.707600	4.646100	-1.918600
H	-5.294300	4.326400	-0.906000
H	-5.394200	3.615800	-2.518900
H	-8.007900	4.079200	0.042100

H	-8.160900	2.398100	0.609900
H	-6.727900	3.346700	1.041700
H	-9.626000	-2.021600	0.325900
H	-9.053500	-0.422800	0.834400
H	-8.922900	-0.923200	-0.875000
H	-8.435600	-3.095000	2.002400
H	-6.671000	-3.056900	2.153300
H	-7.636300	-1.631100	2.614300
H	-8.235900	-3.714200	-0.494200
H	-7.177900	-2.742100	-1.547300
H	-6.483800	-3.733000	-0.235600

242

Ba-Bz_oss

N	-4.526800	1.464900	-0.654500
N	4.700300	1.599100	-0.137600
C	-5.841900	1.460100	-0.642400
C	-6.738300	2.677900	-1.046200
C	-3.585100	2.318700	-1.221700
C	-3.013700	1.947400	-2.481200
C	-1.978200	2.727200	-3.003200
H	-1.554100	2.477700	-3.975600
C	-1.452200	3.810600	-2.305400
H	-0.640500	4.398000	-2.737400
C	-1.956100	4.118300	-1.047900
H	-1.524900	4.948900	-0.484100
C	-3.011900	3.395400	-0.486300
C	-3.541100	0.725200	-3.223300
H	-3.814200	-0.011400	-2.444100
C	-2.467700	0.088500	-4.118200
H	-2.055900	0.852800	-4.796000
H	-1.631000	-0.219300	-3.468000
C	-4.854900	1.027200	-3.977600
H	-4.619400	1.299100	-5.021800
C	-5.871900	-0.106500	-3.924200
C	-3.524500	3.807300	0.884300
H	-4.477700	3.281200	1.054900
C	-2.536300	3.355000	1.975700
H	-2.404300	2.259100	1.934000
H	-1.541000	3.759700	1.728100
C	-3.785400	5.333300	0.954800
H	-3.920000	5.719700	-0.067700
C	-4.992500	5.748900	1.786200
C	6.007400	1.514300	-0.333400
C	6.898900	2.784400	-0.540000
C	3.818800	2.663700	-0.350100
C	3.238400	3.374400	0.748500
C	2.188700	4.266700	0.498800
H	1.715100	4.774300	1.339400
C	1.725400	4.518600	-0.785200
H	0.923600	5.239600	-0.950900
C	2.286500	3.827400	-1.854300
H	1.918600	4.017400	-2.863900
C	3.296600	2.881300	-1.668200
C	3.685600	3.149400	2.186100
H	4.509000	2.416900	2.144100
C	2.555300	2.520000	3.019000
H	1.659500	3.161000	2.973300
H	2.221500	1.577200	2.552500
C	4.267300	4.398400	2.892500
C	3.305900	5.510300	3.298200
H	4.789700	4.040000	3.795000
C	3.787700	2.066200	-2.853300
H	4.740300	1.606200	-2.556500
C	2.850100	0.884400	-3.183200
H	2.646100	0.306000	-2.268200
C	1.515800	1.227200	-3.825200
C	4.060600	2.924000	-4.098700
H	4.665600	3.794000	-3.803200
C	4.769900	2.169500	-5.215000
Ba	-2.583800	-0.085400	0.430000
Ba	2.652200	-0.174200	0.386500
N	-5.013100	-1.326200	0.505300
N	5.063200	-1.433000	-0.033200
C	-6.597200	0.347000	-0.194700
H	-7.660200	0.521800	-0.255400
C	-6.262600	-0.944000	0.270300
C	-7.476100	-1.915500	0.457300
C	-4.550300	-2.535700	1.034500
C	-4.302700	-2.647100	2.436000
C	-3.827200	-3.852900	2.959900
H	-3.677000	-3.938100	4.039600
C	-3.547100	-4.940300	2.140300
H	-3.200300	-5.883900	2.567400
C	-3.675500	-4.794600	0.761500
H	-3.409500	-5.630200	0.111700
C	-4.132200	-3.605500	0.185200
C	-4.475000	-1.464500	3.371900
H	-4.878400	-0.633100	2.768500
C	-3.086100	-1.054600	3.903600
H	-2.689000	-1.882800	4.515100
H	-2.367900	-0.976500	3.067900
C	-5.468500	-1.722900	4.528400
H	-6.130100	-2.557700	4.256800
H	-4.911000	-2.067400	5.417300
C	-4.102300	-3.436800	-1.321200
H	-4.743200	-2.574000	-1.560000
C	-2.682400	-3.056100	-1.797400
H	-2.319700	-2.183000	-1.232100
C	-1.614600	-4.133800	-1.711000
C	-4.652400	-4.647600	-2.088700
C	-4.819800	-4.402800	-3.581900
H	-4.001500	-5.523700	-1.935500
C	6.707900	0.286200	-0.362400
H	7.763400	0.408000	-0.528900
C	6.317100	-1.066000	-0.245600
C	7.456600	-2.119200	-0.468900
C	4.564500	-2.667600	0.381600
C	3.824100	-3.509500	-0.497800
C	3.183600	-4.642000	0.022300
H	2.623600	-5.289000	-0.657900
C	3.239500	-4.963500	1.371700
H	2.730900	-5.851900	1.752200
C	3.947300	-4.130400	2.236000
H	3.981900	-4.374200	3.299300
C	4.602700	-2.987300	1.776900
C	3.671000	-3.229100	-1.985700
H	4.307300	-2.362900	-2.230900
C	2.206800	-2.850700	-2.269400
H	1.562400	-3.641200	-1.850900
H	1.941600	-1.945500	-1.697600
C	4.128900	-4.436800	-2.838800
H	4.873200	-5.015800	-2.270100
H	3.274300	-5.122700	-2.976600
C	5.318900	-2.075600	2.759600
H	6.119900	-1.563300	2.211100
C	4.404200	-0.949300	3.286200
H	4.333400	-0.168900	2.508700
C	3.026500	-1.386200	3.762900
C	5.988000	-2.846900	3.908000
H	6.453800	-3.755200	3.493100
C	7.043700	-2.040100	4.652000
H	-2.878600	5.836100	1.334400
H	-5.312800	1.925400	-3.542500
C	-2.911900	-1.081600	-4.984800
H	-5.624000	-4.926700	-1.654700
H	-2.748100	-2.700300	-2.834200
C	-6.325200	-0.516300	4.893700
C	-3.035100	0.240000	4.697700
C	4.725700	-4.082400	-4.195600
C	1.811200	-2.606700	-3.717100
H	5.228100	-3.202000	4.625100
C	-2.893000	3.730900	3.405600
H	4.927400	-0.435500	4.108100
C	2.905800	2.237900	4.472400
H	5.051500	4.833500	2.260900
H	3.117900	3.342900	-4.486300
H	3.400900	0.181500	-3.828700
C	-8.839000	-1.268700	0.165100
C	-7.326700	-3.092500	-0.515200
C	-7.547100	-2.453800	1.891200
C	-7.450400	3.148700	0.237700
C	-5.979100	3.873800	-1.626500
C	-7.806500	2.282400	-2.080700
C	8.407100	2.496700	-0.480900
C	6.614300	3.433800	-1.899100
C	6.597600	3.803400	0.559600
C	8.813800	-1.491100	-0.819500
C	7.696900	-3.016200	0.754900
C	7.051300	-3.000700	-1.654800
C	0.059500	1.270800	0.796600
C	0.007300	0.278100	1.900500
C	0.010300	-1.112400	1.537100
C	-0.005500	-1.537200	0.221200
C	0.043400	0.860000	-0.517700
C	-0.003000	-0.536000	-0.885700
H	0.038500	0.590300	2.943200
H	0.011900	-1.869500	2.328000
H	0.005600	-2.604400	-0.000800
H	0.058200	1.623200	-1.298700
H	-0.046600	-0.841200	-1.928500
H	0.116100	2.336300	1.015100
H	0.904900	0.322300	-3.949800
H	0.935000	1.926600	-3.208800
H	1.641900	1.679000	-4.820800
H	5.029900	2.842300	-6.046500
H	5.704000	1.710000	-4.852900
H	4.147700	1.362100	-5.631100
H	-6.789100	0.150300	-4.476800
H	-5.479600	-1.038000	-4.354100
H	-6.156800	-0.313200	-2.879800
H	-2.044900	-1.532200	-5.492600
H	-3.402300	-1.874400	-4.403900
H	-3.619500	-0.767300	-5.766600
H	-5.308600	-5.258400	-4.072400
H	-5.440500	-3.512100	-3.770000
H	-3.854900	-4.246100	-4.087900
H	-0.657000	-3.746500	-2.087000
H	-1.454300	-4.468500	-0.675800
H	-1.869900	-5.016100	-2.317800
H	5.028900	-4.990500	-4.739900
H	4.021800	-3.533700	-4.835600
H	5.620500	-3.453500	-4.079500
H	0.772100	-2.246300	-3.769300
H	2.448100	-1.846800	-4.193300
H	1.866200	-3.523000	-4.323100
H	2.094700	1.682300	4.967200
H	3.823600	1.635300	4.553800
H	3.066000	3.161000	5.048400
H	3.824400	6.239200	3.940400
H	2.924100	6.065700	2.430000
H	2.439200	5.137600	3.866400
H	7.518200	-2.640600	5.442900
H	7.838600	-1.700600	3.968700
H	6.621900	-1.144400	5.133800
H	2.440200	-0.533900	4.132000
H	2.442200	-1.877800	2.968300
H	3.102600	-2.118500	4.581400
H	-7.034100	-0.759400	5.700600

H	-5.720800	0.338400	5.228800
H	-6.911900	-0.174700	4.026100
H	-1.993500	0.518200	4.918100
H	-3.485200	1.070700	4.133700
H	-3.568300	0.160200	5.656400
H	-5.073500	6.845800	1.839900
H	-5.924300	5.372700	1.339100
H	-4.947700	5.369100	2.816200
H	-2.883900	4.820200	3.557500
H	-3.892200	3.362300	3.686600
H	-2.167300	3.295900	4.108600
H	8.949700	3.452200	-0.549500
H	8.700200	2.013200	0.462500
H	8.752500	1.869600	-1.315300
H	7.302300	4.279800	-2.056900
H	6.762300	2.721100	-2.723600
H	5.592500	3.826300	-1.949300
H	7.277900	4.665200	0.473800
H	5.574200	4.182400	0.475600
H	6.730600	3.363500	1.559000
H	9.541800	-2.298600	-0.992200
H	8.768400	-0.886100	-1.736400
H	9.208100	-0.863600	-0.006900
H	8.506200	-3.727400	0.525500
H	8.013900	-2.427500	1.627700
H	6.812500	-3.603200	1.027400
H	7.870200	-3.689700	-1.915100
H	6.171600	-3.606800	-1.418100
H	6.828100	-2.385600	-2.538700
H	-8.457900	3.148100	-2.280000
H	-7.351100	1.982800	-3.033300
H	-8.446500	1.457700	-1.742200
H	-6.707400	4.646500	-1.918000
H	-5.294100	4.326500	-0.905500
H	-5.394200	3.616200	-2.518600
H	-8.007700	4.079400	0.042600
H	-8.160700	2.398200	0.610300
H	-6.727500	3.346900	1.042100
H	-9.626200	-2.021400	0.325800
H	-9.053600	-0.422600	0.834300
H	-8.923100	-0.922900	-0.875100
H	-8.435900	-3.095100	2.002000
H	-6.671200	-3.057000	2.152900
H	-7.636500	-1.631300	2.614100
H	-8.236100	-3.713900	-0.494700
H	-7.178100	-2.741700	-1.547700
H	-6.484000	-3.732800	-0.236000

242

Ba-Bz_t

N	4.583300	1.022900	1.192500
N	-4.590100	1.512300	-0.686000
C	5.870600	1.191600	0.976200
C	6.772500	2.228700	1.724000
C	3.686600	1.569700	2.103000
C	3.248300	0.740500	3.186300
C	2.227100	1.196300	4.025000
H	1.905400	0.580700	4.865200
C	1.589100	2.411100	3.800800
H	0.786700	2.744200	4.461600
C	1.971400	3.184000	2.710900
H	1.449900	4.124300	2.515600
C	3.006600	2.795500	1.856300
C	3.891600	-0.622300	3.401700
H	4.130100	-1.017500	2.396200
C	2.935500	-1.610800	4.083900
H	2.648600	-1.209300	5.069400
H	2.000900	-1.650800	3.495400
C	5.268900	-0.531200	4.095300
H	5.139800	-0.700200	5.178900
C	6.312400	-1.479200	3.515400
C	3.353300	3.710900	0.695000
H	4.311700	3.374900	0.269600
C	2.277200	3.565800	-0.399500
H	2.343500	2.548400	-0.823000
H	1.287900	3.607100	0.083000
C	3.516000	5.174600	1.172900
H	3.779900	5.173100	2.242400
C	4.563800	5.986700	0.422500
C	-5.912000	1.563200	-0.662400
C	-6.703600	2.824600	-1.146500
C	-3.635300	2.526900	-0.775700
C	-2.824800	2.678900	-1.948100
C	-1.700600	3.512300	-1.887700
H	-1.050800	3.586900	-2.760200
C	-1.386200	4.245100	-0.750200
H	-0.521500	4.910200	-0.743500
C	-2.186000	4.115800	0.381500
H	-1.934600	4.686000	1.277400
C	-3.281600	3.249700	0.411500
C	-3.146100	1.958100	-3.251000
H	-4.066300	1.380700	-3.064400
C	-2.051000	0.950000	-3.635100
H	-1.079100	1.464700	-3.696600
H	-1.914100	0.225200	-2.816600
C	-3.467500	2.905100	-4.434000
C	-2.312600	3.657400	-5.086300
H	-3.969800	2.297000	-5.204700
C	-4.047200	3.052800	1.711000
H	-5.018200	2.608600	1.455900
C	-3.373100	2.029800	2.647600
H	-3.308700	1.060400	2.129200
C	-2.005500	2.408700	3.189000
C	-4.329200	4.378100	2.436700
H	-4.731500	5.097100	1.706400
C	-5.297400	4.248000	3.604800
Ba	2.556700	-0.513000	0.165600
Ba	-2.638200	-0.360900	-0.080000
N	4.933800	-0.821100	-1.185800
N	-5.189800	-1.231300	0.471200
C	6.580000	0.455100	-0.003600
H	7.638500	0.686300	-0.024800
C	6.191500	-0.471300	-0.999600
C	7.401900	-0.968300	-1.866200
C	4.344500	-1.741700	-2.042600
C	3.479500	-1.258600	-3.073700
C	2.783900	-2.182600	-3.859600
H	2.140600	-1.827000	-4.664500
C	2.879600	-3.551600	-3.632900
H	2.325200	-4.253700	-4.259400
C	3.666800	-4.015400	-2.583100
H	3.711400	-5.087800	-2.386200
C	4.393300	-3.140300	-1.773200
C	3.311000	0.240200	-3.279800
H	3.092500	0.677700	-2.285400
C	2.113900	0.589700	-4.169700
H	2.313300	0.236200	-5.196000
H	1.232600	0.032700	-3.815500
C	4.609400	0.938200	-3.749800
H	5.438100	0.220700	-3.710700
H	4.504500	1.206500	-4.815600
C	5.189600	-3.664400	-0.594200
H	6.049000	-2.993900	-0.455600
C	4.381500	-3.562800	0.713300
H	4.325900	-2.496500	0.990100
C	2.995800	-4.192400	0.685000
C	5.758800	-5.071700	-0.821200
C	6.826400	-5.474900	0.187300
H	4.948300	-5.819700	-0.810100
C	-6.717100	0.489300	-0.215600
H	-7.771200	0.696900	-0.274100
C	-6.426300	-0.787800	0.312000
C	-7.662400	-1.618000	0.799000
C	-4.721800	-2.529700	0.654500
C	-4.123700	-2.943200	1.881700
C	-3.472600	-4.183600	1.936400
H	-3.023800	-4.500700	2.881300
C	-3.383600	-5.018400	0.831100
H	-2.868900	-5.979100	0.901100
C	-3.956000	-4.609700	-0.372500
H	-3.879700	-5.260200	-1.245600
C	-4.613200	-3.385000	-0.491800
C	-4.162000	-2.105300	3.153300
H	-4.763600	-1.205800	2.941900
C	-2.735500	-1.656900	3.521900
H	-2.088600	-2.549500	3.553900
H	-2.318500	-1.049900	2.701400
C	-4.834100	-2.889200	4.308200
H	-5.527700	-3.628900	3.878200
H	-4.064900	-3.487200	4.828600
C	-5.186600	-2.957300	-1.833000
H	-5.998000	-2.245800	-1.632100
C	-4.170400	-2.175100	-2.691400
H	-4.060700	-1.162700	-2.265100
C	-2.818100	-2.843900	-2.890800
C	-5.805900	-4.129600	-2.610400
H	-6.370900	-4.755800	-1.901500
C	-6.728600	-3.695700	-3.741600
H	2.538500	5.684900	1.120900
H	5.642700	0.497400	4.002900
C	3.466300	-3.021200	4.296600
H	6.185700	-5.112600	-1.837100
H	4.976100	-4.005500	1.527800
C	4.976000	2.163900	-2.925700
C	1.768000	2.069000	-4.207400
C	-5.597700	-2.042200	5.319600
C	-2.562800	-0.879200	4.817200
H	-5.014500	-4.781700	-3.017100
C	2.307100	4.582900	-1.529500
H	-4.631000	-1.981600	-3.673200
C	-2.303600	0.183400	-4.924500
H	-4.217500	3.638400	-4.112100
H	-3.388400	4.825000	2.796700
H	-4.062800	1.828300	3.483400
C	8.079900	0.240500	-2.541000
C	8.430800	-1.653100	-0.947100
C	7.054100	-1.957600	-2.984600
C	7.313100	3.220800	0.676200
C	6.061600	3.040300	2.810000
C	7.963100	1.525200	2.399100
C	-8.201700	2.560300	-1.363800
C	-6.583400	3.969600	-0.133300
C	-6.137700	3.304400	-2.482700
C	-9.004300	-0.897600	0.602800
C	-7.794700	-2.980400	0.102800
C	-7.486100	-1.850300	2.303000
C	0.069700	0.500800	-0.915800
C	0.026400	-0.877800	-1.325000
C	-0.062600	-1.916600	-0.339600
C	-0.107900	-1.582700	1.054200
C	-0.017200	0.834600	0.477800
C	-0.103800	-0.204800	1.462300
H	0.108100	-1.144200	-2.379600
H	-0.083200	-2.962700	-0.651100
H	-0.194900	-2.376500	1.798100
H	0.004800	1.879300	0.785700
H	-0.097900	0.058500	2.520400
H	0.101500	1.298600	-1.656300
H	-1.610900	1.617200	3.839500

H	-1.276400	2.557200	2.382000
H	-2.046500	3.333700	3.784600
H	-5.549800	5.235100	4.021500
H	-6.238700	3.768100	3.291200
H	-4.879500	3.645900	4.426200
H	7.277800	-1.378100	4.035000
H	6.007300	-2.531900	3.591700
H	6.482400	-1.257700	2.449100
H	2.676700	-3.669900	4.705800
H	3.816400	-3.476300	3.360300
H	4.304700	-3.040800	5.008600
H	7.236800	-6.468800	-0.047100
H	7.665200	-4.761000	0.187200
H	6.433400	-5.520400	1.214500
H	2.486500	-4.082800	1.653900
H	2.348300	-3.740500	-0.083200
H	3.048400	-5.269200	0.462000
H	-6.051900	-2.677100	6.096400
H	-4.957500	-1.308400	5.826700
H	-6.410900	-1.484800	4.832600
H	-1.521300	-0.535800	4.914700
H	-3.211500	0.008800	4.851700
H	-2.784800	-1.493500	5.702100
H	-1.530400	-0.585000	-5.077100
H	-3.281300	-0.323200	-4.904900
H	-2.288700	0.837100	-5.808900
H	-2.657100	4.146900	-6.010400
H	-1.912400	4.448300	-4.436800
H	-1.476600	2.996500	-5.362700
H	-7.187800	-4.565900	-4.235000
H	-7.544000	-3.056100	-3.366800
H	-6.196700	-3.125600	-4.518800
H	-2.174900	-2.249400	-3.553300
H	-2.275400	-2.985500	-1.943100
H	-2.929700	-3.839400	-3.347600
H	5.888600	2.650100	-3.303300
H	4.171700	2.911900	-2.940700
H	5.157200	1.882900	-1.876500
H	0.895200	2.251600	-4.852200
H	1.521600	2.437600	-3.201400
H	2.593700	2.684000	-4.594900
H	4.580300	7.029800	0.774800
H	5.569200	5.570100	0.582300
H	4.386900	6.005300	-0.661600
H	2.145900	5.607200	-1.162100
H	3.261200	4.570800	-2.076900
H	1.508300	4.373200	-2.255500
H	-8.658400	3.461500	-1.801400
H	-8.377100	1.724900	-2.057400
H	-8.737200	2.352100	-0.426500
H	-7.207900	4.817300	-0.458100
H	-6.926400	3.662300	0.865500
H	-5.551200	4.330800	-0.057900
H	-6.734200	4.147600	-2.864900
H	-5.107400	3.655300	-2.369000
H	-6.156800	2.505000	-3.238000
H	-9.810600	-1.541400	0.986900
H	-9.052200	0.053100	1.152900
H	-9.220000	-0.698100	-0.457100
H	-8.663700	-3.513600	0.519800
H	-7.967400	-2.865000	-0.976700
H	-6.916100	-3.618000	0.252500
H	-8.379000	-2.339800	2.723100
H	-6.627500	-2.498200	2.503300
H	-7.337400	-0.896100	2.830000
H	8.630300	2.279800	2.845200
H	7.628100	0.858600	3.204300
H	8.557800	0.926700	1.696600
H	6.803500	3.678000	3.315500
H	5.288100	3.697800	2.404600
H	5.590400	2.411000	3.575300
H	7.846800	4.045600	1.175500
H	8.010400	2.742600	-0.024700
H	6.496500	3.653700	0.081200
H	8.952300	-0.102200	-3.119800
H	7.395500	0.746600	-3.234800
H	8.430100	0.986900	-1.816500
H	7.962000	-2.143700	-3.579200
H	6.707800	-2.924900	-2.605800
H	6.284400	-1.578300	-3.668400
H	9.267400	-2.044800	-1.547700
H	8.845500	-0.964100	-0.199400
H	7.983900	-2.498700	-0.404200

158

Mg-Bz_s

Mg	-2.186800	0.149400	0.215200
Mg	2.124100	0.088900	-0.117800
N	-3.647700	1.395400	-0.511900
N	-3.586300	-1.250700	0.771600
N	3.506400	-1.264600	-0.797200
N	3.585900	1.399600	0.482000
C	-0.296500	0.813800	1.247600
H	-0.144800	1.356600	2.183600
C	0.236900	-0.583800	1.231200
H	0.550900	-1.085500	2.148600
C	0.099300	-1.272100	0.047400
H	0.322300	-2.338000	-0.028600
C	-0.444900	-0.525900	-1.096300
H	-0.487400	-1.067900	-2.041200
C	0.088600	0.859300	-1.151400
H	0.281900	1.359300	-2.105100
C	0.158100	1.540400	0.041200
H	0.446800	2.592000	0.090400
C	-5.895700	2.344600	-0.737400
H	-6.020900	3.074000	0.079100
H	-6.887800	1.940400	-0.977500
H	-5.504400	2.892700	-1.605200
C	-4.947600	1.255200	-0.297800
C	-5.519900	0.135500	0.343900
H	-6.604400	0.164200	0.453300
C	-4.897300	-1.044500	0.801900
C	-5.800300	-2.112600	1.371700
H	-5.409800	-3.118600	1.165600
H	-6.820100	-2.029000	0.974800
H	-5.855400	-2.009900	2.467600
C	-3.082200	2.447100	-1.271400
C	-2.670600	2.156900	-2.597400
C	-1.963400	3.129900	-3.307300
H	-1.640100	2.923100	-4.329100
C	-1.644800	4.356400	-2.725900
H	-1.080000	5.100900	-3.292100
C	-2.041000	4.623600	-1.420400
H	-1.779800	5.582100	-0.964200
C	-2.763100	3.685000	-0.673600
C	-3.157500	4.003300	0.756700
H	-3.754100	3.156300	1.126800
C	-1.930200	4.119900	1.663700
H	-1.361900	3.181200	1.678700
H	-2.229500	4.352400	2.698200
H	-1.250700	4.916900	1.323400
C	-4.026300	5.261400	0.833500
H	-3.459400	6.159800	0.541300
H	-4.390400	5.421500	1.860800
H	-4.898800	5.191200	0.166800
C	-2.985900	0.802100	-3.206400
H	-2.893400	0.068000	-2.388400
C	-2.012500	0.378100	-4.301700
H	-2.130900	0.980000	-5.217100
H	-2.192400	-0.671600	-4.579400
H	-0.967100	0.462900	-3.973300
C	-4.430000	0.729300	-3.710400
H	-5.151000	0.902700	-2.901300
H	-4.642100	-0.264800	-4.135200
H	-4.608800	1.482000	-4.495100
C	-2.989900	-2.476500	1.152000
C	-2.706500	-2.745700	2.508700
C	-1.973700	-3.895100	2.825600
H	-1.740400	-4.110800	3.871100
C	-1.528000	-4.761700	1.832500
H	-0.953800	-5.652200	2.098800
C	-1.806400	-4.483900	0.495900
H	-1.437600	-5.158400	-0.278300
C	-2.531400	-3.346300	0.130600
C	-2.820600	-3.028700	-1.325300
H	-2.760600	-1.931900	-1.418400
C	-4.243500	-3.432400	-1.721600
H	-4.396700	-4.515300	-1.586400
H	-4.432900	-3.190600	-2.779600
H	-4.996400	-2.903300	-1.122900
C	-1.799800	-3.619300	-2.293400
H	-0.766900	-3.392700	-1.994400
H	-1.957500	-3.209100	-3.302800
H	-1.890900	-4.714700	-2.372700
C	-3.133500	-1.787200	3.605800
H	-3.859300	-1.088400	3.166200
C	-3.822300	-2.501800	4.769100
H	-4.656400	-3.128600	4.418600
H	-4.221300	-1.770700	5.489700
H	-3.125600	-3.153900	5.319300
C	-1.949400	-0.943300	4.085300
H	-1.141000	-1.579600	4.479900
H	-2.258400	-0.250900	4.884600
H	-1.538100	-0.345900	3.259700
C	5.715200	-2.098600	-1.454600
H	5.412700	-3.104200	-1.131400
H	6.764900	-1.930600	-1.184100
H	5.639100	-2.086000	-2.553400
C	4.817200	-1.038100	-0.865400
C	5.440200	0.144300	-0.426200
H	6.521000	0.180100	-0.563000
C	4.881600	1.260000	0.231500
C	5.850500	2.335700	0.658300
H	6.009100	3.044100	-0.170100
H	6.827000	1.904900	0.915200
H	5.468700	2.910100	1.512500
C	2.971000	-2.539300	-1.116700
C	2.651900	-2.873700	-2.449300
C	2.011100	-4.093100	-2.697100
H	1.747300	-4.361700	-3.722700
C	1.694900	-4.964400	-1.659700
H	1.189800	-5.909400	-1.872900
C	2.010700	-4.622500	-0.346500
H	1.741600	-5.302100	0.463500
C	2.643800	-3.412200	-0.050000
C	3.067400	2.499200	1.212000
C	2.628400	2.260100	2.538100
C	1.985200	3.291100	3.227000
H	1.636900	3.123600	4.247300
C	1.763700	4.528100	2.625200
H	1.248000	5.319100	3.174400
C	2.190400	4.747600	1.320000
H	2.001200	5.715200	0.848500
C	2.844500	3.747300	0.591100
C	3.251900	4.014200	-0.847400
H	3.804100	3.132400	-1.203800
C	4.178400	5.228400	-0.955900
H	3.650700	6.159900	-0.696900
H	4.555100	5.339700	-1.984900

H	5.042500	5.141600	-0.280800
C	2.031100	4.178900	-1.757600
H	1.401400	3.279700	-1.758400
H	2.342900	4.373400	-2.796000
H	1.398200	5.019700	-1.434000
C	2.958400	-3.019900	1.383000
H	2.871200	-1.921100	1.430400
C	1.977100	-3.595300	2.400500
H	0.931200	-3.419000	2.111700
H	2.141900	-3.133300	3.386200
H	2.108600	-4.681400	2.529500
C	4.403300	-3.355500	1.763000
H	4.592400	-4.436500	1.665000
H	4.604500	-3.067400	2.807000
H	5.124300	-2.823100	1.128900
C	2.955900	-1.923200	-3.593400
H	3.635600	-1.151200	-3.204000
C	1.688600	-1.202500	-4.058400
H	0.934300	-1.917700	-4.422900
H	1.911600	-0.497200	-4.874500
H	1.233100	-0.640100	-3.232700
C	3.659800	-2.619700	-4.758900
H	4.555000	-3.164800	-4.422900
H	3.970400	-1.884300	-5.517500
H	2.999300	-3.344800	-5.259700
C	2.862700	0.903300	3.178900
H	2.699000	0.146400	2.390700
C	4.314800	0.740400	3.638100
H	5.019400	0.842100	2.802800
H	4.470200	-0.254400	4.084600
H	4.568900	1.498800	4.395400
C	1.899500	0.575500	4.315100
H	2.085800	1.199400	5.203600
H	2.026800	-0.472800	4.625000
H	0.851200	0.713400	4.015500

181

Ba-Ho_open

Ba	1.578765	-0.145687	-0.199004
N	-5.488016	0.234507	0.317655
N	4.191208	-0.851766	0.239432
N	3.148731	2.106447	-0.291252
N	-1.107776	-0.777236	-0.245044
C	3.610041	-2.586641	1.876355
C	-7.539246	0.141212	1.646989
C	2.209068	3.099488	0.038353
C	3.994804	-2.207822	0.556225
C	-4.924292	-0.683332	-0.414209
C	4.472647	2.253563	-0.368824
C	5.216563	3.625588	-0.635306
C	2.152088	3.624592	1.367265
C	-8.832345	0.617983	1.881042
H	-9.397161	0.219314	2.728546
C	3.351893	-3.935165	2.151111
H	3.090609	-4.230198	3.171062
C	-9.415460	1.592636	1.076464
H	-10.432015	1.943127	1.271887
C	7.628579	-0.674077	-0.872438
H	7.175523	-1.229285	-1.707599
H	8.622306	-1.110878	-0.682122
H	7.784459	0.360676	-1.206904
C	-7.365477	1.698390	-0.243017
C	-0.987278	-1.956970	-1.001060
C	3.736135	-4.529627	-0.139845
H	3.770991	-5.291762	-0.922921
C	0.231484	4.417988	-0.507178
H	-0.530182	4.733073	-1.220837
C	-5.771271	-1.673052	-1.306004
C	-2.326393	0.844036	1.111252
C	1.147714	2.924786	-2.321648
H	1.421552	1.851452	-2.275555
C	6.764931	-0.773673	0.402935
C	5.389461	1.181687	-0.265203
H	6.411771	1.512948	-0.401310
C	-0.865559	-4.391722	-1.111967
H	-0.932382	-5.365460	-0.618870
C	6.297934	3.884777	0.435695
H	5.880217	3.858403	1.451996
H	6.735059	4.884426	0.281181
H	7.124876	3.163057	0.395837
C	5.916665	3.523060	-2.010300
H	6.644553	2.701286	-2.051835
H	6.456298	4.460352	-2.222482
H	5.195420	3.364907	-2.825568
C	4.338938	4.886643	-0.674705
H	3.514264	4.819994	-1.390813
H	4.970809	5.735447	-0.981954
H	3.908614	5.136725	0.301988
C	4.024107	-3.201733	-0.469561
C	-2.331927	-0.304364	0.084047
C	-0.695477	-1.907201	-2.397114
C	5.329718	-0.186111	0.116046
C	-2.989294	2.091455	0.512444
H	-2.440241	2.440664	-0.376034
H	-2.996092	2.912926	1.247572
H	-4.025422	1.863021	0.234218
C	0.209885	4.965402	0.770138
H	-0.554463	5.696107	1.044268
C	7.417562	0.057718	1.529672
H	8.410790	-0.355470	1.769422
H	6.811029	0.023959	2.447998
H	7.550759	1.113401	1.261152
C	3.116973	3.181482	2.460933
H	3.946071	2.639558	1.983704
C	-6.796503	0.636919	0.529439
C	3.420063	-4.909049	1.161267
H	3.213788	-5.955261	1.398571
C	1.195704	3.478663	-0.896764
C	1.157952	4.549588	1.697654
H	1.124055	4.955981	2.711807
C	-1.062722	-3.232898	-0.356708
C	-8.663674	2.134990	0.038511
H	-9.095165	2.935624	-0.569056
C	-1.383189	-3.362247	1.126037
H	-1.349498	-2.345395	1.545508
C	-6.915089	-0.823056	2.647154
H	-6.012305	-1.242137	2.177463
C	6.830077	-2.236139	0.867093
H	6.468808	-2.946396	0.116451
H	6.267751	-2.417166	1.790320
H	7.883877	-2.483901	1.071978
C	-3.087600	0.378862	2.363587
H	-4.121162	0.121039	2.102415
H	-3.112108	1.184902	3.115580
H	-2.599367	-0.496796	2.821277
C	3.512500	-1.575603	3.013184
H	3.722620	-0.582704	2.582542
C	-6.760372	-2.447453	-0.419678
H	-6.235466	-3.020881	0.360150
H	-7.328815	-3.164701	-1.034594
H	-7.484077	-1.781965	0.066130
C	-0.595389	-4.340404	-2.478099
H	-0.456369	-5.261643	-3.049584
C	-0.687197	-0.589509	-3.163878
H	-0.629114	0.218565	-2.413700
C	-6.558055	-0.870990	-2.354906
H	-7.285453	-0.194656	-1.889573
H	-7.115987	-1.559521	-3.011171
H	-5.884741	-0.275461	-2.991280
C	-0.509865	-3.104297	-3.105374
H	-0.308708	-3.066010	-4.179222
C	4.372023	-2.861493	-1.914098
H	4.689632	-1.807522	-1.935621
C	-6.552749	2.438806	-1.298140
H	-5.664014	1.827507	-1.517034
C	-0.913604	1.235709	1.556204
H	-0.369186	0.376433	1.984361
H	-0.974838	1.999074	2.346209
H	-0.339361	1.706366	0.739802
C	-4.947876	-2.723816	-2.070546
H	-4.255624	-2.277157	-2.798356
H	-5.640308	-3.367317	-2.637261
H	-4.368233	-3.378521	-1.404035
C	-3.492438	-0.874295	-0.480968
H	-3.221592	-1.678502	-1.157954
C	-7.299087	2.666073	-2.617521
H	-7.687647	1.728073	-3.039966
H	-8.152004	3.352659	-2.492755
H	-6.628728	3.121249	-3.364964
C	-6.061776	3.782244	-0.736857
H	-5.511157	3.648073	0.204949
H	-5.396098	4.292364	-1.453448
H	-6.911744	4.453502	-0.530733
C	-7.822458	-1.995185	3.036087
H	-8.179152	-2.550117	2.156027
H	-7.280083	-2.702339	3.684783
H	-8.708425	-1.659497	3.599011
C	-6.469423	-0.060647	3.904171
H	-5.931185	-0.724161	4.601757
H	-5.806134	0.778527	3.650503
H	-7.339965	0.354918	4.438040
C	2.431224	2.203895	3.422928
H	3.139265	1.831829	4.180664
H	1.592242	2.684777	3.951005
H	2.019647	1.330660	2.895464
C	3.720744	4.349158	3.251105
H	2.965942	4.864375	3.866046
H	4.500173	3.983922	3.938662
H	4.178803	5.101196	2.592164
C	2.107609	-1.546042	3.630314
H	2.033641	-0.771933	4.409232
H	1.316641	-1.345001	2.887997
H	1.851269	-2.509328	4.098034
C	4.554723	-1.822517	4.112271
H	4.489197	-1.045709	4.890796
H	4.398004	-2.796537	4.602935
H	5.579615	-1.812603	3.715191
C	5.523455	-3.704733	-2.477683
H	6.429859	-3.640370	-1.858995
H	5.248504	-4.768715	-2.553411
H	5.785659	-3.364350	-3.492095
C	3.145284	-3.000748	-2.821776
H	2.277486	-2.430652	-2.456142
H	3.366049	-2.657098	-3.844996
H	2.809308	-4.047614	-2.883950
C	-2.796395	-3.909564	1.364202
H	-2.903412	-4.929432	0.958783
H	-3.014740	-3.956292	2.443461
H	-3.553175	-3.267019	0.894716
C	-0.350502	-4.212248	1.872561
H	0.676751	-3.856825	1.705627
H	-0.547895	-4.191949	2.956649
H	-0.384490	-5.268166	1.558778
C	-0.241097	3.013911	-2.958498
H	-0.511811	4.056647	-3.184622
H	-0.254806	2.473644	-3.915477
H	-1.029163	2.593048	-2.317273
C	2.176428	3.559626	-3.263975
H	2.083730	3.135414	-4.276527

H	2.016884	4.646962	-3.339524
H	3.201946	3.384155	-2.920484
C	0.499410	-0.453640	-4.125294
H	0.487967	0.526464	-4.625206
H	1.476364	-0.558658	-3.624076
H	0.472594	-1.215253	-4.919829
C	-2.008275	-0.375071	-3.916125
H	-2.857721	-0.381198	-3.219865
H	-2.008274	0.591591	-4.444963
H	-2.166854	-1.168832	-4.664250

181

Ba-Ho_closed

Ba	-0.000006	0.020695	-0.000045
N	2.455323	-1.263323	1.106117
N	-2.455441	-1.263101	-1.106251
N	-2.500791	1.191623	1.178181
N	2.500846	1.191526	-1.178175
C	-1.490717	-2.148401	-3.215634
C	1.490557	-2.148450	3.215538
C	-1.993886	2.288485	1.895664
C	-1.917255	-2.325844	-1.858320
C	3.740714	-1.013119	0.899935
C	-3.776939	0.951340	0.913455
C	-5.009796	1.644148	1.646451
C	-1.922529	3.563524	1.237575
C	0.873615	-3.218305	3.881262
H	0.547600	-3.066618	4.915077
C	-0.873833	-3.218335	-3.881283
H	-0.547799	-3.066733	-4.915105
C	0.680475	-4.456774	3.288518
H	0.209424	-5.274781	3.838755
C	-6.010789	-2.218995	-0.630282
H	-5.557490	-2.791731	0.191210
H	-6.731492	-2.877015	-1.141609
H	-6.593445	-1.405324	-0.177169
C	1.670712	-3.590664	1.226312
C	1.994037	2.288489	-1.895573
C	-1.083017	-4.622155	-1.970347
H	-0.930587	-5.590411	-1.483053
C	-0.838196	3.279909	3.795188
H	-0.421426	3.173647	4.801916
C	4.954109	-1.722061	1.646968
C	5.009881	1.643931	-1.646397
C	-1.592240	0.963841	4.161968
H	-0.626006	0.930040	4.694799
C	-4.954279	-1.721657	-1.647081
C	-4.240848	-0.028454	-0.004338
H	-5.320800	-0.031149	-0.022327
C	1.317803	4.640478	-1.886735
H	1.275886	5.611889	-1.392360
C	-6.053379	2.158601	0.628541
H	-5.602575	2.841300	-0.108259
H	-6.837028	2.719629	1.162508
H	-6.559103	1.358465	0.071889
C	-5.677845	0.569780	2.533626
H	-6.021578	-0.296884	1.953189
H	-6.549677	0.999636	3.054149
H	-4.983360	0.197272	3.301402
C	-4.709981	2.842903	2.562531
H	-3.992378	2.618288	3.356132
H	-5.652688	3.139956	3.050236
H	-4.339918	3.719673	2.017191
C	-1.670938	-3.590470	-1.226305
C	3.776978	0.951134	-0.913468
C	1.459097	2.161696	-3.210743
C	-3.740815	-1.012807	-0.900067
C	5.677778	0.569643	-2.533784
H	6.021444	-0.297156	-1.953508
H	6.549634	0.999493	-3.054271
H	4.983222	0.197339	-3.301593
C	-0.760711	4.507542	3.158170
H	-0.275691	5.357681	3.643831
C	-5.632319	-0.672587	-2.556306
H	-6.487614	-1.129687	-3.081174
H	-4.940759	-0.289675	-3.320013
H	-6.005344	0.191451	-1.989929
C	-2.578993	3.766501	-0.125271
H	-3.596738	3.351493	-0.044799
C	1.917076	-2.326003	1.858236
C	-0.680752	-4.456771	-3.288451
H	-0.209740	-5.274840	-3.838632
C	-1.458962	2.161546	3.210825
C	-1.317235	4.640353	1.886912
H	-1.275153	5.611777	1.392577
C	1.922868	3.563507	-1.237419
C	1.082747	-4.622271	1.970429
H	0.930280	-5.590556	1.483204
C	2.579306	3.766322	0.125464
H	3.596919	3.350973	0.045086
C	1.693828	-0.924315	4.125087
H	0.741443	-0.850450	4.677571
C	-4.622264	-2.930321	-2.537394
H	-4.200573	-3.772520	-1.979010
H	-3.928956	-2.694172	-3.348487
H	-5.559051	-3.280282	-3.000889
C	4.710160	2.842876	-2.562259
H	3.992489	2.618480	-3.355861
H	5.652877	3.139898	-3.049962
H	4.340229	3.719597	-2.016749
C	-1.693890	-0.924313	-4.125273
H	-0.741486	-0.850560	-4.677740
C	4.621931	-2.930596	2.537393
H	3.928658	-2.694270	3.348468
H	5.558669	-3.280649	3.000917
H	4.200113	-3.772785	1.979087
C	0.761288	4.507823	-3.158012
H	0.276438	5.358072	-3.643649
C	1.592123	0.963997	-4.161929
H	0.625883	0.930436	-4.694765
C	6.010521	-2.219656	0.630192
H	5.557112	-2.792376	-0.191251
H	6.731112	-2.877766	1.141561
H	6.593318	-1.406125	0.177011
C	0.838543	3.280197	-3.795070
H	0.421760	3.174048	-4.801804
C	-2.028787	-4.007388	0.209200
H	-1.216656	-4.701385	0.481599
C	2.028611	-4.007717	-0.209138
H	1.216486	-4.701731	-0.481513
C	6.053564	2.158102	-0.628447
H	5.602862	2.840709	0.108501
H	6.837236	2.719154	-1.162352
H	6.559245	1.357819	-0.071969
C	5.632324	-0.673005	2.556078
H	6.005464	0.190924	1.989610
H	6.487561	-1.130182	3.080974
H	4.940834	-0.289918	3.319761
C	4.240819	-0.028750	0.004261
H	5.320768	-0.031498	0.022275
C	-2.013905	-2.946002	1.301961
H	-2.789519	-2.179353	1.188215
H	-2.151921	-3.431923	2.280093
H	-1.026133	-2.461299	1.360047
C	-3.312575	-4.847303	0.287114
H	-3.320164	-5.649766	-0.466346
H	-3.394209	-5.317947	1.280353
H	-4.214105	-4.238971	0.138670
C	-2.741950	5.231707	-0.530801
H	-3.329287	5.296339	-1.459836
H	-3.267711	5.820139	0.237138
H	-1.772689	5.716755	-0.728329
C	-1.889756	2.978342	-1.239663
H	-0.818942	3.221817	-1.305674
H	-2.033569	1.902902	-1.074216
H	-2.336969	3.209434	-2.217994
C	-1.799852	-0.421808	3.565519
H	-0.997168	-0.702324	2.869394
H	-1.781008	-1.176199	4.368352
H	-2.751364	-0.519341	3.031091
C	-2.646562	1.220312	5.254086
H	-2.512773	2.203139	5.729686
H	-3.669574	1.179921	4.853096
H	-2.572186	0.451219	6.040020
C	2.646504	1.220272	-5.254037
H	2.512959	2.203159	-5.729580
H	3.669509	1.179599	-4.853057
H	2.571930	0.451242	-6.040016
C	1.799409	-0.421726	-3.565535
H	1.780417	-1.176078	-4.368401
H	2.750885	-0.519497	-3.031088
H	0.996644	-0.702093	-2.869443
C	2.013796	-2.946428	-1.301996
H	2.789383	-2.179752	-1.188259
H	2.151895	-3.432434	-2.280075
H	1.026018	-2.461751	-1.360200
C	3.312397	-4.847655	-0.286906
H	4.213930	-4.239325	-0.138459
H	3.319934	-5.650055	0.466621
H	3.394080	-5.318383	-1.280101
C	1.889712	2.978468	1.239856
H	0.818976	3.222319	1.305767
H	2.033174	1.902967	1.074503
H	2.336921	3.209485	2.218207
C	2.742722	5.231496	0.530924
H	1.773607	5.716881	0.728342
H	3.330003	5.295981	1.460004
H	3.268747	5.819708	-0.237003
C	1.905706	0.440533	3.485836
H	1.927859	1.213933	4.270340
H	2.836832	0.514460	2.913075
H	1.077398	0.717088	2.817821
C	2.758534	-1.168835	5.210036
H	2.607510	-2.131009	5.721213
H	3.777842	-1.167049	4.800384
H	2.709649	-0.372613	5.970498
C	-1.905678	0.440601	-3.486136
H	-1.927753	1.213935	-4.270709
H	-2.836808	0.514651	-2.913399
H	-1.077363	0.717148	-2.818129
C	-2.758579	-1.168836	-5.210239
H	-2.709611	-0.372672	-5.970757
H	-2.607605	-2.131057	-5.721342
H	-3.777901	-1.166949	-4.800625

2. References

[S1] S. Meiries, G. Le Duc, A. Chartoire, A. Collado, K. Speck, K. S. A. Arachchige, A. M. Z. Slawin, S. P. Nolan, *Chem. Eur. J.,* 2013, **19**, 17358.

[S2] B. Rösch, T. X. Gentner, J. Langer, C. Färber, J. Eyselein, L. Zhao, C. Ding, G. Frenking, S. Harder, *Science,* 2021, **371**, 1125.

[S3] J. Hicks, M. Juckel, A. Paparo, D. Dange, C. Jones. *Organometallics*, 2018, **37** (24), 4810.

[S4] D. M. Ottmers, H. F. Rase, *Carbon,* 1966, **4**, 125.

[S5] P. J. Bailey, R. A. Coxall, C. M. Dick, S. Fabre, L. C. Henderson, C. Herber, S. T. Liddle, D. Loroño-González, A. Parkin, S. Parsons, *Chem. Eur. J.,* 2003, **9**, 4820.

[S6] P. H. M. Budzelaar, A. B. van Oort, and A. G. Orpen, *Eur. J. Inorg. Chem*., 1998, 1485.

[S7] B. Rösch, T. X. Gentner, J. Eyselein, A. Friedrich, J. Langer, S. Harder, *Chem. Commun*., 2020, **56**, 11402.

[S8] H. M. El-Kaderi, M. J. Heeg, C. H. Winter, *Polyhedron*, 2006, **2**, 224.

[S9] H. Lehmkuhl, A. Shakoor, K. Mehler, C. Krüger, K. Angermund, Y.-H. Tsay, *Chem. Ber*. 1985, **118**, 4239.

[S10] J. Novotny, S. Komorovsky, R. Marek, *Acc. Chem. Res.,* 2024, **57**, 10, 1467.

[S11] T. Müntener, D. Joss, D. Häussinger, S. Hiller, Marek, *Chem. Rev*., 2022, **122**, 10, 9422.

[S12] a) Rigaku Oxford Diffraction, 2018, CrysAlisPro Software system, version 1.171.39.46, Rigaku Corporation, Wroclaw, Poland ([{(^{DIPeP}BDI*)Ba}$_2$(η^6:η^6-C_6H_6)], [{(^{DIPP}BDI*)Ba}$_2$(η^6:η^6-$C_{16}H_{10}$)]); b) Rigaku Oxford Diffraction, 2019, CrysAlisPro Software system, version 1.171.40.53, Rigaku Corporation, Wroclaw, Poland (compounds [(^{DIPeP}BDI*)K]$_4$, [(^{DIPeP}BDI*)Ba(μ-I)]$_2$); c) Rigaku Oxford Diffraction, 2019, CrysAlisPro Software system, version 1.171.40.67a ([(^{DIPeP}BDI*)Ca(μ-I)]$_2$); d) Rigaku Oxford Diffraction, 2020, CrysAlisPro Software system, version 1.171.41.93a, Rigaku Corporation, Wroclaw, Poland ([(^{DIPeP}BDI*)CaI·THF], [{(^{DIPeP}BDI*)Ca}$_2$(η^6:η^6-C_6H_6)]); e) Rigaku Oxford Diffraction, 2022, CrysAlisPro Software system, version 1.171.42.72a, Rigaku Corporation, Wroclaw, Poland ([(^{DIPeP}BDI*)Sr(μ-I)]$_2$ ([{(^{DIPeP}BDI*)Sr}$_2$(η^6:η^6-C_6H_6)],).

[S13] R. C. Clark and J. S. Reid, *Acta Crystallogr., Sect. A: Found. Crystallogr.,* 1995, **51**, 887.

[S14] O. V. Dolomanov, L. J. Bourhis, R.J. Gildea, J. A. K. Howard, H. Puschmann, *J. Appl. Cryst.,* 2009, **42**, 339.

[S15] G. M. Sheldrick, *Acta Crystallogr., Sect. A: Found. Adv.,* 2015, **71**, 3.

[S16] G. M. Sheldrick, *Acta Crystallogr., Sect. C: Struct. Chem.,* 2015, **71**, 3.

[S17] A. Thorn, B. Dittrich and G. M. Sheldrick, *Acta Crystallogr., Sect. A: Found. Crystallogr.* 2012, **68**, 448.

[S18] P. van der Sluis, A. L. Spek, *Acta Crystallogr., Sect. A: Found. Crystallogr.* 1990, **46**, 194.

[S19] J. Bolton, *Mol. Phys.* 1963, **6**, 219.

[S20] Gaussian 16, Revision A.03, Frisch, M. J.; Trucks, G. W.; Schlegel, H. B.; Scuseria, G. E.; Robb, M. A.; Cheeseman, J. R.; Scalmani, G.; Barone, V.; Petersson, G. A.; Nakatsuji, H.; Li, X.; Caricato, M.; Marenich, A. V.; Bloino, J.; Janesko, B. G.; Gomperts, R.; Mennucci, B.; Hratchian, H. P.; Ortiz, J. V.; Izmaylov, A. F.; Sonnenberg, J. L.; Williams-Young, D.; Ding, F.; Lipparini, F.; Egidi, F.; Goings, J.; Peng, B.; Petrone, A.; Henderson, T.; Ranasinghe, D.; Zakrzewski, V. G.; Gao, J.; Rega, N.; Zheng, G.; Liang, W.; Hada, M.; Ehara, M.; Toyota, K.; Fukuda, R.; Hasegawa, J.; Ishida, M.; Nakajima, T.; Honda, Y.; Kitao, O.; Nakai, H.; Vreven, T.; Throssell, K.; Montgomery, J. A., Jr.; Peralta, J. E.; Ogliaro, F.; Bearpark, M. J.; Heyd, J. J.; Brothers, E. N.; Kudin, K. N.; Staroverov, V. N.; Keith, T. A.; Kobayashi, R.; Normand, J.; Raghavachari, K.; Rendell, A. P.; Burant, J. C.; Iyengar, S. S.; Tomasi, J.; Cossi, M.; Millam, J. M.; Klene, M.; Adamo, C.; Cammi, R.; Ochterski, J. W.; Martin, R. L.; Morokuma, K.; Farkas, O.; Foresman, J. B.; Fox, D. J. Gaussian, Inc., Wallingford CT, 2016.

[S21] A. D. Becke, *J. Chem. Phys.,* 1993, **98**, 5648.

[S22] J. P. Perdew, J. A. Chevary, S. H. Vosko, K. A. Jackson, M. R. Pederson, D. J. Singh, C. Fiolhais, *Phys. Rev. B* 1993, **48**, 4978.

[S23] S. Grimme, J. Antony, S. Ehrlich, H. Krieg, *J. Chem. Phys.,* 2010, **132**, 154104.

[S24] S. Grimme, S. Ehrlich, L. E. Goerigk, *J. Comp. Chem.*, 2011, **32**, 1456.

[S25] F. Weigend, R. Ahlrichs, *Phys. Chem. Chem. Phys.,* 2005, **7**, 3297.

[S26] NBO 7.0, E. D. Glendening, J. K. Badenhoop, A. E. Reed, J. E. Carpenter, J. A. Bohmann, C. M. Morales, P. Karafiloglou, C. R. Landis, F. Weinhold, Theoretical Chemistry Institute, University of Wisconsin, Madison, WI, 2018.

[S27] K. Momma, F. Izumi, *J. Appl. Crystallogr.,* 2011, **44**, 1272.

[S28] T. X. Gentner, B. Rösch, G. Ballmann, J. Langer, H. Elsen, S. Harder, *Angew. Chem., Int. Ed.*, 2019, **58**, 607.

[S29] B. Rösch, T. X. Gentner, J. Langer, C. Farber, J. Eyselein, L. Zhao, C. Ding, G. Frenking, S. Harder, *Science*, 2021, **371**, 1125.

[S30] J. Mai, M. Morasch, D. Jędrzkiewicz, J. Langer, B. Rösch, S. Harder, *Angew. Chem. Int. Ed.,* 2023, **62**, e202212463.

[S31] S. Harder, *Organometallics,* 2002, **21**, 3782.
